# Gene bionetworks involved in the epigenetic transgenerational inheritance of altered mate preference: environmental epigenetics and evolutionary biology

**DOI:** 10.1186/1471-2164-15-377

**Published:** 2014-05-16

**Authors:** Michael K Skinner, Marina I Savenkova, Bin Zhang, Andrea C Gore, David Crews

**Affiliations:** Center for Reproductive Biology, School of Biological Sciences, Washington State University, Pullman, WA 99164-4236 USA; Department of Genetics & Genomic Sciences, Institute of Genomics and Multiscale Biology, Mount Sinai School of Medicine, New York, NY 10029 USA; Pharmacology and Toxicology, Austin, Texas; Section of Integrative Biology, University of Texas at Austin, Austin, TX 78712 USA

**Keywords:** Epigenetics, Brain, Networks, Evolution, Behavior

## Abstract

**Background:**

Mate preference behavior is an essential first step in sexual selection and is a critical determinant in evolutionary biology. Previously an environmental compound (the fungicide vinclozolin) was found to promote the epigenetic transgenerational inheritance of an altered sperm epigenome and modified mate preference characteristics for three generations after exposure of a gestating female.

**Results:**

The current study investigated gene networks involved in various regions of the brain that correlated with the altered mate preference behavior in the male and female. Statistically significant correlations of gene clusters and modules were identified to associate with specific mate preference behaviors. This novel systems biology approach identified gene networks (bionetworks) involved in sex-specific mate preference behavior. Observations demonstrate the ability of environmental factors to promote the epigenetic transgenerational inheritance of this altered evolutionary biology determinant.

**Conclusions:**

Combined observations elucidate the potential molecular control of mate preference behavior and suggests environmental epigenetics can have a role in evolutionary biology.

**Electronic supplementary material:**

The online version of this article (doi:10.1186/1471-2164-15-377) contains supplementary material, which is available to authorized users.

## Background

The current molecular paradigm for neo-Darwinian evolutionary biology is that random DNA sequence mutations, gene flow, and genetic drift promote phenotype variation that allows an adaptation event to facilitate natural selection [1]. Although environment has an important role in natural selection, environmental factors generally do not have the capacity to alter DNA sequence or mutation rates. A small group of compounds can act as mutagens, but the vast majority of nutritionally derived and environmental toxicants do not alter DNA sequence [2]. The current genetic paradigm does not completely explain many observations such as rapid evolutionary events, environmental impacts on evolution, and the low frequency of the occurrence of useful mutations [3, 4]. The realization that epigenetics provides an additional molecular mechanism for the environment to influence genome activity and biology has suggested a potential role for environmental epigenetics in evolutionary biology [5–11]. Charles Darwin recognized sexual selection as one of two determinants in evolutionary biology, the other being natural selection [12]. The physical attributes and courtship rituals involved in mate preference are essential for reproductive fitness and propagation of a species. The current study examines how environmental factors can promote an epigenetic event to promote an alteration in mate preference behavior.

Previously we demonstrated that exposure of a gestating female rat to an environmental compound during fetal gonadal sex determination promoted epigenetic reprogramming of the male germline [13–15]. These reprogrammed differential DNA methylation regions (DMR) in the sperm epigenome have recently been shown to be induced by a variety of different environmental toxicants with exposure specific DMR [16, 17]. The initial environmental compound used was the commonly used fungicide vinclozolin which is an anti-androgenic endocrine disruptor [18]. The primordial germ cells during migration down the genital ridge undergo a DNA methylation erasure that then upon gonadal sex determination the DNA re-methylation is initiated in a sex-specific manner [19]. Environmental exposures during this developmental stage modifies the epigenetic programming of the male germline that becomes re-programmed (imprinted-like) and promotes a transgenerational phenotypic variation and adult onset disease state in subsequent generations [13–16]. The epigenetic transgenerational inheritance of adult onset disease (i.e. after one year of age) in males includes infertility, prostate disease, kidney disease, immune abnormalities and spermatogenic defects [20, 21], and in females includes mammary tumor development, kidney disease, reproductive tissue abnormalities and pregnancy abnormalities [22]. This germline mediated epigenetic transgenerational inheritance of adult onset disease is mediated in part through alterations in the sperm epigenome [13, 14]. Since the germline establishes the base line epigenome of the organism, all tissues in both the female and male progeny including the brain appear to have altered tissue specific transgenerational epigenomes, transcriptomes and phenotypes [15, 21, 23–25].

Investigation of the epigenetic transgenerational inheritance of altered brain genome activity and behaviors previously demonstrated anxiety-like behavior increased in females and decreased in males, which correlate to alterations in specific brain region transcriptomes [23]. Altered stress responses are also detected in the transgenerational exposure lineage animals [26]. Interestingly, previous analysis of F3 generation control and vinclozolin lineage female and male rats (i.e. prior to the onset of disease) demonstrated an alteration in mate preference behavior [27]. The female rats, independent of control or vinclozolin lineage, prefer control lineage males if given a choice. This behavioral decision raises the possibility of an epigenetic contribution to mate preference and sexual selection. The current study was designed to directly correlate the altered mate preference behavior with gene networks in specific brain regions in both the females and males. Observations elucidate the potential molecular control of mate preference behavior and demonstrates environmental factors have the capacity to promote the epigenetic transgenerational inheritance of altered mate preference.

Systems biology analysis has allowed biological phenomena such as mate preference to be considered from the molecular to physiological level. The gene bionetwork [28] analysis previously developed to investigate the molecular basis of disease [29] was used in the current study. This approach has been used to identify gene networks associated with disease, such as obesity and diabetes [30]. Recently, we have used this bionetwork analysis to study a normal developmental process of primordial follicle development in the ovary [31, 32]. The gene networks identified were found to contain growth factors that are known to regulate the developmental process [31, 32]. These bionetwork analyses use a large number of microarray transcriptome analyses under different perturbations to identify gene clusters and modules that are coordinately regulated [33, 34]. The gene networks observed identify the genes with the highest level of integration and connection (i.e. connectivity) that associate with the phenotype [29, 33–35]. This genomic approach was used in the current study to identify the gene bionetworks in various brain regions associated with mate preference.

Observations demonstrate an environmental compound exposure can induce an epigenetic reprogramming of the germline that promotes epigenetic transgenerational inheritance of altered mate preference behavior. Although no direct epigenetic modifications in the brain were examined, the environmentally induced epigenetic transgenerational model used indicates epigenetics can be involved in the induction of the altered behavioral phenotypes. Sex-specific effects were observed in both the male and female brain transcriptome and behavior correlations. The gene networks in specific brain regions that statistically correlate with various mate preference behaviors provides insight into this environmentally modified transgenerational behavior. This systems biology approach has elucidated novel mechanisms to be considered in mate preference biology.

## Results

**Figure 1:**
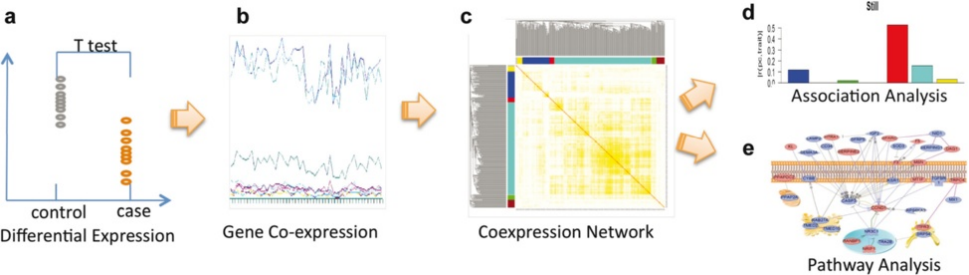
**A flowchart of the analyses carried out in the study. (a)** Differentially expressed (DE) genes are identified by the standard *t*-test. **(b)** Correlations between the expression profiles of the DE genes are calculated to quantify the level of coexpression. **(c)** Coexpression network analysis is performed to identify coexpressed gene modules. A matrix of correlations between gene expression profiles is first transformed through a power function into an adjacency matrix that is further transformed into a topological overlap matrix (TOM). Modules (represented by color bars) comprised of highly coexpressed genes are the identified using a dynamic cut-tree algorithm. **(d)** Association between gene modules and phenotypic traits is then accessed. **(e)** Pathway analysis is then performed on the gene modules of interest to derive regulatory networks for mechanism discovery.

The experimental design involved the development of transgenerational control and vinclozolin lineage animals for a mate preference behavioral analysis [27]. Subsequently, a transcriptome analysis was performed on 6 different brain regions from adult male and female F3 generation Sprague Dawley rats. These brain regions have previously been shown to be associated with mate preference behavior [27, 36]. The transcriptome alterations were statistically correlated with changes in mate preference behaviors. As previously described [13, 37], F0 generation gestating females were transiently exposed daily to vehicle control DMSO or vinclozolin from embryonic day 8–14 (E8-14) during fetal gonadal sex determination. The F1 generation offspring were bred at 90 days of age to generate F2 generation control and vinclozolin lineage progeny and then F2 generation animals were bred to generate the F3 generation control and vinclozolin lineage animals [13]. No sibling or cousin breeding was used to avoid any inbreeding artifacts. The F3 generation control and vinclozolin male and female rats were analyzed at 3–4 months of age for mate preference behaviors, as previously described [27]. This is an age when no major adult onset disease has been detected or is anticipated [20]. Later at 11 month of age, animals were sacrificed and specific brain regions isolated and RNA collected for microarray transcriptome analysis. The differentially regulated gene sets (“Signature lists”) for each brain region were identified. Subsequently a bioinformatics bionetwork analysis [23, 31] was used to correlate gene modules and networks with mate preference behaviors observed (Figure 1).

Females of both control and vinclozolin lineages preferred control lineage males over vinclozolin lineage males [27]. The mate preference behaviors are described in detail in the Methods [27] (Additional file 1: Movie S1). The specific behavioral components associated with mate preference analysis include the following: “Wire Mesh” involved the experimental animal investigating the stimulus animals directly through the Wire Mesh; “Facial Investigation” entailed the actual nose-to-nose contact; “Plexiglas” refers to the experimental animal investigating the area immediately bordering the Wire Mesh that separated the experimental animal from the stimulus animal; “Walking” refers to general investigation of the central testing chamber as measured by undirected walking and sniffing; and “Still” in which the animal was stationary with minimal head movement. Additional file 2: Table S1A presents the values for each behavioral component associated with individual animals. After completion of the behavioral analysis the animals were sacrificed at 11 months of age and 6 different brain regions dissected including the amygdala (Amy), hippocampus (Hipp), olfactory bulb (OlfB), cingulate cortex (CngCtx), entorhinal cortex (EnCtx), and preoptic area-anterior hypothalamus (POAH). The procedure to isolate the brain regions is described in the Materials and Methods. The isolated tissue was immediately placed in Trizol reagent, frozen and stored. RNA was prepared for microarray transcriptome analysis from each animals brain regions independently.

For the microarray analysis each F3 generation control and vinclozolin lineage male and female animal had six different brain regions analyzed which totaled 134 different microarrays. The microarray data were pre-processed and demonstrated two abnormal arrays that were omitted for further analysis (Additional file 3: Figure S1B). Batch effect corrections were made for RNA preparation date and array scan date with no major batch effects detected. The array data were then processed as previously described [31] to identify the differentially expressed gene sets for each brain region (Table 1). The differentially expressed genes in the Signature lists required a greater than 1.2 fold change in expression and all changes in expression were statistically significant with p < 0.05, as described in the Methods. Since a 20% alteration in gene expression for many genes, such as transcription factors, can have dramatic cellular and biological responses [26, 32], a more stringent cut off (e.g. 2×) was not used in the current study. In the current study the primary focus was on the coexpression patterns of the differentially expressed genes through the co-expression network analysis.

**Table 1:** **Differentially expressed Signature genes and their overlap with modules generated in combined network**

Sex-region	Signature lists	Over-expressed	Under-expressed	Combined networks modules										Separate network modules						
				Number of modules	Turquoise	Blue	Brown	Yellow	Green	Red	Black	Pink	Magenta	Number of modules	Turquoise	Blue	Brown	Yellow	Green	Red
Female regions	1833	939	894	4	1090	283	259	104							# genes in module					
					# genes overlapped between module & Signature list															
F-Amy	139	38	101		81	19	15	0						3	71*	49	17			
F-CngCTX	803	481	322		640	16	57	83						4	444	183	82	74		
F-EnCTX	433	279	154		56	191	128	37						3	369*	35	10*			
F-Hipp	70	40	30		18	17	12	0						1	70					
F-OlfB	748	221	527		598	59	69	3						4	416	305	11	10*		
F-POAH	56	24	32		16	18	11	1						1	56*					
Male regions	1693	638	1055	9	505	287	222	155	88	66	50	40	36		# genes in module					
					# genes overlapped between module & Signature list															
M-Amy	175	105	70		35	19	15	3	25	8	10	8	13	2	160*	10*				
M-CngCTX	785	189	596		354	79	193	39	56	8	0	5	0	1	780*					
M-EnCTX	385	210	175		87	133	26	8	13	3	0	5	22	1	378*					
M-Hipp	151	30	121		13	27	0	11	2	41	9	21	0	2	133*	13*				
M-OlfB	356	278	78		71	33	0	114	16	9	47	7	9	6	231	65*	20*	12*	11*	11
M-POAH	43	19	24		3	13	1	4	1	0	1	7	0	1	43*					

*- modules that showed statistically significant correlation with behavior.

**Figure 2:**
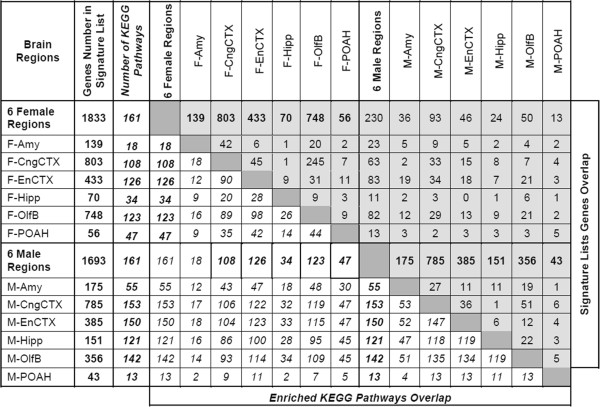
**Number of overlapped differentially expressed genes with pathways and Signature lists.** Number of genes overlapped between Signature lists is shown in regular font on grey background; number of affected KEGG pathways overlapped between Signature lists is shown in italicized font in white background; only KEGG pathways with 5 or more genes affected are counted.

The number of control lineage versus vinclozolin lineage differentially expressed genes in the Signature lists ranged from 43 to 803 with both up-regulated and down-regulated genes (Table 1). The total number of control versus vinclozolin lineage differentially expressed genes for all brain regions combined was 1833 for females and 1693 for males. A list of all the genes separated by brain region, sex and functional gene categories is presented in Additional file 4: Table S2 A-I. The overlap and differences between the Signature lists of each brain region for male and female is shown in Figure 2. The majority of genes were distinct to the different lists in a comparison of the brain regions. The one exception was an overlap between the cingulate cortex (CngCtx) and olfactory bulb (OlfB) in the female. Therefore, each brain region Signature list was distinct from each other and between the sexes.

Analysis of the cellular pathways and processes associated with the gene Signature lists for each brain region is shown in Figure 2 and Additional file 5. The top 36 pathways with the greatest combined number of genes associated are shown in Additional file 5. An extended list of pathways and processes with the associated genes from the different modules and tissues is presented in Additional file 6: Table S3. Several of the most highly represented pathways in the male and female were the MAPK signaling pathway, olfactory transduction, neuroactive ligand-receptor interactions and axon guidance. The Signature list genes distributed relatively evenly across the different pathways with no major over-representation identified. Most major cellular processes and pathways were represented with no major predominance of any individual specific regulatory mechanism observed (Additional file 6: Table S3).

**Figure 3:**
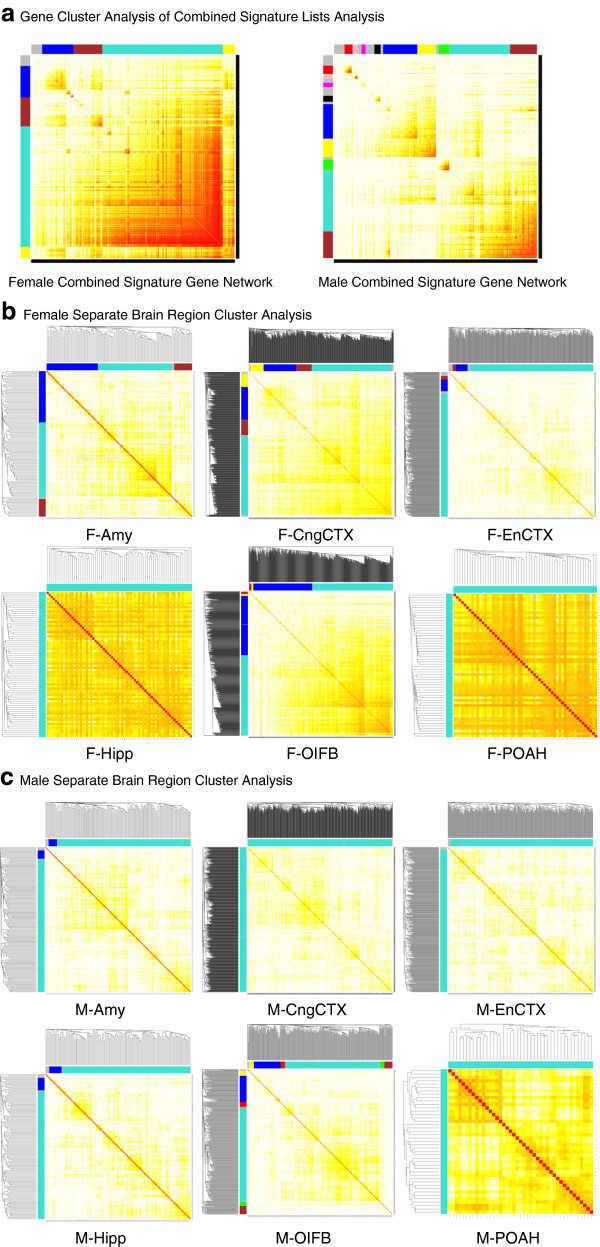
**Male and female brain region bionetwork cluster analysis and corresponding gene modules.** Topological overlap matrixes of the gene co-expression network consisting of genes differentially expressed in F3 generation vinclozolin lineage rat brain as compared to F3 generation lineage Control animals. Genes in the rows and columns are sorted by an agglomerative hierarchical clustering algorithm. The different shades of color signify the strength of the connections between the nodes (from white signifying not significantly correlated to red signifying highly significantly correlated). Modules identified are colored along both column and row and are boxed. **(a)** Matrixes of combined network for male and female brain regions. **(b)** Matrixes of separate network for female amygdala (F-Amy), cingulate cortex (F-CngCTX), enterorhinal cortex (F-EnCTX), hippocampus (F-Hipp), olfactory bulbs (F-OlfB), and preoptic area-anterior hypothalamus (F-POAH). **(c)** Matrixes of separate network for male amygdala (M-Amy), cingulate cortex (M-CngCTX), enterorhinal cortex (M-EnCTX), hippocampus (M-Hipp), olfactory bulbs (M-OlfB), and preoptic area-anterior hypothalamus (M-POAH).

A bionetwork cluster analysis was performed on the differentially expressed genes in the various brain regions as previously described [29, 31] to identify gene modules and networks with coordinated and interconnected relationships (i.e. connectivity) [38] (Figure 1). Initially all the differentially expressed genes in the combined brain regions for male or female were analyzed, termed combined networks (Figure 3a). This combined analysis was performed to potentially identify common gene networks or modules similar among all the brain regions that potentially correlate with the mate preference behavior parameters. The increased number of microarrays and data associated with the combined analysis also improves the power of the cluster and network analysis. The gene cluster analysis is shown and individual modules of genes identified are presented in different colors on the axis. The module colors represent increasing levels of connectivity [38] with white being negligible and red being highest. The combined Signature lists provided 4 modules in the female and 9 gene modules in the male. This can be seen as the blocked gene clusters designated as modules of different colors (Figure 3a). The number of genes in each module for male and female brain regions is shown in Table 1. This combined network analysis and modules were correlated with the mate preference behavior, but no significant correlations were found using this combined analysis (Additional file 7: Table S4).

Due to the distinct functions of each brain region and distinct gene Signature lists, the combined analysis was found not to provide the specificity needed to identify the behavioral correlations with gene modules. Therefore, a more specific network analysis using the individual Signature lists for each brain region separately was performed, termed separate networks. Each brain region differentially expressed gene Signature list was used for separate network analysis. The cluster analysis identified specific gene modules for each brain region from the male and female gene Signature lists presented (Figure 3b, c). The modularity for the specific brain regions was not as strong as the combined region analysis. Each separate brain region is shown and the gene modules are identified by the different colors. The brain regions had 1–6 different modules and associated gene networks (Table 1). The same differentially expressed gene Signature lists were used, but the network analysis was from the separate lists (Figure 3b and c). All subsequent analyses used the gene modules from this region specific network analyses.

**Figure 4:**
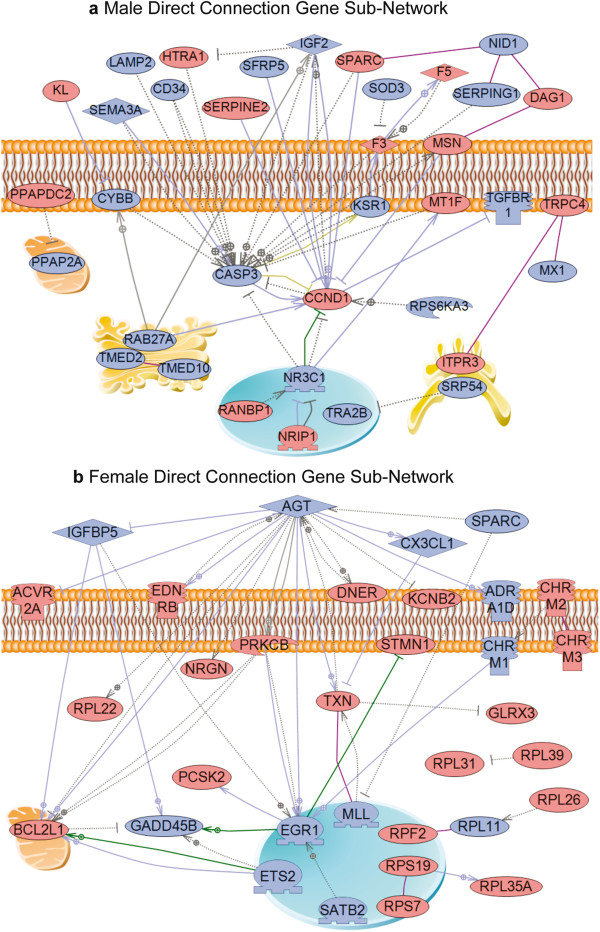
**Direct connection sub-networks for most highly top 10% connected genes from each module of separate network for male (a) or female (b).** Only directly connected genes are shown according to their location in the cell (on membrane, in Golgi apparatus, nucleus, cytoplasm or outside the cell). Node shapes and color code: oval and circle – protein; diamond – ligand; circle/oval on tripod platform – transcription factor; ice cream cone – receptor; crescent – kinase or protein kinase; irregular polygon – phosphatase; red color indicates up-regulated genes, blue – down-regulated. Arrows with plus sign show positive regulation/activation, arrows with minus sign – negative regulation/inhibition; grey arrows represent regulation, lilac - expression, purple – binding, green – promoter binding, and yellow – protein modification.

The cluster analysis (Figure 3b and c) for each brain region provided modules of genes with coordinated gene expression and identified a connectivity index [29–31, 33, 38] for each associated gene. The connectivity index (k.in) for each of the differentially expressed genes in each region is presented in Additional file 4: Table S2. The top 10% of genes with the highest connectivity index for each of the gene modules was identified (Additional file 4: Table S2 as the genes in bold font). From this combined list of 185 genes for male and 225 genes for female, a gene sub-network analysis was performed. The most highly interconnected genes in all modules for female and male brain regions were used to identify the common direct connection interactions between genes in a gene sub-network (Figure 4). The female gene sub-network identified angiogenesis, growth and apoptosis as predominant cellular processes affected (Figure 4b). The male gene sub-network identified apoptosis as a predominant pathway affected (Figure 4a). These gene networks identify the common connections within the brain regions with the most highly interconnected genes differentially expressed between the control and vinclozolin F3 generation animals.

**Figure 5:**
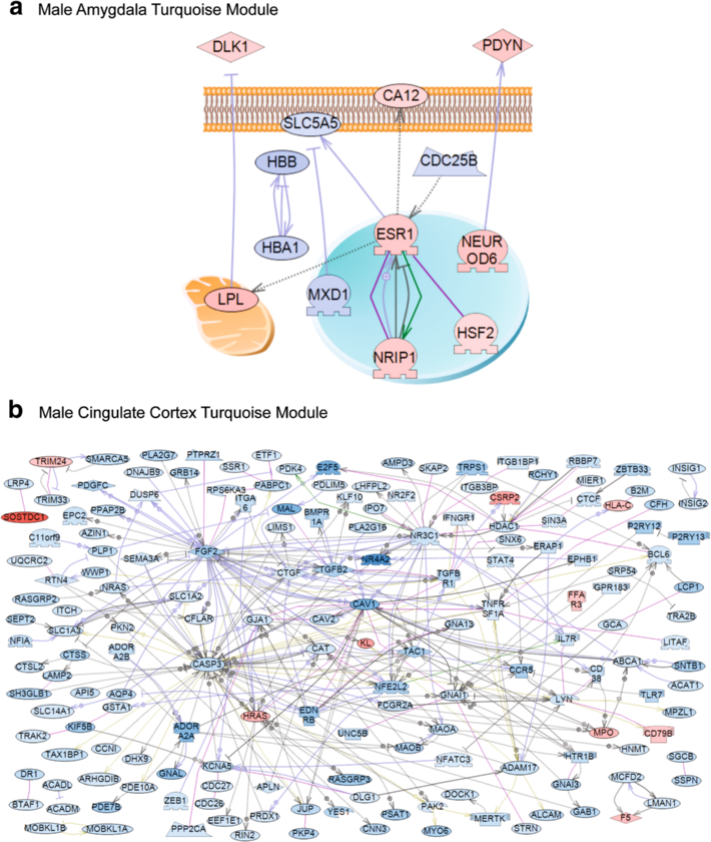
**Direct connection sub-networks for male amygdala turquoise module (a) and male cingulate cortex turquoise module (b).** Only directly connected genes are shown. Node and arrows shapes and color code is the same as for Figure 4.

Analysis of the gene networks for each individual brain region gene module demonstrated that only males exhibited direct connection gene sub-networks for gene modules (male amygdala and cingulate cortex turquoise modules) (Figure 5). This region specific examination of gene network modules demonstrated most regions did not have direct connection sub-networks, but indirect interactions with various pathways and processes. An alternative analysis used the entire Signature list for each brain region to identify region specific gene sub-networks (Additional file 8: Figure S2 A-K). How these gene networks may correlate with the alterations in mate preference behavior required a statistical correlation of the gene sets with the behaviors (Figure 1).

**Figure 6:**
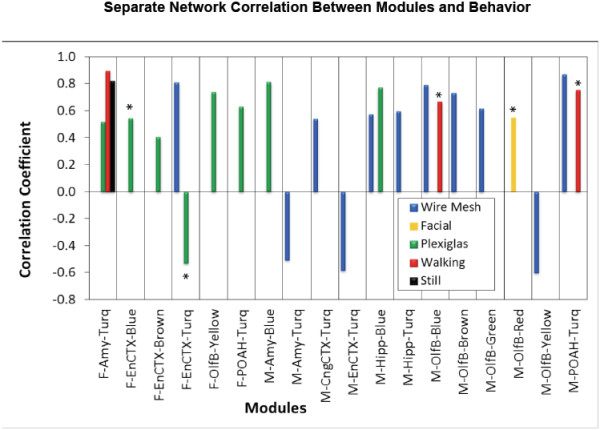
**Gene module correlation with mate preference behavior parameters.** Separate network male and female modules highly correlated to types of behavior: Wire Mesh (blue bars), Facial Investigation (orange), Plexiglas (green), Walking (red) and Still (black). Bars not marked with asterisks have p-value < 0.05; bars marked with one asterisk have correlation coefficient >0.5 and > p-value = 0.1 - 0.05.

In considering the mate preference behaviors, the female is the discriminatory sex to choose a mate, while the male is non-discriminatory and has phenotypes and behaviors to be selected [27, 39]. The altered gene expression and correlations with behaviors needs to consider this in data interpretation. The behavioral parameters (Additional file 2: Table S1) for the mate preference analysis were statistically correlated to the separate network gene modules for the different brain regions, (Additional file 9: Table S5). The correlation and the p-values associated with the statistical correlation coefficients are presented. All correlations with a single or multiple principle component comparison are presented. Considering a p < 0.05 or correlation coefficient >0.5 and p = 0.05-0.1 between the gene module and behavior demonstrated correlations in four female brain regions and six modules with the female behavior (Table 2 and Additional file 9: Table S5). A summary of the statistically significant correlations and/or those with strong correlation coefficients is shown in Figure 6. Nearly all the female brain regions had statistically significant correlation with the Plexiglas behavioral parameter. The female amygdala (F-Amy) had a turquoise module with significant correlation with the Walking and Still parameters. The turquoise modules of female entorhinal cortex (F-EnCtx) had a strong correlation with the Wire Mesh and Plexiglas behavioral parameters (Figure 6).

**Table 2:** **Gene modules highly correlated to different mate preference behavior parameters**

Sex-region	Behavior trait		Wire mesh		Facial		Plexiglas		Still		Walking	
	Module	# PC*	Correlation	p-value	Correlation	p-value	Correlation	p-value	Correlation	p-value	Correlation	p-value
**F-Amy**	Turquoise	2					0.52	**0.028**	0.82	**0.012**	0.90	**0.001**
**F-EnCTX**	Blue	1					0.55	**0.066**				
	Brown	1					0.41	**0.037**				
	Turquoise	3	0.81	**0.029**			−0.53	**0.083**				
**F-OlfB**	Yellow	2					0.74	**0.044**				
**F-POAH**	Turquoise	1					0.63	**0.0386**				
**M-Amy**	Blue	2					0.81	**0.008**				
	Turquoise	1	−0.51	**0.022**								
**M-CngCTX**	Turquoise	1	0.54	**0.048**								
**M-EnCTX**	Turquoise	1	−0.58	**0.037**								
**M-Hipp**	Blue	1	0.57	**0.023**			0.77	**0.017**				
	Turquoise	1	0.60	**0.034**								
**M-OlfB**	Blue	2	0.79	**0.012**							0.67	**0.072**
	Brown	1	0.73	**0.012**								
	Green	1	0.62	**0.020**								
	Red	1			0.55	**0.081**						
	Yellow	1	−0.60	**0.033**								
**M-POAH**	Turquoise	1	0.87	**0.002**							0.753	**0.091**

*- number of principal components (PC) used to calculate correlation between modules and behavior.

The six male brain regions and associated gene modules had a number of statistically significant correlations with the mate preference parameters (Table 2 and Additional file 9: Table S5). All the male brain regions had statistically significant correlation with at least one module and the Wire Mesh behavioral parameter. Amy and Hipp also had correlations with the behavioral Plexiglas parameter (Figure 6). Therefore, at least one gene module in nearly all brain regions statistically correlated to the mate preference parameters analyzed. These correlations can now be considered in regards to the regulatory roles of gene networks identified for mate preference behavior alterations for the female (chooser) versus the male (selected) (Figure 5 and Additional file 8: Figure S2).

The direct connection gene sub-networks for the critical male amygdala (M-Amy) and cingulate cortex (M-CngCtx) turquoise modules are shown in Figure 5. The Signature list for each brain region sub-networks demonstrate distinct networks for each region (Additional file 8: Figure S2). Since nearly all the brain regions and key modules (Figure 6) have a statistically significant correlation with the Wire Mesh for male or Plexiglas for female mate preference behavior parameters, the combined gene sub-network (Figure 4) for all male or female brain regions identifies a potentially associated molecular control of behavior. Alternately, the analysis of separate regions differentially expressed gene sets (Signature lists) identified distinct gene sub-networks that associate with the different regions (Additional file 8: Figure S2). These potential gene sub-networks correlate and potentially regulate the mate choice behavior for the female and selection behavior/phenotype for the male. In addition to the gene networks, a correlation of critical cellular pathways in specific brain regions and modules (Additional file 5) that are associated with the epigenetic transgenerational inheritance of altered mate preference behavior.

The epigenetic transgenerational inheritance of the altered mate preference behavior requires the transmission of an altered epigenome in the germline (sperm) [5, 13, 15]. Previously the altered DNA methylation of the F3 generation sperm was characterized with 48 differentially DNA methylated regions (DMR) being identified in gene promoters [14]. These vinclozolin induced sperm DMR are in part what promotes an altered epigenome in the embryo and all developing tissues transgenerationally [15]. Although any developing tissue (e.g. brain) will have a dramatic cascade of epigenetic and genetic steps to achieve an adult fully differentiated state [40, 41], the possibility that some of the original germline epigenetic marks (DMR) may persist was investigated. The genes associated with the 48 previously identified sperm DMR were compared with the male and female brain region gene Signature lists. The comparison demonstrated the majority of the DMR did not correspond to differentially expressed genes in various brain regions. Only Rnase1 in the male Amy, Ig6-2a in the male EnCtx, Parp9 in the female CngCtx and Rp132 in the female OlfB overlapped. Interestingly, a copy number variation (CNV) in the Fam111a site previously identified [14] was found in all brain regions identified with the epigenome analysis. This provides a positive control for the technology and ability to detect the DMR. Therefore, some of the original sperm DMR programmed sites may persist, but the vast majority of brain development and epigenetic programming, and potential distal regularity role of DMR in epigenetic control regions [25], is distinct from the original germline epigenetic marks.

## Discussion

A systems biology analysis of environmentally induced epigenetic transgenerational inheritance of altered mate preference behavior was performed to suggest a potential role for epigenetics in evolutionary biology. Previous research has demonstrated that environmental toxicants such as the fungicide vinclozolin can promote a reprogramming of the germline epigenome during fetal gonadal sex determination that then transmits altered phenotypes and adult onset disease states transgenerationally in the absence of future environmental exposure [41]. This is referred to as epigenetic transgenerational inheritance [5, 15] and suggests a role for environmental epigenetics in the inheritance of phenotypic variation and disease, independent of classic genetic inheritance mechanisms. The basic molecular mechanism involved in this non-genetic form of inheritance is the ability of environmental factors to influence the epigenetic programming of the germline [15, 19]. The primordial germ cells during migration down the genital ridge undergo an erasure of DNA methylation that then is initiated to re-methylate at the time of gonadal sex determination in a sex-specific manner [19]. An environmental toxicant such as vinclozolin appears to alter gonadal development to influence germline DNA methylation programming [42] and the differential DNA methylation regions (DMR) in the sperm become imprinted-like sites that appear to not get erased at fertilization so are transmitted to subsequent generations and male and female progeny [13–16]. In addition to vinclozolin, a number of other environmental toxicants such as the plastic compound bisphenol A (BPA) [16, 43], dioxin [16, 44], methoxycholor [13], phthalates [16], pesticides [16], hydrocarbons [16], and DDT [17] have been shown to induce transgenerational phenotypes. Other environmental factors such as nutrition and stress can also promote transgenerational phenotypes [45–48].

The vinclozolin induced epigenetic transgenerational phenotypes previously identified included adult onset rat disease after 12 months of age of male infertility, mammary tumors, prostate disease, kidney disease and immune abnormalities [20]. Therefore, the mate preference analysis was performed prior to adult onset disease to remove the disease as a confounding factor. A brain-behavior transgenerational phenotype observed was increased female anxiety and decreased male anxiety behaviors [23]. This transgenerational anxiety behavior was also examined on a molecular level to identify brain region specific changes in different gene expression and gene networks associated with the behavior [23]. Similar observations were made in the analysis of transgenerational stress responses [26]. Interestingly, in a previous study we found that vinclozolin induced alterations in mate preference behavior [27]. Females from either control or vinclozolin F3 generation lineages prefer control lineage males over vinclozolin lineage males, whereas no altered mate preference in males was observed [27]. While the standard argument would be that the females are the discriminating sex and distinguish between males on as yet undetermined phenotype characteristic(s), it is important to realize that the absence of evidence (in the male) does not mean the evidence of absence of male involvement since preference is only the first step in a mating sequence. That is, under natural circumstances this is followed by a mutual decision. Mating in rodents involves pheromone and auditory cues produced by both sexes and evidence suggests (see below) that it is under such unfettered conditions that the complementarity of behavior and brain are expressed.

Observations from the current study need to consider the effects on the female brain as potentially altering female discrimination and preference. The effects on the male brain are presumed to be associated with the characteristics (e.g. auditory cues and pheromone production) being selected. This altered mate preference behavior suggests the existence of an environmentally altered epigenetic transgenerational inheritance of mate preference behavior [15]. The current study was designed to identify the gene bionetworks in various male and female brain regions that correlate with the behavior of the transgenerational inheritance model.

A novel gene bionetwork analysis was developed to identify gene networks correlated to disease [29]. The approach was to use a large number of microarrays to identify transcriptomes in specific tissues associated with control versus disease individuals in large cohorts. Differentially regulated genes that are coordinately regulated and having connectivity [38] are clustered in large gene sets to identify modules of genes that associate with the disease [29, 30, 33–35] (Figure 1). More recently, we have used a similar approach to investigate a normal development process to identify gene bionetworks associated with development [31, 32]. The primordial follicle development in the ovary was investigated to identify a network of growth factors and associated signaling systems that regulate follicle development [31, 32]. This bioinformatics approach to identify regulatory gene networks was used in the current study to correlate brain gene networks to mate preference behavior (Figure 1) in an epigenetic transgenerational model [27]. The six different brain regions isolated from F3 generation control and vinclozolin lineage females and males were used in a microarray analysis to determine the differential gene expression in each brain region. The region specific gene sets, “Signature list”, and associated gene networks were investigated.

Analysis of significant pathways and cellular processes potentially influenced by the differentially expressed gene sets and networks did not identify predominant or over represented pathways. One pathway identified that previously has been shown to correlate with sexual selection is the olfactory transduction pathway [49–52]. However, most of the brain regions and specific networks or gene modules affected similar pathways with overlap between most. Therefore, no specific pathways were identified and most major pathways were influenced. A limitation in gene expression studies is that individual genes are assigned a specific function, but the gene may be involved in functional categories. This limitation needs to be considered in any gene expression data interpretation, but genome wide transcriptome analysis for gene sets has been shown to start to address this issue [53]. Combined observations suggest that the distinct differential expressed gene sets for the different brain regions appear to regulate common cellular processes and pathways among the brain regions and modules.

In contrast, analysis of gene networks identified unique gene sub-networks and gene modules associated with each brain region differentially expressed gene sets. The specific inter-connected genes were unique and overall networks of connected genes distinct. Therefore, the different functions associated with each brain region and associated with the altered mate preference behavior were identified. A statistical correlation of the gene modules for each brain region with the different mate preference parameters measured identified a number of statistically significant correlations. All but one female brain region (Hipp) had statistically significant correlations with the Plexiglas behavior parameter. The female behavior directly associates with the discrimination and mate preference choice. Interestingly, all the male brain regions had some modules with statistically significant correlations with the Wire Mesh behavior parameter. The male behavior and/or phenotype (e.g. pheromonal production) associates with the selected behaviors and characteristics of the non-discriminant sex. Therefore, direct correlations with the gene modules, specific brain regions and mate preference behavior parameters were identified. The specific gene modules and behavioral parameters statistically correlated were distinct between the sexes and brain regions, but strong correlations of the gene networks to the mate preference behavior was established. Interestingly, both the Plexiglas and Wire Mesh are indicators of interest and assessment of the stimulus animal.

**Figure 7:**
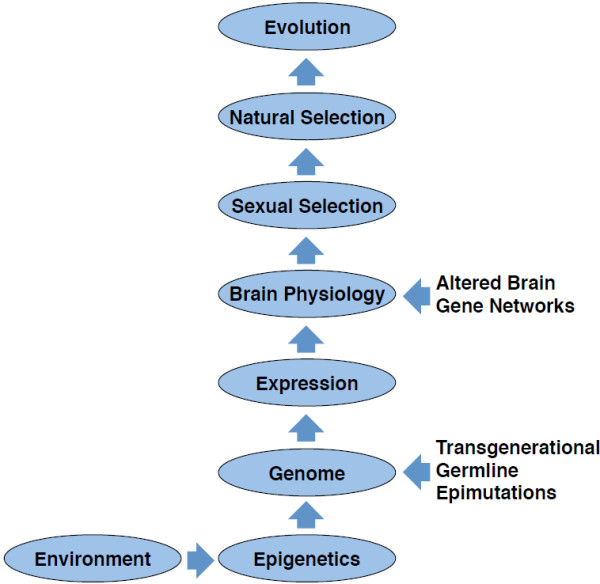
**Schematic of role of epigenetics in evolution.**

The gene bionetwork analysis and statistical correlation with the mate preference behavior provides insight into the molecular basis of how various male and female brain regions correlate and in part control the various behavioral parameters. Observations provide one of the first genomic and systems biology analysis of mate preference behavior (Figures 1 and 7). The experimental model used involved the ability of an environmental compound (vinclozolin) to induce an epigenetic modification of the germline (sperm) to promote epigenetic transgenerational inheritance of an altered mate preference behavior. This altered mate preference behavior was due to a baseline alteration in the epigenomes of all male and female tissues, including the brain, which are derived from the epigenetically altered germline [25]. The current study used a systems biology approach to help elucidate the molecular control of this process.

Darwin [12] considered natural selection and sexual selection as distinct processes driving the evolution of traits. Natural selection results in traits that are adaptive responses to changes in the environment. The resulting variation in traits between and within species is shaped by differential survivorship. In other words, animals that survive are those with traits that are adaptive to their environment [54]. Darwin [12] conceived of sexual selection as arising from aggressive interactions between males (male-male competition) and the female's selection of a mate (mate choice). Males compete amongst themselves for access to females. Aggression between males can have a direct effect on female reproduction by preventing other breeding males from having access to females or from harming the female. Importantly aggression amongst males can also have an indirect effect by inhibiting or suppressing the normal reproductive physiology of the female or even terminate a pregnancy [55].

Mate preference, in its simplest form, states that males compete for females and females choose between them. Although most research has focused on how females choose males, male choice of females is also important [56–58]. This point cannot be overemphasized. That is, in virtually all paradigms published to date, the choosing individual is the independent variable and the stimulus animal is the dependent variable. Although this study is similar to previous studies in that there are restrained stimulus animals and freely moving individuals that are the investigators, it differs in several important ways. First, the ‘round robin’ testing method insured that all males and all females served both as stimulus animals and experimental animals; thus, the “preferences” exhibited reflect both the males and the females. Second, this study extends to the molecular level events in the brain of the individuals, both of which have exhibited preferences, namely brain transgenerational transcriptome alterations that correlated to the opposing sex behaviors. In essence we are seeing the outcome of the complementary nature of mate choice. This has never been shown before.

Evolution favors reproductive success, and it is in the individual's interest to focus on selecting the best mate and to avoid mating with the wrong species [59, 60]. Making the correct choice of a mate has a pronounced impact on reproductive success of both partners. Except in unusual systems, in nature the mating partners choose one another [55, 57, 60]. Experiments with flies [61], birds [62], and rodents [63, 64] indicate that individuals who are allowed to select, and be selected by, their mate enjoy greater reproductive success than force-paired animals. This consent is based not only on the internal milieu that motivates each individual to seek a partner, but also on the satisfactory nature of the phenotypic traits the potential mate displays.

There are a number of sexual selection hypotheses, all of which emphasize that females choosing optimally will produce young whose viability and survivorship are enhanced by the female’s choice of mate [65]. The most attractive, and one that takes into account that mating is a cooperative act that involves both partners, is the sensory exploitation hypothesis [66]. This hypothesis postulates that males have evolved calls and/or pheromones to exploit the preexisting sensory biases in the female that themselves evolved for reasons independent of female choice. Male behavior then has changed to maximize stimulation of the female's sensory systems. Therefore, the coordination of the complementary signal and receiver, mounting and lordosis, coordination of egg and sperm maturation and release is required for successful completion for reproduction. These complementary processes are evident at all levels of biological organization [55, 67] and we extend it here to the level of the genome and epigenome.

## Conclusions

The ability of an environmental factor to alter mate preference behavior suggests a critical role of environment in evolutionary biology. This is distinct from the generally accepted role of the environment in natural selection where environment is the active factor in the selection of an adaptive phenotype, but alternately here it involves the induction of phenotypes that can be acted on by natural selection. Since the majority of environmental factors can not alter DNA sequence or promote mutagenesis [2], an additional molecular mechanism to consider involves environmental epigenetics [15]. Many environmental compounds and factors such as nutrition can modify the epigenome to alter phenotypic variation. The role of epigenetics in evolutionary biology has been suggested previously [5, 8–11, 15, 68], but no significant experimental evidence has been provided. The current study demonstrates an environmental factor can promote the epigenetic transgenerational inheritance of an altered mate preference behavior. The epigenetic modification of the germline (sperm) has been previously established [13, 14, 16] and will lead to epigenetic alterations in the brain transcriptomes of both females and males [23] to alter the mate preference behavior [27]. Therefore, the current study provides direct experimental evidence for a potential role of environmental epigenetics in evolution by regulating a critical determinant such as mate preference on a molecular level (i.e. altered gene networks) in specific brain regions in a sex-specific manner (Figure 7). Although no direct epigenetic alterations were examined in the brain, the germline (sperm) epigenetic alterations that generate this altered male or female brain development have been documented [13, 14, 16]. This molecular mechanism does not suggest genetics will not have a critical role in evolutionary biology, but suggests environmental epigenetics will be an additional mechanism to consider. Epigenetics provides a mechanism for the environment to impact phenotypic variation and natural selection. Epigenetic and genetic mechanisms will cooperate to regulate on a molecular basis evolutionary biology. This appears to be a “neo-Lamarckian concept to facilitate neo-Darwinian evolution” [40, 41].The systems biology approach used in the current study links an environmental exposure, epigenetic transgenerational inheritance and molecular regulation of brain function to mate preference and evolutionary biology (Figure 7). Epigenetics will have a central role in how environmental factors influence how the gene networks emerge to induce phenotypic variation. Although genetics is critical for all aspects of biology, epigenetics provides the plasticity to allow the environment to alter biological events. This type of systems approach to understand complex biological traits, such as sexual selection, provides insights into how the various components (environment, phenotype and evolution) interact in a systems biology manner.

## Methods

### Animal housing protocol

Male and female rats of the F3 generation of Vinclozolin (Vinclozolin-Lineage) and DMSO Control (Control-Lineage) Lineages were selected out of litters from untreated F2 generation mothers in Dr. Michael Skinner’s laboratory at Washington State University according to established protocols [13]. Briefly, gestating female F0 generation Sprague Dawley rats were injected with the fungicide vinclozolin (100 mg/kg) daily during fetal gonadal sex determination (E8-E14) and the F1 generation were bred to generate the F2 generation and then the F2 bred to generate the F3 generation [13]. At approximately PND 10 (before weaning), each animal was injected with a small microchip (AVID Identification system Inc. Norco, CA) subcutaneously between the shoulder blades. The animals were then shipped to the University of Texas from Washington State University on postnatal day (PND) 22, one day after weaning. Upon arriving at the University of Texas, one animal from each Lineage (Control and Vinclozolin) was pair-housed (one control and one vinclozolin animal) and remained in these dyads throughout the duration of the study. Because of the natural variation in dates of breeding, there was a 4-day spread of birth date of animals in the first cohort but in the second cohort, all animals were born on the same day. However, all pair-housed animals were no more than one day apart in birth age and were paired randomly to prevent an age effect on cagemates.

Each dyad of animals was randomly placed in a six-wide, five-high metal housing rack in standard translucent polycarbonate rat cages (46 × 24 × 20.5 cm) with *ad libitum* access to tap water and standard rat chow (Purina rodent chow #5LL2 Prolab RMH 1800 diet). The animal room was on a 14:10 light/dark schedule. For environmental enrichment, a 7 cm diameter PVC pipe was placed in each cage.

### Ethics statement

All experimental protocols for the procedures with rats were pre-approved by the Washington State University Animal Care and Use Committee (IACUC approval # 02568–026) and by the University of Texas at Austin Animal Care and Use Committee (Public Health Service Animal Welfare Assurance Number A4107-01).

### Behavioral testing

After habituation to the testing arena, each individual was tested individually (when used as an experimental subject) or in pairs (when used as stimulus animals) with all individuals; the order of the testing was rotated during the course of both the male and female trials. All tests were conducted during the dark phase of the light cycle, beginning at 1200 h, 4 h after the progesterone injection, in a room illuminated with low levels of red light. Before trials, to confirm that females were receptive, each female was placed with a sexually experienced but otherwise experimentally naïve male; all females exhibited robust lordosis (arched back and lifted head posture) in response to mounting by the male.

Partner preference tests consisted of placing an individual (male or female) in the center of a large three-chamber glass-testing arena (122 × 46 × 54 cm). At either end was a small compartment (28 × 28 × 12.5 cm) containing the stimulus rats separated by a Wire-mesh barrier to allow exchange of olfactory, visual, and tactile cues. The area directly in front of the stimulus cage was marked by tape. Tests were conducted 2 h after the onset of the dark cycle under red-light illumination and lasted 10 min; all tests were videotaped for further review and analysis. At the end of each test, all animals were removed, and the entire testing arena was washed with a household cleaner and then wiped down with 70% ethanol to remove scent marks and residual odors. All males were tested with both types of females as stimulus animals (72 trials), and all females were tested with both types of males as stimulus animals (72 trials) (Movie S1).

The videotaped trials were analyzed by using JWatcher v1.0 (http://www.jwatcher.ucla.edu) computer software to quantify the behavior of each experimental animal. Time spent with a stimulus animal was recorded as soon as all four paws of the experimental animal crossed over the line of tape marking the boundary of that stimulus animal’s compartment. As soon as one paw crossed over the tape back into the center compartment, the time recorded with the experimental animal was stopped. Preference behaviors were defined as those directed to the stimulus animal and included time spent in contact with the Wire Mesh separating the experimental and stimulus animal (Wire Mesh), during which the animals often touched noses through the Wire Mesh (facial investigation), and contacted the Plexiglas surface surrounding the front of the stimulus cage; the cumulative total time in these preference behaviors toward each stimulus animal was also calculated (Total). Other activity measured included undirected walking and sniffing (walking), standing still with minimal head movement (still). Videos demonstrating the test can be viewed as supporting information (Movie S1).

### Brain processing

The brain was removed in less than 1 minute and placed in crushed ice to chill. The brain was then cut in half in the sagittal plane along the midline. In all cases but one the right side was blocked and then 6 areas dissected (see list below) within 3–5 min. This procedure was done on iced tissue. The dissected brain areas were placed in chilled Trizol (150 l) in 1.5 ml Eppendorf tubes according to manufacturers specifications in each tube. No tissue fragment was more than 3 mm but in those instances, but where there were multiple fragments the amount of Trizol was doubled (approximately). After all animals were dissected, the Eppendorf tubes were vortexed (15 sec) and then frozen on dry ice. The brain regions collected were according to Paxinos & Watson [69]: olfactory bulbs (OlfB); cingulate cortex (CngCtx), anterior to POAH (Bregma 4.7 to 1.7); preoptic area-anterior hypothalamus (POAH), 4 mm rostral to anterior commissure (AC) (Bregma −0.26 to −1.40); amygdaloid nuclei (Amy), 3 mm caudal to AC (Bregma −2.3 to −3.6); hippocampus (Hipp), 6 mm caudal to AC (Bregma −2.12 to −4.52); entorhinal cortex (EnCtx) (Bregma −5.60 to −7.80).

### RNA preparation

Brain area samples from individual rats were homogenized in 150 μl Trizol and then 600 μl Trizol was added to final volume of 750 ml. Samples were stored at −80 or −20°C until RNA extraction. For microarray analysis, from 4 to 6 biological replicas (animals) were prepared as above for each brain area Control or Vinclozolin group depending on samples availability (Additional file 2: Table S1B). A total of 132 (67 Control and 65 Vinclozolin) samples/chips were analyzed: (6 brain areas) × (2 Male or Female) × (2 Control or Vinclozolin) × (4–6 biological replicas). RNA from individual animal brain area was extracted from Trizol samples according to standard Trizol extraction protocol (Invitrogen, USA) and stored in aqueous solution at −80°C until microarray analysis.

### Microarray analysis

The microarray analysis was performed by the Genomics Core Laboratory, Center for Reproductive Biology, Washington State University, Pullman, WA using standard Affymetrix reagents and protocol. Briefly, mRNA was transcribed into cDNA with random primers, cRNA was transcribed, and single-stranded sense DNA was synthesized which was fragmented and labeled with biotin. Biotin-labeled ssDNA was then hybridized to the Rat Gene 1.0 ST microarrays containing more than 30,000 transcripts (Affymetrix, Santa Clara, CA, USA). Hybridized chips were scanned on Affymetrix Scanner 3000. CEL files containing raw data were then pre-processed and analyzed with Partek Genomic Suite 6.5 software (Partek Incorporated, St. Louis, MO) using an RMA, GC-content adjusted algorithm. Raw data pre-processing was performed in 12 groups, one for each male or female brain area. Comparison of array sample histogram graphs for each group showed if data for all chips were similar and appropriate for further analysis (Additional file 3: Figure S1). By this criterion, 2 microarray samples (not counted in Additional file 4: Table S2B and not shown on Additional file 3: Figure S1) were omitted from repeated group pre-processing and further analysis.

The microarray quantitative data involves signals from an average of 28 different oligonucleotides (probes) arrayed for each transcript and many genes are represented on the chip by several transcripts. The hybridization to each probe must be consistent to allow a statistically significant quantitative measure of resulting gene expression signal. Therefore, the microarray provides an unbiased and highly stringent quantitative procedure compared to other protocols [70]. In contrast, a quantitative PCR procedure uses only two oligonucleotides and primer bias is a major factor in this type of analysis. Therefore, we did not attempt to use PCR based approaches as we feel the microarray analysis is more accurate and reproducible without primer bias such as PCR based approaches [31].

All microarray CEL files from this study have been deposited with the NCBI gene expression and hybridization array data repository GEO (GEO series accession number: GSE33830) and can be also accessed through http://www.skinner.wsu.edu. For gene annotation, Affymetrix annotation file RaGene1_0stv1.na31.rn4.transcript.csv was used.

### Network analysis

The network analysis was restricted to genes differentially expressed between the control and the treatment groups based on previously established criteria: (1) fold change of group means ≥ 1.2 or ≤ 0.83; (2) *T* test p-value ≤ 0.05. The union of the differentially expressed genes from the different treatments resulted in 1,693 genes for males and 1833 for females being identified and used for constructing a weighted gene co-expression network [71, 72]. Unlike traditional un-weighted gene co-expression networks in which two genes (nodes) are either connected or disconnected, the weighted gene co-expression network analysis assigns a connection weight to each gene pair using soft-thresholding and thus is robust to parameter selection. The weighted network analysis begins with a matrix of the Pearson correlations between all gene pairs, then converts the correlation matrix into an adjacency matrix using a power function *f(x) = x*^*β*^. The parameter *β* of the power function is determined in such a way that the resulting adjacency matrix (i.e., the weighted co-expression network) is approximately scale-free. To measure how well a network satisfies a scale-free topology, we use the fitting index proposed by Zhang & Horvath [71] (i.e., the model fitting index *R*^*2*^ of the linear model that regresses *log(p(k))* on *log(k)* where k is connectivity and *p(k)* is the frequency distribution of connectivity). The fitting index of a perfect scale-free network is 1.

To explore the modular structures of the co-expression network, the adjacency matrix is further transformed into a topological overlap matrix [73]. As the topological overlap between two genes reflects not only their direct interaction, but also their indirect interactions through all the other genes in the network. Previous studies [71, 73] have shown that topological overlap leads to more cohesive and biologically meaningful modules. To identify modules of highly co-regulated genes, we used average linkage hierarchical clustering to group genes based on the topological overlap of their connectivity, followed by a dynamic cut-tree algorithm to dynamically cut clustering dendrogram branches into gene modules [74]. Such networks were generated from all combined male or female differentially expressed genes (2 combined networks) or from each individual male or female brain region Signature lists (12 separate networks). From one to ten modules were identified in combined or separate networks and the module size was observed to range from 10 to 780 genes (Table 1).

To distinguish between modules, each module was assigned a unique color identifier, with the remaining, poorly connected genes colored grey. The hierarchical clustering over the topological overlap matrix (TOM) and the identified modules is shown (Figure 1). In this type of map, the rows and the columns represent genes in a symmetric fashion, and the color intensity represents the interaction strength between genes. This TOM heatmap highlights that genes in the transcriptional network fall into distinct network modules, where genes within a given module are more interconnected with each other (blocks along the diagonal of the matrix) than with genes in other modules. Therefore, there are two types of global connectivity, adjacency-based one and TO based one. The adjacency-based connectivity (*k.all*) is defined as the sum of the power-function transformed correlations between the gene *g* and all the other genes in the whole network while the TO-based connectivity (*to.all*) is defined as the sum of the topological overlaps between the gene *g* and all the other genes. By default, connectivity used throughout the paper refers to TO-based connectivity *to.all*.

Gene Co-expression Network Analysis Clarification: Gene networks provide a convenient framework for exploring the context within which single genes operate. Networks are simply graphical models comprised of nodes and edges. For gene co-expression networks, an edge between two genes may indicate that the corresponding expression traits are correlated in a given population of interest. Depending on whether the interaction strength of two genes is considered, there are two different approaches for analyzing gene co-expression networks: 1) an unweighted network analysis that involves setting hard thresholds on the significance of the interactions, and 2) a weighted approach that avoids hard thresholds. Weighted gene co-expression networks preserve the continuous nature of gene-gene interactions at the transcriptional level and are robust to parameter selection. An important end product from the gene co-expression network analysis is a set of gene modules in which member genes are more highly correlated with each other than with genes outside a module. Most gene co-expression modules are enriched for GO functional annotations and are informative for identifying the functional components of the network that are associated with disease [75].

This gene co-expression network analysis (GCENA) has been increasingly used to identify gene sub-networks for prioritizing gene targets associated with a variety of common human diseases such as cancer and obesity [38, 76–79]. One important end product of GCENA is the construction of gene modules comprised of highly interconnected genes. A number of studies have demonstrated that co-expression network modules are generally enriched for known biological pathways, for genes that are linked to common genetic loci and for genes associated with disease [33, 38, 71, 75–78, 80, 81]. In this way, one can identify key groups of genes that are perturbed by genetic loci that lead to disease, and that define at the molecular level disease states. Furthermore, these studies have also shown the importance of the hub genes in the modules associated with various phenotypes. For example, GCENA identified ASPM, a hub gene in the cell cycle module, as a molecular target of glioblastoma [78] and MGC4504, a hub gene in the unfolded protein response module, as a target potentially involved in susceptibility to atherosclerosis [77].

### Pathway analysis

Resulting lists of differentially expressed genes for each male or female brain area as well as for each module generated in the combined network and some generated in separate networks analysis were analyzed for KEGG (Kyoto Encyclopedia for Genes and Genome, Kyoto University, Japan) pathway enrichment using Pathway-Express, a web-based tool freely available as part of the Onto-Tools (http://vortex.cs.wayne.edu) [82] as well as KEGG website ‘Search Pathway’ tool (http://www.genome.jp/kegg/tool/search_pathway.html). Global literature analysis of various gene lists was performed using Pathway Studio 8.0 software (Ariadne Genomics, Inc., Rockville, MD).

## Electronic supplementary material

Additional file 1: Movie S1: The first 19 sec of a 10-min mate-preference trial is shown. The trial is conducted under dim red light during the nocturnal (active) phase of the rats' light cycle. At the beginning of the video, the male is in the center of the chamber. The chamber is demarcated into thirds by tape on its floor. A stimulus female can be seen at the far end of the apparatus; the other stimulus female is not visible due to the position of camera. The stimulus females are free-moving in their chambers, but they are separated from the male by a wire mesh that is bounded by Plexiglas barrier. This enables the animals to communicate by olfactory, pheromonal, or behavioral cues, but physical interaction is limited to touching across the wire mesh. The trial begins with the removal of a holding box that confines the male. The male can be seen moving into the zone in front of one stimulus female and then moving across the central portion of the cage to the other stimulus female (out of sight). Several behaviors of the male can be seen on the video such as sniffing, facial investigation, walking, and standing of the female. The male is also seen investigating the various parts of the chamber, including the wire mesh, surrounding Plexiglas partition, and the glass walls of the chamber. Behaviors were scored for each male toward each pair of opposite lineage (Control- or Vinclozolin-Lineage) stimulus females. (AVI 4 MB)

Additional file 2: Table S1: Behavior and Sample Information. (PDF 60 KB)

Additional file 3: Figure S1A: Samples Histograms After Pre-processing (Male). **Figure S1B.** Samples Histograms After Pre-Processing (Female). Figure S1. Sample histograms and box plots for male (S1A) female (S1B) microarray signal values after pre-processing with RMA, GCcontent adjusted algorithm. Plots for F3 generation control (red) and F3 generation vinclozolin (blue) chips for female amygdala (A), cingulate cortex (B), enterorhinal cortex (C), hippocampus (D), olfactory bulbs (E), and preoptic areaanterior hypothalamus (F). (PDF 4 MB)

Additional file 4: Table S2: Genes Differentially Expressed in F3 Generation Vinclozolin Versus Control Lineage Male and Female Rat Brain Regions. (PDF 618 KB)

Additional file 5: **Top cellular pathways affected by signature gene lists and chosen modules from separate networks.** (PDF 80 KB)

Additional file 6: Table S3: Pathways Affected Male and Female Brain Region Signature Gene Lists and Chosen Modules from Separate Networks. (PDF 54 KB)

Additional file 7: Table S4: Correlation between combined network modules and behavior trait for F3-Vinclozolin rat brain regions. (PDF 2 MB)

Additional file 8: Figure S2: (Color) Brain Region Specific Signature List Direct Connection Gene Sub-Networks. Legend: Figure S2. Direct connection sub-networks for signature lists:female amygdala (A), female preoptic area-anterior hypothalamus(B), female hippocampus (C), female enterorhinal cortex (D), female cingulate cortex(E), female olfactory bulbs (F), male amygdala (G), male hippocampus (H), male cingulate cortex(I), male enterorhinal cortex (J), male olfactory bulbs (K) obtained by global literature analysis using Pathway Studio 8.0 software (Ariadne Genomics, Inc., Rockville, MD). Numbers in brackets on figures subtitles indicate number of genes in the list. Only directly connected genes are shown. Some sub-networks (G, H, J) show gene location in the cell (on membrane, in Golgi apparatus, nucleus, cytoplasm or outside the cell). Node shapes and color code: oval and circle – protein; diamond – ligand; circle/oval on tripod platform – transcription factor; ice cream cone – receptor; crescent – kinase or protein kinase; irregular polygon – phosphatase; red color indicates up-regulated genes, blue – down-regulated. Arrows with plus sign show positive regulation/activation, arrows with minus sign – negative regulation/inhibition; grey arrows represent regulation, lilac - expression, purple – binding, green – promoter binding, and yellow – protein modification. (PDF 66 KB)

Additional file 9: Table S5: Correlation between separate network modules and behavior trait for F3-Vinlozolin rat brain regions. (PDF 66 KB)

## Abbreviations

DMR: Differential DNA methylation regions
E8-E14: Embryonic day 8–14
CngCtx: Cingulate cortex
OlfB: Olfactory bulb
k.in: Connectivity index
Amy: Amygdala
EnCtx: Entorhinal cortex
Hipp: Hippocampus
POAH: Preoptic area-anterior hypothalamus
BPA: Bisphenol A.

## References

[1] Charlesworth B, Charlesworth D (2009). Darwin and genetics. Genetics.

[2] Stein RA (2012). Epigenetics and environmental exposures. J Epidemiol Community Health.

[3] Kalinowski ST (2002). Evolutionary and statistical properties of three genetic distances. Mol Ecol.

[4] Wilkins AS (2007). Genetic networks as transmitting and amplifying devices for natural genetic tinkering. Novartis Found Symp.

[5] Jirtle RL, Skinner MK (2007). Environmental epigenomics and disease susceptibility. Nat Rev Genet.

[6] Crews D, McLachlan JA (2006). Epigenetics, evolution, endocrine disruption, health, and disease. Endocrinology.

[7] Damiani G (2007). The Yin and Yang of anti-Darwinian epigenetics and Darwinian genetics. Riv Biol.

[8] Day T, Bonduriansky R (2011). A unified approach to the evolutionary consequences of genetic and nongenetic inheritance. Am Nat.

[9] Kuzawa CW, Thayer ZM (2011). Timescales of human adaptation: the role of epigenetic processes. Epigenomics.

[10] Flatscher R, Frajman B, Schonswetter P, Paun O (2012). Environmental heterogeneity and phenotypic divergence: can heritable epigenetic variation aid speciation?. Genet Res Int.

[11] Klironomos FD, Berg J, Collins S (2013). How epigenetic mutations can affect genetic evolution: model and mechanism. Bioessays.

[12] Darwin C (1871). The Descent of Man, and Selection in Relation to Sex.

[13] Anway MD, Cupp AS, Uzumcu M, Skinner MK (2005). Epigenetic transgenerational actions of endocrine disruptors and male fertility. Science.

[14] Guerrero-Bosagna C, Settles M, Lucker BJ, Skinner MK (2010). Epigenetic transgenerational actions of vinclozolin on promoter regions of the sperm epigenome. PLoS One.

[15] Skinner MK, Manikkam M, Guerrero-Bosagna C (2010). Epigenetic transgenerational actions of environmental factors in disease etiology. Trends Endocrinol Metab.

[16] Manikkam M, Guerrero-Bosagna C, Tracey R, Haque MM, Skinner MK (2012). Transgenerational actions of environmental compounds on reproductive disease and epigenetic biomarkers of ancestral exposures. PLoS One.

[17] Skinner MK, Manikkam M, Tracey R, Nilsson E, Haque MM, Guerrero-Bosagna C (2013). Ancestral DDT exposures promote epigenetic transgenerational inheritance of obesity. BMC Med.

[18] Kelce WR, Gray LE, Wilson EM (1998). Antiandrogens as environmental endocrine disruptors. Reprod Fertil Dev.

[19] Morgan HD, Santos F, Green K, Dean W, Reik W (2005). Epigenetic reprogramming in mammals. Hum Mol Genet.

[20] Anway MD, Leathers C, Skinner MK (2006). Endocrine disruptor vinclozolin induced epigenetic transgenerational adult-onset disease. Endocrinology.

[21] Anway MD, Skinner MK (2008). Transgenerational effects of the endocrine disruptor vinclozolin on the prostate transcriptome and adult onset disease. Prostate.

[22] Nilsson EE, Anway MD, Stanfield J, Skinner MK (2008). Transgenerational epigenetic effects of the endocrine disruptor vinclozolin on pregnancies and female adult onset disease. Reproduction.

[23] Skinner MK, Anway MD, Savenkova MI, Gore AC, Crews D (2008). Transgenerational epigenetic programming of the brain transcriptome and anxiety behavior. PLoS One.

[24] Anway MD, Rekow SS, Skinner MK (2008). Transgenerational epigenetic programming of the embryonic testis transcriptome. Genomics.

[25] Skinner MK, Manikkam M, Haque MM, Zhang B, Savenkova M (2012). Epigenetic transgenerational inheritance of somatic transcriptomes and epigenetic control regions. Genome Biol.

[26] Crews D, Gillette R, Scarpino SV, Manikkam M, Savenkova MI, Skinner MK (2012). Epigenetic transgenerational inheritance of altered stress responses. Proc Natl Acad Sci USA.

[27] Crews D, Gore AC, Hsu TS, Dangleben NL, Spinetta M, Schallert T, Anway MD, Skinner MK (2007). Transgenerational epigenetic imprints on mate preference. Proc Natl Acad Sci USA.

[28] Friend SH (2010). The need for precompetitive integrative bionetwork disease model building. Clin Pharmacol Ther.

[29] Schadt EE, Lamb J, Yang X, Zhu J, Edwards S, Guhathakurta D, Sieberts SK, Monks S, Reitman M, Zhang C, Lum PY, Leonardson A, Thieringer R, Metzger JM, Yang L, Castle J, Zhu H, Kash SF, Drake TA, Sachs A, Lusis AJ (2005). An integrative genomics approach to infer causal associations between gene expression and disease. Nat Genet.

[30] Yang X, Deignan JL, Qi H, Zhu J, Qian S, Zhong J, Torosyan G, Majid S, Falkard B, Kleinhanz RR, Karlsson J, Castellani LW, Mumick S, Wang K, Xie T, Coon M, Zhang C, Estrada-Smith D, Farber CR, Wang SS, van Nas A, Ghazalpour A, Zhang B, Macneil DJ, Lamb JR, Dipple KM, Reitman ML, Mehrabian M, Lum PY, Schadt EE (2009). Validation of candidate causal genes for obesity that affect shared metabolic pathways and networks. Nat Genet.

[31] Nilsson EE, Savenkova MI, Schindler R, Zhang B, Schadt EE, Skinner MK (2010). Gene bionetwork analysis of ovarian primordial follicle development. PLoS One.

[32] Nilsson E, Zhang B, Skinner MK (2013). Gene bionetworks that regulate ovarian primordial follicle assembly. BMC Genomics.

[33] Zhu J, Zhang B, Smith EN, Drees B, Brem RB, Kruglyak L, Bumgarner RE, Schadt EE (2008). Integrating large-scale functional genomic data to dissect the complexity of yeast regulatory networks. Nat Genet.

[34] Millstein J, Zhang B, Zhu J, Schadt EE (2009). Disentangling molecular relationships with a causal inference test. BMC Genet.

[35] Pandey G, Zhang B, Chang AN, Myers CL, Zhu J, Kumar V, Schadt EE (2010). An integrative multi-network and multi-classifier approach to predict genetic interactions. PLoS Comput Biol.

[36] DiBenedictis BT, Ingraham KL, Baum MJ, Cherry JA (2012). Disruption of urinary odor preference and lordosis behavior in female mice given lesions of the medial amygdala. Physiol Behav.

[37] Anway MD, Memon MA, Uzumcu M, Skinner MK (2006). Transgenerational effect of the endocrine disruptor vinclozolin on male spermatogenesis. J Androl.

[38] Ghazalpour A, Doss S, Zhang B, Wang S, Plaisier C, Castellanos R, Brozell A, Schadt EE, Drake TA, Lusis AJ, Horvath S (2006). Integrating genetic and network analysis to characterize genes related to mouse weight. PLoS Genet.

[39] Sakata JT, Crews D (2004). Developmental sculpting of social phenotype and plasticity. Neurosci Biobehav Rev.

[40] Skinner MK (2011). Role of epigenetics in developmental biology and transgenerational inheritance. Birth Defects Res C Embryo Today.

[41] Skinner MK (2011). Environmental epigenetic transgenerational inheritance and somatic epigenetic mitotic stability. Epigenetics.

[42] Skinner M, Guerrero-Bosagna C, Haque MM, Nilsson E, Bhandari R, McCarrey J (2013). Environmentally induced transgenerational epigenetic reprogramming of primordial germ cells and subsequent germline. PLoS One.

[43] Salian S, Doshi T, Vanage G (2009). Impairment in protein expression profile of testicular steroid receptor coregulators in male rat offspring perinatally exposed to Bisphenol A. Life Sci.

[44] Bruner-Tran KL, Osteen KG (2011). Developmental exposure to TCDD reduces fertility and negatively affects pregnancy outcomes across multiple generations. Reprod Toxicol.

[45] Waterland RA, Travisano M, Tahiliani KG, Rached MT, Mirza S (2008). Methyl donor supplementation prevents transgenerational amplification of obesity. Int J Obes (Lond).

[46] Painter RC, Osmond C, Gluckman P, Hanson M, Phillips DI, Roseboom TJ (2008). Transgenerational effects of prenatal exposure to the Dutch famine on neonatal adiposity and health in later life. BJOG.

[47] Pembrey ME, Bygren LO, Kaati G, Edvinsson S, Northstone K, Sjostrom M, Golding J (2006). Sex-specific, male-line transgenerational responses in humans. Eur J Hum Genet.

[48] Dias BG, Ressler KJ (2014). Parental olfactory experience influences behavior and neural structure in subsequent generations. Nat Neurosci.

[49] Buck LB (2004). Olfactory receptors and odor coding in mammals. Nutr Rev.

[50] Brennan PA, Zufall F (2006). Pheromonal communication in vertebrates. Nature.

[51] Dulac C, Wagner S (2006). Genetic analysis of brain circuits underlying pheromone signaling. Annu Rev Genet.

[52] Green RE, Krause J, Briggs AW, Maricic T, Stenzel U, Kircher M, Patterson N, Li H, Zhai W, Fritz MH, Hansen NF, Durand EY, Malaspinas AS, Jensen JD, Marques-Bonet T, Alkan C, Prufer K, Meyer M, Burbano HA, Good JM, Schultz R, Aximu-Petri A, Butthof A, Hober B, Hoffner B, Siegemund M, Weihmann A, Nusbaum C, Lander ES, Russ (2010). A draft sequence of the Neandertal genome. Science.

[53] Kim SY, Kim YS (2008). A gene sets approach for identifying prognostic gene signatures for outcome prediction. BMC Genomics.

[54] Williams G (1966). Adaptation and Natural Selection: A Critique of Some Current Evolutionary Thought.

[55] Crews D, Becker JBM, McCarthy M, Crews D (1992). Diversity of hormone-behavior relations in reproductive behavior. Introduction to Behavioral Endocrinology.

[56] Crews D (1998). The evolutionary antecedents to love. Psychoneuroendocrinology.

[57] Gowaty PA, Anderson WW, Bluhm CK, Drickamer LC, Kim YK, Moore AJ (2007). The hypothesis of reproductive compensation and its assumptions about mate preferences and offspring viability. Proc Natl Acad Sci USA.

[58] Mattle B, Wilson AB (2009). Body size preferences in the pot-bellied seahorse Hippocampus abdominalis: choosy males and indiscriminate females. Behav Ecol Sociobiol.

[59] Carson HL (1987). The contribution of sexual behavior to Darwinian fitness. Behav Genet.

[60] Carson HL (2003). Mate choice theory and the mode of selection in sexual populations. Proc Natl Acad Sci U S A.

[61] Gowaty PA, Steinichen R, Anderson WW (2002). Mutual interest between the sexes and reproductive success in Drosophila pseudoobscura. Evolution.

[62] Stunden CE, Bluhm CK, Cheng KM, Rajamahendran R (1999). Factors affecting reproductive performance in captive Mallard ducks. Theriogenology.

[63] Drickamer LC, Gowaty PA, Holmes CM (2000). Free female mate choice in house mice affects reproductive success and offspring viability and performance. Anim Behav.

[64] Drickamer LCGP, Wagner DM (2003). Free mutual mate preferences in house mice affect reproductive success and offspring performance. Animal Behav.

[65] Kirkpatrick M, Ryan MJ (1991). The evolution of mating preferences and the paradox of the lek. Nature.

[66] Ryan M (1990). Sexual selection, sensory systems, and sensory exploitation. Oxford Survey Evol Biol.

[67] Beach F, Potter RWJ (1979). Animal models for human sexuality. Sex, Hormones and Behaviour.

[68] Crews D (2005). Evolution of neuroendocrine mechanisms that regulate sexual behavior. Trends Endocrinol Metab.

[69] Paxinos G, Watson C (2007). The Rat Brain in Stereotaxic Coordinates.

[70] Bosotti R, Locatelli G, Healy S, Scacheri E, Sartori L, Mercurio C, Calogero R, Isacchi A (2007). Cross platform microarray analysis for robust identification of differentially expressed genes. BMC Bioinforma.

[71] Zhang B, Horvath S (2005). A general framework for weighted gene co-expression network analysis. Stat Appl Genet Mol Biol.

[72] Zhu J, Wiener MC, Zhang C, Fridman A, Minch E, Lum PY, Sachs JR, Schadt EE (2007). Increasing the power to detect causal associations by combining genotypic and expression data in segregating populations. PLoS Comput Biol.

[73] Ravasz E, Somera AL, Mongru DA, Oltvai ZN, Barabasi AL (2002). Hierarchical organization of modularity in metabolic networks. Science.

[74] Langfelder P, Zhang B, Horvath S (2008). Defining clusters from a hierarchical cluster tree: the dynamic tree cut package for r. Bioinformatics.

[75] Lum PY, Chen Y, Zhu J, Lamb J, Melmed S, Wang S, Drake TA, Lusis AJ, Schadt EE (2006). Elucidating the murine brain transcriptional network in a segregating mouse population to identify core functional modules for obesity and diabetes. J Neurochem.

[76] Chen Y, Zhu J, Lum PY, Yang X, Pinto S, MacNeil DJ, Zhang C, Lamb J, Edwards S, Sieberts SK, Leonardson A, Castellini LW, Wang S, Champy MF, Zhang B, Emilsson V, Doss S, Ghazalpour A, Horvath S, Drake TA, Lusis AJ, Schadt EE (2008). Variations in DNA elucidate molecular networks that cause disease. Nature.

[77] Gargalovic PS, Imura M, Zhang B, Gharavi NM, Clark MJ, Pagnon J, Yang WP, He A, Truong A, Patel S, Nelson SF, Horvath S, Berliner JA, Kirchgessner TG, Lusis AJ (2006). Identification of inflammatory gene modules based on variations of human endothelial cell responses to oxidized lipids. Proc Natl Acad Sci U S A.

[78] Horvath S, Zhang B, Carlson M, Lu KV, Zhu S, Felciano RM, Laurance MF, Zhao W, Qi S, Chen Z, Lee Y, Scheck AC, Liau LM, Wu H, Geschwind DH, Febbo PG, Kornblum HI, Cloughesy TF, Nelson SF, Mischel PS (2006). Analysis of oncogenic signaling networks in glioblastoma identifies ASPM as a molecular target. Proc Natl Acad Sci U S A.

[79] Emilsson V, Thorleifsson G, Zhang B, Leonardson AS, Zink F, Zhu J, Carlson S, Helgason A, Walters GB, Gunnarsdottir S, Mouy M, Steinthorsdottir V, Eiriksdottir GH, Bjornsdottir G, Reynisdottir I, Gudbjartsson D, Helgadottir A, Jonasdottir A, Styrkarsdottir U, Gretarsdottir S, Magnusson KP, Stefansson H, Fossdal R, Kristjansson K, Gislason HG, Stefansson T, Leifsson BG, Thorsteinsdottir U, Lamb JR, Gulcher (2008). Genetics of gene expression and its effect on disease. Nature.

[80] Schadt EE, Molony C, Chudin E, Hao K, Yang X, Lum PY, Kasarskis A, Zhang B, Wang S, Suver C, Zhu J, Millstein J, Sieberts S, Lamb J, GuhaThakurta D, Derry J, Storey JD, Avila-Campillo I, Kruger MJ, Johnson JM, Rohl CA, van Nas A, Mehrabian M, Drake TA, Lusis AJ, Smith RC, Guengerich FP, Strom SC, Schuetz E, Rushmore TH (2008). Mapping the genetic architecture of gene expression in human liver. PLoS Biol.

[81] Zhu J, Zhang B, Schadt EE (2008). A systems biology approach to drug discovery. Adv Genet.

[82] Draghici S, Khatri P, Tarca AL, Amin K, Done A, Voichita C, Georgescu C, Romero R (2007). A systems biology approach for pathway level analysis. Genome Res.

