# Supplementary material for: Gene bionetworks involved in the epigenetic transgenerational inheritance of altered mate preference: environmental epigenetics and evolutionary biology
Source: BMC Genomics. 2014 May 16;15(1):377. doi: 10.1186/1471-2164-15-377 (PMC4073506; doi:10.1186/1471-2164-15-377)
Supplement: Supplementary file 2 — Additional file 2: Table S1: Behavior and Sample Information. (PDF 60 KB) [file 12864_2013_6162_MOESM2_ESM.pdf]

Supplemental Table S1. Behavior and Sample Information

A. Behavior Parameters for F3 generation Vinclozolin and F3 Generation Control Lineage Animals

Sample Name	Animal ID	Cage Side	Cage/ Dyad	Wire Mesh	Facial Invest.	Plexiglas	Still	Walking
F-Con-1	SK8	Left	1	43.20	15.28	8.92	21.96	24.22
F-Con-2	SK12	Right	2	-3.73	-4.90	7.04	31.46	3.01
F-Con-3	SK11	Right	3	4.76	-1.72	1.99	-13.81	-3.31
F-Con-4	SK9	Right	4	8.43	1.12	-0.83	13.37	25.98
F-Con-5	SK7	Right	5	11.65	10.95	8.06	37.83	9.60
F-Con-6	SK10	Left	6	12.74	-0.86	5.90	-6.76	32.18
F-Vin-1	SK1	Left	1	5.05	-2.02	-0.24	52.22	7.96
F-Vin-2	SK2	Left	2	10.62	5.87	10.06	14.65	9.22
F-Vin-3	SK3	Left	3	-6.84	-1.48	-0.41	12.94	-10.29
F-Vin-4	SK4	Left	4	12.38	-0.99	1.66	61.44	1.78
F-Vin-5	SK5	Left	5	34.37	14.97	9.05	29.62	18.08
F-Vin-6	SK6	Left	6	4.53	0.31	-2.41	22.13	8.44
M-Con-7	SK300	Left	7	10.34	-0.62	-4.42	0.89	3.70
M-Con-8	SK1000	Left	8	-14.94	0.48	-9.54	-24.08	-3.82
M-Con-9	SK900	Left	9	13.26	-0.23	-1.16	43.37	43.41
M-Con-10	SK400	Left	10	7.89	-4.71	4.09	10.25	7.13
M-Con-11	SK600	Left	11	30.77	-5.15	0.83	-24.93	-5.16
M-Con-12	SK700	Left	12	-7.07	4.38	-6.92	-1.42	-16.49
M-Vin-7	SK100	Left	7	0.34	0.08	-0.21	28.16	16.48
M-Vin-8	SK200	Right	8	3.95	-1.23	-4.60	32.05	36.93
M-Vin-9	SK500	Left	9	-14.74	-1.54	-1.42	2.99	-4.07
M-Vin-10	SK800	Left	10	-19.87	-4.14	1.48	4.04	-10.53
M-Vin-11	SK1100	Left	11	-1.59	-1.84	0.99	1.98	-19.04
M-Vin-12	SK1200	Left	12	-1.07	4.20	-7.75	1.05	18.71

Note (F) female, (M) male, (Con) control lineage, (Vin) vinclozolin lineage

B. Microarray Samples Number by Brain Region

Sex	Region	Con	Vin
**Female**	Amy	5	6
	CngCTX	6	6
	EnCTX	6	6
	Hipp	4	5
	OlfB	6	5
	POAH	5	6
**Male**	Amy	6	6
	CngCTX	6	5
	EnCTX	6	4
	Hipp	6	6
	OlfB	6	6
	POAH	5	4
