# Supplementary material for: Gene bionetworks involved in the epigenetic transgenerational inheritance of altered mate preference: environmental epigenetics and evolutionary biology
Source: BMC Genomics. 2014 May 16;15(1):377. doi: 10.1186/1471-2164-15-377 (PMC4073506; doi:10.1186/1471-2164-15-377)

# Supplemental Figure S1A: Samples Histograms After Pre-processing (Male)

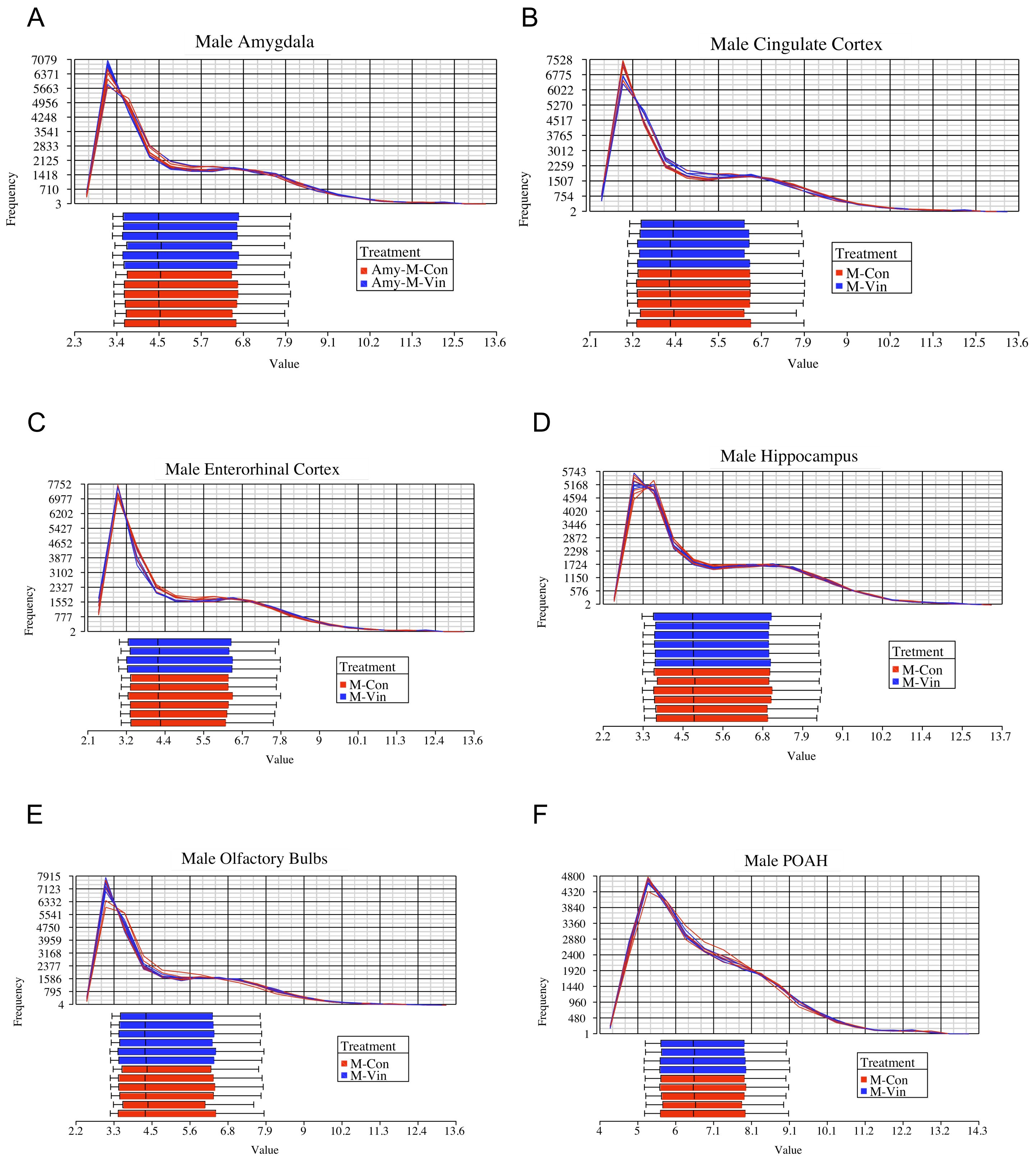

# Supplemental Figure S1B: Samples Histograms After Pre-Processing (Female)

A

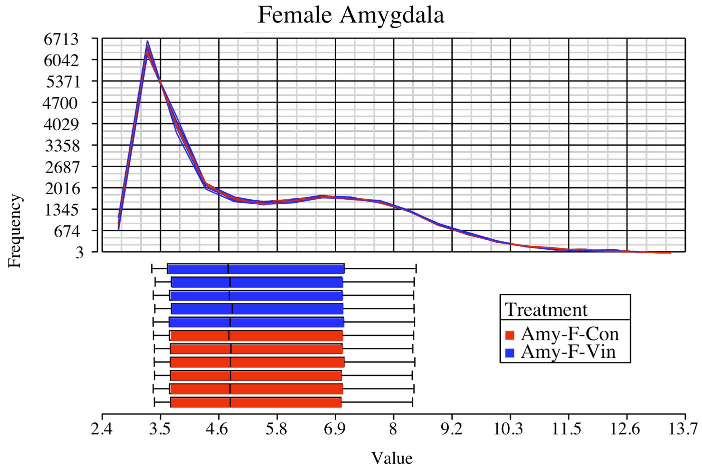


B

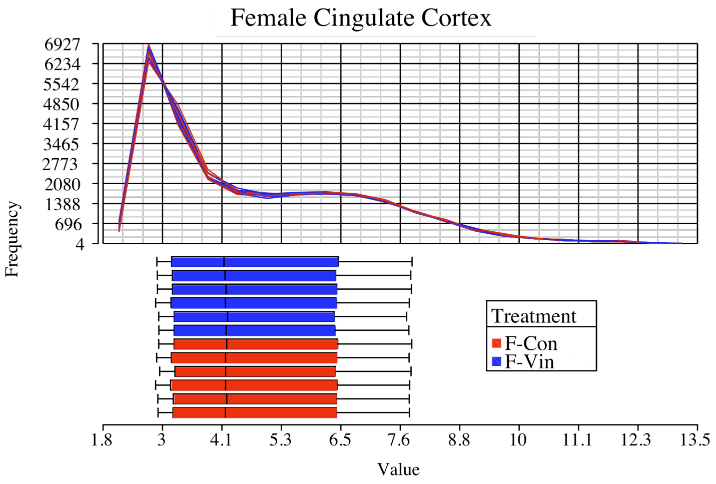


C

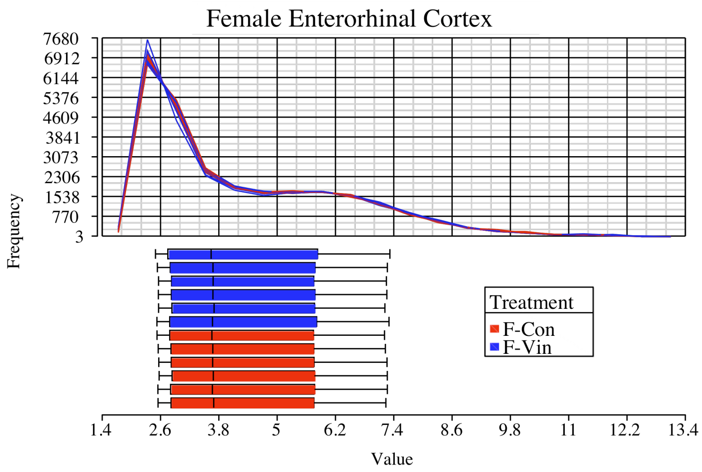


D

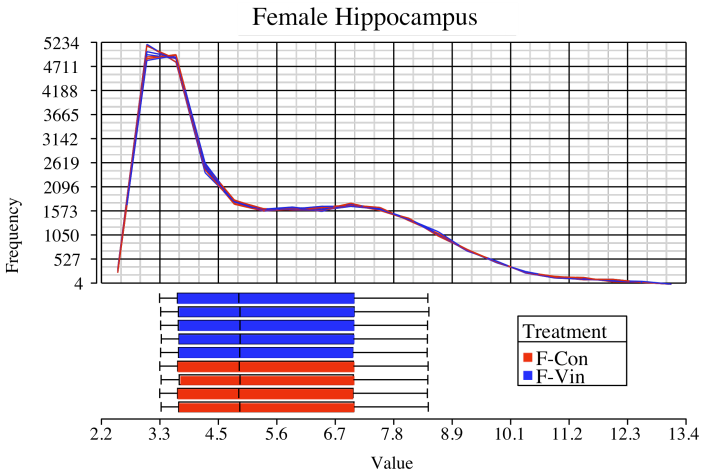


E

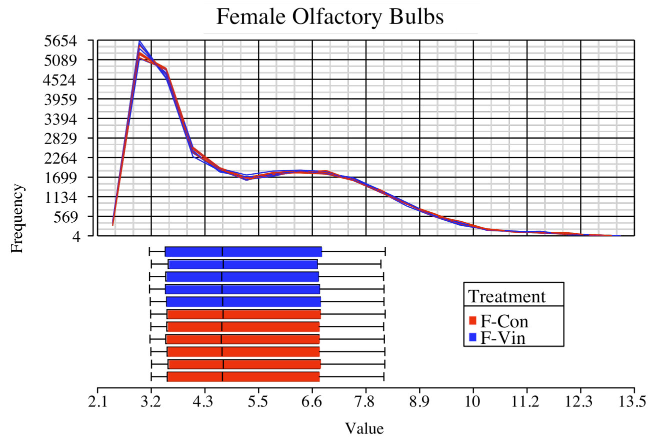


F

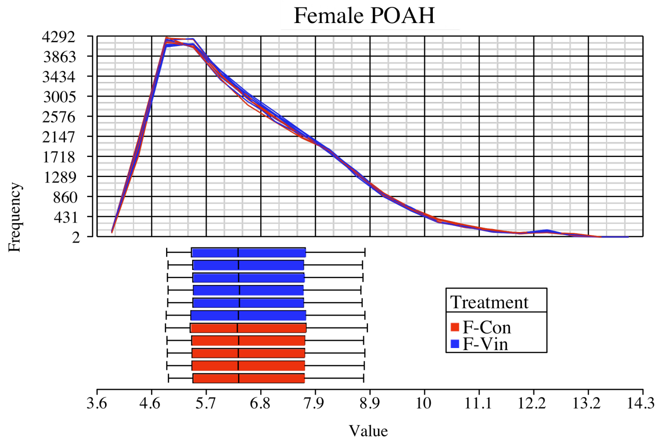

Supplement: Supplementary file 3 — Additional file 3: Figure S1A: Samples Histograms After Pre-processing (Male). Figure S1B. Samples Histograms After Pre-Processing (Female). Figure S1. Sample histograms and box plots for male (S1A) female (S1B) microarray signal values after pre-processing with RMA, GCcontent adjusted algorithm. Plots for F3 generation control (red) and F3 generation vinclozolin (blue) chips for female amygdala (A), cingulate cortex (B), enterorhinal cortex (C), hippocampus (D), olfactory bulbs (E), and preoptic areaanterior hypothalamus (F). (PDF 4 MB) [file 12864_2013_6162_MOESM3_ESM.pdf]
