# Supplementary material for: Gene bionetworks involved in the epigenetic transgenerational inheritance of altered mate preference: environmental epigenetics and evolutionary biology
Source: BMC Genomics. 2014 May 16;15(1):377. doi: 10.1186/1471-2164-15-377 (PMC4073506; doi:10.1186/1471-2164-15-377)
Supplement: Supplementary file 4 — Additional file 4: Table S2: Genes Differentially Expressed in F3 Generation Vinclozolin Versus Control Lineage Male and Female Rat Brain Regions. (PDF 618 KB) [file 12864_2013_6162_MOESM4_ESM.pdf]

Table S2. GenesDifferentially Expressed in F3 Generation Vinclozolin Versus Control Lineage Male and Female Rat Brain Regions

Table S2-A. Differentially Expressed Genes in Male Rat Amygdala (169 genes & ESTs)

Gene Symbol	GenBank, Ref.Sequence	Vin/Con Ratio	Vin-Con mean_dif	k.in **	Module**	ProbeSet ID	Gene Title
**Cell Cycle**							
Cdc25b	NM_133572	0.81	-11.8	2.5	turq	10840020	cell division cycle 25 homolog B (S. pombe)
Cp110	NM_001108501	1.23	15.7	7.4	turq	10710179	CP110 protein <centrosomal protein of 110 kDa>
**Cytoskeleton-ECM**							
Hydin	ENSRNOT00000059128	1.23	2.6	6.5	turq	10807965	hydrocephalus inducing
Ka11	NM_001008750	0.82	-2.2	7.4	turq	10747288	type I keratin KA11
Pcdh17	NM_001107279	1.20	47.4	11.2	turq	10781745	protocadherin 17
Pcdh19	NM_001169129	1.20	24.4	6.9	turq	10939226	protocadherin 19
Scg2	NM_022669	1.35	166	8.6	turq	10929263	secretogranin II (chromogranin C)
Tspan18	NM_001107750	0.80	-5.7	6.0	turq	10847562	tetraspanin 18
**Development**							
Ahi1	NM_001002277	1.22	99	3.0	turq	10702025	Abelson helper integration site 1
Bex4	NM_001037554	1.41	12.4	4.1	turq	10935038	brain expressed gene 4
Clmn	NM_001106755	1.22	23.2	8.0	turq	10892035	calmin
Neurod6	NM_001109237	1.31	27.9	5.9	turq	10862698	neurogenic differentiation 6
Ntn1	AY028417	1.22	4.9	2.3	turq	10743781	netrin 1
Postn	NM_001108550	0.80	-2.5	5.3	turq	10815369	periostin, osteoblast specific factor
**Rsrc1**	**NM_001014172**	**1.24**	**28.0**	**11.4**	**turq**	**10815795**	**arginine/serine-rich coiled-coil 1**
**Sfrp5**	**NM_001107591**	**0.81**	**-3.9**	**0.8**	**blue**	**10715448**	**secreted frizzled-related protein 5**
Slit2	NM_022632	1.25	14.8	6.1	turq	10777137	slit homolog 2 (Drosophila)
**Trpc4**	**NM_080396**	**1.32**	**23.9**	**13.3**	**turq**	**10815352**	**transient receptor potential cation channel, subfamily C, member 4**
Vgll3	ENSRNOT00000042316	1.42	8.3	2.6	turq	10749839	vestigial like 3 (Drosophila)
**DNA repair**							
RGD1561537	ENSRNOT00000025965	1.26	23.1	7.7	turq	10796965	similar to putative repair and recombination helicase RAD26L
**Electron Transport**							
Cyp26b1	NM_181087	1.24	2.6	8.2	turq	10863608	cytochrome P450, family 26, subfamily b, polypeptide 1
**Epigenetics**							
Apeg3	ENSRNOT00000037528	1.27	7.7	4.6	turq	10718666	antisense paternally expressed gene 3
Taf1b	ENSRNOT00000006187	1.21	8.7	2.3	turq	10883865	TATA box binding protein (Tbp)-associated factor, RNA polymerase I, B
**Golgi Apparatus**							
Ap1s2	NM_001127531	1.22	60.2	9.6	turq	10937769	adaptor-related protein complex 1, sigma 2 subunit
**Growth Factors**							
Dlk1	NM_053744	1.24	6.5	6.9	turq	10886806	delta-like 1 homolog (Drosophila)
Ccl7	NM_001007612	0.83	-2.5	5.0	turq	10736702	chemokine (C-C motif) ligand 7
Megf10	NM_001100657	0.83	-14.4	0.1	blue	10801833	multiple EGF-like domains 10
**Immune Response**							
Igdcc3	NM_001108160	0.82	-4.6	0.0	grey	10910895	immunoglobulin superfamily, DCC subclass, member 3
RT1-M3-1	NM_022921	0.83	-4.6	0.1	blue	10830841	RT1 class Ib, locus M3, gene 1
RT1-M6-1	NM_001008852	0.76	-4.9	9.1	turq	10827686	RT1 class I, locus M6, gene 1
Spag7	NM_001107016	1.21	15.1	6.5	turq	10744498	sperm associated antigen 7
**Metabolism & Transport**							
Alas2	NM_013197	0.82	-3.2	5.8	turq	10932912	aminolevulinate, delta-, synthase 2
Car12	NM_001080756	1.21	26.4	5.0	turq	10911145	carbonic anyhydrase 12
**Csgalnact1**	**NM_001107309**	**1.22**	**21.3**	**18.1**	**turq**	**10787757**	**chondroitin sulfate N-acetylgalactosaminyltransferase 1**
Doc2b	NM_031142	1.33	8.2	6.9	turq	10744970	double C2-like domains, beta
Hba-a2	NM_013096	0.79	-621	6.0	turq	10741756	hemoglobin alpha, adult chain 2
Hbb	NM_033234	0.75	-92.6	4.1	turq	10724311	hemoglobin, beta
Hcn2	NM_053684	0.81	-33.3	3.1	turq	10900793	hyperpolarization activated cyclic nucleotide-gated potassium channel 2
Kctd4	NM_001109650	1.31	55.4	6.0	turq	10781566	potassium channel tetramerisation domain containing 4
Lpl	NM_012598	1.35	13.2	7.1	turq	10791250	lipoprotein lipase
Ndor1	NM_001107818	0.82	-12.8	3.8	turq	10843380	NADPH dependent diflavin oxidoreductase 1
Pla2g2a	NM_031598	0.81	-3.2	7.0	turq	10873341	phospholipase A2, group IIA (platelets, synovial fluid)
Pter	NM_022224	1.20	4.5	2.9	turq	10796440	phosphotriesterase related
Pus3	NM_001108134	1.24	6.3	11.1	turq	10908990	pseudouridylate synthase 3
Slc5a5	NM_052983	0.82	-2.3	2.0	turq	10787401	solute carrier family 5 (sodium iodide symporter), member 5
**Slc9a4**	**NM_173098**	**1.31**	**13.2**	**11.4**	**turq**	**10922895**	**solute carrier family 9 (sodium**
**Proteolysis**							
Cpxm2	NM_001106306	0.79	-4.0	0.0	grey	10726255	carboxypeptidase X (M14 family), member 2
Pgc	NM_133284	0.70	-5.2	7.5	turq	10926299	progastricsin (pepsinogen C)
Tmprss5	NM_153311	0.82	-3.2	0.1	blue	10909828	transmembrane protease, serine 5

**Usp27x**	**ENSRNOT00000003800**	**1.29**	**16.8**	**12.1**	**turq**	**10932570**	**ubiquitin specific peptidase 27, X-linked**
Usp29	NM_001108465	1.29	42.6	7.4	turq	10718664	ubiquitin specific peptidase 29
**Receptors & Binding Proteins**							
Abcc1	NM_022281	1.23	20.4	0.7	blue	10740293	ATP-binding cassette, sub-family C (CFTR
Chrm5	NM_017362	1.27	5.5	1.9	turq	10848165	cholinergic receptor, muscarinic 5
Esr1	NM_012689	1.28	3.0	7.7	turq	10702695	estrogen receptor 1
Gabra5	NM_017295	1.27	96.5	9.8	turq	10722347	gamma-aminobutyric acid (GABA) A receptor, alpha 5
**Glra2**	**NM_012568**	**1.49**	**15.1**	**11.3**	**turq**	**10933393**	**glycine receptor, alpha 2**
Htr2c	NM_012765	1.45	81.7	6.3	turq	10937302	5-hydroxytryptamine (serotonin) receptor 2C
N4bp2	ENSRNOT00000031792	1.26	7.7	3.5	turq	10776778	NEDD4 binding protein 2
Nxph1	NM_012994	1.20	21.2	3.3	turq	10853719	neurexophilin 1
Olr1584	NM_001000081	0.83	-2.2	4.5	turq	10765827	olfactory receptor 1584
**Pgrmc1**	**NM_021766**	**1.32**	**116**	**15.4**	**turq**	**10936341**	**progesterone receptor membrane component 1**
S100a5	NM_001106438	0.80	-5.5	3.6	turq	10817061	S100 calcium binding protein A5
Stxbp6	ENSRNOT00000005618	1.23	53.1	7.9	turq	10889772	syntaxin binding protein 6 (amisyn)
Vom2r44	NM_001099498	1.22	6.4	1.9	turq	10815724	vomeronasal 2 receptor 44
**Signaling**							
Fgd5	NM_001108637	0.83	-4.5	10.9	turq	10857382	FYVE, RhoGEF and PH domain containing 5
Gpr101	NM_001108258	1.36	6.4	5.7	turq	10939901	G protein-coupled receptor 101
Gpr22	NM_001106722	1.20	22.5	6.8	turq	10889575	G protein-coupled receptor 22
Hsf2	NM_031694	1.21	50.0	9.5	turq	10830201	heat shock transcription factor 2
**MGC109340**	**NM_001024267**	**1.22**	**45.1**	**15.5**	**turq**	**10935031**	**similar to Microsomal signal peptidase 23 kDa subunit (SPase 22 kDa subunit) (SPC22**
Nmbr	NM_012799	1.31	5.5	5.5	turq	10701839	neuromedin B receptor
Pacsin3	NM_001009966	0.81	-7.7	7.0	turq	10837845	protein kinase C and casein kinase substrate in neurons 3
Pdp2	NM_145091	1.21	7.2	4.0	turq	10809001	pyruvate dehyrogenase phosphatase catalytic subunit 2
Pdyn	NM_019374	1.29	9.5	4.6	turq	10849857	prodynorphin
Rab9b	NM_001109018	1.21	42.1	9.5	turq	10939460	RAB9B, member RAS oncogene family
**RGD1561963**	**ENSRNOT00000064161**	**1.23**	**35.0**	**11.3**	**turq**	**10929321**	**similar to Dedicator of cytokinesis protein 10 (Protein zizimin 3)**
Rhoj	NM_001008320	0.83	-4.3	6.1	turq	10885299	ras homolog gene family, member J
RragB	NM_053972	1.22	87.4	4.3	turq	10937479	Ras-related GTP binding B
Sla	NM_178097	1.25	7.7	9.0	turq	10904161	src-like adaptor
Syde2	ENSRNOT00000036282	1.24	5.1	3.6	turq	10819662	synapse defective 1, Rho GTPase, homolog 2 (C. elegans)
**Transcription**							
Foxp2	XM_001056575	1.37	5.0	10.8	turq	10853783	forkhead box P2
Klf5	NM_053394	0.79	-5.4	0.6	blue	10781829	Kruppel-like factor 5
Klhl15	NM_001108021	1.21	10.0	5.0	turq	10934007	kelch-like 15 (Drosophila)
LOC499110	ENSRNOT00000041861	1.24	10.0	9.2	turq	10705431	similar to Zinc finger protein 354A (Transcription factor 17) (Renal transcription factor Kid-1)
Mxd1	NM_001100749	0.78	-12.2	9.3	turq	10863726	max dimerization protein 1
Nfkb2	NM_001008349	0.83	-2.4	4.1	turq	10715787	nuclear factor of kappa light polypeptide gene enhancer in B-cells 2, p49
Nol8	NM_001108408	1.24	10.6	4.1	turq	10797631	nucleolar protein 8
**Nrip1**	**NM_001100560**	**1.27**	**29.8**	**14.8**	**turq**	**10752754**	**nuclear receptor interacting protein 1**
RGD1311064	ENSRNOT00000040609	1.31	20.9	5.2	turq	10703495	similar to KRAB zinc finger protein KR18
RGD1564836	ENSRNOT00000038940	0.81	-19.7	4.7	turq	10937775	similar to HMGA1b
Rprm	NM_001044276	0.82	-8.8	0.2	blue	10845372	reprimo, TP53 dependent G2 arrest mediator candidate
Sirt4	NM_001107147	1.27	4.7	6.7	turq	10758972	sirtuin (silent mating type information regulation 2 homolog) 4 (S. cerevisiae)
**Tiparp**	**NM_001107679**	**1.33**	**24.0**	**13.6**	**turq**	**10815763**	**TCDD-inducible poly(ADP-ribose) polymerase**
Zc3h15	NM_001010963	1.21	11.9	3.6	turq	10764523	zinc finger CCCH-type containing 15
Zfp583	NM_001134609	1.20	5.7	7.1	turq	10703864	zinc finger protein 583
Zfp748	ENSRNOT00000022527	1.20	20.8	3.9	turq	10796904	zinc finger protein 748
**Zfp9**	**NM_001127635**	**1.23**	**5.0**	**15.1**	**turq**	**10864974**	**zinc finger protein 9**
**Translation & Protein Modification**							
Cwc22	ENSRNOT00000037684	1.24	14.2	1.2	turq	10846661	CWC22 spliceosome-associated protein homolog (S. cerevisiae)
Ncl	NM_012749	1.28	49.3	2.0	turq	10779091	nucleolin
Rbm24	ENSRNOT00000022768	1.22	8.8	0.1	blue	10797774	RNA binding motif protein 24
RGD1562381	ENSRNOT00000040565	1.22	2.2	2.0	turq	10890687	similar to ribosomal protein S17
RGD1562905	ENSRNOT00000068097	0.70	-5.5	5.8	turq	10812148	similar to 60S ribosomal protein L17 (L23)
RGD1565520	XR_086190	0.83	-134	10.4	turq	10843448	similar to 60S ribosomal protein L7a
Rnase1	NM_001029904	0.82	-2.3	0.0	grey	10783193	ribonuclease, RNase A family, 1 (pancreatic)
**Miscellaneous & Unknown**							
Omp	NM_012616	0.78	-6.7	8.3	turq	10723805	olfactory marker protein
RGD1561672	ENSRNOT00000056708	1.20	3.7	4.1	turq	10778861	similar to novel protein
Fam114a1	ENSRNOT00000002883	1.20	5.9	5.8	turq	10776888	family with sequence similarity 114, member A1
Fam118b	BC098017	1.21	8.1	1.4	turq	10916052	family with sequence similarity 118, member B
LOC292449	AY389467	1.26	6.7	3.9	turq	10701663	similar to hypothetical protein
LOC500392	NM_001024340	1.25	5.0	3.8	turq	10875102	similar to hypothetical protein FLJ25692
LOC500625	ENSRNOT00000006877	1.28	12.2	8.9	turq	10883443	hypothetical protein LOC500625
LOC681382	ENSRNOT00000058235	1.26	2.9	3.9	turq	10933953	hypothetical protein LOC681382
LOC690074	ENSRNOT00000056319	0.81	-2.9	2.2	turq	10930409	hypothetical protein LOC690074
Mctp1	ENSRNOT00000017828	1.30	35.7	5.9	turq	10812270	multiple C2 domains, transmembrane 1

RGD1307882	ENSRNOT00000046873	1.20	42.9	2.5	turq	10919330	similar to CG9346-PA
RGD1562726	NM_001109052	1.26	5.9	5.6	turq	10753129	similar to Putative protein C21orf62 homolog
RGD1565493	XM_002729803	1.22	7.8	3.5	turq	10903292	similar to DKFZP434I092 protein
Ptcd3	NM_001134718	1.25	29.4	11.2	turq	10863158	Pentatricopeptide repeat domain 3
Wdr60	ENSRNOT00000006144	1.22	10.0	4.0	turq	10892677	WD repeat domain 60
**ESTs**							
	**---**	**0.53**	**-34.3**	**15.8**	**turq**	**10851484**	
	**ENSRNOT00000059064**	**0.72**	**-6.5**	**15.0**	**turq**	**10781110**	
	**ENSRNOT00000004252**	**1.23**	**20.9**	**13.3**	**turq**	**10935021**	
	**---**	**1.29**	**29.3**	**12.8**	**turq**	**10788687**	
	ENSRNOT00000018668	1.22	14.8	10.3	turq	10715190	
	ENSRNOT00000044930	0.72	-55.8	9.9	turq	10798463	
	ENSRNOT00000061700	0.81	-2.8	9.6	turq	10853227	
	---	0.69	-31.6	8.6	turq	10776216	
	ENSRNOT00000057365	0.67	-63.3	8.6	turq	10924441	
	ENSRNOT00000058123	0.75	-7.9	8.2	turq	10902762	
	GENSCAN00000037874	1.25	7.7	8.1	turq	10807953	
	ENSRNOT00000053817	0.79	-3.5	8.0	turq	10796147	
RGD1559879	ENSRNOT00000057187	1.21	3.1	7.8	turq	10830484	similar to chromosome 6 open reading frame 199
	ENSRNOT00000005413	0.79	-6.2	7.7	turq	10895439	
	ENSRNOT00000056070	0.75	-12.5	7.6	turq	10905664	
	---	0.82	-6.6	7.4	turq	10901954	
	ENSRNOT00000063576	0.80	-102	7.3	turq	10891487	
	ENSRNOT00000053785	0.83	-5.4	7.0	turq	10811730	
	ENSRNOT00000054460	0.71	-35.3	6.9	turq	10756606	
	ENSRNOT00000041305	0.65	-20.3	6.9	turq	10744937	
	ENSRNOT00000033462	0.82	-3.0	6.3	turq	10793335	
	---	1.32	7.7	6.0	turq	10885293	
	ENSRNOT00000035017	1.26	9.4	5.7	turq	10820589	
	---	1.36	4.5	5.5	turq	10908604	
	ENSRNOT00000055650	0.81	-5.4	5.4	turq	10737460	
	ENSRNOT00000063593	0.82	-2.9	5.3	turq	10907802	
	ENSRNOT00000034404	0.82	-2.8	5.1	turq	10900071	
	ENSRNOT00000047961	0.74	-5.0	5.0	turq	10877368	
	ENSRNOT00000058481	0.76	-7.3	5.0	turq	10862873	
	ENSRNOT00000007245	0.78	-5.9	4.7	turq	10901836	
	ENSRNOT00000054653	0.78	-4.4	4.6	turq	10922743	
LOC100361083	XR_086263	1.21	6.2	4.5	turq	10879660	hypothetical LOC100361083
	---	0.81	-4.1	4.5	turq	10875254	
RGD1308147	BC088171	1.24	13.0	4.5	turq	10798724	similar to expressed sequence AW209491
	ENSRNOT00000059964	1.39	5.7	4.5	turq	10813643	
	rno-mir-9-1	0.80	-6.6	4.5	turq	10816485	
	ENSRNOT00000037006	1.22	15.3	4.3	turq	10896494	
	ENSRNOT00000054650	0.75	-6.1	4.3	turq	10929761	
	---	1.37	7.9	3.8	turq	10823419	
	ENSRNOT00000019601	1.32	37.2	3.8	turq	10799084	
	ENSRNOT00000054119	0.78	-3.1	3.8	turq	10796879	
	ENSRNOT00000051757	1.30	13.8	2.7	turq	10935150	
	ENSRNOT00000046954	0.83	-2.3	2.4	turq	10882569	
	---	0.78	-2.3	2.3	turq	10832326	
	---	0.81	-2.2	1.9	turq	10774341	
	NC_001665	1.34	724	1.3	turq	10930620	
	ENSRNOT00000060603	0.79	-7.8	0.7	turq	10908817	
	ENSRNOT00000068960	1.24	5.6	0.4	blue	10814096	
	ENSRNOT00000052539	0.75	-13.4	0.3	blue	10886988	
	GENSCAN00000044033	0.81	-2.4	0.0	grey	10904699	
	ENSRNOT00000053993	0.75	-8.6	0.0	grey	10756951	

*** - genes in top 10% connectivity of each module are marked by bold font**

**- Modules and k.in are given for seaparate networks; abbreviations used for modules: *turq* - turquoise, *ylw* -yellow, *brwn* - brown

Table S2-B. Differentially Expressed Genes in Male Rat Hippocampus (146 genes & ESTs)

Gene Symbol	GenBank, Ref.Sequence	Vin/Con Ratio	Vin-Con mean_dif	k.in **	Module**	ProbeSet ID	Gene Title
**Apoptosis**							
Hrk	NM_057130	1.26	12.3	3.5	turq	10762426	harakiri, BCL2 interacting protein (contains only BH3 domain)

**Cytoskeleton-ECM**							
Cgnl1	NM_001108164	0.75	-6.5	7.0	turq	10918600	cingulin-like 1
Col8a1	NM_001107100	0.81	-2.1	3.5	turq	10750685	collagen, type VIII, alpha 1
Dnah5	ENSRNOT00000017372	0.82	-3.2	4.3	turq	10813995	dynein, axonemal, heavy chain 5
Dnali1	NM_001031647	0.82	-2.9	6.2	turq	10879718	dynein, axonemal, light intermediate chain 1
Flna	NM_001134599	0.79	-7.2	8.3	turq	10936086	filamin A, alpha
**Hydin**	**ENSRNOT00000059128**	**0.70**	**-4.5**	**10.5**	**turq**	**10807965**	**hydrocephalus inducing**
Lama2	ENSRNOT00000014917	0.78	-5.7	6.1	turq	10702096	laminin, alpha 2
**Nid1**	**ENSRNOT00000003349**	**0.77**	**-6.5**	**11.9**	**turq**	**10799017**	**nidogen 1**
RGD1566343	NM_001047866	0.83	-6.4	0.0	grey	10763433	similar to contactin associated protein-like 5 isoform 1
Tmem204	NM_001009620	1.24	3.6	0.0	grey	10741330	transmembrane protein 204
**Development**							
Fras1	ENSRNOT00000002814	1.31	9.5	6.6	turq	10775647	Fraser syndrome 1 homolog (human)
Mustn1	NM_181368	0.83	-3.3	6.7	turq	10786532	musculoskeletal, embryonic nuclear protein 1
Net1	NM_001039023	0.82	-6.1	4.4	turq	10796074	neuroepithelial cell transforming 1
Pacrg	NM_001077677	0.78	-12.4	6.1	turq	10703104	Park2 co-regulated
Pmch	NM_012625	0.80	-3.7	5.7	turq	10894708	pro-melanin-concentrating hormone
Rshl3	NM_001107629	0.72	-4.2	3.5	turq	10830081	radial spokehead-like 3
Vamp8	NM_031827	0.75	-10.5	6.1	turq	10863218	vesicle-associated membrane protein 8
**Electron Transport**							
**Cybb**	**NM_023965**	**0.81**	**-4.6**	**1.0**	**blue**	**10936899**	**cytochrome b-245, beta polypeptide**
Gpx3	NM_022525	0.81	-5.5	4.3	turq	10733680	glutathione peroxidase 3
Ucp2	NM_019354	0.74	-24.8	8.4	turq	10709093	uncoupling protein 2 (mitochondrial, proton carrier)
**Epigenetics**							
Satb2	NM_001109306	0.83	-5.2	0.3	blue	10928191	SATB homeobox 2
Drd1a	NM_012546	1.41	14.5	4.8	turq	10794195	dopamine receptor D1A
Hist1h1b	NM_001109417	0.77	-19.0	5.0	turq	10795291	histone cluster 1, H1b
**Golgi Apparatus**							
St6galnac2	NM_001031652	0.82	-2.5	5.9	turq	10749330	ST6 (alpha-N-acetyl-neuraminyl-2,3-beta-galactosyl-1,3)-N-acetylgalactosaminide alpha-2,6-sial
**Growth Factors**							
Bmp7	ENSRNOT00000009656	0.71	-15.3	5.8	turq	10852106	bone morphogenetic protein 7
**Igf2**	**NM_031511**	**0.61**	**-16.3**	**11.3**	**turq**	**10726999**	**insulin-like growth factor 2**
Mdk	NM_030859	0.58	-37.2	9.6	turq	10847432	midkine
Pf4	NM_001007729	0.81	-3.0	3.9	turq	10775914	platelet factor 4
Tgfbr2	NM_031132	0.77	-7.8	7.0	turq	10920745	transforming growth factor, beta receptor II
**Immune Response**							
C1r	NM_001134555	0.82	-10.2	3.9	turq	10858655	complement component 1, r subcomponent
**Cd34**	**NM_001107202**	**0.73**	**-9.7**	**10.4**	**turq**	**10766869**	**CD34 molecule**
**Cd9**	**NM_053018**	**0.78**	**-66.5**	**10.0**	**turq**	**10865715**	**CD9 molecule**
F13a1	NM_021698	0.79	-3.2	9.3	turq	10794734	coagulation factor XIII, A1 polypeptide
Ifitm3	NM_001136124	0.80	-8.2	10.0	turq	10726682	interferon induced transmembrane protein 3
LOC287167	NM_001013853	0.75	-30.6	8.4	turq	10741765	globin, alpha
Xkr8	NM_001012099	1.25	4.7	5.4	turq	10872681	XK, Kell blood group complex subunit-related family, member 8
**Metabolism & Transport**							
Akr1c19	NM_001100576	0.79	-5.0	6.9	turq	10796050	aldo-keto reductase family 1, member C19
Cald1	NM_013146	0.75	-8.9	5.9	turq	10854446	caldesmon 1
Cp	NM_012532	0.81	-8.9	7.1	turq	10814430	ceruloplasmin
Elovl7	ENSRNOT00000014074	0.79	-19.7	3.9	turq	10812922	ELOVL family member 7, elongation of long chain fatty acids (yeast)
Enkur	NM_001106126	0.78	-12.4	5.1	turq	10799977	enkurin, TRPC channel interacting protein
Ephx1	NM_001034090	0.78	-31.0	3.2	turq	10770342	epoxide hydrolase 1, microsomal
Extl2	NM_001100704	0.83	-45.0	5.3	turq	10818590	exostoses (multiple)-like 2
Galm	NM_001007704	0.81	-15.7	6.1	turq	10882496	galactose mutarotase (aldose 1-epimerase)
Glb1l	NM_001127529	0.83	-9.2	6.1	turq	10929105	galactosidase, beta 1-like
Gstm2	NM_177426	0.72	-5.7	2.4	turq	10825915	glutathione S-transferase mu 2
Mreg	ENSRNOT00000021152	0.83	-4.3	2.6	turq	10928813	melanoregulin
Nt5dc2	NM_001009271	0.63	-14.1	3.4	turq	10786624	5'-nucleotidase domain containing 2
Ostf1	NM_148892	0.79	-20.4	2.9	turq	10729252	osteoclast stimulating factor 1
**Plcb4**	**NM_024353**	**0.83**	**-10.6**	**10.9**	**turq**	**10840183**	**phospholipase C, beta 4**
Plce1	NM_053758	0.81	-4.3	4.3	turq	10715153	phospholipase C, epsilon 1
Ppic	NM_001004215	0.82	-6.9	6.8	turq	10804480	peptidylprolyl isomerase C
Slc12a4	NM_019229	0.83	-11.1	6.5	turq	10810743	solute carrier family 12 (potassium
Slc2a12	NM_001107451	0.63	-14.4	6.1	turq	10717325	solute carrier family 2 (facilitated glucose transporter), member 12
**Slc43a3**	**NM_001107743**	**0.81**	**-6.9**	**13.4**	**turq**	**10837366**	**solute carrier family 43, member 3**
Slc5a7	NM_053521	0.82	-4.3	0.6	blue	10921208	solute carrier family 5 (choline transporter), member 7
Sod3	NM_012880	0.73	-26.3	5.7	turq	10777108	superoxide dismutase 3, extracellular
Sulf1	NM_134378	0.69	-21.2	0.3	blue	10874981	sulfatase 1
**Proteolysis**							
**Serping1**	**NM_199093**	**0.70**	**-26.7**	**11.2**	**turq**	**10846854**	**serine (or cysteine) peptidase inhibitor, clade G, member 1**
**Spint2**	**NM_001082549**	**0.78**	**-20.9**	**14.5**	**turq**	**10720479**	**serine peptidase inhibitor, Kunitz type, 2**

**Receptors & Binding Proteins**							
Igfbp2	NM_013122	0.48	-41.5	8.5	turq	10924223	insulin-like growth factor binding protein 2
Mrc2	NM_001024687	0.81	-4.2	6.2	turq	10738972	mannose receptor, C type 2
Olr1541	NM_001000728	0.83	-3.1	0.5	blue	10750624	olfactory receptor 1541
Olr566	NM_001000665	1.23	3.4	5.6	turq	10837528	olfactory receptor 566
Ranbp3l	ENSRNOT00000050942	0.70	-17.8	3.2	turq	10813563	RAN binding protein 3-like
S100a11	NM_001004095	0.70	-9.0	5.5	turq	10817183	S100 calcium binding protein A11 (calizzarin)
S100a5	NM_001106438	0.82	-5.0	4.1	turq	10817061	S100 calcium binding protein A5
Tlr3	NM_198791	0.82	-5.6	2.9	turq	10788238	toll-like receptor 3
Ttr	NM_012681	0.23	-77.2	8.1	turq	10800426	transthyretin
**Signaling**							
Alpk1	ENSRNOT00000030798	0.81	-6.3	8.9	turq	10826672	alpha-kinase 1
Anxa2	NM_019905	0.66	-21.5	6.8	turq	10911287	annexin A2
Dab2	NM_024159	0.71	-26.3	4.9	turq	10813361	disabled homolog 2 (Drosophila)
Enpp2	NM_057104	0.57	-200	4.9	turq	10903736	ectonucleotide pyrophosphatase
Fkbp9	NM_001007646	0.81	-31.8	6.2	turq	10855785	FK506 binding protein 9
Itga1	NM_030994	0.83	-3.7	3.2	turq	10821450	integrin, alpha 1
Itgb6	NM_001004263	0.78	-3.3	4.6	turq	10845587	integrin, beta 6
Mapk4	ENSRNOT00000047271	0.82	-21.0	7.3	turq	10805202	mitogen-activated protein kinase 4
Nmbr	NM_012799	1.26	4.6	3.1	turq	10701839	neuromedin B receptor
Nmur2	NM_022275	0.79	-2.6	3.3	turq	10742835	neuromedin U receptor 2
Pip5k1b	NM_001012743	0.82	-15.3	6.1	turq	10729444	phosphatidylinositol-4-phosphate 5-kinase, type I, beta
Rhoj	NM_001008320	0.75	-6.7	9.9	turq	10885299	ras homolog gene family, member J
Styxl1	NM_001037788	0.77	-6.0	3.2	turq	10757606	serine/threonine/tyrosine interacting-like 1
Tns1	ENSRNOT00000035277	0.80	-5.5	4.6	turq	10928855	tensin 1
**Transcription**							
Armc2	ENSRNOT00000031047	0.83	-5.9	0.5	blue	10833806	armadillo repeat containing 2
Asb15	BC097405	0.78	-2.8	0.4	blue	10853995	ankyrin repeat and SOCS box-containing protein 15
Btg2	NM_017259	1.25	10.5	5.1	turq	10767767	BTG family, member 2
Dhx37	NM_001105926	0.82	-6.4	8.2	turq	10758106	DEAH (Asp-Glu-Ala-His) box polypeptide 37
Dnajc21	NM_138856	0.78	-11.3	4.6	turq	10821900	DnaJ (Hsp40) homolog, subfamily C, member 21
Hey2	NM_130417	0.81	-8.4	4.6	turq	10702361	hairy/enhancer-of-split related with YRPW motif 2
**Nupr1**	**NM_053611**	**0.76**	**-13.0**	**10.5**	**turq**	**10725778**	**nuclear protein, transcriptional regulator, 1**
Wwtr1	NM_001024869	0.76	-21.0	0.5	blue	10823284	WW domain containing transcription regulator 1
Zc3hav1	NM_173045	0.82	-4.4	0.8	blue	10861976	zinc finger CCCH type, antiviral 1
Zic2	NM_001108392	0.78	-10.8	9.4	turq	10782156	Zic family member 2 (odd-paired homolog, Drosophila)
Znf697	ENSRNOT00000026253	1.20	3.3	3.2	turq	10817769	zinc finger protein 697
**Translation & Protein Modification**							
**Rbms2**	**NM_001025403**	**0.83**	**-21.6**	**10.4**	**turq**	**10899726**	**RNA binding motif, single stranded interacting protein 2**
RGD1561841	XM_001055235	1.29	6.3	9.8	turq	10823044	similar to ribosomal protein L31
RGD1562381	ENSRNOT00000040565	0.82	-2.2	1.3	turq	10890687	similar to ribosomal protein S17
Slfn5	ENSRNOT00000035730	0.67	-10.8	3.1	turq	10736784	schlafen family member 5
**Miscellaneous & Unknown**							
Omp	NM_012616	0.81	-5.6	0.0	grey	10723805	olfactory marker protein
**LOC363267**	**NM_001025025**	**0.82**	**-2.0**	**10.1**	**turq**	**10924785**	**hypothetical protein LOC363267**
LOC500046	ENSRNOT00000065975	0.82	-5.5	2.1	turq	10853931	similar to hypothetical protein FLJ21986
LOC654482	NM_001039174	0.63	-17.9	5.3	turq	10914823	hypothetical protein LOC654482
RGD1309108	ENSRNOT00000010743	1.23	3.1	4.2	turq	10916458	similar to hypothetical protein FLJ23554
RGD1563307	ENSRNOT00000030832	0.78	-4.9	3.3	turq	10720939	similar to Set beta isoform
RGD1565493	XM_002729803	0.79	-3.9	6.2	turq	10903280	similar to DKFZP434I092 protein
Ttc18	NM_001108370	0.83	-6.4	7.5	turq	10779130	tetratricopeptide repeat domain 18
RGD1306991	NM_001014183	0.72	-127	3.7	turq	10840226	similar to Protein C20orf103 precursor
**ESTs**							
	**NC_001665**	**1.43**	**934**	**11.8**	**turq**	**10930580**	
	ENSRNOT00000053783	1.22	9.7	8.7	turq	10701689	
	ENSRNOT00000055790	0.78	-6.5	8.4	turq	10866205	
	---	0.76	-7.0	8.2	turq	10785259	
	ENSRNOT00000032313	0.82	-2.2	7.8	turq	10807891	
	ENSRNOT00000055574	0.83	-4.9	7.5	turq	10840481	
MGC114440	BC098937	0.80	-2.7	7.3	turq	10872852	similar to RIKEN cDNA 4930555I21
RGD1309926	ENSRNOT00000005478	0.82	-11.2	7.1	turq	10732657	similar to RIKEN cDNA G431001E03 gene
	ENSRNOT00000053313	1.20	3.0	6.4	turq	10835526	
	ENSRNOT00000043852	0.74	-4.4	5.9	turq	10797671	
	ENSRNOT00000056240	1.30	20.7	5.8	turq	10926089	
	GENSCAN00000004438	0.82	-3.4	5.5	turq	10807879	
	ENSRNOT00000049035	1.21	5.5	5.4	turq	10789035	
	---	1.36	14.1	5.4	turq	10727640	
RGD1559879	ENSRNOT00000057187	0.75	-4.7	5.1	turq	10830484	similar to chromosome 6 open reading frame 199
	GENSCAN00000037874	0.78	-5.7	5.1	turq	10807925	

RGD1562658	NM_001109075	0.76	-7.9	5.1	turq	10765503	similar to RIKEN cDNA 1700009P17
	ENSRNOT00000037995	0.82	-12.4	5.0	turq	10758724	
	---	1.32	6.7	4.7	turq	10726674	
	---	1.32	15.5	4.6	turq	10752799	
	---	0.82	-3.2	3.6	turq	10859117	
	ENSRNOT00000003115	1.25	12.8	3.6	turq	10731326	
	---	0.79	-3.0	3.3	turq	10830759	
	ENSRNOT00000061078	1.21	9.3	3.2	turq	10756530	
	ENSRNOT00000052558	0.76	-8.6	2.9	turq	10778080	
	ENSRNOT00000046689	1.78	10.2	2.6	turq	10888777	
	ENSRNOT00000052469	0.77	-5.0	2.5	turq	10923862	
	ENSRNOT00000054460	0.77	-26.6	2.4	turq	10756606	
	ENSRNOT00000037006	0.83	-14.8	2.3	turq	10896494	
	ENSRNOT00000032733	1.24	3.7	2.0	turq	10756452	
	---	1.22	4.5	1.9	turq	10900073	
	ENSRNOT00000058328	0.81	-5.6	1.3	turq	10885611	
	ENSRNOT00000058469	1.23	5.1	0.8	turq	10902413	
	ENSRNOT00000015673	0.82	-2.6	0.7	blue	10736469	
RGD1306739	NM_001134576	0.80	-9.4	0.7	blue	10829771	similar to RIKEN cDNA 1700040L02
RGD1305587	BC158618	1.21	34.6	0.6	blue	10833144	similar to RIKEN cDNA 2010107G23
	rno-let-7c-1	1.23	2.9	0.0	grey	10749977	
	ENSRNOT00000057318	1.25	6.5	0.0	grey	10934792	

*** - genes belonning to top 10% of each module withing each brain region are marked by bold font**

**- Modules and k.in are given for seaparate networks; abbreviations used for modules: *turq* - turquoise, *ylw* -yellow, *brwn* - brown

Table S2-C. Differentially Expressed Genes in Male Rat Cingulate Cortex (763 genes and ESTs)

Gene Symbol	GenBank, Ref.Sequence	Vin/Con Ratio	Vin-Con mean_dif	k.in **	Module**	ProbeSet ID	Gene Title
**Apoptosis**							
Api5	NM_001127379	0.79	-62.1	26.1	turq	10847693	apoptosis inhibitor 5
**Casp3**	**NM_012922**	**0.77**	**-23.1**	**55.7**	**turq**	**10791652**	**caspase 3**
Cflar	NM_001033864	0.83	-11.0	35.8	turq	10923580	CASP8 and FADD-like apoptosis regulator
Tmbim1	NM_001007713	0.74	-34.3	25.2	turq	10928902	transmembrane BAX inhibitor motif containing 1
Tnfrsf1a	NM_013091	0.83	-5.0	6.4	turq	10858967	tumor necrosis factor receptor superfamily, member 1a
**Cell Cycle**							
Anapc4	NM_001107220	0.82	-46.5	16.4	turq	10777052	anaphase promoting complex subunit 4
Ccni	NM_001105998	0.81	-200	33.1	turq	10771584	cyclin I
Cdc26	NM_001013240	0.82	-27.5	40.4	turq	10877411	cell division cycle 26
Cdc27	NM_001024793	0.82	-23.2	17.0	turq	10748154	cell division cycle 27 homolog (S. cerevisiae)
Lin54	NM_001100564	0.82	-12.5	17.9	turq	10771412	lin-54 homolog (C. elegans)
Rbbp7	NM_031816	0.80	-91.6	41.9	turq	10937805	retinoblastoma binding protein 7
Rbbp9	NM_019219	0.80	-14.8	29.3	turq	10850427	retinoblastoma binding protein 9
**Cytoskeleton-ECM**							
Add3	NM_001164103	0.83	-130	23.9	turq	10730869	adducin 3 (gamma)
Cldn10	NM_001106058	0.81	-45.0	30.7	turq	10782020	claudin 10
Col6a1	ENSRNOT00000001679	1.24	5.6	15.7	turq	10829437	collagen, type VI, alpha 1
Csrp2	NM_177425	1.33	4.7	5.0	turq	10833152	cysteine and glycine-rich protein 2
Ermn	NM_001008311	0.73	-112	6.8	turq	10845401	ermin, ERM-like protein <ezrin/radixin/moesin >
Evi2a	NM_001044287	0.68	-41.9	24.5	turq	10745342	ecotropic viral integration site 2A
Fxc1	NM_053371	0.77	-3.6	13.5	turq	10709582	fractured callus expressed transcript 1
Gpm6b	NM_138846	0.83	-416	25.5	turq	10937666	glycoprotein m6b
Jup	NM_031047	0.76	-29.1	18.9	turq	10747330	junction plakoglobin
**Ka11**	**NM_001008750**	**1.23**	**2.3**	**54.8**	**turq**	**10747288**	**type I keratin KA11**
Kif5b	NM_057202	0.69	-199	36.0	turq	10795548	kinesin family member 5B
**Lamp2**	**NM_017068**	**0.76**	**-160**	**68.2**	**turq**	**10931919**	**lysosomal-associated membrane protein 2**
Lman1	NM_053886	0.83	-24.9	36.1	turq	10802347	lectin, mannose-binding, 1
Lsm14a	NM_001127552	0.83	-29.7	31.0	turq	10721028	LSM14A, SCD6 homolog A (S. cerevisiae)
Mapre1	NM_138509	0.77	-43.3	37.7	turq	10841066	microtubule-associated protein, RP
Mfap3l	NM_001012049	0.72	-149	25.4	turq	10791474	microfibrillar-associated protein 3-like
Mid2	ENSRNOT00000020145	0.81	-12.0	23.3	turq	10935215	midline 2
Mtmr10	NM_001100846	0.77	-25.4	38.8	turq	10707707	myotubularin related protein 10
Mynn	NM_001012178	0.83	-10.4	11.3	turq	10822697	myoneurin
Myo10	NM_001107657	0.83	-15.6	20.6	turq	10813894	myosin X
Myo6	ENSRNOT00000016428	0.72	-70.8	11.3	turq	10911882	myosin VI
Ninj1	NM_012867	0.79	-5.3	23.9	turq	10797705	ninjurin 1
Nudcd2	NM_001009621	0.82	-17.2	23.8	turq	10732811	NudC domain containing 2
Pcdhb18	XM_001055991	0.81	-27.5	18.3	turq	10801201	protocadherin beta 18

Pdlim5	NM_053326	0.83	-25.3	12.6	turq	10827079	PDZ and LIM domain 5
Pkp4	NM_001106482	0.76	-112	29.8	turq	10836321	plakophilin 4
Plp1	NM_030990	0.77	-1132	3.4	turq	10935064	proteolipid protein 1
RGD1305645	ENSRNOT00000036990	0.72	-6.2	12.6	turq	10922964	similar to RIKEN cDNA 1500015O10
RGD1561357	ENSRNOT00000010623	0.74	-248	19.6	turq	10866576	similar to LIM domain only 3
RGD1563615	ENSRNOT00000046525	0.73	-8.3	3.7	turq	10793429	similar to Contactin associated protein-like 3 precursor (Cell recognition molecule Caspr3)
Sgcb	ENSRNOT00000002921	0.79	-67.8	29.0	turq	10772318	sarcoglycan, beta (dystrophin-associated glycoprotein)
Sostdc1	NM_153737	1.54	7.6	39.4	turq	10884274	sclerostin domain containing 1
Tspan12	NM_001015026	0.68	-107	30.3	turq	10861213	tetraspanin 12
Tspan2	NM_022589	0.73	-85.6	3.1	turq	10817898	tetraspanin 2
Tspan3	NM_001005547	0.82	-118	20.6	turq	10917692	tetraspanin 3
**Tspan6**	**NM_001100672**	**0.74**	**-49.1**	**56.1**	**turq**	**10939233**	**tetraspanin 6**
Ttll2	ENSRNOT00000044831	1.25	15.4	15.3	turq	10718132	tubulin tyrosine ligase-like family, member 2
**Development**							
Amot	ENSRNOT00000049482	0.78	-19.9	17.8	turq	10932704	angiomotin
**Auts2l**	**NM_001107136**	**1.31**	**41.4**	**52.9**	**turq**	**10761297**	**autism susceptibility candidate 2-like**
Bbs10	NM_001109286	0.79	-9.3	31.0	turq	10895394	Bardet-Biedl syndrome 10
Bex4	NM_001037554	0.51	-23.7	33.2	turq	10935038	brain expressed gene 4
Chrdl1	NM_199502	0.70	-21.6	28.0	turq	10932773	kohjirin
Cnpy4	NM_001108852	0.83	-7.1	5.9	turq	10757239	canopy 4 homolog (zebrafish)
Cpne3	NM_001107917	0.82	-17.1	6.6	turq	10875680	copine III
Dlg1	NM_012788	0.83	-74.5	27.9	turq	10754902	discs, large homolog 1 (Drosophila)
**Dpy19l4**	**ENSRNOT00000037869**	**0.80**	**-17.2**	**49.0**	**turq**	**10875464**	**dpy-19-like 4 (C. elegans)**
Dynlt3	NM_001013228	0.80	-55.3	38.6	turq	10932366	dynein light chain Tctex-type 3
Epm2a	AF347030	0.77	-14.7	19.6	turq	10701792	epilepsy, progressive myoclonus type 2A
Kl	NM_031336	1.34	3.2	3.6	turq	10759762	Klotho
Lix1	NM_001106214	0.75	-189	28.6	turq	10703414	Lix1 homolog (chicken)
LOC312831	NM_001014060	0.81	-7.8	42.1	turq	10866738	similar to SRY (sex determining region Y)-box 5 isoform a
LOC683577	ENSRNOT00000061721	0.81	-16.4	6.9	turq	10931811	similar to odd Oz
LOC687620	ENSRNOT00000012282	0.79	-11.1	7.2	turq	10884701	similar to mirror-image polydactyly 1
Lrrc48	NM_001013857	0.74	-35.6	26.6	turq	10734045	leucine rich repeat containing 48
Mal	NM_012798	0.61	-111	8.7	turq	10849700	mal, T-cell differentiation protein
Mpzl1	NM_001007728	0.77	-36.8	31.2	turq	10769538	myelin protein zero-like 1
Nipa1	NM_001107519	0.76	-50.4	12.8	turq	10722326	non imprinted in Prader-Willi
Ntn1	AY028417	0.76	-6.9	13.3	turq	10743781	netrin 1
Odz3	NM_001169133	0.72	-32.1	11.3	turq	10788086	odz, odd Oz/ten-m homolog 3 (Drosophila)
Olfml1	NM_001013192	0.63	-71.2	46.3	turq	10709687	olfactomedin-like 1
Opalin	NM_001017386	0.68	-32.1	8.2	turq	10730148	oligodendrocytic myelin paranodal and inner loop protein
Osgin2	ENSRNOT00000012394	0.81	-12.9	11.1	turq	10875642	oxidative stress induced growth inhibitor family member 2
Pno1	NM_199083	0.76	-39.3	36.9	turq	10778568	partner of NOB1 homolog (S. cerevisiae)
Porf1	ENSRNOT00000005162	0.65	-43.6	22.1	turq	10891400	preoptic regulatory factor 1
Prrx1	NM_153821	0.83	-16.6	17.2	turq	10769422	paired related homeobox 1
Qser1	NM_001139493	0.77	-31.0	22.4	turq	10847955	glutamine and serine rich 1
Rex2	ENSRNOT00000040918	0.73	-19.2	39.1	turq	10873880	reduced expression 2
**Sar1b**	**NM_001009622**	**0.77**	**-131**	**50.0**	**turq**	**10733389**	**SAR1 homolog B (S. cerevisiae)**
Sema3a	NM_017310	0.81	-18.1	19.1	turq	10860481	sema domain, immunoglobulin domain (Ig), short basic domain, secreted, (semaphorin) 3A
Sntb1	NM_001130542	0.71	-20.2	27.8	turq	10903816	syntrophin, beta 1
Sspn	NM_001109255	0.78	-11.2	12.4	turq	10859641	sarcospan
Stox2	NM_001134863	0.73	-166	21.1	turq	10788175	storkhead box 2
Suhw3	ENSRNOT00000044435	0.81	-13.8	35.9	turq	10939617	suppressor of hairy wing homolog 3 (Drosophila)
**Tapt1**	**ENSRNOT00000004274**	**0.82**	**-32.8**	**57.4**	**turq**	**10773098**	**transmembrane anterior posterior transformation 1**
Tcp1	NM_012670	0.82	-88.1	36.2	turq	10863886	t-complex 1
Tex10	NM_001106653	0.79	-18.6	7.1	turq	10876688	testis expressed gene 10
Trps1	NM_001134837	0.68	-68.0	7.1	turq	10903676	trichorhinophalangeal syndrome I homolog (human)
Tsga14	NM_001025770	0.79	-8.1	14.0	turq	10861591	testis specific, 14
Unc13c	NM_173146	0.63	-25.2	18.2	turq	10918674	unc-13 homolog C (C. elegans) [=Munc13-3]
Zfhx2	NM_001098803	1.25	7.8	7.5	turq	10783753	zinc finger homeobox 2
**DNA Repair**							
RGD1561537	ENSRNOT00000025965	0.81	-18.6	9.4	turq	10796970	similar to putative repair and recombination helicase RAD26L
**Electron Transport**							
Etfdh	NM_198742	0.83	-19.1	21.1	turq	10823803	electron-transferring-flavoprotein dehydrogenase
**Magt1**	**NM_053946**	**0.70**	**-59.0**	**51.5**	**turq**	**10938935**	**magnesium transporter 1**
Pdcl3	NM_001025709	0.79	-17.9	44.4	turq	10922735	phosducin-like 3
Prdx1	NM_057114	0.83	-87.8	30.5	turq	10871286	peroxiredoxin 1
Tmem126a	NM_001011557	0.80	-8.4	11.8	turq	10723639	transmembrane protein 126A
Uqcrc2	NM_001006970	0.83	-143	32.4	turq	10710427	ubiquinol cytochrome c reductase core protein 2
**Epigenetics**							
Alkbh8	ENSRNOT00000003432	0.80	-21.8	27.7	turq	10892898	alkB, alkylation repair homolog 8 (E. coli)
Cav1	NM_031556	0.61	-39.6	37.5	turq	10853816	caveolin 1, caveolae protein

Cav2	NM_131914	0.77	-6.4	24.9	turq	10853810	caveolin 2
Chd7	NM_001107906	0.83	-4.1	9.8	turq	10867539	chromodomain helicase DNA binding protein 7
Coq5	NM_001039022	0.83	-18.9	16.4	turq	10762709	coenzyme Q5 homolog, methyltransferase (S. cerevisiae)
Epc2	NM_001108581	0.80	-25.9	26.3	turq	10719954	enhancer of polycomb homolog 2 (Drosophila)
Hdac1	NM_001025409	0.79	-22.4	30.1	turq	10880012	histone deacetylase 1
Hnmt	NM_031044	0.83	-9.9				histamine N-methyltransferase
Ipo7	NM_001107545	0.78	-66.6	33.8	turq	10709799	importin 7
Mettl11b	ENSRNOT00000039581	0.74	-5.9	45.9	turq	10769436	methyltransferase like 11B
Mier1	NM_001131012	0.83	-13.8	8.6	turq	10870401	mesoderm induction early response 1 homolog (Xenopus laevis)
Pcaf	NM_001024252	0.80	-28.3	38.5	turq	10926113	p300/CBP-associated factor <K(lysine) acetyltransferase 2B>
Rcbtb2	NM_199084	0.82	-23.6	17.1	turq	10781426	regulator of chromosome condensation (RCC1) and BTB (POZ) domain containing protein 2
**Golgi Apparatus**							
Hs6st2	ENSRNOT00000040165	0.76	-24.5	29.1	turq	10939725	heparan sulfate 6-O-sulfotransferase 2
Manea	NM_080785	0.79	-44.7	38.0	turq	10875823	mannosidase, endo-alpha
**Scoc**	**NM_001013235**	**0.79**	**-46.6**	**62.1**	**turq**	**10806864**	**short coiled-coil protein**
**Ap3m1**	**NM_133593**	**0.65**	**-67.0**	**59.1**	**turq**	**10779018**	**adaptor-related protein complex 3, mu 1 subunit**
**Bet1**	**NM_019251**	**0.77**	**-80.2**	**77.4**	**turq**	**10860858**	**blocked early in transport 1 homolog (S. cerevisiae)**
Galnt1	NM_024373	0.72	-75.8	31.7	turq	10800497	UDP-N-acetyl-alpha-D-galactosamine:polypeptide N-acetylgalactosaminyltransferase 1 (GalNAc
Golga5	NM_001033065	0.82	-11.6	18.2	turq	10886465	golgi autoantigen, golgin subfamily a, 5
**Selt**	**NM_001014253**	**0.78**	**-346**	**64.6**	**turq**	**10815552**	**selenoprotein T**
**Growth Factors**							
Ccr5	NM_053960	0.71	-21.7	14.9	turq	10914618	chemokine (C-C motif) receptor 5
Cetn3	ENSRNOT00000021735	0.79	-35.9	42.7	turq	10812346	centrin, EF-hand protein, 3 (CDC31 homolog, yeast)
Ctgf	NM_022266	0.83	-7.0	4.1	turq	10717233	connective tissue growth factor
Fgf2	NM_019305	0.69	-30.2	47.2	turq	10815026	fibroblast growth factor 2
Gab1	NM_001108444	0.76	-30.4	26.6	turq	10806927	GRB2-associated binding protein 1
Grb14	NM_031623	0.75	-26.6	40.9	turq	10845751	growth factor receptor bound protein 14
Negr1	NM_021682	0.82	-153	10.8	turq	10819880	neuronal growth regulator 1
Pdgfc	NM_031317	0.73	-32.2	9.2	turq	10816026	platelet derived growth factor C
RGD1307225	NM_001107663	0.80	-6.2	17.9	turq	10814545	similar to MEGF6 <multiple EGF-like-domains 6>
Sept2	NM_057148	0.77	-114	41.5	turq	10925600	septin 2
Tgfb2	NM_031131	0.71	-43.0	32.3	turq	10770577	transforming growth factor, beta 2
**Tgfbr1**	**NM_012775**	**0.75**	**-35.5**	**50.2**	**turq**	**10868923**	**transforming growth factor, beta receptor 1**
**Hormone**							
Tac1	NM_012666	0.70	-12.0	34.5	turq	10853683	tachykinin 1
**Immune Response**							
A1i3	NM_001037975	1.25	3.5	13.8	turq	10858444	alpha-1-inhibitor III
B2m	NM_012512	0.78	-350	18.9	turq	10839232	beta-2 microglobulin
Cd38	NM_013127	0.78	-11.7	18.5	turq	10777232	CD38 molecule
Cd79b	NM_133533	1.21	4.0	17.3	turq	10748273	Cd79b molecule, immunoglobulin-associated beta
Cfh	NM_130409	0.74	-47.0	10.8	turq	10768269	complement factor H
Ctage5	NM_001106734	0.83	-13.9	7.5	turq	10884772	CTAGE family, member 5 <Cutaneous T-cell lymphoma-associated antigen 5>
F5	NM_001047878	1.24	2.3	32.6	turq	10765212	coagulation factor V (proaccelerin, labile factor)
Fcgr2b	NM_175756	0.78	-6.3	26.4	turq	10769771	Fc fragment of IgG, low affinity IIb, receptor (CD32)
Lims1	NM_001145456	0.83	-35.0	4.6	turq	10833523	LIM and senescent cell antigen-like domains 1
LOC688858	XM_001068599	1.28	8.8	12.8	turq	10759581	similar to CD209a antigen
Mcfd2	NM_139253	0.81	-25.8	29.7	turq	10888301	multiple coagulation factor deficiency 2
**Mx2**	**NM_134350**	**0.78**	**-19.7**	**48.8**	**turq**	**10750524**	**myxovirus (influenza virus) resistance 2**
RT1-CE1	NM_001008832	1.22	10.3	15.2	turq	10831105	RT1 class I, locus1
RT1-M6-1	NM_001008852	1.42	6.3	29.6	turq	10827686	RT1 class I, locus M6, gene 1
Vsig2	NM_001106812	1.32	6.0	25.8	turq	10909080	V-set and immunoglobulin domain containing 2
**Metabolism & Transport**							
Aadat	NM_017193	0.63	-36.1	47.9	turq	10791478	aminoadipate aminotransferase
Aass	NM_001100963	0.77	-14.6	14.3	turq	10861242	aminoadipate-semialdehyde synthase
Acadl	NM_012819	0.81	-28.5	5.3	turq	10928602	acyl-Coenzyme A dehydrogenase, long-chain
Acadm	NM_016986	0.81	-19.2	43.3	turq	10827454	acyl-Coenzyme A dehydrogenase, C-4 to C-12 straight chain
Acat1	NM_017075	0.81	-56.3	43.1	turq	10917448	acetyl-coenzyme A acetyltransferase 1
Adi1	NM_199097	0.68	-22.2	31.5	turq	10883982	acireductone dioxygenase 1
Adpgk	NM_001100723	0.75	-29.7	9.7	turq	10910482	ADP-dependent glucokinase
Aldh6a1	NM_031057	0.72	-82.0	46.5	turq	10891120	aldehyde dehydrogenase 6 family, member A1
Alg11	NM_001108401	0.83	-93.1	20.1	turq	10792504	asparagine-linked glycosylation 11, alpha-1,2-mannosyltransferase homolog (yeast)
Alg5	NM_001025407	0.82	-9.7	17.8	turq	10815425	asparagine-linked glycosylation 5, dolichyl-phosphate beta-glucosyltransferase homolog
Amac1	NM_001127658	1.34	10.9	39.4	turq	10744212	acyl-malonyl condensing enzyme 1
Ampd3	NM_031544	0.76	-45.5	16.3	turq	10709880	adenosine monophosphate deaminase 3
Apold1	NM_001003403	0.80	-2.5	14.6	turq	10859262	apolipoprotein L domain containing 1
Aqp4	NM_012825	0.76	-230	28.9	turq	10803305	aquaporin 4
Asnsd1	NM_001106763	0.83	-33.4	26.8	turq	10923155	asparagine synthetase domain containing 1
Atp11a	NM_001107324	0.78	-37.0	3.4	turq	10792859	ATPase, class VI, type 11A
Atp6v1a	ENSRNOT00000002727	0.82	-13.7	40.3	turq	10823611	ATPase, H+ transporting, lysosomal V1 subunit A

Azin1	NM_022585	0.80	-99.3	32.6	turq	10903466	antizyme inhibitor 1
Car2	NM_019291	0.81	-117	3.1	turq	10822234	carbonic anhydrase II
Car5b	NM_001005551	0.74	-17.9	41.7	turq	10933449	carbonic anhydrase 5b, mitochondrial
Cat	NM_012520	0.81	-31.5	36.7	turq	10838155	catalase
Chst2	ENSRNOT00000056689	0.83	-102	22.3	turq	10919328	carbohydrate sulfotransferase 2
Cmbl	NM_001008770	0.71	-27.5	11.2	turq	10814105	carboxymethylenebutenolidase homolog (Pseudomonas)
Cml5	NM_080884	0.75	-110	10.9	turq	10863679	camello-like 5
Coq10b	NM_001009671	0.77	-21.6	35.5	turq	10923338	coenzyme Q10 homolog B (S. cerevisiae)
Cplx3	NM_001109295	0.70	-6.1	41.2	turq	10917776	complexin 3
Dars	NM_053799	0.76	-35.5	23.4	turq	10767356	aspartyl-tRNA synthetase
Dbt	NM_053312	0.77	-28.1	20.3	turq	10818594	dihydrolipoamide branched chain transacylase E2
Dio2	NM_031720	0.67	-10.6	29.3	turq	10891402	deiodinase, iodothyronine, type II
Dpyd	NM_031027	0.83	-7.6	5.9	turq	10818660	dihydropyrimidine dehydrogenase
Echdc1	NM_001007734	0.70	-37.5	43.5	turq	10717413	enoyl Coenzyme A hydratase domain containing 1
Erlin1	NM_001106353	0.81	-18.6	17.1	turq	10715519	ER lipid raft associated 1
**Exoc5**	**NM_022204**	**0.80**	**-36.5**	**53.8**	**turq**	**10782990**	**exocyst complex component 5**
Folh1	NM_057185	0.80	-38.9	23.2	turq	10723523	folate hydrolase 1
Gatm	NM_031031	0.83	-53.9	11.0	turq	10849290	glycine amidinotransferase (L-arginine:glycine amidinotransferase)
Ggh	NM_012960	0.81	-34.9	41.0	turq	10867821	gamma-glutamyl hydrolase (conjugase, folylpolygammaglutamyl hydrolase)
Gja1	NM_012567	0.82	-176	8.0	turq	10830189	gap junction protein, alpha 1
Gldc	NM_001107583	0.71	-42.0	29.4	turq	10729635	glycine dehydrogenase (decarboxylating)
**Glo1**	**NM_207594**	**0.83**	**-117**	**53.0**	**turq**	**10832115**	**glyoxalase 1**
Glud1	NM_012570	0.81	-279	14.2	turq	10786969	glutamate dehydrogenase 1
Gsta3	NM_031509	0.82	-139	17.6	turq	10926958	glutathione S-transferase A3
Gsta4	NM_001106840	0.66	-16.4	14.1	turq	10911797	glutathione S-transferase alpha 4
Gyg1	NM_031043	0.77	-36.6	39.6	turq	10822481	glycogenin 1
Gykl1	NM_134341	1.20	1.9	15.5	turq	10801717	glycerol kinase-like 1
Hadh	NM_057186	0.79	-55.4	21.7	turq	10826828	hydroxyacyl-Coenzyme A dehydrogenase
Hscb	NM_001108340	0.82	-14.2	10.0	turq	10759241	HscB iron-sulfur cluster co-chaperone homolog (E. coli)
Hsdl2	NM_001025697	0.83	-43.3	11.9	turq	10869267	hydroxysteroid dehydrogenase like 2
Idh1	NM_031510	0.78	-85.6	31.6	turq	10928563	isocitrate dehydrogenase 1 (NADP+), soluble
Inpp4b	NM_053917	0.76	-15.2	8.0	turq	10810378	inositol polyphosphate-4-phosphatase, type II
Isoc1	NM_001014242	0.75	-42.4	41.5	turq	10801929	isochorismatase domain containing 1
Kcna5	NM_012972	0.77	-6.7	15.7	turq	10865730	potassium voltage-gated channel, shaker-related subfamily, member 5
Kcnc3	NM_053997	1.20	10.8	16.7	turq	10706597	potassium voltage gated channel, Shaw-related subfamily, member 3
Kcnmb1	NM_019273	1.20	2.5	35.7	turq	10732716	potassium large conductance calcium-activated channel, subfamily M, beta member 1
Klk8	NM_001107509	1.27	4.6	31.3	turq	10706424	kallikrein related-peptidase 8
Lcp1	NM_001012044	0.71	-26.8	29.8	turq	10781496	lymphocyte cytosolic protein 1
LOC314140	NM_001009694	0.81	-13.9	5.4	turq	10884698	ribose-phosphate pyrophosphokinase I -like
LOC684506	ENSRNOT00000043300	1.20	2.4	41.8	turq	10754876	similar to SMP3 mannosyltransferase
Maoa	NM_033653	0.79	-46.8	27.6	turq	10936717	monoamine oxidase A
Maob	NM_013198	0.74	-110	10.7	turq	10932211	monoamine oxidase B
Mccc2	NM_001012177	0.81	-22.7	34.6	turq	10820847	methylcrotonoyl-Coenzyme A carboxylase 2 (beta)
Mfsd6	NM_001106911	0.83	-23.6	17.6	turq	10923191	major facilitator superfamily domain containing 6
Moxd1	ENSRNOT00000061234	0.74	-47.0	9.7	turq	10717240	monooxygenase, DBH-like 1
Mpo	NM_001107036	1.25	2.3	27.8	turq	10737310	myeloperoxidase
Nanp	NM_001009409	0.78	-14.6	41.5	turq	10850714	N-acetylneuraminic acid phosphatase
Nnt	NM_001013157	0.77	-48.8	43.3	turq	10821556	nicotinamide nucleotide transhydrogenase
Nup155	NM_053952	0.79	-21.6	32.5	turq	10813472	nucleoporin 155
Pank3	NM_001108272	0.74	-79.2	29.8	turq	10732789	pantothenate kinase 3
Papss2	NM_001106375	0.79	-7.6	10.2	turq	10714818	3'-phosphoadenosine 5'-phosphosulfate synthase 2
Pgcp	NM_031640	0.73	-20.8	43.7	turq	10896028	plasma glutamate carboxypeptidase
Pgm3	NM_001108772	0.74	-17.8	33.6	turq	10919090	phosphoglucomutase 3
Pigy	NM_001024370	0.83	-35.1	27.9	turq	10855850	phosphatidylinositol glycan anchor biosynthesis, class Y
Pla2g2d	NM_001013428	1.22	3.4	16.3	turq	10873336	phospholipase A2, group IID
Pla2g7	NM_001009353	0.74	-85.3	34.1	turq	10926683	phospholipase A2, group VII (platelet-activating factor acetylhydrolase, plasma)
Plcd4	NM_080688	0.81	-16.1	9.0	turq	10924335	phospholipase C, delta 4
Pon2	NM_001013082	0.72	-152	28.4	turq	10860888	paraoxonase 2
**Pop4**	**NM_001009642**	**0.82**	**-15.6**	**69.1**	**turq**	**10721191**	**processing of precursor 4, ribonuclease P**
**Ppap2a**	**NM_022538**	**0.81**	**-32.9**	**49.1**	**turq**	**10813048**	**phosphatidic acid phosphatase type 2A**
Ppap2b	NM_138905	0.77	-267	18.2	turq	10870481	phosphatidic acid phosphatase type 2B
Prtfdc1	NM_001106127	0.79	-111	29.8	turq	10799968	phosphoribosyl transferase domain containing 1
Psat1	NM_198738	0.71	-141	26.3	turq	10729096	phosphoserine aminotransferase 1
Ptp4a1	NM_031579	0.82	-92.0	41.7	turq	10923470	protein tyrosine phosphatase 4a1
Pts	NM_017220	0.78	-4.9	10.1	turq	10917209	6-pyruvoyl-tetrahydropterin synthase
**Pus3**	**NM_001108134**	**0.70**	**-10.7**	**56.5**	**turq**	**10908990**	**pseudouridylate synthase 3**
Pxmp3	NM_017234	0.81	-16.4	40.6	turq	10814379	peroxisomal membrane protein 3
RGD1309102	NM_001106160	0.82	-32.1	29.1	turq	10803405	similar to TRS85 homolog
RGD1562284	NM_001134557	0.80	-5.2	37.5	turq	10882461	similar to Glutaminyl-peptide cyclotransferase precursor (QC)

RGD1565002	ENSRNOT00000007645	0.82	-46.1	23.9	turq	10890537	similar to Dehydrogenase/reductase SDR family member 7 precursor (Retinal short-chain dehy
Rtn4	NM_031831	0.81	-143	9.8	turq	10774766	reticulon 4
Sccpdh	NM_001013985	0.82	-58.6	33.0	turq	10766105	saccharopine dehydrogenase (putative)
Sepp1	NM_019192	0.79	-110	44.2	turq	10813236	selenoprotein P, plasma, 1
Sgpp1	ENSRNOT00000006913	0.75	-40.2	41.0	turq	10890650	sphingosine-1-phosphate phosphatase 1
**Sh3bgrl**	**NM_001173339**	**0.81**	**-72.3**	**54.7**	**turq**	**10934669**	**SH3 domain binding glutamic acid-rich protein like**
Slc14a1	NM_019346	0.83	-52.8	14.7	turq	10805335	solute carrier family 14 (urea transporter), member 1
Slc15a2	NM_031672	0.76	-88.8	18.0	turq	10751404	solute carrier family 15 (H+
Slc16a12	ENSRNOT00000030024	0.79	-8.1	2.9	turq	10729795	solute carrier family 16, member 12 (monocarboxylic acid transporter 12)
Slc1a2	NM_001035233	0.83	-463	20.3	turq	10838130	solute carrier family 1 (glial high affinity glutamate transporter), member 2
Slc1a3	NM_019225	0.80	-256	14.1	turq	10821824	solute carrier family 1 (glial high affinity glutamate transporter), member 3
Slc25a40	NM_001037186	0.83	-14.5	3.9	turq	10853292	solute carrier family 25, member 40
Slc30a10	NM_001105985	0.83	-11.7	8.4	turq	10766556	solute carrier family 30, member 10
Slc31a1	NM_133600	0.83	-28.3	39.3	turq	10869310	solute carrier family 31 (copper transporters), member 1
Slc35f1	NM_001109338	0.78	-44.1	4.6	turq	10830164	solute carrier family 35, member F1
Slc7a2	NM_022619	0.81	-20.0	29.1	turq	10791902	solute carrier family 7 (cationic amino acid transporter, y+ system), member 2
Smpdl3a	NM_001005539	0.69	-30.5	47.1	turq	10830230	sphingomyelin phosphodiesterase, acid-like 3A
Ston2	NM_001135874	0.78	-64.7	14.9	turq	10891445	stonin 2
Suclg2	NM_001100750	0.76	-94.7	32.4	turq	10864302	succinate-CoA ligase, GDP-forming, beta subunit
**Sult1d1**	**NM_021769**	**0.65**	**-46.1**	**69.8**	**turq**	**10771919**	**sulfotransferase family 1D, member 1**
Timm8a1	NM_053370	0.78	-27.8	18.2	turq	10939289	translocase of inner mitochondrial membrane 8 homolog a1 (yeast)
**Tmed10**	**NM_053467**	**0.83**	**-62.5**	**58.8**	**turq**	**10891293**	**transmembrane emp24-like trafficking protein 10 (yeast)**
**Tmed2**	**NM_031722**	**0.83**	**-110**	**56.0**	**turq**	**10761718**	**transmembrane emp24 domain trafficking protein 2**
Tmed7	NM_001105758	0.78	-90.2	46.5	turq	10804391	transmembrane emp24 protein transport domain containing 7
Trak2	NM_133560	0.83	-61.1	4.5	turq	10928307	trafficking protein, kinesin binding 2
Trappc2	NM_001024965	0.81	-48.7	44.3	turq	10937660	trafficking protein particle complex 2
Ttpa	NM_013048	0.76	-20.9	45.3	turq	10867815	tocopherol (alpha) transfer protein
**Proteolysis**							
March7	NM_001012087	0.81	-46.5	37.0	turq	10836394	membrane-associated ring finger (C3HC4) 7
Adam17	NM_020306	0.79	-15.2	10.6	turq	10889339	ADAM metallopeptidase domain 17
Cpm	NM_001108098	0.74	-13.2	10.0	turq	10895589	carboxypeptidase M
Ctsl1	NM_013156	0.79	-43.0	30.9	turq	10793458	cathepsin L1
Ctss	NM_017320	0.72	-289	34.2	turq	10817429	cathepsin S
Cul2	NM_001108417	0.76	-56.5	28.7	turq	10798856	cullin 2
Erap1	NM_030836	0.80	-19.6	35.8	turq	10812162	endoplasmic reticulum aminopeptidase 1
Ermp1	NM_184050	0.81	-35.4	41.1	turq	10729610	endoplasmic reticulum metallopeptidase 1
Itch	NM_001005887	0.82	-45.0	46.4	turq	10841301	itchy E3 ubiquitin protein ligase homolog (mouse)
**LOC681578**	**NM_001109444**	**0.72**	**-141**	**72.6**	**turq**	**10815512**	**similar to ring finger protein 13**
LOC690476	ENSRNOT00000058730	1.22	3.8	12.6	turq	10751864	similar to ubiquitin carboxyl-terminal hydrolase CYLD
Lonrf3	ENSRNOT00000017550	0.83	-18.0	37.6	turq	10936346	LON peptidase N-terminal domain and ring finger 3
Mcpt4l1	XM_001056983	1.22	2.5	33.8	turq	10780538	mast cell protease 4-like 1
Pkia	NM_053772	0.75	-93.8	18.6	turq	10822386	protein kinase (cAMP-dependent, catalytic) inhibitor alpha
Pkn2	NM_001105755	0.82	-28.2	7.2	turq	10827105	protein kinase N2
Pm20d2	NM_001107922	0.81	-5.7	24.6	turq	10875853	peptidase M20 domain containing 2
Psmd10	NM_053925	0.82	-11.4	21.9	turq	10822092	proteasome (prosome, macropain) 26S subunit, non-ATPase, 10
Psme4	NM_001025140	0.83	-37.0	17.3	turq	10774830	proteasome (prosome, macropain) activator subunit 4
**Rchy1**	**NM_001007618**	**0.74**	**-40.1**	**69.3**	**turq**	**10771751**	**ring finger and CHY zinc finger domain containing 1**
RGD1561819	ENSRNOT00000059660	1.35	4.7	14.4	turq	10784046	similar to Natural killer cell protease 1 precursor (RNKP-1) (Granzyme B)
Rnf133	NM_001044278	1.29	3.3	18.6	turq	10861297	ring finger protein 133
Rnf135	ENSRNOT00000005428	1.26	2.0	35.0	turq	10736636	ring finger protein 135
rnf141	NM_001001800	0.78	-114	40.1	turq	10724888	ring finger protein 141
**Rspry1**	**NM_001100945**	**0.81**	**-22.5**	**52.4**	**turq**	**10809286**	**ring finger and SPRY domain containing 1**
Scpep1	NM_133383	0.78	-52.2	21.2	turq	10746139	serine carboxypeptidase 1
**Ubxn8**	**NM_001106086**	**0.83**	**-13.3**	**49.0**	**turq**	**10788583**	**UBX domain protein 8**
Ufc1	NM_001003709	0.75	-56.3	31.4	turq	10769862	ubiquitin-fold modifier conjugating enzyme 1
Ufm1	NM_001126080	0.81	-16.4	46.5	turq	10823174	ubiquitin-fold modifier 1
Usp53	NM_001106468	0.80	-46.6	16.9	turq	10826517	ubiquitin specific peptidase 53
Wwp1	NM_001024757	0.77	-77.8	24.4	turq	10875698	WW domain containing E3 ubiquitin protein ligase 1
**Receptors & Binding Proteins**							
Abca1	NM_178095	0.83	-39.5	7.7	turq	10876769	ATP-binding cassette, sub-family A (ABC1), member 1
Adora2a	NM_053294	0.63	-14.7	10.3	turq	10832577	adenosine A2a receptor
Adora2b	NM_017161	0.77	-9.2	16.7	turq	10734291	adenosine A2B receptor
**Bmpr1a**	**NM_030849**	**0.73**	**-87.4**	**51.0**	**turq**	**10790481**	**bone morphogenetic protein receptor, type IA**
Bmpr1b	NM_001024259	0.76	-22.5	31.9	turq	10827068	bone morphogenetic protein receptor, type IB
Cacybp	NM_001004208	0.78	-67.2	40.5	turq	10769131	calcyclin binding protein
Calcrl	NM_012717	0.66	-24.0	48.3	turq	10846762	calcitonin receptor-like
Ednrb	NM_017333	0.67	-262	45.9	turq	10785724	endothelin receptor type B
Epha3	NM_031564	0.70	-24.4	35.0	turq	10752630	Eph receptor A3
Ephb1	NM_001104528	0.80	-18.2	44.8	turq	10919590	Eph receptor B1

**Ffar3**	**NM_001108912**	**1.22**	**3.7**	**52.8**	**turq**	**10720792**	**free fatty acid receptor 3**
Fzd8	NM_001044251	0.63	-24.6	30.7	turq	10795673	frizzled homolog 8 (Drosophila)
Gabra3	NM_017069	0.75	-88.9	10.2	turq	10940090	gamma-aminobutyric acid (GABA) A receptor, alpha 3
**Gabrg1**	**NM_080586**	**0.71**	**-141**	**48.7**	**turq**	**10772544**	**gamma-aminobutyric acid (GABA) A receptor, gamma 1**
Gca	NM_001106483	0.80	-41.0	12.8	turq	10836446	grancalcin
Glra2	NM_012568	0.65	-16.0	17.1	turq	10933393	glycine receptor, alpha 2
Htr1b	NM_022225	0.77	-18.3	22.2	turq	10918979	5-hydroxytryptamine (serotonin) receptor 1B
Ifngr1	NM_053783	0.83	-31.9	17.0	turq	10701924	interferon gamma receptor 1
Il7r	NM_001106418	0.79	-4.1	18.6	turq	10821851	interleukin 7 receptor
Itgb1bp1	NM_001106719	0.81	-15.2	22.2	turq	10889331	integrin beta 1 binding protein 1
Itgb3bp	NM_001013213	0.83	-5.1	4.3	turq	10878272	integrin beta 3 binding protein (beta3-endonexin)
Ivns1abp	NM_001047085	0.79	-170	30.0	turq	10764626	influenza virus NS1A binding protein
LOC302473	NM_001106947	0.77	-43.4	21.5	turq	10940016	similar to SLIT and NTRK-like family, member 4
LOC684623	ENSRNOT00000003659	0.81	-14.7	13.7	turq	10769177	similar to Probable G-protein coupled receptor 52
LOC684994	ENSRNOT00000033048	0.83	-11.6	10.9	turq	10782469	similar to retinoic acid receptor, beta
Lpar4	NM_001106940	0.82	-6.0	16.7	turq	10934631	lysophosphatidic acid receptor 4
Lrp4	NM_031322	0.81	-24.6	20.9	turq	10837881	low density lipoprotein receptor-related protein 4
Mfrp	NM_001108137	1.28	4.3	7.9	turq	10909428	membrane frizzled-related protein
Olr1129	NM_001000879	1.30	4.5	0.0	grey	10915259	olfactory receptor 1129
Olr1225	NM_001001083	1.22	3.0	14.8	turq	10909151	olfactory receptor 1225
Olr1249	NM_001001020	1.23	3.8	12.3	turq	10909201	olfactory receptor 1249
Olr1328	NM_001000599	1.29	3.6	29.8	turq	10909302	olfactory receptor 1328
Olr1362	NM_001006598	1.30	4.1	33.6	turq	10740778	olfactory receptor 1362
Olr1382	NM_214831	1.23	3.7	13.9	turq	10740810	olfactory receptor 1382
Olr1387	NM_001000001	1.21	3.6	41.2	turq	10733072	olfactory receptor 1387
Olr1425	NM_001000010	1.44	5.0	8.1	turq	10742940	olfactory receptor 1425
Olr1681	NM_001001006	1.24	3.0	16.2	turq	10830773	olfactory receptor 1681
Olr510	NM_001000312	1.21	2.3	13.1	turq	10846996	olfactory receptor 510
**Olr550**	**NM_001000322**	**1.37**	**19.8**	**54.5**	**turq**	**10847051**	**olfactory receptor 550**
Olr624	NM_001000650	1.24	4.4	21.9	turq	10837580	olfactory receptor 624
Olr717	NM_001000621	1.25	3.5	19.4	turq	10847225	olfactory receptor 717
Olr769	NM_001000371	1.20	5.1	14.4	turq	10848113	olfactory receptor 769
Olr775	NM_001000374	1.23	2.2	8.9	turq	10848118	olfactory receptor 775
Olr917	NM_001001354	1.24	4.3	12.6	turq	10900006	olfactory receptor 917
P2ry12	NM_022800	0.70	-53.7	24.6	turq	10823365	purinergic receptor P2Y, G-protein coupled, 12
P2ry13	NM_001002853	0.70	-32.2	27.2	turq	10823363	purinergic receptor P2Y, G-protein coupled, 13
Pgrmc1	NM_021766	0.80	-94.5	39.7	turq	10936341	progesterone receptor membrane component 1
Reep3	NM_001106386	0.82	-22.7	26.6	turq	10829816	receptor accessory protein 3
Rorb	ENSRNOT00000018137	0.77	-40.0	16.9	turq	10729256	RAR-related orphan receptor B
Sec13	NM_001006978	0.81	-41.0	33.0	turq	10864680	SEC13 homolog (S. cerevisiae)
Sec23b	NM_001108593	0.80	-20.3	13.1	turq	10840396	Sec23 homolog B (S. cerevisiae)
Strn	NM_019148	0.83	-38.5	12.3	turq	10887842	striatin, calmodulin binding protein
Taar5	NM_001009650	1.24	2.6	20.7	turq	10717284	trace amine-associated receptor 5
Tax1bp1	NM_001004199	0.79	-57.5	30.5	turq	10855576	Tax1 (human T-cell leukemia virus type I) binding protein 1
Tlr7	NM_001097582	0.75	-8.1	32.7	turq	10933345	toll-like receptor 7
Unc5b	NM_022207	0.75	-9.5	12.5	turq	10833013	unc-5 homolog B (C. elegans)
V1rm6	NM_001008930	1.24	3.7	14.7	turq	10704202	vomeronasal 1 receptor, M6
Vom2r56	NM_001099484	1.23	5.2	24.4	turq	10901190	vomeronasal 2 receptor, 56
**Signaling**							
Ak3	NM_013218	0.80	-40.5	33.4	turq	10729590	adenylate kinase 3
Akirin1	NM_001030054	0.83	-38.6	44.3	turq	10879667	akirin 1
Alcam	NM_031753	0.77	-132	27.2	turq	10750878	activated leukocyte cell adhesion molecule
Alpk1	ENSRNOT00000030798	0.78	-9.2	33.2	turq	10826680	alpha-kinase 1
Anp32e	NM_001013200	0.77	-57.7	19.9	turq	10817459	acidic (leucine-rich) nuclear phosphoprotein 32 family, member E
Anxa3	NM_012823	0.74	-44.1	29.2	turq	10775628	annexin A3
Apln	NM_031612	0.81	-14.4	12.3	turq	10939564	apelin
Appl2	NM_001108741	0.80	-25.6	11.1	turq	10894560	adaptor protein, phosphotyrosine interaction, PH domain and leucine zipper containing 2
Arhgdib	NM_001009600	0.82	-8.6	14.9	turq	10866526	Rho, GDP dissociation inhibitor (GDI) beta
Arrdc3	NM_001007797	0.75	-61.3	38.0	turq	10834022	arrestin domain containing 3
Ascc3	NM_001107640	0.82	-22.8	20.3	turq	10830699	activating signal cointegrator 1 complex subunit 3
Atl3	NM_001044241	0.83	-14.8	31.0	turq	10713520	atlastin GTPase 3
Bmp2k	ENSRNOT00000060868	0.81	-18.5	23.3	turq	10775606	BMP-2 inducible kinase
Caln1	NM_001077201	0.78	-46.4	3.6	turq	10761301	calneuron 1
**Camk2d**	**NM_012519**	**0.81**	**-45.7**	**48.7**	**turq**	**10818920**	**calcium**
Cdc42ep3	NM_001048044	0.81	-11.6	17.8	turq	10887939	CDC42 effector protein (Rho GTPase binding) 3
Cdk5rap1	NM_145721	0.82	-5.6	17.1	turq	10850903	CDK5 regulatory subunit associated protein 1
Chn2	NM_032084	0.82	-22.6	20.3	turq	10855615	chimerin (chimaerin) 2
Cks1b	NM_001135749	0.80	-4.9	8.2	turq	10878032	CDC28 protein kinase regulatory subunit 1B
Csrnp1	NM_001108786	0.82	-2.7	25.2	turq	10920967	cysteine-serine-rich nuclear protein 1

Ctxn2	NM_001162935	0.69	-13.8	15.0	turq	10839365	cortexin 2
Dock1	NM_001143858	0.81	-26.0	39.0	turq	10711791	dedicator of cyto-kinesis 1
Dok6	ENSRNOT00000058139	0.73	-126	15.1	turq	10805605	docking protein 6
Dusp15	NM_001108598	0.78	-10.8	15.7	turq	10850841	dual specificity phosphatase 15
Dusp6	NM_053883	0.83	-21.4	36.1	turq	10895144	dual specificity phosphatase 6
Emr1	NM_001007557	0.76	-8.7	36.2	turq	10931222	EGF-like module containing, mucin-like, hormone receptor-like 1
Enpp4	NM_001106892	0.78	-36.8	9.9	turq	10921910	ectonucleotide pyrophosphatase
Eps8	ENSRNOT00000009328	0.83	-19.9	8.0	turq	10866544	epidermal growth factor receptor pathway substrate 8
Fastkd5	XR_086207	0.79	-11.4	31.9	turq	10849925	FAST kinase domains 5
Fkbp10	NM_001014120	0.79	-6.3	17.2	turq	10738177	FK506 binding protein 10
Gna13	NM_001013119	0.78	-48.1	38.3	turq	10748537	guanine nucleotide binding protein, alpha 13
Gnai1	NM_013145	0.81	-89.1	29.1	turq	10853229	guanine nucleotide binding protein (G protein), alpha inhibiting 1
Gnai3	NM_013106	0.75	-60.5	41.2	turq	10825962	guanine nucleotide binding protein (G protein), alpha inhibiting 3
Gnal	ENSRNOT00000025172	0.66	-92.0	31.2	turq	10802391	guanine nucleotide binding protein, alpha stimulating, olfactory type
Gnb4	NM_001013910	0.65	-37.2	27.5	turq	10822717	guanine nucleotide binding protein (G protein), beta polypeptide 4
Gng12	ENSRNOT00000007403	0.72	-46.3	31.8	turq	10856050	guanine nucleotide binding protein (G protein), gamma 12
Gpatch4	NM_001024979	0.78	-14.9	17.3	turq	10816418	G patch domain containing 4
Gpr137b	NM_001105978	0.82	-19.5	0.0	grey	10795679	G protein-coupled receptor 137B
Gpr183	NM_001109386	0.82	-4.2	9.7	turq	10786028	G protein-coupled receptor 183
Gpr34	NM_001024925	0.74	-28.2	16.6	turq	10936742	G protein-coupled receptor 34
Gpr88	NM_031696	0.67	-15.3	25.9	turq	10826261	G-protein coupled receptor 88
Hipk1	NM_001100986	0.81	-56.6	28.2	turq	10825582	homeodomain interacting protein kinase 1
Hrsp12	NM_031714	0.77	-80.0	39.4	turq	10903227	heat-responsive protein 12
Hspa13	NM_019271	0.79	-77.3	35.4	turq	10752738	heat shock protein 70kDa family, member 13
Insig1	NM_022392	0.81	-82.3	38.4	turq	10859880	insulin induced gene 1
Insig2	NM_178091	0.80	-32.1	11.6	turq	10767175	insulin induced gene 2
Ints4	ENSRNOT00000016770	0.82	-21.6	39.0	turq	10708785	integrator complex subunit 4
Itga6	ENSRNOT00000045394	0.78	-42.7	5.5	turq	10836849	integrin, alpha 6
Itgb8	NM_001108726	0.72	-90.4	46.4	turq	10892835	integrin, beta 8
LOC500584	ENSRNOT00000051887	0.83	-20.2	37.2	turq	10881461	similar to casein kinase 1, gamma 3 isoform 2
Map4k4	NM_001106904	0.82	-39.2	9.9	turq	10922783	mitogen-activated protein kinase kinase kinase kinase 4
Mapk1ip1l	NM_001108373	0.78	-38.4	15.4	turq	10779671	mitogen-activated protein kinase 1 interacting protein 1-like
Mertk	NM_022943	0.79	-33.2	12.3	turq	10839674	c-mer proto-oncogene tyrosine kinase
MGC109340	NM_001024267	0.71	-74.7	18.8	turq	10935031	similar to Microsomal signal peptidase 23 kDa subunit (SPase 22 kDa subunit) (SPC22
**Mobkl1a**	**NM_001108357**	**0.80**	**-21.9**	**53.6**	**turq**	**10776034**	**MOB1, Mps One Binder kinase activator-like 1A (yeast)**
Mobkl1b	NM_001033891	0.81	-38.5	28.9	turq	10856707	MOB1, Mps One Binder kinase activator-like 1B (yeast)
Nek7	NM_001108346	0.77	-45.9	15.0	turq	10768177	NIMA (never in mitosis gene a)-related kinase 7
Nucks1	NM_022799	0.80	-112	38.5	turq	10887667	nuclear casein kinase and cyclin-dependent kinase substrate 1
Oxsr1	NM_001108194	0.82	-35.4	2.7	turq	10920841	oxidative-stress responsive 1
RGD1303142	NM_201560	0.81	-8.9	20.6	turq	10851492	oxidative stress responsive gene
Pak2	NM_053306	0.83	-29.1	34.6	turq	10751671	p21 protein (Cdc42
Pde10a	NM_022236	0.83	-16.8	21.0	turq	10718043	phosphodiesterase 10A
Pde7b	NM_080894	0.70	-32.6	12.9	turq	10717069	phosphodiesterase 7B
Pdk4	NM_053551	0.82	-7.0	20.3	turq	10860900	pyruvate dehydrogenase kinase, isozyme 4
Pdp2	NM_145091	0.79	-8.6	40.0	turq	10809001	pyruvate dehyrogenase phosphatase catalytic subunit 2
Peli1	NM_001100565	0.76	-54.6	28.1	turq	10774375	pellino 1
**Pik3c2a**	**NM_001108500**	**0.76**	**-29.5**	**59.0**	**turq**	**10725114**	**phosphoinositide-3-kinase, class 2, alpha polypeptide**
Plk3	NM_022187	0.82	-3.6	33.0	turq	10878938	polo-like kinase 3 (Drosophila)
Ppp1r15b	NM_001107175	0.83	-41.4	14.7	turq	10763969	protein phosphatase 1, regulatory (inhibitor) subunit 15b
Ppp2ca	NM_017039	0.82	-347	48.3	turq	10733417	protein phosphatase 2, catalytic subunit, alpha isoform
Prkd3	NM_001024263	0.75	-18.3	37.1	turq	10887918	protein kinase D3
Prkrir	ENSRNOT00000021228	0.74	-26.9	36.2	turq	10708903	protein-kinase, interferon-inducible double stranded RNA dependent inhibitor, repressor of
Ptprz1	NM_013080	0.76	-171	19.1	turq	10853963	protein tyrosine phosphatase, receptor-type, Z polypeptide 1
Rab14	NM_053589	0.82	-176	33.1	turq	10844658	RAB14, member RAS oncogene family
Rab21	NM_001004238	0.80	-98.9	47.1	turq	10902393	RAB21, member RAS oncogene family
**Rab27a**	**NM_017317**	**0.77**	**-10.5**	**53.8**	**turq**	**10911524**	**RAB27A, member RAS oncogene family**
**Rab2b**	**NM_001037645**	**0.77**	**-39.0**	**55.0**	**turq**	**10783320**	**RAB2B, member RAS oncogene family**
Rab9a	NM_053458	0.75	-34.3	33.8	turq	10933366	RAB9A, member RAS oncogene family
Rasgrp2	NM_001082977	0.77	-5.8	28.3	turq	10713402	RAS guanyl releasing protein 2 (calcium and DAG-regulated)
Rasgrp3	NM_001108009	0.69	-28.1	13.4	turq	10888404	RAS guanyl releasing protein 3 (calcium and DAG-regulated)
RGD1560166	ENSRNOT00000033018	0.81	-5.1	18.8	turq	10919924	similar to Probable G-protein coupled receptor 62 (hGPCR8)
RGD1561963	ENSRNOT00000064161	0.83	-32.1	22.5	turq	10929321	similar to Dedicator of cytokinesis protein 10 (Protein zizimin 3)
Rin2	NM_001107786	0.82	-12.4	21.6	turq	10840448	Ras and Rab interactor 2
Riok2	NM_001009687	0.74	-29.8	36.3	turq	10703403	RIO kinase 2 (yeast)
Rnd3	NM_001007641	0.74	-30.1	10.1	turq	10845124	Rho family GTPase 3
**Rps6ka3**	**ENSRNOT00000008938**	**0.78**	**-51.1**	**48.9**	**turq**	**10938025**	**ribosomal protein S6 kinase polypeptide 3**
Sh3glb1	NM_001011929	0.74	-59.9	39.6	turq	10827138	SH3-domain GRB2-like endophilin B1
Shc4	ENSRNOT00000011084	0.76	-11.8	9.7	turq	10849423	SHC (Src homology 2 domain containing) family, member 4
Skap2	NM_130413	0.82	-16.3	9.2	turq	10862527	src kinase associated phosphoprotein 2

Smek1	NM_001108050	0.80	-26.0	34.3	turq	10891719	SMEK homolog 1, suppressor of mek1 (Dictyostelium)
Snx6	NM_001108711	0.80	-50.2	39.7	turq	10889944	sorting nexin 6
**Sppl2a**	**NM_001107770**	**0.80**	**-43.2**	**49.7**	**turq**	**10849598**	**signal peptide peptidase-like 2A**
**Srp54a**	NM_053871	**0.79**	**-21.2**	**55.8**	**turq**	**10884642**	**signal recognition particle 54a**
Ssr3	NM_031120	0.81	-108	31.7	turq	10823500	signal sequence receptor, gamma
Stk17b	NM_133392	0.76	-29.6	18.4	turq	10927992	serine/threonine kinase 17b
Tbc1d16	XM_221188	0.80	-9.4	22.3	turq	10931651	TBC1 domain family, member 16
Cnn3	NM_019359	0.75	-94.4	28.5	turq	10818698	calponin 3, acidic
**Transcription**							
**Ankra2**	**NM_207595**	**0.80**	**-24.4**	**52.4**	**turq**	**10812698**	**ankyrin repeat, family A (RFXANK-like), 2**
Bcl6	NM_001107084	0.81	-15.6	26.3	turq	10751931	B-cell CLL/lymphoma 6
Blzf1	NM_001017494	0.82	-10.3	14.8	turq	10769468	basic leucine zipper nuclear factor 1
Btaf1	ENSRNOT00000024465	0.83	-25.0	15.5	turq	10715025	BTAF1 RNA polymerase II, B-TFIID transcription factor-associated, (Mot1 homolog, S. cerevisiae
Bzw1	NM_198789	0.83	-119	18.0	turq	10923560	basic leucine zipper and W2 domains 1
Ccdc153	NM_001013953	1.22	4.7	38.6	turq	10909463	coiled-coil domain containing 153
Cdig2	ENSRNOT00000016167	0.76	-44.8	10.4	turq	10803238	Cdig2 protein <cadmium-inducible gene; transcription upregulated by cadmium >
Ctcf	NM_031824	0.81	-58.8	17.3	turq	10807300	CCCTC-binding factor (zinc finger protein)
Dhx38	NM_001106185	0.81	-8.0	24.1	turq	10810896	DEAH (Asp-Glu-Ala-His) box polypeptide 38
Dhx40	NM_001005873	0.79	-39.5	32.8	turq	10745974	DEAH (Asp-Glu-Ala-His) box polypeptide 40
Dhx9	NM_001107184	0.81	-77.1	34.7	turq	10768697	DEAH (Asp-Glu-Ala-His) box polypeptide 9
Dnajb9	NM_012699	0.82	-27.1	35.0	turq	10889766	DnaJ (Hsp40) homolog, subfamily B, member 9
Dnajc30	NM_001109024	0.76	-7.8	27.6	turq	10761225	DnaJ (Hsp40) homolog, subfamily C, member 30
Dr1	NM_001011914	0.77	-47.6	29.1	turq	10775080	down-regulator of transcription 1
E2f5	ENSRNOT00000014361	0.60	-9.9	41.9	turq	10718609	E2F transcription factor 5
Fam134b	NM_001034912	0.79	-54.5	38.4	turq	10813934	family with sequence similarity 134, member B
**Fbxo30**	**NM_001007690**	**0.75**	**-19.8**	**61.2**	**turq**	**10701788**	**F-box protein 30**
Foxp2	XM_001056575	0.77	-4.1	14.7	turq	10853783	forkhead box P2
Gli3	NM_080405	0.82	-7.3	13.7	turq	10798706	GLI-Kruppel family member GLI3
Gzf1	NM_001107788	0.77	-15.2	20.8	turq	10840571	GDNF-inducible zinc finger protein 1
Higd1a	NM_080902	0.80	-32.5	18.7	turq	10914415	HIG1 hypoxia inducible domain family, member 1A
Hras	NM_001130441	1.26	19.4	13.0	turq	10938817	Harvey rat sarcoma virus oncogene
Kank1	NM_001037197	0.78	-16.4	15.4	turq	10714505	KN motif and ankyrin repeat domains 1
Klf10	NM_031135	0.83	-5.7	12.5	turq	10903459	Kruppel-like factor 10
**Klhl9**	**NM_001107944**	**0.82**	**-111**	**58.7**	**turq**	**10877984**	**kelch-like 9 (Drosophila)**
Lhfpl2	NM_001106402	0.83	-10.9	13.1	turq	10812526	lipoma HMGIC fusion partner-like 2
Litaf	NM_001105735	0.83	-8.2	20.3	turq	10731493	lipopolysaccharide-induced TNF factor
LOC100125361	NM_001109677	0.81	-8.7	19.3	turq	10743144	zinc finger protein LOC100125361
Lyn	NM_030857	0.80	-8.2	29.5	turq	10867461	v-yes-1 Yamaguchi sarcoma viral related oncogene homolog
Lzic	NM_001013241	0.81	-15.2	24.4	turq	10874080	leucine zipper and CTNNBIP1 domain containing
Mageh1	NM_001013250	0.81	-19.2	17.6	turq	10937492	melanoma antigen, family H, 1
Mcc	NM_001170534	0.82	-8.6	2.2	turq	10804316	mutated in colorectal cancers
Mpeg1	NM_022617	0.72	-24.9	28.9	turq	10714103	macrophage expressed gene 1
Mtf1	NM_001108677	0.81	-17.4	18.9	turq	10871857	metal-regulatory transcription factor 1
Nfatc3	NM_001108447	0.83	-4.2	13.7	turq	10807452	nuclear factor of activated T-cells, cytoplasmic, calcineurin-dependent 3
Nfe2l2	NM_031789	0.71	-45.8	36.1	turq	10846286	nuclear factor, erythroid derived 2, like 2
Nfia	NM_012988	0.78	-50.0	19.0	turq	10870020	nuclear factor I/A
Nr2f2	NM_080778	0.83	-16.7	31.5	turq	10722736	nuclear receptor subfamily 2, group F, member 2
**Nr3c1**	**NM_012576**	**0.79**	**-57.8**	**50.4**	**turq**	**10804132**	**nuclear receptor subfamily 3, group C, member 1**
Nr4a2	NM_019328	0.51	-63.4	32.6	turq	10845384	nuclear receptor subfamily 4, group A, member 2
Nras	NM_080766	0.79	-42.1	1.9	turq	10817938	neuroblastoma ras oncogene
Nudt2	NM_207596	0.82	-7.1	18.3	turq	10755257	nudix (nucleoside diphosphate linked moiety X)-type motif 2
Nxt2	NM_001108120	0.82	-4.3	28.0	turq	10937391	nuclear transport factor 2-like export factor 2
Pla2g16	NM_017060	0.77	-24.4	21.2	turq	10713538	phospholipase A2, group XVI
Ptgr2	NM_001015009	0.78	-59.0	33.9	turq	10885892	prostaglandin reductase 2
Pura	ENSRNOT00000025756	0.80	-37.3	18.9	turq	10800991	purine rich element binding protein A
Rfx3	NM_001012172	0.82	-48.2	23.1	turq	10729525	regulatory factor X, 3 (influences HLA class II expression)
RGD1563838	ENSRNOT00000033811	0.65	-189	30.6	turq	10707357	similar to leucine zipper protein 2
**RGD1564836**	**ENSRNOT00000038940**	**1.33**	**27.6**	**60.4**	**turq**	**10937775**	**similar to HMGA1b**
Sin3a	NM_001108761	0.82	-20.0	8.2	turq	10910270	SIN3 homolog A, transcription regulator (yeast)
Smarca5	NM_001107419	0.77	-57.4	46.1	turq	10806937	SWI/SNF related, matrix associated, actin dependent regulator of chromatin, subfamily a, mem
Spop	NM_001100496	0.80	-100	20.7	turq	10737609	speckle-type POZ protein
Ssr1	NM_001008891	0.83	-42.1	32.8	turq	10794721	signal sequence receptor, alpha
Stat1	NM_032612	0.81	-21.8	23.3	turq	10927842	signal transducer and activator of transcription 1
Stx7	NM_021869	0.81	-54.8	31.3	turq	10717256	syntaxin 7
Supv3l1	NM_001012462	0.82	-23.4	19.6	turq	10833154	suppressor of var1, 3-like 1 (S. cerevisiae)
Tcea1	NM_001025735	0.80	-69.8	39.7	turq	10779353	transcription elongation factor A (SII) 1
Tdpoz1	XM_345239	1.32	7.6	31.3	turq	10824790	TD and POZ domain containing 1
**Tiparp**	**NM_001107679**	**0.64**	**-36.4**	**49.1**	**turq**	**10815763**	**TCDD-inducible poly(ADP-ribose) polymerase**
Tox3	NM_001106171	0.79	-10.0	10.7	turq	10806303	TOX high mobility group box family member 3

Trim24	NM_001044266	1.21	3.6	39.7	turq	10861908	tripartite motif-containing 24
**Trim33**	**ENSRNOT00000025633**	**0.80**	**-37.8**	**61.3**	**turq**	**10817989**	**tripartite motif-containing 33**
Trip4	NM_001134981	0.81	-19.6	20.3	turq	10918302	thyroid hormone receptor interactor 4
Yes1	NM_033298	0.74	-28.8	24.9	turq	10926070	Yamaguchi sarcoma viral (v-yes) oncogene homolog 1
Zbtb20	NM_001105880	0.83	-32.4	11.0	turq	10754116	zinc finger and BTB domain containing 20
Zbtb33	NM_001109314	0.74	-47.5	33.6	turq	10936263	zinc finger and BTB domain containing 33
Zbtb41	ENSRNOT00000015596	0.68	-71.6	47.9	turq	10764404	zinc finger and BTB domain containing 41
Zdbf2	ENSRNOT00000016038	0.71	-25.6	5.2	turq	10923866	zinc finger, DBF-type containing 2
**Zeb1**	**NM_013164**	**0.82**	**-31.9**	**49.8**	**turq**	**10798780**	**zinc finger E-box binding homeobox 1**
Zfand5	NM_001106356	0.83	-89.3	24.0	turq	10714338	zinc finger, AN1-type domain 5
Zfp180	NM_144757	0.83	-50.1	14.4	turq	10704895	zinc finger protein 180
Zfp189	NM_001107930	0.76	-29.8	25.4	turq	10869010	zinc finger protein 189
Zfp191	NM_182955	0.80	-19.9	22.9	turq	10803494	zinc finger protein 191
Zfp275	NM_001106343	0.82	-11.0	30.6	turq	10935878	zinc finger protein 275
Zfp418	ENSRNOT00000020751	0.77	-12.3	13.3	turq	10703818	zinc finger protein 418
**Zfp426l2**	**NM_001134585**	**0.81**	**-12.1**	**50.6**	**turq**	**10908248**	**zinc finger protein 426-like 2**
Zkscan3	NM_001012053	0.83	-10.1	24.6	turq	10795335	zinc finger with KRAB and SCAN domains 3
Zmpste24	NM_001107974	0.83	-53.0	39.3	turq	10879467	zinc metallopeptidase, STE24 homolog (S. cerevisiae)
Znf496	ENSRNOT00000058308	1.24	7.1	5.9	turq	10743150	zinc finger protein 496
Znf654	ENSRNOT00000050533	0.82	-23.1	35.7	turq	10752654	zinc finger protein 654
**Translation & Protein Modification**							
**Arl5a**	**NM_053979**	**0.81**	**-58.9**	**53.7**	**turq**	**10845298**	**ADP-ribosylation factor-like 5A**
Arl8b	NM_001024332	0.81	-206	38.8	turq	10857618	ADP-ribosylation factor-like 8B
Brunol4	NM_001107400	0.78	-104	6.2	turq	10803542	bruno-like 4, RNA binding protein (Drosophila)
Cpsf2	NM_001106753	0.81	-27.3	20.0	turq	10886414	cleavage and polyadenylation specific factor 2
**Eef1e1**	**NM_001106106**	**0.82**	**-50.8**	**65.2**	**turq**	**10794680**	**eukaryotic translation elongation factor 1 epsilon 1**
Eif1a	NM_001008773	0.80	-38.0	36.1	turq	10801541	eukaryotic translation initiation factor 1A
Eif1ay	NM_001106963	0.75	-96.6	39.7	turq	10938019	eukaryotic translation initiation factor 1A, Y-linked
Eif2s2	NM_199380	0.79	-55.5	26.8	turq	10850970	eukaryotic translation initiation factor 2, subunit 2 beta
Eif2s3x	NM_001100542	0.81	-73.9	42.0	turq	10938409	eukaryotic translation initiation factor 2, subunit 3, structural gene X-linked
Eif3j	NM_001077670	0.77	-77.1	17.2	turq	10788079	eukaryotic translation initiation factor 3, subunit J
Etf1	NM_001008344	0.83	-43.3	11.1	turq	10803843	eukaryotic translation termination factor 1
Mrpl12	NM_001029900	0.80	-5.9	19.8	turq	10740153	mitochondrial ribosomal protein L12
Ormdl2	NM_001105940	0.81	-18.2	19.6	turq	10899943	ORM1-like 2 (S. cerevisiae)
Pabpc1	NM_134353	0.77	-152	34.0	turq	10903346	poly(A) binding protein, cytoplasmic 1
Polr1e	NM_001107938	0.79	-8.7	19.5	turq	10868682	polymerase (RNA) I polypeptide E
Pum2	NM_001106715	0.78	-149	39.0	turq	10883510	pumilio homolog 2 (Drosophila)
Rbm18	NM_001107838	0.80	-41.9	31.4	turq	10844747	RNA binding motif protein 18
Rexo2	NM_001008326	0.82	-32.2	25.5	turq	10917095	REX2, RNA exonuclease 2 homolog (S. cerevisiae)
Rpl7a	NM_001114391	1.21	84.3	26.3	turq	10742690	ribosomal protein L7a
Rpp30	ENSRNOT00000025412	0.80	-38.1	36.7	turq	10714952	ribonuclease P/MRP 30 subunit (human)
Rpusd4	NM_001025284	0.83	-3.3	16.1	turq	10908960	RNA pseudouridylate synthase domain containing 4
Taf9b	NM_133615	0.80	-39.0	30.8	turq	10938952	TAF9B RNA polymerase II, TATA box binding protein (TBP)-associated factor
**Tra2b**	**NM_057119**	**0.82**	**-46.3**	**55.9**	**turq**	**10752050**	**transformer 2 beta homolog (Drosophila)**
Zscan12	ENSRNOT00000024906	0.81	-8.0	20.2	turq	10798521	zinc finger and SCAN domain containing 12
**Miscellaneous & Unknown**							
Cbfb	NM_001013191	0.82	-9.4	19.9	turq	10807131	core-binding factor, beta subunit
Fam102b	NM_001163568	0.80	-50.1	7.4	turq	10826137	family with sequence similarity 102, member B
Fam118b	BC098017	0.82	-8.4	21.1	turq	10916052	family with sequence similarity 118, member B
Fam133b	BC094528	0.68	-11.7	28.8	turq	10936823	family with sequence similarity 133, member B
Fam45a	NM_001127681	0.82	-75.2	44.9	turq	10716470	family with sequence similarity 45, member A
Fam49a	NM_001106718	0.83	-107	26.0	turq	10883636	family with sequence similarity 49, member A
Fam63b	ENSRNOT00000020089	0.79	-35.0	33.6	turq	10918535	family with sequence similarity 63, member B
Fam91a1	BC166852	0.83	-49.1	31.1	turq	10896674	family with sequence similarity 91, member A1
Gramd3	NM_001014011	0.79	-48.7	45.9	turq	10801794	GRAM domain containing 3
**LOC100188933**	**NM_001134695**	**0.83**	**-3.8**	**54.5**	**turq**	**10833598**	**hypothetical protein LOC100188933**
LOC100233176	NM_001142941	0.82	-9.8	36.9	turq	10891864	hypothetical protein LOC100233176
LOC682650	ENSRNOT00000060689	1.32	5.0	38.2	turq	10803921	hypothetical protein LOC682650
LOC685501	ENSRNOT00000034801	1.23	2.9	26.5	turq	10912449	hypothetical protein LOC685501
LOC691649	ENSRNOT00000005237	0.72	-58.7	31.9	turq	10734061	hypothetical protein LOC691649
MGC95208	NM_001005552	0.81	-23.6	42.0	turq	10933015	similar to 4930453N24Rik protein
Prb1	L17318	1.31	8.0	24.3	turq	10866332	proline-rich protein BstNI subfamily 1
RGD1303127	NM_001004244	0.78	-40.4	20.1	turq	10906863	similar to hypothetical protein FLJ20436
**RGD1305045**	**NM_001106496**	**0.77**	**-123**	**67.4**	**turq**	**10838506**	**similar to hypothetical protein**
RGD1305089	NM_001106730	0.80	-19.4	14.8	turq	10884648	similar to 1110008L16Rik protein
RGD1306001	NM_001130696	0.79	-5.7	32.2	turq	10905980	similar to 2210021J22Rik protein
RGD1306622	NM_001170487	0.80	-13.8	33.4	turq	10728647	similar to KIAA0954 protein
RGD1307051	NM_001108091	0.80	-9.6	0.0	grey	10902112	similar to hypothetical protein FLJ21963
RGD1307439	NM_001127540	1.23	2.5	38.7	turq	10726740	similar to hypothetical protein MGC35138

RGD1308116	ENSRNOT00000021964	0.75	-29.7	19.6	turq	10821581	similar to hypothetical protein MGC42105
RGD1309621	ENSRNOT00000055437	0.78	-11.8	0.0	grey	10859774	similar to hypothetical protein FLJ10652
RGD1311269	NR_033170	0.81	-6.0	23.9	turq	10928207	similar to hypothetical protein FLJ37953
RGD1560187	NM_001173556	0.83	-59.7	42.8	turq	10880897	similar to Hypothetical UPF0327 protein
RGD1560477	XM_002729098	1.21	3.8	42.2	turq	10824806	similar to TDPOZ3
RGD1562641	ENSRNOT00000029573	1.31	2.7	37.1	turq	10787039	similar to hypothetical protein 4930474N05
RGD1563070	NM_001134541	0.81	-26.9	16.5	turq	10779602	similar to hypothetical protein
RGD1564541	NM_001107950	0.82	-15.6	14.1	turq	10932029	similar to hypothetical protein FLJ22965
RGD1564791	ENSRNOT00000042424	1.31	3.9	16.2	turq	10798896	similar to hypothetical protein 4930474N05
Rwdd2b	NM_001100559	0.82	-11.7	8.9	turq	10752897	RWD domain containing 2B
Spetex-2E	NM_001011702	1.83	23.9	24.9	turq	10779309	Spetex-2E protein
Thumpd3	NM_001170546	0.75	-33.0	32.8	turq	10857667	THUMP domain containing 3
Tm4sf19	NM_001105873	0.80	-4.1	9.7	turq	10754831	transmembrane 4 L six family member 19
Tm9sf3	ENSRNOT00000018043	0.78	-127	48.2	turq	10730173	transmembrane 9 superfamily member 3
Tmem117	ENSRNOT00000008159	0.77	-21.1	27.3	turq	10898879	transmembrane protein 117
**Tmem135**	**NM_001013896**	**0.82**	**-33.9**	**49.2**	**turq**	**10723560**	**transmembrane protein 135**
Tmem136	NM_001108136	0.82	-5.2	8.5	turq	10916640	transmembrane protein 136
Tmem158	NM_057212	0.79	-12.7	30.0	turq	10921117	transmembrane protein 158
Tmem167b	NM_001135260	0.81	-63.0	19.4	turq	10832915	transmembrane protein 167B
Tmem179b	NM_001109572	0.83	-12.4	11.9	turq	10728519	transmembrane protein 179B
Tmem47	NM_001109317	0.76	-184	40.1	turq	10933809	transmembrane protein 47
Tmem55a	NM_001024900	0.82	-33.8	34.6	turq	10867714	transmembrane protein 55A
**Tmem70**	**NM_001109258**	**0.80**	**-28.8**	**67.2**	**turq**	**10867095**	**transmembrane protein 70**
Tmem72	NM_001108643	1.22	2.5	8.1	turq	10864887	transmembrane protein 72
Tmem87a	ENSRNOT00000033788	0.82	-33.6	23.0	turq	10848829	transmembrane protein 87A
Tmem9b	NM_001106289	0.79	-68.0	46.6	turq	10724778	TMEM9 domain family, member B
**Tmtc2**	**XM_001080732**	**0.68**	**-40.2**	**54.0**	**turq**	**10902080**	**transmembrane and tetratricopeptide repeat containing 2**
Ttc9c	NM_001007693	0.82	-41.0	34.3	turq	10728549	tetratricopeptide repeat domain 9C
**Wdr44**	**ENSRNOT00000040742**	**0.78**	**-27.9**	**50.1**	**turq**	**10936437**	**WD repeat domain 44**
**ESTs**							
	**ENSRNOT00000053975**	**1.37**	**49.1**	**68.8**	**turq**	**10798467**	
**RGD1562161**	**BC166438**	**0.82**	**-8.7**	**63.6**	**turq**	**10937989**	**similar to chromosome X open reading frame 23**
**LOC293103**	**NM_001013897**	**0.77**	**-44.1**	**63.3**	**turq**	**10723601**	**similar to RIKEN cDNA 0610007P06**
**LOC290577**	**XM_002725158**	**0.74**	**-7.8**	**62.0**	**turq**	**10786993**	**hypothetical LOC290577**
	**BC104707**	**0.74**	**-39.0**	**58.6**	**turq**	**10812903**	
**RGD1562987**	**NM_001173472**	**0.79**	**-135**	**57.3**	**turq**	**10802712**	**similar to cDNA sequence BC031181**
	**ENSRNOT00000054573**	**1.58**	**51.8**	**56.6**	**turq**	**10860325**	
	**---**	**0.63**	**-83.9**	**55.3**	**turq**	**10856922**	
	**ENSRNOT00000063607**	**1.27**	**4.3**	**54.9**	**turq**	**10867526**	
	**ENSRNOT00000058224**	**1.23**	**2.9**	**53.0**	**turq**	**10895630**	
	**---**	**1.94**	**86.6**	**52.7**	**turq**	**10821115**	
	**ENSRNOT00000053805**	**1.38**	**57.8**	**52.4**	**turq**	**10714346**	
	**ENSRNOT00000053805**	**1.38**	**57.8**	**52.4**	**turq**	**10813854**	
	**ENSRNOT00000053805**	**1.38**	**57.8**	**52.4**	**turq**	**10905668**	
	**ENSRNOT00000053805**	**1.38**	**57.8**	**52.4**	**turq**	**10921286**	
	**---**	**0.64**	**-49.7**	**50.5**	**turq**	**10903674**	
	**---**	**1.20**	**21.4**	**49.1**	**turq**	**10805324**	
**RGD1306613**	**NM_001107356**	**0.80**	**-30.8**	**48.9**	**turq**	**10861026**	**similar to RIKEN cDNA 1600012F09**
	**ENSRNOT00000013315**	**0.82**	**-42.9**	**48.8**	**turq**	**10903941**	
**RGD1306936**	**NM_001106593**	**0.78**	**-24.7**	**48.6**	**turq**	**10855462**	**similar to chromosome 7 open reading frame 30**
LOC498750	BC089212	0.82	-82.4	47.7	turq	10856187	similar to cDNA sequence BC005537
LOC100125371	ENSRNOT00000057627	0.83	-65.9	46.6	turq	10777992	hypothetical LOC100125371
	ENSRNOT00000032075	1.20	2.6	46.5	turq	10744805	
	ENSRNOT00000027351	0.81	-76.4	43.3	turq	10730610	
	ENSRNOT00000032933	0.77	-19.9	42.8	turq	10705422	
	ENSRNOT00000014756	1.28	2.2	42.8	turq	10922255	
	ENSRNOT00000060749	1.51	13.1	40.9	turq	10908655	
RGD1565430	ENSRNOT00000041350	1.23	2.5	40.6	turq	10939741	similar to RIKEN cDNA 1700001F22
	ENSRNOT00000053360	1.59	16.8	40.5	turq	10887030	
	BC087666	0.73	-82.2	40.5	turq	10891594	
	---	1.35	10.3	40.5	turq	10901954	
	BC168232	0.70	-21.6	40.3	turq	10881474	
RGD1307279	NM_001008343	0.83	-14.5	40.3	turq	10805571	similar to RIKEN cDNA 2700002I20
	ENSRNOT00000054035	1.21	4.0	38.1	turq	10768996	
	ENSRNOT00000041882	1.75	137	36.6	turq	10791650	
RGD1566225	ENSRNOT00000049312	1.33	4.2	36.4	turq	10935409	similar to RIKEN cDNA 1700001F22
	ENSRNOT00000010523	1.24	2.0	36.4	turq	10853391	
	---	1.31	6.4	36.4	turq	10909581	
LOC501738	NM_001167664	1.22	2.2	35.8	turq	10739463	hypothetical LOC501738

	ENSRNOT00000053517	1.57	25.9	35.6	turq	10887034	
	ENSRNOT00000053290	1.36	19.2	34.7	turq	10778375	
	ENSRNOT00000004252	0.82	-20.0	33.9	turq	10935021	
	---	0.77	-33.8	33.5	turq	10772272	
	---	0.72	-260	33.4	turq	10850541	
	ENSRNOT00000056070	1.42	15.7	33.4	turq	10905664	
	ENSRNOT00000043281	0.69	-65.7	33.3	turq	10860179	
	ENSRNOT00000040233	1.41	59.0	33.2	turq	10829660	
	ENSRNOT00000055652	1.26	2.9	32.7	turq	10859436	
	ENSRNOT00000053495	1.27	4.4	32.6	turq	10934005	
	ENSRNOT00000063699	1.21	5.7	32.4	turq	10728572	
	ENSRNOT00000053556	1.22	5.3	32.0	turq	10864523	
	ENSRNOT00000018425	0.83	-26.2	31.8	turq	10855765	
	ENSRNOT00000063576	1.52	193	31.4	turq	10891487	
	ENSRNOT00000058158	1.33	15.8	30.9	turq	10805597	
	---	1.22	2.4	30.9	turq	10730587	
	---	1.23	3.1	30.7	turq	10857182	
RGD1560585	ENSRNOT00000056597	1.20	2.1	30.6	turq	10939906	similar to RIKEN cDNA 1700001F22
	ENSRNOT00000052487	1.43	12.0	30.5	turq	10827448	
	---	1.24	11.9	30.1	turq	10726754	
	ENSRNOT00000056931	0.80	-22.9	30.0	turq	10935229	
RGD1564736	ENSRNOT00000056775	1.35	5.3	29.9	turq	10935428	similar to RIKEN cDNA 1700001F22
	---	1.63	44.8	29.7	turq	10777420	
RGD1309676	BC079275	0.82	-29.5	29.1	turq	10790640	similar to RIKEN cDNA 5730469M10
	ENSRNOT00000047224	0.83	-70.7	28.9	turq	10702330	
	ENSRNOT00000011205	0.78	-16.5	28.0	turq	10894523	
LOC311134	ENSRNOT00000037015	0.78	-9.2	27.5	turq	10846604	hypothetical LOC311134
RGD1311406	ENSRNOT00000065673	1.26	3.5	27.5	turq	10921397	similar to RIKEN cDNA B430306N03 gene
	ENSRNOT00000052698	1.40	9.1	27.1	turq	10731138	
	rno-mir-423	1.25	4.1	26.9	turq	10736484	
	ENSRNOT00000057365	1.65	80.2	26.9	turq	10924441	
	ENSRNOT00000053105	1.27	9.8	26.9	turq	10881643	
	---	1.40	12.5	26.0	turq	10735889	
	---	0.64	-85.6	25.7	turq	10723297	
RGD1309308	NM_001126279	0.80	-30.7	25.5	turq	10875795	similar to RIKEN cDNA 1810074P20
	---	1.35	12.7	25.5	turq	10911811	
	---	0.57	-315	25.3	turq	10805603	
	ENSRNOT00000005413	1.28	6.4	25.3	turq	10895439	
	ENSRNOT00000058202	1.23	3.0	25.2	turq	10895665	
	ENSRNOT00000052886	1.22	3.9	24.9	turq	10791563	
	ENSRNOT00000033462	1.22	3.1	24.6	turq	10793335	
	---	1.24	8.3	24.2	turq	10809368	
	ENSRNOT00000063789	1.25	4.9	24.1	turq	10892616	
	ENSRNOT00000059556	1.20	2.9	24.0	turq	10922779	
	---	0.73	-17.8	24.0	turq	10772697	
	ENSRNOT00000050523	1.26	7.3	23.7	turq	10703461	
	ENSRNOT00000047410	1.33	6.3	23.4	turq	10901605	
	---	1.43	7.5	22.7	turq	10932908	
	rno-mir-34c	1.23	4.8	22.3	turq	10917337	
	ENSRNOT00000053933	1.22	3.9	22.3	turq	10830079	
	ENSRNOT00000052441	1.27	11.6	21.8	turq	10841716	
	ENSRNOT00000061147	1.26	3.1	21.7	turq	10702327	
RGD1308147	BC088171	0.79	-14.5	21.6	turq	10798724	similar to expressed sequence AW209491
	ENSRNOT00000058182	1.24	2.9	21.3	turq	10895683	
	ENSRNOT00000046253	1.21	4.2	20.1	turq	10795984	
	ENSRNOT00000045406	0.73	-17.1	20.0	turq	10881451	
	ENSRNOT00000052821	1.33	7.9	20.0	turq	10767278	
	ENSRNOT00000044930	1.29	44.9	19.9	turq	10798463	
	ENSRNOT00000058227	1.31	109	19.8	turq	10846255	
	ENSRNOT00000053907	1.38	9.6	19.6	turq	10746789	
RGD1308114	BC079348	0.78	-9.8	19.3	turq	10795444	similar to cDNA sequence AF397014
	ENSRNOT00000053228	1.26	4.5	18.8	turq	10935473	
	---	0.71	-22.4	18.8	turq	10920743	
	ENSRNOT00000060585	1.21	3.4	18.7	turq	10922179	
	ENSRNOT00000054622	1.48	9.0	18.5	turq	10898938	
LOC100151767	NM_001127503	0.75	-14.1	18.4	turq	10917240	hypothetical LOC100151767
	ENSRNOT00000055439	1.27	5.9	17.9	turq	10867018	
	ENSRNOT00000053474	1.21	2.3	17.8	turq	10776289	
	---	0.75	-17.9	17.7	turq	10722718	

	ENSRNOT00000033914	1.23	5.4	17.7	turq	10856191	
	---	1.25	4.3	17.7	turq	10901534	
	---	1.27	3.9	16.8	turq	10755550	
	ENSRNOT00000054041	1.20	3.5	16.3	turq	10820151	
	rno-mir-487b	1.35	6.3	16.1	turq	10887084	
	ENSRNOT00000018310	0.82	-26.3	15.7	turq	10794691	
	ENSRNOT00000009998	0.76	-16.1	15.6	turq	10859382	
	---	1.32	6.7	15.5	turq	10902859	
	ENSRNOT00000032578	1.23	4.4	15.1	turq	10918485	
	---	1.50	15.4	15.0	turq	10864479	
RGD1564295	ENSRNOT00000056784	1.23	2.8	15.0	turq	10939527	similar to RIKEN cDNA 1700001F22
	---	1.24	4.6	14.8	turq	10898887	
	ENSRNOT00000063690	1.23	2.0	14.7	turq	10756366	
	ENSRNOT00000053478	1.34	5.7	14.7	turq	10846650	
	ENSRNOT00000005665	0.72	-38.7	14.0	turq	10748601	
	ENSRNOT00000046064	1.34	6.7	13.8	turq	10909863	
	ENSRNOT00000058481	1.28	6.4	13.2	turq	10862873	
	ENSRNOT00000000455	1.24	6.3	13.1	turq	10830126	
	ENSRNOT00000041305	1.66	24.7	13.0	turq	10744937	
	ENSRNOT00000044873	1.23	5.7	12.9	turq	10808012	
	ENSRNOT00000054186	1.45	8.9	12.6	turq	10887022	
	ENSRNOT00000053200	1.26	3.8	12.6	turq	10850438	
	---	0.80	-8.5	12.5	turq	10755067	
	---	1.23	2.2	12.2	turq	10774341	
	ENSRNOT00000024998	1.21	2.7	12.2	turq	10709364	
	---	1.59	40.6	11.8	turq	10776216	
	ENSRNOT00000053941	1.22	2.5	11.0	turq	10845397	
	ENSRNOT00000007475	1.35	3.0	10.9	turq	10864300	
	---	1.36	7.6	10.8	turq	10877567	
	ENSRNOT00000010681	0.82	-23.6	10.7	turq	10869772	
	ENSRNOT00000059478	1.27	17.3	10.6	turq	10927668	
	ENSRNOT00000051327	1.27	3.7	10.5	turq	10890904	
	ENSRNOT00000053171	1.28	6.4	10.2	turq	10820133	
	ENSRNOT00000011342	0.79	-8.4	10.2	turq	10869664	
	ENSRNOT00000036594	1.24	2.8	9.4	turq	10885604	
	ENSRNOT00000055266	0.81	-12.1	9.0	turq	10819852	
	ENSRNOT00000061636	1.22	3.2	8.8	turq	10731413	
	ENSRNOT00000052448	1.44	14.6	8.6	turq	10899123	
	---	1.62	23.9	7.5	turq	10939699	
	ENSRNOT00000036063	0.79	-14.2	6.8	turq	10923727	
	ENSRNOT00000054563	1.37	306	6.6	turq	10866957	
	ENSRNOT00000039319	1.22	3.2	6.5	turq	10771889	
	ENSRNOT00000052060	1.29	4.8	6.2	turq	10783533	
RGD1306739	NM_001134576	0.83	-7.8	5.9	turq	10829771	similar to RIKEN cDNA 1700040L02
	ENSRNOT00000053512	1.21	6.5	5.3	turq	10859302	
	ENSRNOT00000053547	1.39	15.1	5.1	turq	10772638	
	GENSCAN00000010217	1.23	2.2	5.0	turq	10767032	
	---	1.21	3.3	4.1	turq	10886210	
	ENSRNOT00000053840	1.49	36.5	3.9	turq	10864773	
	ENSRNOT00000032427	1.20	3.0	3.2	turq	10829618	
	ENSRNOT00000031391	0.83	-2.3	2.2	turq	10914454	
	ENSRNOT00000053488	1.29	5.9	2.2	turq	10703398	
	U15425	0.75	-3.7	1.1	turq	10881301	
	ENSRNOT00000053376	1.22	8.5	0.0	grey	10736240	

*** - genes belonning to top 10% of each module withing each brain region are marked by bold font**

**- Modules and k.in are given for seaparate networks; abbreviations used for modules: *turq* - turquoise, *ylw* -yellow, *brwn* - brown

Table S2-D. Differentially Expressed Genes in Female Rat Enterorhinal Cortex (384 genes and ESTs)

Gene Symbol	GenBank, Ref.Sequence	Vin/Con Ratio	Vin-Con mean_dif	k.in **	Module**	ProbeSet ID	Gene Title
**Apoptosis**							
LOC689416	XM_001070715	0.81	-2.5	15.3	turq	10824810	similar to TD and POZ domain containing 2
Perp	NM_001106265	0.70	-12.8	5.5	turq	10701913	PERP, TP53 apoptosis effector
Tifa	NM_001014044	1.20	5.9	1.5	turq	10818983	TRAF-interacting protein with forkhead-associated domain
Tnfrsf1a	NM_013091	1.22	5.4	3.7	turq	10858967	tumor necrosis factor receptor superfamily, member 1a
**Cell Cycle**							
Cdc26	NM_001013240	1.24	29.8	16.9	turq	10877411	cell division cycle 26

Cenpk	NM_001106407	1.32	8.4	21.6	turq	10812823	centromere protein K
Cspp1	ENSRNOT00000008480	1.25	42.7	8.3	turq	10875087	centrosome and spindle pole associated protein 1
G2e3	NM_001106726	1.29	15.6	15.4	turq	10884489	G2/M-phase specific E3 ubiquitin ligase
Pelo	NM_001007634	1.32	9.9	9.3	turq	10821402	pelota homolog (Drosophila)
**Pot1**	**BC085834**	**1.34**	**14.2**	**24.1**	**turq**	**10861361**	**protection of telomeres 1A**
**Cytoskeleton-ECM**							
Cdh20	NM_001012748	1.55	35.4	18.2	turq	10763225	cadherin 20
Flrt2	NM_001106750	1.34	14.4	12.8	turq	10886269	fibronectin leucine rich transmembrane protein 2
Gpc3	NM_012774	0.71	-14.1	3.9	turq	10939764	glypican 3
Kif26b	NM_001109079	0.82	-4.7	6.0	turq	10766082	kinesin family member 26B
Map1lc3a	NM_199500	1.24	16.7	13.9	turq	10841333	microtubule-associated protein 1 light chain 3 alpha
Nck2	NM_001108216	1.22	17.9	9.3	turq	10922956	NCK adaptor protein 2
Pcdh19	NM_001169129	0.70	-44.6	14.6	turq	10939226	protocadherin 19
Pcdhb8	NM_001014779	1.21	10.5	8.1	turq	10801180	protocadherin beta 8
RGD1559781	ENSRNOT00000000807	0.78	-4.1	12.3	turq	10829824	similar to Dynein, axonemal, light chain 4
Serinc2	NM_001031656	0.75	-6.5	3.9	turq	10880095	serine incorporator 2
Ska2	NM_001009624	1.20	6.2	0.0	grey	10711496	spindle and kinetochore associated complex subunit 2
Ska2	NM_001009624	1.21	7.6	0.0	grey	10737129	spindle and kinetochore associated complex subunit 2
Thsd7b	ENSRNOT00000005212	1.29	11.5	14.7	turq	10763704	thrombospondin, type I, domain containing 7B
**Development**							
**Cbln1**	**NM_001109127**	**1.55**	**16.4**	**23.7**	**turq**	**10806351**	**cerebellin 1 precursor**
**Cln5**	**ENSRNOT00000013381**	**1.22**	**6.3**	**22.0**	**turq**	**10781882**	**ceroid-lipofuscinosis, neuronal 5**
Cpne5	NM_001107616	1.20	31.2	9.3	turq	10832034	copine V
Ctns	ENSRNOT00000030343	1.20	10.7	5.9	turq	10744759	cystinosis, nephropathic
Cux1	ENSRNOT00000001928	1.21	12.6	19.1	turq	10757507	cut-like homeobox 1
Cux2	NM_001105931	1.37	13.1	11.1	turq	10758624	cut-like homeobox 2
Dcaf17	NM_001177688	1.20	4.5	8.7	turq	10836769	DDB1 and CUL4 associated factor 17
Eed	BC168872	0.83	-26.6	2.3	turq	10723623	embryonic ectoderm development
Lgi2	NM_001107219	1.27	20.4	19.3	turq	10772939	leucine-rich repeat LGI family, member 2
Midn	ENSRNOT00000060698	1.24	19.8	13.8	turq	10900592	midnolin
Nedd8	NM_138878	1.21	36.0	10.1	turq	10783863	neural precursor cell expressed, developmentally down-regulated 8
Ntn3	NM_053732	0.82	-3.5	14.5	turq	10741009	netrin 3
**Odz3**	**NM_001169133**	**1.36**	**32.5**	**23.9**	**turq**	**10788086**	**odz, odd Oz/ten-m homolog 3 (Drosophila)**
Olfm2	NM_001015017	1.38	12.8	7.5	turq	10915345	olfactomedin 2
**Sema3a**	**NM_017310**	**1.66**	**45.9**	**23.7**	**turq**	**10860481**	**sema domain, immunoglobulin domain (Ig), short basic domain, secreted, (semaphorin) 3A**
Sema3b	NM_001079942	1.34	13.7	20.7	turq	10920089	sema domain, immunoglobulin domain (Ig), short basic domain, secreted, (semaphorin) 3B
Shisa6	ENSRNOT00000057509	0.70	-21.4	12.3	turq	10743715	shisa homolog 6 (Xenopus laevis)
Sipa1l3	ENSRNOT00000038929	0.75	-9.1	11.7	turq	10720510	signal-induced proliferation-associated 1 like 3
Slit2	NM_022632	0.79	-15.6	8.5	turq	10777137	slit homolog 2 (Drosophila)
Slit3	NM_031321	0.81	-17.5	14.6	turq	10732750	slit homolog 3 (Drosophila)
Svs5	NM_133516	0.80	-2.8	21.2	turq	10842098	seminal vesicle secretory protein 5
Tex264	NM_001007665	1.24	33.3	18.3	turq	10919960	testis expressed 264
Ulk2	ENSRNOT00000003792	1.23	41.2	18.4	turq	10743482	Unc-51 like kinase 2 (C. elegans)
Wbscr17	NM_001025112	0.79	-15.7	8.0	turq	10757940	Williams-Beuren syndrome chromosome region 17 homolog (human)
Wbscr27	NM_001109499	1.26	8.5	6.6	turq	10768112	Williams Beuren syndrome chromosome region 27
Asap1	NM_001044245	1.34	84.0	8.6	turq	10904031	ArfGAP with SH3 domain, ankyrin repeat and PH domain 1
**DNA Repair**							
Pms2	NM_001105908	1.26	16.4	16.3	turq	10760343	PMS2 postmeiotic segregation increased 2 (S. cerevisiae)
**Electron Transport**							
Ucp2	NM_019354	1.23	17.6	4.9	turq	10709093	uncoupling protein 2 (mitochondrial, proton carrier)
**Epigenetics**							
Alkbh8	ENSRNOT00000003432	1.27	24.2	17.1	turq	10892898	alkB, alkylation repair homolog 8 (E. coli)
Coq5	NM_001039022	1.22	20.7	11.5	turq	10762709	coenzyme Q5 homolog, methyltransferase (S. cerevisiae)
**Mettl14**	**NM_001106470**	**1.24**	**22.9**	**24.5**	**turq**	**10826547**	**methyltransferase like 14**
Pgbd5	NM_001106198	1.33	13.1	13.0	turq	10811896	piggyBac transposable element derived 5
Phf5a	NM_138888	1.22	16.1	16.1	turq	10905651	PHD finger protein 5A
Satb1	NM_001012129	1.47	65.4	14.1	turq	10921274	SATB homeobox 1
Scmh1	NM_001109669	1.26	19.7	4.2	turq	10871629	sex comb on midleg homolog 1 (Drosophila)
**Golgi Apparatus**							
Hs6st2	ENSRNOT00000040165	1.23	19.6	9.0	turq	10939725	heparan sulfate 6-O-sulfotransferase 2
**Gnpda2**	**NM_001106005**	**1.24**	**13.5**	**24.5**	**turq**	**10772556**	**glucosamine-6-phosphate deaminase 2**
**Growth Factors**							
Fam19a2	ENSRNOT00000005537	1.31	24.9	12.0	turq	10895766	family with sequence similarity 19 (chemokine (C-C motif)-like), member A2
Fgf22	NM_130751	0.74	-8.8	17.3	turq	10900789	fibroblast growth factor 22
Gdf10	NM_024375	0.78	-4.9	15.2	turq	10786905	growth differentiation factor 10
Grb14	NM_031623	1.25	21.5	8.6	turq	10845751	growth factor receptor bound protein 14
Gria1	NM_031608	0.69	-377	5.9	turq	10733723	glutamate receptor, ionotropic, AMPA 1
Il16	NM_001105749	0.77	-5.5	20.6	turq	10723351	interleukin 16
**Il34**	**NM_001025766**	**1.22**	**26.4**	**24.1**	**turq**	**10810976**	**interleukin 34**

Mdk	NM_030859	1.25	15.4	13.8	turq	10847432	midkine
RGD1307225	NM_001107663	1.69	15.6	18.0	turq	10814545	similar to MEGF6 <multiple EGF-like-domains 6>
RGD1307225	NM_001107663	1.39	61.7	21.7	turq	10814540	similar to MEGF6 <multiple EGF-like-domains 6>
Ctgf	NM_022266	1.24	8.3	4.3	turq	10717233	connective tissue growth factor
**Immune Response**							
C1ql2	NM_001105949	0.64	-18.5	10.5	turq	10931669	complement component 1, q subcomponent-like 2
C1ql3	NM_001109403	0.71	-65.1	2.7	turq	10799733	complement component 1, q subcomponent-like 3
Cd180	NM_001106405	0.83	-5.1	4.3	turq	10812775	CD180 molecule
Clk4	NM_001013041	1.27	41.4	8.1	turq	10733321	CDC like kinase 4
Ctage5	NM_001106734	1.27	18.6	7.3	turq	10884772	CTAGE family, member 5 <Cutaneous T-cell lymphoma-associated antigen 5>
**F3**	**NM_013057**	**1.32**	**27.2**	**27.3**	**turq**	**10818708**	**coagulation factor III (thromboplastin, tissue factor)**
Ica1l	NM_199400	1.30	11.0	15.0	turq	10928433	islet cell autoantigen 1-like
**Ighg**	**BC095846**	**0.75**	**-5.1**	**24.5**	**turq**	**10892509**	**Immunoglobulin heavy chain (gamma polypeptide)**
Ighmbp2	NM_031586	0.82	-15.5	8.9	turq	10727303	immunoglobulin mu binding protein 2
RT1-CE11	NM_001008834	0.77	-5.6	0.0	grey	10833939	RT1 class I, locus CE11
Slpi	NM_053372	0.78	-5.7	2.8	turq	10851581	secretory leukocyte peptidase inhibitor
Xkr8	NM_001012099	0.77	-5.4	10.1	turq	10872681	XK, Kell blood group complex subunit-related family, member 8
**Xkr8**	**NM_001012099**	**0.76**	**-7.2**	**22.5**	**turq**	**10872686**	**XK, Kell blood group complex subunit-related family, member 8**
**Metabolism & Transport**							
Ptges3l1	BC087125	1.26	3.8	0.0	grey	10938126	prostaglandin E synthase 3-like 1
Aadat	NM_017193	1.23	16.9	8.7	turq	10791478	aminoadipate aminotransferase
Acaa2	NM_130433	1.35	28.0	8.7	turq	10802691	acetyl-Coenzyme A acyltransferase 2
Ace	NM_012544	1.22	4.3	6.0	turq	10739035	angiotensin I converting enzyme (peptidyl-dipeptidase A) 1
Apeh	NM_012500	1.25	15.3	7.9	turq	10920261	N-acylaminoacyl-peptide hydrolase
Atox1	NM_053359	1.30	17.6	8.4	turq	10742813	ATX1 antioxidant protein 1 homolog (yeast)
Bdh2	NM_001106473	1.31	4.2	19.8	turq	10819192	3-hydroxybutyrate dehydrogenase, type 2
Blvra	NM_053850	1.21	7.2	3.4	turq	10813126	biliverdin reductase A
Cacnb4	NM_001105733	1.35	82.7	16.6	turq	10845306	calcium channel, voltage-dependent, beta 4 subunit
Car12	NM_001080756	0.58	-70.7	18.2	turq	10911145	carbonic anyhydrase 12
**Colec10**	**NM_001130541**	**0.81**	**-2.3**	**30.6**	**turq**	**10896526**	**collectin sub-family member 10**
Coq10b	NM_001009671	1.23	17.8	17.7	turq	10923338	coenzyme Q10 homolog B (S. cerevisiae)
Cplx3	NM_001109295	1.36	5.4	4.3	turq	10917776	complexin 3
**Dut**	**NM_053592**	**1.23**	**20.2**	**24.1**	**turq**	**10717992**	**deoxyuridine triphosphatase**
Fuca2	NM_001004218	1.21	22.5	16.6	turq	10701817	fucosidase, alpha-L- 2, plasma
Fut9	NM_053465	1.37	83.0	13.1	turq	10875817	fucosyltransferase 9 (alpha (1,3) fucosyltransferase)
Fxyd6	NM_022005	1.26	67.5	13.7	turq	10909639	FXYD domain-containing ion transport regulator 6
Galt	NM_001013089	1.23	6.3	8.5	turq	10868384	galactose-1-phosphate uridylyltransferase
Gsta4	NM_001106840	1.30	11.1	8.8	turq	10911797	glutathione S-transferase alpha 4
Hbq1	ENSRNOT00000067457	0.79	-2.6	17.9	turq	10741770	hemoglobin, theta 1
Kcnb2	NM_054000	1.22	14.6	8.0	turq	10874952	potassium voltage gated channel, Shab-related subfamily, member 2
Kcng3	NM_133426	1.23	6.0	17.5	turq	10888131	potassium voltage-gated channel, subfamily G, member 3
Kcnj2	NM_017296	1.21	10.3	2.6	turq	10739353	potassium inwardly-rectifying channel, subfamily J, member 2
Kcnq2	NM_133322	0.82	-61.5	18.4	turq	10852437	potassium voltage-gated channel, KQT-like subfamily, member 2
Kctd6	NM_001107253	0.81	-12.8	7.9	turq	10782658	potassium channel tetramerisation domain containing 6
Kctd9	NM_001108871	1.25	11.7	7.0	turq	10781243	potassium channel tetramerisation domain containing 9
Klk1b21	NM_001013067	0.82	-2.2	9.1	turq	10706468	kallikrein 1-related peptidase b21
Klk8	NM_001107509	0.79	-4.4	11.1	turq	10706424	kallikrein related-peptidase 8
LOC685173	XM_001062675	0.81	-2.3	21.2	turq	10899693	similar to carbonic anhydrase 13
**Mat2b**	**NM_001044282**	**1.25**	**86.5**	**24.0**	**turq**	**10742121**	**methionine adenosyltransferase II, beta**
Mocs2	NM_001162413	1.21	53.2	14.2	turq	10813136	molybdenum cofactor synthesis 2
**Moxd1**	**ENSRNOT00000061234**	**1.20**	**29.5**	**28.2**	**turq**	**10717240**	**monooxygenase, DBH-like 1**
**Mppe1**	**NM_001108435**	**1.25**	**5.0**	**25.4**	**turq**	**10804964**	**metallophosphoesterase 1**
**Nat9**	**NM_001134835**	**1.25**	**10.5**	**27.5**	**turq**	**10748882**	**N-acetyltransferase 9 (GCN5-related, putative)**
Ndufv3	NM_022607	1.28	13.2	12.9	turq	10829158	NADH dehydrogenase (ubiquinone) flavoprotein 3
Nos2	NM_012611	0.81	-3.0	8.6	turq	10736312	nitric oxide synthase 2, inducible
Pgcp	NM_031640	1.23	13.9	5.1	turq	10896028	plasma glutamate carboxypeptidase
Pgm2	NM_001106007	1.23	15.9	9.4	turq	10776938	phosphoglucomutase 2
Plcd1	NM_017035	1.26	9.4	11.3	turq	10920803	phospholipase C, delta 1
Plcl1	NM_053456	1.23	20.4	9.4	turq	10923361	phospholipase C-like 1
Pld5	ENSRNOT00000005332	1.25	11.4	5.0	turq	10770161	phospholipase D family, member 5
**Pmm1**	**NM_001008323**	**1.20**	**89.2**	**23.8**	**turq**	**10904002**	**phosphomannomutase 1**
Ppapdc1a	ENSRNOT00000027681	0.72	-29.4	6.4	turq	10711454	phosphatidic acid phosphatase type 2 domain containing 1A
**Ppapdc2**	**NM_001034854**	**1.30**	**44.4**	**24.4**	**turq**	**10714634**	**phosphatidic acid phosphatase type 2 domain containing 2**
Pyroxd2	NM_001004261	1.36	8.0	12.8	turq	10715400	pyridine nucleotide-disulphide oxidoreductase domain 2
Qpctl	NM_001106230	1.24	16.4	14.6	turq	10719386	glutaminyl-peptide cyclotransferase-like
Rdh10	NM_181478	1.25	23.7	11.8	turq	10874929	retinol dehydrogenase 10 (all-trans)
RGD1565002	ENSRNOT00000007645	1.30	61.6	17.1	turq	10890537	similar to Dehydrogenase/reductase SDR family member 7 precursor (Retinal short-chain dehy
RGD1565338	ENSRNOT00000006775	0.83	-8.0	5.7	turq	10884898	similar to voltage-dependent anion channel 1
Slc24a4	NM_001108051	0.81	-15.4	7.4	turq	10886429	solute carrier family 24 (sodium

Slc25a32	NM_001173334	1.25	16.7	6.9	turq	10903482	solute carrier family 25, member 32
Slc35f4	ENSRNOT00000020175	1.45	24.4	10.1	turq	10783011	solute carrier family 35, member F4
Smpdl3b	NM_001025737	0.78	-11.4	21.5	turq	10872671	sphingomyelin phosphodiesterase, acid-like 3B
Sod1	NM_017050	1.24	89.0	15.0	turq	10753005	superoxide dismutase 1, soluble
Sord	NM_017052	1.21	36.1	14.3	turq	10839254	sorbitol dehydrogenase
**Thnsl1**	**NM_001025035**	**1.30**	**11.8**	**23.9**	**turq**	**10796696**	**threonine synthase-like 1 (S. cerevisiae)**
Tmed10	NM_053467	1.22	63.6	17.3	turq	10886029	transmembrane emp24-like trafficking protein 10 (yeast)
Tomm7	NM_001135174	1.23	113	7.6	turq	10852964	translocase of outer mitochondrial membrane 7 homolog (yeast)
Uap1	ENSRNOT00000058560	1.28	11.7	9.3	turq	10882882	UDP-N-acteylglucosamine pyrophosphorylase 1
Xylb	NM_001033704	0.82	-4.1	6.2	turq	10914209	xylulokinase homolog (H. influenzae)
**Proteolysis**							
Cpm	NM_001108098	1.25	9.9	19.6	turq	10895589	carboxypeptidase M
Gzmc	NM_134332	0.69	-4.6	20.5	turq	10784059	granzyme C
Hecw2	NM_001108218	1.23	42.7	16.5	turq	10928001	HECT, C2 and WW domain containing E3 ubiquitin protein ligase 2
LOC494529	NM_001009974	1.38	13.7	13.2	turq	10906037	92Aa-Protein
Rnf103	NM_053438	1.27	21.4	6.9	turq	10856282	ring finger protein 103
Rnf112	NM_138613	0.81	-22.9	12.0	turq	10743409	ring finger protein 112
Senp17	NM_001002833	0.77	-3.0	8.2	turq	10865813	Sumo1/sentrin/SMT3 specific peptidase 17
Ube2q2l	NM_001135997	1.22	3.5	13.5	turq	10936821	ubiquitin-conjugating enzyme E2Q family member 2-like
Ubxn2a	NM_001109482	1.26	21.3	21.2	turq	10889148	UBX domain protein 2A
Ubxn2a	NM_001109482	1.25	21.7	12.9	turq	10939465	UBX domain protein 2A
Ubxn2a	NM_001109482	1.27	23.3	6.3	turq	10830696	UBX domain protein 2A
**Receptors & Binding Proteins**							
Chrna7	NM_012832	0.79	-26.6	11.7	turq	10722532	cholinergic receptor, nicotinic, alpha 7
Dear	ENSRNOT00000042146	1.20	73.4	11.0	turq	10816160	dual endothelin 1, angiotensin II receptor
Epha3	NM_031564	1.33	19.9	16.9	turq	10752630	Eph receptor A3
Fzd8	NM_001044251	1.37	17.5	9.7	turq	10795673	frizzled homolog 8 (Drosophila)
Hhip	ENSRNOT00000024616	1.23	6.6	11.0	turq	10806946	Hedgehog-interacting protein
Htr3a	NM_024394	0.79	-4.1	13.1	turq	10917123	5-hydroxytryptamine (serotonin) receptor 3a
**Il1rapl1**	**NM_177935**	**1.38**	**39.7**	**31.1**	**turq**	**10938291**	**interleukin 1 receptor accessory protein-like 1**
**Il4ra**	**NM_133380**	**0.74**	**-19.4**	**27.0**	**turq**	**10710782**	**interleukin 4 receptor, alpha**
Il6ra	NM_017020	0.77	-12.8	18.3	turq	10824530	interleukin 6 receptor, alpha
Nab1	NM_022856	1.23	9.6	11.5	turq	10923198	Ngfi-A binding protein 1
Npy2r	NM_023968	0.57	-18.6	13.9	turq	10815655	neuropeptide Y receptor Y2
Npy2r	NM_023968	0.57	-18.6	13.9	turq	10823937	neuropeptide Y receptor Y2
Olr1330	NM_001000476	0.77	-5.5	10.1	turq	10916410	olfactory receptor 1330
Olr1425	NM_001000010	0.70	-4.7	18.4	turq	10742940	olfactory receptor 1425
Olr1448	NM_001000019	0.63	-31.5	6.0	turq	10733888	olfactory receptor 1448
Olr1458	NM_001000021	0.80	-4.3	12.7	turq	10733906	olfactory receptor 1458
Olr1585	NM_001001109	0.83	-2.4	13.9	turq	10765829	olfactory receptor 1585
Olr1657	NM_001000536	0.80	-3.1	21.2	turq	10798511	olfactory receptor 1657
Olr385	ENSRNOT00000043459	0.80	-2.3	17.3	turq	10716056	olfactory receptor 385
Olr425	NM_001000393	0.81	-4.8	8.4	turq	10844770	olfactory receptor 425
Olr540	NM_001000321	0.83	-3.6	19.8	turq	10847035	olfactory receptor 540
Olr546	NM_001001054	0.82	-3.9	12.9	turq	10847049	olfactory receptor 546
Olr630	NM_001001059	0.82	-2.5	12.7	turq	10847111	olfactory receptor 630
Olr703	NM_001000359	0.82	-2.4	20.6	turq	10847199	olfactory receptor 703
Olr796	NM_001000600	0.77	-5.5	8.8	turq	10838449	olfactory receptor 796
Plxna4a	NM_001107852	0.83	-35.9	5.2	turq	10861678	plexin A4, A
Pvrl1	ENSRNOT00000008614	0.76	-24.7	2.3	turq	10909394	poliovirus receptor-related 1
**Ranbp1**	**NM_001108324**	**1.26**	**102**	**22.4**	**turq**	**10752334**	**RAN binding protein 1**
V1rj2	NM_001008929	0.67	-9.9	4.7	turq	10704297	vomeronasal 1 receptor, J2
Vom2r25	NM_001099482	1.25	2.3	14.2	turq	10703640	vomeronasal 2 receptor, 25
Vom2r80	NM_001111321	0.82	-3.3	4.8	turq	10703589	vomeronasal 2 receptor, 80
**Signaling**							
Agbl1	ENSRNOT00000031334	0.81	-3.2	7.8	turq	10722858	ATP/GTP binding protein-like 1
Calml4	NM_001127575	0.81	-2.8	12.2	turq	10910716	calmodulin-like 4
Dgkg	NM_013126	0.57	-68.4	13.7	turq	10752007	diacylglycerol kinase, gamma
Efhd1	NM_001109310	1.24	30.2	8.7	turq	10925014	EF-hand domain family, member D1
Gpr101	NM_001108258	0.79	-4.9	6.5	turq	10939901	G protein-coupled receptor 101
Gpr61	NM_001107715	1.23	9.0	2.5	turq	10825973	G protein-coupled receptor 61
Gpr83	NM_080411	0.77	-9.4	14.2	turq	10908072	G protein-coupled receptor 83
Gpr88	NM_031696	1.72	22.1	12.0	turq	10826261	G-protein coupled receptor 88
Hipk3	NM_031787	1.25	60.3	16.3	turq	10847903	homeodomain interacting protein kinase 3
**Ksr1**	**NM_001108284**	**0.77**	**-13.5**	**24.0**	**turq**	**10745303**	**kinase suppressor of ras 1**
LOC289641	ENSRNOT00000031638	1.30	5.8	0.0	grey	10772802	similar to Centaurin-delta 1 (Cnt-d1)
Mesp2	NM_001106273	0.82	-4.8	4.0	turq	10708143	mesoderm posterior 2 homolog (mouse)
**Nek4**	**NM_001013134**	**0.82**	**-14.2**	**25.9**	**turq**	**10786563**	**NIMA (never in mitosis gene a)-related kinase 4**
Nod1	NM_001109236	0.80	-4.5	14.7	turq	10862649	nucleotide-binding oligomerization domain containing 1

**Nxn**	**NM_001108285**	**1.41**	**23.0**	**25.5**	**turq**	**10736545**	**nucleoredoxin**
Pak3	NM_019210	0.81	-88.2	15.5	turq	10937362	p21 protein (Cdc42
Pdk4	NM_053551	1.32	10.1	7.9	turq	10860900	pyruvate dehydrogenase kinase, isozyme 4
Pex7	NM_001034147	1.26	8.6	11.3	turq	10717056	peroxisomal biogenesis factor 7
Phospho1	NM_001105833	1.22	8.3	5.1	turq	10737659	phosphatase, orphan 1
Ppm1l	NM_001107681	1.36	52.3	20.0	turq	10815919	protein phosphatase 1 (formerly 2C)-like
**Ptpre**	**NM_053767**	**0.76**	**-23.8**	**21.9**	**turq**	**10711852**	**protein tyrosine phosphatase, receptor type, E**
**Ptpru**	**ENSRNOT00000036148**	**0.67**	**-16.1**	**21.9**	**turq**	**10880172**	**protein tyrosine phosphatase, receptor type, U**
Qrfp	NM_198200	0.82	-4.8	4.6	turq	10844273	pyroglutamylated RFamide peptide
Rerg	ENSRNOT00000030850	0.80	-16.1	13.3	turq	10866535	RAS-like, estrogen-regulated, growth-inhibitor
RGD1306565	ENSRNOT00000051496	1.27	12.7	7.3	turq	10701949	similar to apoptosis signal-regulating kinase 1
RGD1561963	ENSRNOT00000064161	0.75	-47.5	7.7	turq	10929321	similar to Dedicator of cytokinesis protein 10 (Protein zizimin 3)
Rgs4	NM_017214	1.33	125	13.5	turq	10769672	regulator of G-protein signaling 4
Rgs5	NM_019341	1.50	38.4	10.8	turq	10765437	regulator of G-protein signaling 5
Rreb1	NM_001107348	0.79	-6.6	3.2	turq	10798010	ras responsive element binding protein 1
Sel1l3	ENSRNOT00000006610	1.26	14.5	8.4	turq	10772915	sel-1 suppressor of lin-12-like 3 (C. elegans)
Sipa1l3	ENSRNOT00000038929	0.76	-17.2	19.4	turq	10720514	signal-induced proliferation-associated 1 like 3
Smpdl3b	NM_001025737	0.75	-11.9	12.3	turq	10880339	sphingomyelin phosphodiesterase, acid-like 3B
Stk32a	ENSRNOT00000031370	0.78	-5.4	15.8	turq	10801411	serine/threonine kinase 32a
Stk32b	NM_001107224	0.83	-2.3	10.2	turq	10773259	serine/threonine kinase 32b
Stk32b	NM_001107224	0.70	-5.5	9.3	turq	10773255	serine/threonine kinase 32b
Trim31	NM_001106376	0.78	-3.0	8.1	turq	10830912	tripartite motif-containing 31
**Transcription**							
Ccdc34	NM_001108587	1.28	10.8	8.4	turq	10838373	coiled-coil domain containing 34
Ccdc85b	ENSRNOT00000027896	1.30	24.5	1.5	turq	10727854	coiled-coil domain containing 85B
Dnajc2	NM_053776	1.26	18.9	9.3	turq	10853110	DnaJ (Hsp40) homolog, subfamily C, member 2
Fat3	NM_138544	1.41	28.6	18.3	turq	10915131	FAT tumor suppressor homolog 3 (Drosophila)
Fbxl7	NM_001108545	0.83	-2.2	10.4	turq	10822094	F-box and leucine-rich repeat protein 7
Fos	NM_022197	1.44	7.5	21.5	turq	10886031	FBJ osteosarcoma oncogene
Id3	NM_013058	1.32	21.3	4.5	turq	10872972	inhibitor of DNA binding 3
Junb	NM_021836	1.25	13.2	8.2	turq	10806585	jun B proto-oncogene
Kbtbd8	NM_001109250	1.38	9.5	13.7	turq	10857423	kelch repeat and BTB (POZ) domain containing 8
Klhl3	ENSRNOT00000034877	0.82	-7.1	7.0	turq	10793893	kelch-like 3 (Drosophila)
LOC100363994	XM_002725924	1.23	6.7	16.4	turq	10814162	zinc finger protein 455-like
LOC683302	XM_001065309	1.27	20.6	11.3	turq	10787048	similar to tumor protein, translationally-controlled 1
LOC691468	XM_002724499	1.29	15.2	9.9	turq	10733914	similar to Zinc finger protein 84 (Zinc finger protein HPF2)
Mef2c	ENSRNOT00000041296	1.32	142	11.5	turq	10820223	myocyte enhancer factor 2C
Mlh1	NM_031053	1.21	5.5	12.4	turq	10920568	mutL homolog 1 (E. coli)
Myt1	NM_001108615	0.80	-9.5	14.4	turq	10843100	myelin transcription factor 1
Nfe2l2	NM_031789	1.32	38.2	8.2	turq	10846286	nuclear factor, erythroid derived 2, like 2
Noc4l	NM_001014129	0.82	-11.6	8.7	turq	10759342	nucleolar complex associated 4 homolog (S. cerevisiae)
Nr4a1	NM_024388	1.45	42.6	11.8	turq	10899387	nuclear receptor subfamily 4, group A, member 1
Pla2g16	NM_017060	1.28	23.7	8.5	turq	10713538	phospholipase A2, group XVI
Pole3	NM_001007652	1.26	11.1	10.0	turq	10877443	polymerase (DNA directed), epsilon 3 (p17 subunit)
Prox1	NM_001107201	0.69	-12.5	3.0	turq	10770680	prospero homeobox 1
RGD1306520	NM_001127485	1.22	10.8	9.4	turq	10818230	similar to receptor-interacting factor 1
RGD1560095	ENSRNOT00000040584	0.53	-27.6	12.5	turq	10833564	similar to double homeobox, 4
RGD1560095	ENSRNOT00000040584	0.36	-66.5	18.7	turq	10830272	similar to double homeobox, 4
RGD1563091	XM_575365	1.40	23.7	10.1	turq	10860801	similar to OEF2
RGD1565635	ENSRNOT00000021981	1.29	12.7	9.1	turq	10800085	similar to zinc finger protein 124
Rprm	NM_001044276	1.28	10.9	18.7	turq	10845372	reprimo, TP53 dependent G2 arrest mediator candidate
Runx1t1	NM_001108657	1.22	18.2	6.8	turq	10867701	runt-related transcription factor 1; translocated to, 1 (cyclin D-related)
Stx2	NM_012748	0.79	-10.3	7.3	turq	10758050	syntaxin 2
Tbr1	ENSRNOT00000065340	1.37	55.1	18.3	turq	10845628	T-box, brain, 1
Tdpoz1	XM_345239	0.74	-7.7	9.0	turq	10824790	TD and POZ domain containing 1
Zbtb33	NM_001109314	1.28	40.2	7.6	turq	10936263	zinc finger and BTB domain containing 33
Zdhhc21	NM_001039009	1.23	29.2	7.3	turq	10877772	zinc finger, DHHC-type containing 21
Zfp110	NM_001024775	1.25	23.1	14.6	turq	10704223	zinc finger protein 110
Zfp26	NM_001108995	1.21	17.6	5.2	turq	10915316	zinc finger protein 26
Zfp317	NM_001134634	1.21	13.8	10.7	turq	10908182	zinc finger protein 317
Zfp397os	ENSRNOT00000039216	1.26	8.8	16.2	turq	10803488	zinc finger protein 397 opposite strand
**Translation & Protein Modification**							
Ctdsp1	NM_001128079	1.24	11.5	15.9	turq	10924304	CTD (carboxy-terminal domain, RNA polymerase II, polypeptide A) small phosphatase 1
LOC690662	XM_001075132	0.81	-2.1	17.2	turq	10808010	similar to 60S ribosomal protein L29 (P23)
**mrpl11**	**NM_001006973**	**1.22**	**19.3**	**25.8**	**turq**	**10712955**	**mitochondrial ribosomal protein L11**
Mrps28	NM_001047909	1.20	6.9	5.5	turq	10814342	mitochondrial ribosomal protein S28
Ncl	NM_012749	0.74	-58.7	9.8	turq	10779091	nucleolin
RGD1564138	XM_345989	0.75	-8.8	4.4	turq	10913800	similar to 60S ribosomal protein L29 (P23)
RGD1564268	ENSRNOT00000041359	0.79	-6.0	5.1	turq	10904416	similar to ribosomal protein L36

Rnpc3	NM_001100810	1.27	25.4	16.2	turq	10826216	RNA-binding region (RNP1, RRM) containing 3
**Rps2-ps5**	**ENSRNOT00000044113**	**0.79**	**-11.0**	**21.9**	**turq**	**10901611**	**ribosomal protein S2, pseudogene 5**
Rps6	NM_017160	1.22	224	15.2	turq	10877916	ribosomal protein S6
**Miscellaneous & Unknown**							
Krcc1	NM_001009413	1.32	18.9	11.7	turq	10856259	lysine-rich coiled-coil 1
LOC292449	AY389467	0.71	-9.6	1.0	turq	10701663	similar to hypothetical protein
LOC314016	ENSRNOT00000006122	0.82	-3.4	16.3	turq	10884031	similar to mKIAA0230 protein
LOC497934	NM_001017474	0.81	-7.3	10.3	turq	10743853	similar to hypothetical protein FLJ20014
LOC498154	NM_001025033	0.82	-10.3	12.2	turq	10757090	hypothetical protein LOC498154
LOC681849	ENSRNOT00000067627	1.21	44.6	10.0	turq	10918776	similar to Protein C6orf142 homolog
LOC686092	ENSRNOT00000050090	1.20	21.4	5.8	turq	10923334	similar to UPF0197 protein C11orf10 homolog
LOC690728	NM_001109608	1.23	33.5	19.2	turq	10866926	similar to Protein C12orf11 (Sarcoma antigen NY-SAR-95)
LOC691130	ENSRNOT00000065949	0.82	-3.6	6.6	turq	10783406	hypothetical protein LOC691130
**LOC691130**	**ENSRNOT00000060358**	**0.81**	**-2.7**	**30.6**	**turq**	**10783448**	**hypothetical protein LOC691130**
Luc7l3	NM_001108291	1.25	69.0	12.1	turq	10746286	LUC7-like 3 (S. cerevisiae)
Otud1	ENSRNOT00000022734	1.23	7.6	3.8	turq	10796673	OTU domain containing 1
RGD1304810	NM_001107314	1.20	15.3	17.4	turq	10791983	similar to 6430573F11Rik protein
RGD1308616	ENSRNOT00000068128	0.82	-4.0	21.7	turq	10879213	similar to KIAA0467 protein
RGD1309621	ENSRNOT00000055437	1.22	9.7	3.8	turq	10859774	similar to hypothetical protein FLJ10652
RGD1311307	NM_001025719	0.79	-4.6	7.8	turq	10794800	similar to 1300014I06Rik protein
RGD1560883	ENSRNOT00000018479	1.27	18.5	11.6	turq	10812297	similar to KIAA0825 protein
RGD1562529	ENSRNOT00000022875	1.20	13.2	3.4	turq	10849172	similar to hypothetical protein FLJ21439
**RGD1563070**	**NM_001134541**	**1.30**	**34.4**	**23.3**	**turq**	**10779602**	**similar to hypothetical protein**
RGD1563774	ENSRNOT00000045483	0.72	-6.9	2.6	turq	10875576	similar to hypothetical protein 4930474N05
RGD1564386	XM_001074961	0.79	-3.1	21.3	turq	10824808	similar to TDPOZ3
RGD1565192	NM_001134639	1.25	4.6	10.6	turq	10777034	similar to 1810013D10Rik protein
Tdrd3	NM_001012043	1.27	26.1	18.7	turq	10781752	tudor domain containing 3
Tmem150c	NM_001108354	0.71	-65.1	14.7	turq	10771456	transmembrane protein 150C
Tmem205	NM_001106804	1.30	24.5	7.0	turq	10915657	transmembrane protein 205
Tmtc3	NM_001135858	1.21	26.4	8.7	turq	10902024	transmembrane and tetratricopeptide repeat containing 3
Wdr61	NM_001025743	1.21	32.0	16.3	turq	10917583	WD repeat domain 61
**ESTs**							
	**rno-mir-99a**	**1.31**	**12.8**	**30.4**	**turq**	**10749975**	
	**---**	**0.66**	**-17.3**	**30.2**	**turq**	**10875062**	
	**ENSRNOT00000061600**	**0.78**	**-5.0**	**24.9**	**turq**	**10752972**	
	**ENSRNOT00000063561**	**0.70**	**-14.3**	**24.4**	**turq**	**10874979**	
	**ENSRNOT00000054098**	**0.81**	**-3.2**	**23.4**	**turq**	**10788045**	
	**ENSRNOT00000054414**	**0.65**	**-29.0**	**22.7**	**turq**	**10710087**	
RGD1563483	ENSRNOT00000034101	0.70	-8.2	21.7	turq	10771580	similar to CDNA sequence BC061212
	ENSRNOT00000009238	1.23	10.1	20.4	turq	10733004	
	ENSRNOT00000041961	1.73	31.3	19.8	turq	10800562	
RGD1306682	NM_001108296	1.22	27.4	19.7	turq	10746901	similar to RIKEN cDNA 1810046J19
	ENSRNOT00000053941	0.81	-2.5	19.4	turq	10845397	
	ENSRNOT00000056532	0.81	-2.4	18.9	turq	10879676	
	ENSRNOT00000063429	1.21	6.3	18.8	turq	10917318	
	---	0.66	-14.8	18.7	turq	10864479	
	ENSRNOT00000005365	0.81	-2.5	18.4	turq	10884736	
	ENSRNOT00000055580	0.82	-2.5	17.8	turq	10746440	
LOC500124	BC078992	1.29	5.2	17.7	turq	10862505	similar to RIKEN cDNA 4921507P07
RGD1560108	NM_001109161	0.81	-6.0	17.7	turq	10713512	similar to RIKEN cDNA 2700081O15
	---	1.24	2.5	17.3	turq	10769229	
	ENSRNOT00000044243	0.66	-9.5	17.3	turq	10802991	
RGD1311021	NM_001107530	0.83	-5.4	17.2	turq	10723013	hypothetical LOC308765
	NC_001665	0.76	-530	16.0	turq	10930593	
	ENSRNOT00000059302	0.80	-2.6	15.9	turq	10877376	
	ENSRNOT00000037606	0.83	-5.4	15.3	turq	10814851	
	ENSRNOT00000060414	0.76	-4.1	14.5	turq	10779984	
	ENSRNOT00000060414	0.76	-4.1	14.5	turq	10780062	
	NM_001170550	1.27	47.1	14.3	turq	10801506	
	ENSRNOT00000052503	0.78	-2.4	14.0	turq	10767629	
	ENSRNOT00000011389	1.25	23.0	13.9	turq	10855599	
	ENSRNOT00000052904	1.24	6.9	13.9	turq	10934710	
	---	0.68	-10.9	13.6	turq	10877667	
	NC_001665	0.61	-193	13.5	turq	10930588	
	---	0.83	-2.2	13.2	turq	10822031	
	GENSCAN00000008612	0.79	-2.5	12.8	turq	10812901	
	ENSRNOT00000034149	0.78	-4.3	12.8	turq	10722501	
	ENSRNOT00000052308	0.82	-2.2	12.7	turq	10775303	
	ENSRNOT00000043325	0.79	-13.0	12.4	turq	10872324	

	ENSRNOT00000040147	0.83	-2.8	11.4	turq	10707912	
	ENSRNOT00000037995	1.23	13.1	11.2	turq	10758724	
	---	0.83	-6.5	11.2	turq	10778586	
	ENSRNOT00000068960	1.21	6.0	11.2	turq	10904356	
	ENSRNOT00000047908	0.81	-4.7	10.7	turq	10702656	
	ENSRNOT00000055984	0.78	-15.4	10.7	turq	10873027	
RGD1308544	ENSRNOT00000023969	1.24	8.7	10.7	turq	10796887	LOC361192
	ENSRNOT00000068390	1.22	27.8	10.7	turq	10796679	
	---	1.24	11.8	10.3	turq	10778799	
	ENSRNOT00000040711	0.79	-4.0	10.2	turq	10813045	
	ENSRNOT00000042300	0.79	-4.2	9.8	turq	10856100	
	---	0.72	-37.4	9.8	turq	10788687	
	---	1.31	21.6	9.7	turq	10931176	
	ENSRNOT00000013868	1.22	3.1	9.7	turq	10785473	
	rno-mir-150	0.83	-3.4	9.5	turq	10706722	
	GENSCAN00000039416	0.60	-50.4	9.3	turq	10704655	
	ENSRNOT00000054249	0.77	-6.1	9.1	turq	10778315	
	ENSRNOT00000037540	0.71	-17.3	8.6	turq	10781962	
	ENSRNOT00000037540	0.71	-17.3	8.6	turq	10864711	
	ENSRNOT00000045197	1.27	4.0	8.5	turq	10788453	
	ENSRNOT00000051642	1.26	2.8	8.3	turq	10847426	
	ENSRNOT00000058227	0.76	-107	7.8	turq	10846255	
	ENSRNOT00000045406	1.31	15.2	7.6	turq	10881451	
	ENSRNOT00000054456	1.25	21.6	7.5	turq	10752726	
	ENSRNOT00000061266	0.82	-2.1	7.2	turq	10932104	
RGD1560277	XM_001066043	1.21	5.6	7.2	turq	10708167	similar to RIKEN cDNA D330012F22 gene
	ENSRNOT00000054600	0.78	-13.4	7.0	turq	10887064	
	ENSRNOT00000047223	0.82	-55.9	6.9	turq	10787828	
	rno-mir-297	0.64	-12.7	6.6	turq	10748763	
RGD1563020	ENSRNOT00000016940	1.50	13.0	6.4	turq	10926747	similar to OTTHUMP00000016566
LOC499823	ENSRNOT00000046112	0.77	-6.7	6.2	turq	10837321	LRRG00114
	ENSRNOT00000057530	0.69	-10.1	5.8	turq	10743683	
	ENSRNOT00000045544	0.81	-3.8	5.7	turq	10870890	
	ENSRNOT00000019601	0.82	-21.9	5.3	turq	10915751	
	---	0.50	-37.5	5.1	turq	10815996	
	NC_001665	0.63	-1172	4.8	turq	10930580	
	ENSRNOT00000019601	0.78	-32.4	4.7	turq	10799084	
	ENSRNOT00000061602	1.21	13.1	4.7	turq	10888395	
	---	0.75	-65.8	4.4	turq	10805722	
	ENSRNOT00000042217	0.80	-2.2	4.4	turq	10719064	
	---	0.81	-3.0	4.3	turq	10927211	
	ENSRNOT00000052493	0.82	-4.2	3.8	turq	10870039	
	ENSRNOT00000011530	1.32	22.1	3.7	turq	10891878	
	---	1.26	4.9	3.7	turq	10935213	
	---	1.31	8.3	3.6	turq	10861115	
	ENSRNOT00000053261	0.77	-49.5	3.6	turq	10707641	
	ENSRNOT00000044832	0.82	-5.0	1.9	turq	10817985	
	ENSRNOT00000037540	0.67	-34.6	1.8	turq	10766998	
	ENSRNOT00000023480	0.80	-9.6	0.0	grey	10876015	
	ENSRNOT00000034426	0.76	-21.7	0.0	grey	10711987	

*** - genes belonning to top 10% of each module withing each brain region are marked by bold font**

**- Modules and k.in are given for seaparate networks; abbreviations used for modules: *turq* - turquoise, *ylw* -yellow, *brwn* - brown

Table S2-E.Differentially Expressed Genes in Male Rat Olfactory Bulbs (352 genes and ESTs)

Gene Symbol	GenBank, Ref.Sequence	Vin/Con Ratio	Vin-Con mean_dif	k.in **	Module**	ProbeSet ID	Gene Title
**Apoptosis**							
Casp3	NM_012922	1.31	23.7	0.5	red	10791652	caspase 3
Tmbim1	NM_001007713	1.48	46.0	25.0	turq	10928902	transmembrane BAX inhibitor motif containing 1
Tnfrsf11b	NM_012870	0.81	-5.5	8.2	turq	10903725	tumor necrosis factor receptor superfamily, member 11b
**Cell Cycle**							
**Ccnd1**	**NM_171992**	**1.44**	**37.7**	**27.3**	**turq**	**10727260**	**cyclin D1**
Ccnd2	NM_022267	1.28	112	18.2	turq	10865782	cyclin D2
Cdkl4	ENSRNOT00000061975	1.31	8.9	4.6	turq	10888046	cyclin-dependent kinase-like 4
Egr1	NM_012551	0.72	-65.6	0.9	brwn	10800919	early growth response 1
**Cytoskeleton-ECM**							
Afap1l2	ENSRNOT00000023113	1.42	6.8	16.2	turq	10731025	actin filament associated protein 1-like 2

Cdh19	NM_001009448	1.60	14.3	4.0	turq	10767016	cadherin 19, type 2
Cdh20	NM_001012748	0.82	-15.2	0.5	green	10763225	cadherin 20
Csrp1	NM_017148	1.29	180	16.9	turq	10764221	cysteine and glycine-rich protein 1
**Dag1**	**ENSRNOT00000026327**	**1.31**	**60.6**	**29.8**	**turq**	**10920298**	**dystroglycan 1 (dystrophin-associated glycoprotein 1)**
**Dnali1**	**NM_001031647**	**1.28**	**3.6**	**1.6**	**green**	**10879718**	**dynein, axonemal, light intermediate chain 1**
Frem2	ENSRNOT00000031487	1.24	4.7	4.6	turq	10823127	Fras1 related extracellular matrix protein 2
Lgmn	NM_022226	1.31	93.9	21.2	turq	10891834	legumain
Lims2	NM_001012163	1.26	23.1	21.0	turq	10803602	LIM and senescent cell antigen like domains 2
Lman2l	NM_001106900	1.25	29.1	2.4	blue	10927352	lectin, mannose-binding 2-like
Matn2	ENSRNOT00000008361	1.51	40.2	18.0	turq	10896065	matrilin 2
**Msn**	**NM_030863**	**1.23**	**12.8**	**4.2**	**blue**	**10934056**	**moesin**
Nid1	ENSRNOT00000003349	1.32	6.8	7.5	turq	10799017	nidogen 1
Scin	NM_198748	1.36	4.4	0.4	brwn	10889731	scinderin
Smtnl2	ENSRNOT00000020379	1.25	2.7	10.3	turq	10744709	smoothelin-like 2
**Sparc**	**NM_012656**	**1.24**	**337**	**30.6**	**turq**	**10742802**	**secreted protein, acidic, cysteine-rich (osteonectin)**
Stom	NM_001011965	1.69	12.8	15.6	turq	10844666	stomatin
Tppp3	NM_001009639	1.45	16.1	16.8	turq	10810631	tubulin polymerization-promoting protein family member 3
Tspan18	NM_001107750	1.47	9.7	14.1	turq	10847562	tetraspanin 18
**Development**							
Adfp	NM_001007144	1.25	5.9	11.6	turq	10877907	adipose differentiation related protein
Aph1b	NM_001047090	1.30	24.7	0.1	ylw	10918368	anterior pharynx defective 1 homolog B (C. elegans)
Cryab	NM_012935	1.37	50.9	18.0	turq	10909892	crystallin, alpha B
Gulp1	NM_001013171	1.27	6.7	8.4	turq	10923036	GULP, engulfment adaptor PTB domain containing 1
**Lgi4**	**NM_199499**	**1.28**	**62.3**	**32.2**	**turq**	**10706088**	**leucine-rich repeat LGI family, member 4**
Lrrc46	NM_001004201	1.22	5.4	1.1	green	10746619	leucine rich repeat containing 46
Lrrc48	NM_001013857	1.23	24.6	3.1	blue	10734045	leucine rich repeat containing 48
Mmachc	NM_001107962	1.22	19.2	3.0	blue	10878905	methylmalonic aciduria (cobalamin deficiency) cblC type, with homocystinuria
Mmd2	NM_001037217	1.30	199	24.8	turq	10756844	monocyte to macrophage differentiation-associated 2
Mpzl1	NM_001007728	1.29	36.6	24.1	turq	10769538	myelin protein zero-like 1
Nedd9	NM_001011922	1.23	12.5	9.0	turq	10794599	neural precursor cell expressed, developmentally down-regulated 9
Nell1	NM_031069	1.23	50.9	8.4	turq	10707275	NEL-like 1 (chicken)
Nphp1	NM_001106506	0.80	-16.4	0.7	brwn	10849735	nephronophthisis 1 (juvenile) homolog (human)
Olfml1	NM_001013192	1.68	82.8	12.1	turq	10709687	olfactomedin-like 1
Pdpn	NM_019358	1.29	56.6	11.8	turq	10881293	podoplanin
**Pion**	**NM_001107845**	**1.52**	**8.7**	**1.4**	**red**	**10860235**	**pigeon homolog (Drosophila)**
Pmp22	NM_017037	1.31	53.6	18.0	turq	10734382	peripheral myelin protein 22
Postn	NM_001108550	1.75	6.5	8.7	turq	10815369	periostin, osteoblast specific factor
RGD1564081	ENSRNOT00000031492	1.22	7.1	1.5	turq	10914853	similar to novel protein similar to human oligophrenin 1 (OPHN1)
Sema3g	NM_001100882	1.29	3.1	11.3	turq	10786646	sema domain, immunoglobulin domain (Ig), short basic domain, secreted, (semaphorin) 3G
Sfrp5	NM_001107591	1.66	9.8	14.4	turq	10715448	secreted frizzled-related protein 5
Silv	NM_001108729	1.50	21.8	19.7	turq	10893213	silver homolog (mouse)
Sntb1	NM_001130542	1.43	21.6	11.6	turq	10903816	syntrophin, beta 1
Sox2	NM_001109181	1.33	26.5	2.6	blue	10814847	SRY (sex determining region Y)-box 2
Synpo2	ENSRNOT00000019931	1.25	2.5	25.1	turq	10826541	synaptopodin 2
Tor1aip2	NM_001165896	1.20	34.5	0.6	blue	10764837	torsin A interacting protein 2
Unc13c	NM_173146	1.34	16.2	0.6	blue	10918674	unc-13 homolog C (C. elegans) [=Munc13-3]
Vwa5a	NM_198755	1.21	6.8	4.0	blue	10909210	von Willebrand factor A domain containing 5A
Vwce	ENSRNOT00000028268	1.39	5.5	1.1	red	10713945	von Willebrand factor C and EGF domains
**DNA Repair**							
Tdp1	NM_001031657	1.20	5.3	1.0	brwn	10886347	tyrosyl-DNA phosphodiesterase 1
**Electron Transport**							
Cyp39a1	NM_001106893	1.44	6.3	3.8	turq	10926668	cytochrome P450, family 39, subfamily a, polypeptide 1
Pdcl	NM_022247	1.28	41.0	2.3	blue	10844776	phosducin-like
**Epigenetics**							
Cav1	NM_031556	1.38	26.3	2.6	blue	10853816	caveolin 1, caveolae protein
Rcc2	ENSRNOT00000008940	1.32	44.9	9.8	turq	10873567	regulator of chromosome condensation 2
Satb1	NM_001012129	0.83	-29.6	0.6	brwn	10921274	SATB homeobox 1
Satb2	NM_001109306	0.76	-7.8	3.1	turq	10928191	SATB homeobox 2
Hist2h2be	ENSRNOT00000028770	1.47	11.6	0.4	red	10817529	histone cluster 2, H2be
**Golgi Apparatus**							
**Hexb**	**NM_001011946**	**1.21**	**63.9**	**4.2**	**blue**	**10820693**	**hexosaminidase B**
Hs2st1	NM_001100518	1.25	54.4	6.1	turq	10827129	heparan sulfate 2-O-sulfotransferase 1
St6galnac2	NM_001031652	1.59	6.1	19.8	turq	10749330	ST6 (alpha-N-acetyl-neuraminyl-2,3-beta-galactosyl-1,3)-N-acetylgalactosaminide alpha-2,6-sial
**Growth Factors**							
Bmp15	NM_021670	1.24	2.7	2.8	turq	10937238	bone morphogenetic protein 15
Ccl2	NM_031530	1.30	5.1	8.5	turq	10736697	chemokine (C-C motif) ligand 2
Ccl7	NM_001007612	1.42	4.7	0.3	red	10736702	chemokine (C-C motif) ligand 7
Cx3cr1	NM_133534	1.38	47.4	11.2	turq	10920981	chemokine (C-X3-C motif) receptor 1
Cxcr4	NM_022205	1.33	6.6	9.8	turq	10767373	chemokine (C-X-C motif) receptor 4

Efemp1	NM_001012039	1.34	30.9	8.3	turq	10774638	EGF-containing fibulin-like extracellular matrix protein 1
Gab1	NM_001108444	1.29	28.3	21.3	turq	10806927	GRB2-associated binding protein 1
Lass2	NM_001033700	1.24	24.8	15.2	turq	10817383	LAG1 homolog, ceramide synthase 2
Megf10	NM_001100657	1.46	29.5	19.7	turq	10801833	multiple EGF-like domains 10
Nrg3	ENSRNOT00000046842	0.80	-34.4	1.7	turq	10790605	neuregulin 3
Pdgfc	NM_031317	1.45	39.1	17.0	turq	10816026	platelet derived growth factor C
Spon1	NM_172067	1.29	50.3	27.1	turq	10710067	spondin 1, extracellular matrix protein
**Immune Response**							
C1ql2	NM_001105949	1.29	10.8	8.6	turq	10931669	complement component 1, q subcomponent-like 2
C3ar1	NM_032060	1.20	2.9	2.2	blue	10865369	complement component 3a receptor 1
Cd14	NM_021744	1.26	5.2	10.5	turq	10803991	CD14 molecule
Cd200	NM_031518	1.23	79.5	12.2	turq	10751091	Cd200 molecule
**Cd9**	**NM_053018**	**1.43**	**97.3**	**31.7**	**turq**	**10865715**	**CD9 molecule**
Igsf1	NM_175763	1.25	31.8	5.1	turq	10939668	immunoglobulin superfamily, member 1
Lrig1	ENSRNOT00000017384	1.28	15.2	16.5	turq	10864279	leucine-rich repeats and immunoglobulin-like domains 1
Ly6g6e	NM_001001972	1.20	3.4	18.3	turq	10831229	lymphocyte antigen 6 complex, locus G6E
Mxra8	NM_001007002	1.38	13.3	12.3	turq	10874728	matrix-remodelling associated 8
RGD1308195	NM_001130551	1.48	5.3	11.3	turq	10904525	similar to secreted Ly6 <lymphocyte antigen 6 complex>
RT1-CE16	NM_001008839	1.21	3.4	15.9	turq	10833969	RT1 class I, locus CE16
**Metabolism & Transport**							
Stard9	ENSRNOT00000048141	0.80	-4.3	7.9	turq	10839058	StAR-related lipid transfer (START) domain containing 9
Accn4	NM_022234	1.21	6.1	13.6	turq	10924576	amiloride-sensitive cation channel 4, pituitary
**Acsbg1**	**NM_134389**	**1.38**	**242**	**28.8**	**turq**	**10917568**	**acyl-CoA synthetase bubblegum family member 1**
Akr1c19	NM_001100576	1.24	4.6	1.8	blue	10796050	aldo-keto reductase family 1, member C19
Aldh7a1	ENSRNOT00000020325	1.23	48.5	11.1	turq	10804522	aldehyde dehydrogenase 7 family, member A1
Aldh9a1	NM_022273	1.26	30.2	3.9	turq	10765399	aldehyde dehydrogenase 9 family, member A1
Aldoc	NM_012497	1.29	767	15.5	turq	10745095	aldolase C, fructose-bisphosphate
Asrgl1	NM_145089	1.21	88.6	23.8	turq	10728578	asparaginase like 1
B3galt5	NM_001105887	1.21	9.3	1.4	turq	10750489	UDP-Gal:betaGlcNAc beta 1,3-galactosyltransferase, polypeptide 5
B3gnt4	NM_001105938	0.76	-4.1	10.9	turq	10761880	UDP-GlcNAc:betaGal beta-1,3-N-acetylglucosaminyltransferase 4
Car5b	NM_001005551	1.40	20.0	1.1	blue	10933449	carbonic anhydrase 5b, mitochondrial
Car8	NM_001009662	0.79	-12.5	0.5	red	10875375	carbonic anhydrase 8
**Clic6**	**NM_176078**	**1.42**	**5.0**	**1.0**	**brwn**	**10750296**	**chloride intracellular channel 6**
Ddah2	NM_212532	1.35	55.1	19.1	turq	10831240	dimethylarginine dimethylaminohydrolase 2
Elovl2	NM_001109118	1.32	68.0	0.6	red	10794609	elongation of very long chain fatty acids (FEN1
**Fads1**	**NM_053445**	**1.25**	**143**	**32.6**	**turq**	**10713857**	**fatty acid desaturase 1**
Fnta	NM_012847	1.20	66.0	21.0	turq	10788766	farnesyltransferase, CAAX box, alpha
**Fntb**	**NM_172034**	**1.29**	**14.8**	**30.4**	**turq**	**10885450**	**farnesyltransferase, CAAX box, beta**
Fuca1	NM_012562	1.32	32.7	13.4	turq	10872940	fucosidase, alpha-L- 1, tissue
Galntl6	NM_001135756	0.83	-57.9	3.0	turq	10787962	UDP-N-acetyl-alpha-D-galactosamine:polypeptide N-acetylgalactosaminyltransferase-like 6
Gfpt2	NM_001002819	1.43	7.1	16.6	turq	10733139	glutamine-fructose-6-phosphate transaminase 2
Glb1	NM_001108192	1.39	24.7	16.0	turq	10913974	galactosidase, beta 1
Gltp	NM_001134413	1.27	24.5	20.8	turq	10759089	glycolipid transfer protein
Gpld1	NM_001100512	1.32	35.0	14.1	turq	10798331	glycosylphosphatidylinositol specific phospholipase D1
Gpt2	NM_001012057	1.23	16.7	12.7	turq	10809824	glutamic pyruvate transaminase (alanine aminotransferase) 2
Gsta3	NM_031509	1.30	184	10.0	turq	10926958	glutathione S-transferase A3
Hadh	NM_057186	1.33	66.5	24.5	turq	10826828	hydroxyacyl-Coenzyme A dehydrogenase
Hpdl	NM_001014068	1.29	4.0	17.2	turq	10878920	4-hydroxyphenylpyruvate dioxygenase-like
Hsd17b11	NM_001004209	1.26	59.5	21.1	turq	10771224	hydroxysteroid (17-beta) dehydrogenase 11
Kcnj16	NM_053314	1.42	44.2	1.0	blue	10739351	potassium inwardly-rectifying channel, subfamily J, member 16
Kcnk13	NM_022293	0.82	-3.8	0.3	brwn	10886364	potassium channel, subfamily K, member 13
**Kctd12**	**XM_001076656**	**1.21**	**3.4**	**27.6**	**turq**	**10785626**	**potassium channel tetramerisation domain containing 12**
Kctd18	NM_001106914	1.26	15.1	1.2	blue	10928220	potassium channel tetramerisation domain containing 18
**Kirrel**	**NM_207606**	**1.27**	**8.5**	**28.2**	**turq**	**10824123**	**kin of IRRE like (Drosophila)**
Lpcat2	ENSRNOT00000022359	1.21	13.9	0.2	brwn	10809524	lysophosphatidylcholine acyltransferase 2
Mboat2	NM_001108016	1.25	53.1	10.1	turq	10883912	membrane bound O-acyltransferase domain containing 2
Mfsd2	NM_001106683	1.35	29.3	22.3	turq	10879516	major facilitator superfamily domain containing 2
Moxd1	ENSRNOT00000061234	1.35	47.2	15.0	turq	10717240	monooxygenase, DBH-like 1
Mthfd1	NM_022508	1.28	8.3	9.2	turq	10839726	methylenetetrahydrofolate dehydrogenase (NADP+ dependent) 1, methenyltetrahydrofolate
Mthfd2	NM_001109398	0.80	-7.7	18.8	turq	10870316	methylenetetrahydrofolate dehydrogenase (NADP+ dependent) 2, methenyltetrahydrofolate
Nit2	NM_001034126	1.25	21.2	6.8	turq	10750731	nitrilase family, member 2
Nnt	NM_001013157	1.22	37.9	3.3	blue	10821556	nicotinamide nucleotide transhydrogenase
Oas1i	NM_001009680	1.24	3.5	8.9	turq	10758771	2 ' -5 ' oligoadenylate synthetase 1I
Papss1	NM_001106471	1.28	65.0	14.2	turq	10819066	3'-phosphoadenosine 5'-phosphosulfate synthase 1
Pla2g5	NM_017174	1.44	6.7	9.2	turq	10880872	phospholipase A2, group V
Plcd4	NM_080688	1.30	20.5	17.2	turq	10924335	phospholipase C, delta 4
Pon2	NM_001013082	1.27	111	2.3	turq	10860888	paraoxonase 2
Ppic	NM_001004215	1.32	9.7	13.2	turq	10804480	peptidylprolyl isomerase C
Psat1	NM_198738	1.47	159	3.6	blue	10729096	phosphoserine aminotransferase 1

Pygl	NM_022268	1.27	5.1	0.7	brwn	10890441	phosphorylase, glycogen, liver
Retsat	NM_145084	1.38	121	9.8	turq	10856424	retinol saturase (all trans retinol 13,14 reductase)
Sardh	NM_053664	1.30	7.2	23.9	turq	10843938	sarcosine dehydrogenase
Slc15a2	NM_031672	1.22	63.6	1.1	blue	10751404	solute carrier family 15 (H+
Slc1a3	NM_019225	1.21	221	3.8	blue	10821824	solute carrier family 1 (glial high affinity glutamate transporter), member 3
Slc22a2	NM_031584	1.43	6.2	8.4	turq	10717955	solute carrier family 22 (organic cation transporter), member 2
Slc24a4	NM_001108051	1.23	15.8	1.6	green	10886429	solute carrier family 24 (sodium
Slc25a20	NM_053965	1.25	26.9	8.3	turq	10913218	solute carrier family 25 (carnitine
Slc28a2	NM_031664	0.72	-8.8	6.1	turq	10849279	solute carrier family 28 (sodium-coupled nucleoside transporter), member 2
Slc2a5	NM_031741	1.25	11.5	12.6	turq	10874126	solute carrier family 2 (facilitated glucose
Slc3a2	NM_019283	1.28	195	22.3	turq	10728507	solute carrier family 3 (activators of dibasic and neutral amino acid transport), member 2
Slc43a3	NM_001107743	1.57	14.8	22.9	turq	10837366	solute carrier family 43, member 3
Slc44a2	NM_001134715	1.22	37.1	17.4	turq	10908369	solute carrier family 44, member 2
Slc4a2	NM_017048	1.25	8.7	16.9	turq	10860021	solute carrier family 4 (anion exchanger), member 2
**Sod3**	**NM_012880**	**1.71**	**44.8**	**28.4**	**turq**	**10777108**	**superoxide dismutase 3, extracellular**
Stoml3	NM_001106431	1.54	7.9	0.6	red	10815341	stomatin (Epb7.2)-like 3
Trak2	NM_133560	1.27	78.7	10.7	turq	10928307	trafficking protein, kinesin binding 2
Ugdh	NM_031325	1.23	21.6	2.6	blue	10772705	UDP-glucose dehydrogenase
**Proteolysis**							
Cndp2	NM_001010920	1.23	31.4	5.9	turq	10805540	CNDP dipeptidase 2 (metallopeptidase M20 family)
**Htra1**	**NM_031721**	**1.39**	**156**	**34.3**	**turq**	**10711566**	**HtrA serine peptidase 1**
Laptm4b	NM_001013174	1.30	99	20.6	turq	10896056	lysosomal protein transmembrane 4 beta
Pgc	NM_133284	2.27	12.6	17.6	turq	10926299	progastricsin (pepsinogen C)
Plat	NM_013151	1.37	36.2	1.4	green	10792421	plasminogen activator, tissue
rnf141	NM_001001800	1.20	84.6	3.4	blue	10724888	ring finger protein 141
Serpinb1a	NM_001031642	1.23	6.4	2.5	blue	10794866	serine (or cysteine) proteinase inhibitor, clade B, member 1a
**Serpine2**	**NM_019197**	**1.29**	**110**	**29.0**	**turq**	**10929288**	**serine (or cysteine) peptidase inhibitor, clade E, member 2**
Tmprss5	NM_153311	1.42	5.8	9.2	turq	10909828	transmembrane protease, serine 5
**Receptors & Binding Proteins**							
Efcab1	NM_001106930	1.32	4.8	1.0	blue	10931558	EF hand calcium binding domain 1
Ephb1	NM_001104528	1.22	15.7	21.6	turq	10919590	Eph receptor B1
Frzb	NM_001100527	1.27	13.8	13.9	turq	10846740	frizzled-related protein
Fzd8	NM_001044251	1.27	12.6	4.7	turq	10795673	frizzled homolog 8 (Drosophila)
Gfra1	NM_012959	1.32	22.2	9.6	turq	10731075	GDNF family receptor alpha 1
Ifngr1	NM_053783	1.23	36.1	15.8	turq	10701924	interferon gamma receptor 1
LOC305633	ENSRNOT00000000092	1.27	5.7	0.3	brwn	10771535	similar to Antxr2 <anthrax toxin receptor 2> protein
**Lrp4**	**NM_031322**	**1.29**	**30.4**	**28.9**	**turq**	**10837881**	**low density lipoprotein receptor-related protein 4**
Lrp5	NM_001106321	1.24	4.3	17.3	turq	10727349	low density lipoprotein receptor-related protein 5
**Mt1a**	**NM_138826**	**1.30**	**52.7**	**32.9**	**turq**	**10809392**	**metallothionein 1a**
Mt2A	NM_001137564	1.22	112	12.6	turq	10809399	metallothionein 2A
Nab1	NM_022856	0.83	-8.7	0.3	green	10923198	Ngfi-A binding protein 1
Ntsr1	NM_001108967	1.25	5.5	11.5	turq	10842828	neurotensin receptor 1
Olr1645	NM_001000102	1.21	2.0	0.2	ylw	10783366	olfactory receptor 1645
**Ppfibp2**	**NM_001100582**	**1.21**	**6.8**	**5.9**	**blue**	**10709691**	**PTPRF interacting protein, binding protein 2 (liprin beta 2)**
**Rlbp1**	**NM_001106274**	**1.35**	**72.2**	**29.3**	**turq**	**10722918**	**retinaldehyde binding protein 1**
Rxrg	NM_031765	1.56	15.8	15.5	turq	10765413	retinoid X receptor gamma
Sobpl	NM_001104640	0.81	-13.4	0.5	brwn	10833823	sine oculis-binding protein homolog-like (Drosophila)
Tacr3	NM_017053	0.83	-7.1	0.4	brwn	10819139	tachykinin receptor 3
Tax1bp3	NM_001025419	1.42	21.9	23.5	turq	10735662	Tax1 (human T-cell leukemia virus type I) binding protein 3
Ttr	NM_012681	2.03	34.9	1.8	turq	10800426	transthyretin
**Signaling**							
Ak3	NM_013218	1.35	54.9	24.4	turq	10729590	adenylate kinase 3
Bcar3	NM_001107722	1.25	6.7	15.8	turq	10818809	breast cancer anti-estrogen resistance 3
Cmtm3	NM_001106164	1.30	5.2	4.9	turq	10809018	CKLF-like MARVEL transmembrane domain containing 3
**Cnn3**	**NM_019359**	**1.27**	**81.6**	**28.0**	**turq**	**10818698**	**calponin 3, acidic**
Csmd1	NM_001037327	0.82	-19.7	10.0	turq	10789301	CUB and Sushi multiple domains 1
Ctnna1	NM_001007145	1.21	43.1	3.3	blue	10800924	catenin (cadherin associated protein), alpha 1
Dact1	ENSRNOT00000011466	1.40	6.2	16.4	turq	10885110	dapper, antagonist of beta-catenin, homolog 1 (Xenopus laevis)
Dock1	NM_001143858	1.24	26.0	3.8	blue	10711791	dedicator of cyto-kinesis 1
Dtx4	NM_001047855	1.34	16.1	20.6	turq	10729009	deltex homolog 4 (Drosophila)
Dusp16	NM_001106624	1.23	19.0	27.0	turq	10866401	dual specificity phosphatase 16
Enpp2	NM_057104	1.27	82.3	0.4	brwn	10903736	ectonucleotide pyrophosphatase
Frmd6	ENSRNOT00000009610	1.20	12.1	4.1	turq	10885015	FERM domain containing 6
Gbp5	NM_001108569	1.21	2.7	2.4	blue	10819489	guanylate binding protein 5
Gnal	ENSRNOT00000025172	1.28	53.8	4.4	turq	10802391	guanine nucleotide binding protein, alpha stimulating, olfactory type
Gpsm2	ENSRNOT00000016250	1.32	10.3	5.9	turq	10826082	G-protein signaling modulator 2 (AGS3-like, C. elegans)
**Itfg3**	**NM_001009701**	**1.22**	**9.1**	**28.2**	**turq**	**10741741**	**integrin alpha FG-GAP repeat containing 3**
**Itpr3**	**NM_013138**	**1.30**	**4.0**	**33.3**	**turq**	**10828516**	**inositol 1,4,5-triphosphate receptor, type 3**
Map3k11	NM_001013150	1.21	6.1	7.8	turq	10713102	mitogen-activated protein kinase kinase kinase 11

Nek6	NM_182953	1.29	24.1	22.9	turq	10835964	NIMA (never in mitosis gene a)-related kinase 6
Pde7a	NM_031080	0.80	-7.7	15.1	turq	10822444	phosphodiesterase 7A
Plekhb1	NM_172033	1.40	249	23.7	turq	10723992	pleckstrin homology domain containing, family B (evectins) member 1
Prkcsh	NM_001106806	1.21	39.9	8.6	turq	10908576	protein kinase C substrate 80K-H
Prkx	NM_001033963	1.21	9.4	3.4	blue	10938101	protein kinase, X-linked
**Ptprcap**	**NM_001024289**	**0.83**	**-3.2**	**5.3**	**blue**	**10712841**	**protein tyrosine phosphatase, receptor type, C-associated protein**
Rab31	NM_145094	1.21	50.9	20.1	turq	10930259	RAB31, member RAS oncogene family
Ramp2	NM_031646	1.24	12.9	11.1	turq	10738341	receptor (G protein-coupled) activity modifying protein 2
Ramp3	NM_020100	0.82	-6.0	0.7	blue	10774115	receptor (G protein-coupled) activity modifying protein 3
RGD1564174	AB510354	1.23	17.6	19.1	turq	10778404	similar to novel protein similar to Tensin Tns
RGD1565043	ENSRNOT00000016745	1.32	15.3	26.6	turq	10792662	similar to Rho guanine nucleotide exchange factor (GEF) 10
**RGD1566017**	**ENSRNOT00000017688**	**1.24**	**49.2**	**29.6**	**turq**	**10716679**	**similar to SAM and SH3 domain containing protein 1 (Proline-glutamate repeat-containing**
Rgr	NM_001107299	1.38	4.1	18.1	turq	10790555	retinal G protein coupled receptor
Rgs5	NM_019341	0.72	-29.9	0.3	ylw	10765437	regulator of G-protein signaling 5
Rhoj	NM_001008320	1.79	13.6	18.5	turq	10885299	ras homolog gene family, member J
S1pr3	ENSRNOT00000019473	1.65	12.3	15.4	turq	10797566	sphingosine-1-phosphate receptor 3
Samsn1	NM_130821	1.37	5.7	1.6	blue	10752744	SAM domain, SH3 domain and nuclear localization signals, 1
Shc4	ENSRNOT00000011084	1.47	17.1	14.3	turq	10849423	SHC (Src homology 2 domain containing) family, member 4
Sike	NM_001012182	1.21	22.1	0.2	ylw	10817910	suppressor of IKK epsilon
Stk32a	ENSRNOT00000031370	1.27	5.4	4.7	turq	10801411	serine/threonine kinase 32a
Stra6	NM_001029924	1.51	6.6	10.2	turq	10910431	stimulated by retinoic acid gene 6
Tulp3	ENSRNOT00000007634	1.29	14.5	1.1	blue	10865830	tubby-like protein 3
**Transcription**							
Btg2	NM_017259	0.73	-14.8	0.0	grey	10767767	BTG family, member 2
Ccdc80	NM_022543	1.24	5.7	4.3	turq	10753982	coiled-coil domain containing 80
Erbb3	NM_017218	1.46	17.3	9.8	turq	10899839	v-erb-b2 erythroblastic leukemia viral oncogene homolog 3 (avian)
Fat3	NM_138544	0.78	-19.7	0.0	grey	10915131	FAT tumor suppressor homolog 3 (Drosophila)
Fbxl12	NM_001025700	0.80	-4.7	12.6	turq	10915341	F-box and leucine-rich repeat protein 12
Hes1	NM_024360	1.24	8.9	18.9	turq	10754943	hairy and enhancer of split 1 (Drosophila)
Hey2	NM_130417	1.48	16.0	6.0	turq	10702361	hairy/enhancer-of-split related with YRPW motif 2
Kank1	NM_001037197	1.33	18.9	11.1	turq	10714505	KN motif and ankyrin repeat domains 1
Lats2	NM_001107267	1.28	21.7	14.7	turq	10784164	large tumor suppressor 2
Litaf	NM_001105735	1.24	9.3	19.1	turq	10731493	lipopolysaccharide-induced TNF factor
LOC684563	ENSRNOT00000061995	1.23	8.7	1.9	blue	10899741	similar to zinc finger CCCH-type containing 10
Lzic	NM_001013241	1.32	20.5	4.0	blue	10874080	leucine zipper and CTNNBIP1 domain containing
Mia	NM_030852	1.54	6.1	12.6	turq	10719977	melanoma inhibitory activity
Nfkb2	NM_001008349	1.23	2.7	20.2	turq	10715787	nuclear factor of kappa light polypeptide gene enhancer in B-cells 2, p49
Nr4a3	NM_031628	0.75	-12.9	0.1	ylw	10868940	nuclear receptor subfamily 4, group A, member 3
Ppara	NM_013196	1.38	6.8	21.8	turq	10898359	peroxisome proliferator activated receptor alpha
Pttg1ip	NM_001013238	1.27	79.5	17.1	turq	10832299	pituitary tumor-transforming 1 interacting protein
RGD1565798	XM_001060204	1.26	3.1	1.3	blue	10906647	similar to tumor protein, translationally-controlled 1
Wwtr1	NM_001024869	1.45	28.6	14.3	turq	10823284	WW domain containing transcription regulator 1
Zc3hav1	NM_173045	1.28	5.6	13.6	turq	10861976	zinc finger CCCH type, antiviral 1
Zfp583	NM_001134609	0.79	-7.2	0.8	red	10703864	zinc finger protein 583
Zfp651	ENSRNOT00000043346	1.22	13.0	6.1	turq	10921059	zinc finger protein 651
Zhx2	ENSRNOT00000007326	1.21	22.1	12.5	turq	10896632	zinc fingers and homeoboxes 2
Znf488	ENSRNOT00000031496	1.31	4.7	9.4	turq	10790433	zinc finger protein 488
**Phf11**	**NM_001024272**	**0.81**	**-3.6**	**0.6**	**ylw**	**10784227**	**PHD finger protein 11**
**Translation & Protein Modification**							
Hnrnpa2b1	NM_001104613	0.77	-51.5	9.3	turq	10862522	heterogeneous nuclear ribonucleoprotein A2
Ormdl2	NM_001105940	1.22	17.5	5.6	turq	10899943	ORM1-like 2 (S. cerevisiae)
RGD1564300	ENSRNOT00000027967	0.79	-17.1	5.1	turq	10711657	similar to phosphoseryl-tRNA kinase
Rnase4	NM_020082	1.28	26.2	2.8	blue	10779832	ribonuclease, RNase A family 4
**Rpl35a**	**NM_021264**	**0.82**	**-54.7**	**5.5**	**blue**	**10872093**	**ribosomal protein L35a**
Rrp1	NM_001012073	1.22	22.1	8.5	turq	10829196	ribosomal RNA processing 1 homolog (S. cerevisiae)
**Srrm4**	**ENSRNOT00000001507**	**0.83**	**-10.5**	**1.3**	**brwn**	**10758916**	**serine/arginine repetitive matrix 4**
**Miscellaneous & Unknown**							
Epb4.1l2	ENSRNOT00000016601	1.21	25.1	3.2	blue	10717170	erythrocyte membrane protein band 4.1-like 2
Fam111a	NM_001109163	0.73	-6.7	4.8	turq	10714106	family with sequence similarity 111, member A
Fam198b	NM_199105	1.29	7.5	16.9	turq	10816017	family with sequence similarity 198, member B
**Fam69c**	**ENSRNOT00000020858**	**1.39**	**25.8**	**32.0**	**turq**	**10803006**	**family with sequence similarity 69, member C**
Gramd3	NM_001014011	1.23	41.4	2.0	blue	10801794	GRAM domain containing 3
LOC500625	ENSRNOT00000006877	0.72	-16.1	11.7	turq	10883443	hypothetical protein LOC500625
LOC654482	NM_001039174	1.72	21.1	20.9	turq	10914823	hypothetical protein LOC654482
Mcts1	NM_001044237	0.82	-11.1	1.0	green	10936240	malignant T cell amplified sequence 1
Morn4	NM_001024975	1.29	62.6	5.8	turq	10715455	MORN repeat containing 4
Omp	NM_012616	1.50	11.2	17.2	turq	10723805	olfactory marker protein
Prrc1	NM_001033887	1.26	7.2	21.3	turq	10801859	proline-rich coiled-coil 1
Rftn2	ENSRNOT00000020910	1.21	30.2	4.2	turq	10928167	raftlin family member 2

RGD1311648	NM_001014075	1.25	21.1	9.6	turq	10883495	similar to hypothetical protein FLJ21820
RGD1564053	NM_001109259	0.82	-15.7	0.3	ylw	10875009	similar to hypothetical protein
RGD1565493	XM_002729803	1.21	7.4	0.9	green	10903292	similar to DKFZP434I092 protein
Spetex-2E	NM_001011702	1.59	17.8	1.6	blue	10779309	Spetex-2E protein
Tanc2	ENSRNOT00000003601	0.83	-36.9	8.5	turq	10739003	tetratricopeptide repeat, ankyrin repeat and coiled-coil containing 2
Tm7sf3	NM_001011970	1.24	40.7	17.4	turq	10866943	transmembrane 7 superfamily member 3
Tmem117	ENSRNOT00000008159	1.22	15.9	6.1	turq	10898879	transmembrane protein 117
Tmem150c	NM_001108354	1.47	75.8	19.4	turq	10771456	transmembrane protein 150C
Tmem167b	NM_001135260	1.22	61.9	3.8	blue	10832915	transmembrane protein 167B
Tmem176a	NM_001039008	1.35	66.3	22.4	turq	10855416	transmembrane protein 176A
Tmem195	NM_001135899	1.36	14.3	9.3	turq	10884292	transmembrane protein 195
Tmem88b	NM_001109426	1.25	21.1	5.3	turq	10882221	transmembrane protein 88B
**ESTs**							
	**---**	**0.73**	**-9.1**	**27.4**	**turq**	**10799880**	
RGD1560493	ENSRNOT00000032053	0.75	-7.6	25.1	turq	10937331	similar to chromosome X open reading frame 45
	ENSRNOT00000043108	1.22	13.6	21.0	turq	10802591	
RGD1309051	BC079166	1.20	6.7	19.9	turq	10885404	similar to chromosome 14 open reading frame 50
	ENSRNOT00000064950	0.78	-7.3	19.9	turq	10817119	
	ENSRNOT00000036735	1.31	28.3	19.3	turq	10854239	
	ENSRNOT00000053360	0.74	-10.8	16.5	turq	10887030	
	---	0.76	-4.5	15.9	turq	10823117	
	ENSRNOT00000043140	1.29	4.7	15.7	turq	10924132	
	ENSRNOT00000056335	0.81	-7.8	14.7	turq	10864908	
	ENSRNOT00000015993	1.38	37.9	14.6	turq	10808167	
	---	0.65	-20.1	12.7	turq	10708587	
	---	0.76	-8.7	12.0	turq	10905770	
RGD1305225	BC087050	0.82	-13.0	11.7	turq	10857329	similar to RIKEN cDNA C130022K22 gene
	---	1.30	5.1	8.7	turq	10766780	
	ENSRNOT00000053320	0.58	-31.7	8.3	turq	10722405	
	GENSCAN00000024407	0.73	-9.3	8.2	turq	10800742	
	ENSRNOT00000009998	1.36	18.3	8.2	turq	10859382	
	ENSRNOT00000046689	0.57	-10.1	7.9	turq	10888777	
	ENSRNOT00000005665	1.55	53.5	6.3	turq	10748601	
	rno-mir-98	1.25	5.1	5.9	turq	10933128	
	ENSRNOT00000052487	0.77	-8.6	5.7	turq	10827448	
	---	0.83	-2.3	5.0	turq	10921378	
	---	1.41	23.3	4.6	turq	10920743	
	---	0.82	-4.1	4.4	turq	10821989	
	---	1.21	5.3	3.5	turq	10939931	
	---	0.79	-7.5	3.0	turq	10896351	
	ENSRNOT00000053884	1.34	9.0	2.9	turq	10804339	
	---	1.21	2.4	2.1	turq	10830759	
	ENSRNOT00000061295	0.78	-7.2	1.8	turq	10803438	
**RGD1306739**	**NM_001134576**	**1.27**	**10.0**	**5.1**	**blue**	**10829771**	**similar to RIKEN cDNA 1700040L02**
	ENSRNOT00000057365	1.58	71.3	4.2	blue	10924441	
	ENSRNOT00000008998	2.01	34.7	3.8	blue	10918480	
	ENSRNOT00000041882	1.58	109	3.6	blue	10791650	
	---	1.91	34.4	3.3	blue	10851484	
	ENSRNOT00000053019	0.74	-219	3.0	blue	10722451	
	Y09171	1.85	34.8	2.8	blue	10780173	
	GENSCAN00000029030	0.82	-13.2	2.3	blue	10812214	
	ENSRNOT00000046129	0.71	-11.2	2.2	blue	10798473	
	ENSRNOT00000030304	0.80	-10.9	2.0	blue	10760564	
	ENSRNOT00000056343	1.39	5.1	1.9	blue	10936019	
	ENSRNOT00000052934	0.83	-35.7	1.7	blue	10722425	
	---	1.47	34.6	1.3	blue	10777420	
	---	1.30	8.9	1.3	blue	10901954	
	ENSRNOT00000053799	0.77	-22.6	1.1	blue	10722423	
	ENSRNOT00000063341	0.78	-7.3	1.0	blue	10836267	
	ENSRNOT00000055081	1.22	4.0	0.8	blue	10852153	
	ENSRNOT00000058123	1.37	8.4	0.7	blue	10902762	
	ENSRNOT00000027508	0.82	-3.1	0.7	blue	10713816	
	ENSRNOT00000042038	0.80	-2.8	0.6	blue	10852187	
	ENSRNOT00000029245	0.78	-20.6	1.0	brwn	10938793	
	ENSRNOT00000053106	0.81	-5.9	0.9	brwn	10908229	
	ENSRNOT00000068390	0.79	-33.6	0.9	brwn	10796679	
	---	0.74	-7.3	0.9	brwn	10726674	
	ENSRNOT00000041507	0.78	-5.1	0.7	brwn	10718421	
	ENSRNOT00000054628	1.37	11.8	0.2	brwn	10897037	

	rno-mir-421	0.67	-15.7	1.2	green	10938824	
	ENSRNOT00000053171	0.80	-5.8	0.6	green	10820133	
	---	0.82	-28.9	0.3	green	10809476	
	---	0.78	-2.2	1.0	red	10826983	
RGD1561795	NM_001109289	1.22	3.0	0.9	red	10914996	similar to RIKEN cDNA 1700012B09
	**ENSRNOT00000039543**	**0.83**	**-1.9**	**0.5**	**ylw**	**10805263**	
	ENSRNOT00000001469	1.21	9.4	0.4	ylw	10760483	
	ENSRNOT00000047961	1.37	5.2	0.3	ylw	10877368	
	---	0.83	-10.1	0.3	ylw	10889412	
	ENSRNOT00000032824	1.21	14.2	0.2	ylw	10760514	
	rno-mir-185	0.81	-7.5	0.0	grey	10752318	
	ENSRNOT00000007245	1.21	4.4	0.0	grey	10901836	
	---	0.82	-10.9	0.0	grey	10791602	
	---	0.82	-10.9	0.0	grey	10833416	

*** - genes belonning to top 10% of each module withing each brain region are marked by bold font**

**- Modules and k.in are given for seaparate networks; abbreviations used for modules: *turq* - turquoise, *ylw* -yellow, *brwn* - brown

Table S2-F. Differentially Expressed Genes in Male Rat Preoptic Area-Anterior Hypothalamus (41 genes and ESTs)

Gene Symbol	GenBank, Ref.Sequence	Vin/Con Ratio	Vin-Con mean_dif	k.in **	Module**	ProbeSet ID	Gene Title
**Cell Cycle**							
Egr3	NM_017086	1.25	13.9	11.5	turq	10781337	early growth response 3
**Cytoskeleton-ECM**							
Cldn2	NM_001106846	1.30	2.7	13.3	turq	10935177	claudin 2
Pcdhb5	NM_001114602	0.76	-8.0	11.1	turq	10801174	protocadherin beta 5
Sostdc1	NM_153737	1.43	6.2	13.3	turq	10884274	sclerostin domain containing 1
Stom	NM_001011965	0.82	-4.7	8.9	turq	10844666	stomatin
**Development**							
A2bp1	NM_001106974	1.21	123	7.6	turq	10740567	ataxin 2 binding protein 1
**Kl**	**NM_031336**	**1.41**	**3.7**	**13.8**	**turq**	**10759762**	**Klotho**
Nptx1	NM_153735	1.23	55.5	8.3	turq	10749523	neuronal pentraxin 1
Nptx2	NM_001034199	1.23	35.2	8.9	turq	10760290	neuronal pentraxin 2
Sncg	NM_031688	0.54	-69.3	10.2	turq	10790471	synuclein, gamma (breast cancer-specific protein 1)
**Epigenetics**							
LOC690521	ENSRNOT00000042148	0.79	-6.3	7.9	turq	10841240	similar to High mobility group protein 1 (HMG-1) (High mobility group protein B1) (Amphoterin
**Growth Factors**							
RGD1307225	NM_001107663	0.82	-5.6	13.5	turq	10814545	similar to MEGF6 <multiple EGF-like-domains 6>
**Immune Response**							
**F5**	**NM_001047878**	**1.24**	**2.3**	**13.9**	**turq**	**10765212**	**coagulation factor V (proaccelerin, labile factor)**
**Metabolism & Transport**							
Ace	NM_012544	1.23	4.6	9.3	turq	10739035	angiotensin I converting enzyme (peptidyl-dipeptidase A) 1
Clic6	NM_176078	1.23	3.1	6.6	turq	10750296	chloride intracellular channel 6
**Orai2**	**NM_001170403**	**1.30**	**8.2**	**14.1**	**turq**	**10757562**	**ORAI calcium release-activated calcium modulator 2**
**Receptors & Binding Proteins**							
LOC304239	ENSRNOT00000049215	0.76	-19.5	8.6	turq	10756334	similar to RalA binding protein 1
RGD1566059	ENSRNOT00000046220	0.80	-3.1	11.9	turq	10838423	similar to olfactory receptor Olfr1289
Ttr	NM_012681	2.40	45.8	10.1	turq	10800426	transthyretin
**Signaling**							
tGap1	NM_001007635	0.81	-19.6	11.5	turq	10834050	GTPase activating protein testicular GAP1
**Transcription**							
Anks1b	ENSRNOT00000064242	0.79	-222	9.0	turq	10894814	ankyrin repeat and sterile alpha motif domain containing 1B
Btg2	NM_017259	1.20	8.6	5.0	turq	10767767	BTG family, member 2
RGD1564400	ENSRNOT00000033258	1.24	27.3	9.0	turq	10836556	similar to Eukaryotic translation initiation factor 5 (eIF-5)
Tiam1	NM_001100558	1.20	38.5	10.1	turq	10750144	T-cell lymphoma invasion and metastasis 1
**Miscellaneous & Unknown**							
LOC292449	AY389467	1.25	6.4	7.6	turq	10701663	similar to hypothetical protein
**ESTs**							
	**ENSRNOT00000054766**	**1.25**	**4.2**	**14.4**	**turq**	**10729550**	
	---	0.78	-6.0	13.0	turq	10726674	
	ENSRNOT00000054171	0.83	-9.0	11.9	turq	10732803	
	ENSRNOT00000054104	0.81	-6.0	11.9	turq	10887336	
	ENSRNOT00000052997	0.79	-41.2	10.9	turq	10908106	
	---	0.80	-15.7	10.9	turq	10840063	
	rno-mir-99a	0.78	-11.4	10.7	turq	10749975	
	ENSRNOT00000055075	0.83	-3.1	10.5	turq	10842663	
	ENSRNOT00000053917	0.75	-9.9	10.2	turq	10887026	
	ENSRNOT00000053517	0.78	-13.9	8.5	turq	10887034	

	rno-mir-128-1	0.80	-9.6	7.9	turq	10763685	
	rno-mir-151	0.80	-4.0	7.8	turq	10904414	
	ENSRNOT00000052644	1.27	6.6	7.0	turq	10864505	
	ENSRNOT00000061351	0.82	-10.4	6.7	turq	10853290	
	ENSRNOT00000050822	0.82	-2.8	6.4	turq	10852195	
	ENSRNOT00000035499	1.21	2.1	5.9	turq	10746252	

Table S2-G. Differentially Expressed Genes in Female Rat Amygdala (133 genes & ESTs)

Gene Symbol	GenBank, Ref.Sequence	Vin/Con Ratio	Vin-Con mean_dif	k.in **	Module**	ProbeSet ID	Gene Title
**Cytoskeleton-ECM**							
Cdh29	ENSRNOT00000046259	1.24	5.1	1.1	blue	10913123	cadherin-like 29
Dnah9	ENSRNOT00000005583	0.80	-5.6	0.0	grey	10734422	dynein, axonemal, heavy polypeptide 9
Plunc	NM_172031	0.82	-1.9	2.0	blue	10841170	palate, lung, and nasal epithelium associated
Serinc2	NM_001031656	0.83	-4.7	11.3	turq	10880095	serine incorporator 2
**Development**							
Ntng1	NM_001106465	1.33	24.8	8.5	turq	10826148	netrin G1
Surf4	ENSRNOT00000007573	1.20	1.9	0.9	blue	10843907	surfeit 4
**Electron Transport**							
Cyp26a1	NM_130408	1.22	3.1	6.3	turq	10729890	cytochrome P450, family 26, subfamily a, polypeptide 1
Cyp26b1	NM_181087	0.81	-2.8	2.1	blue	10863608	cytochrome P450, family 26, subfamily b, polypeptide 1
**Immune Response**							
A2m	NM_012488	0.77	-15.2	1.7	blue	10858408	alpha-2-macroglobulin
Cd80	NM_012926	1.20	3.9	0.7	blue	10754176	Cd80 molecule
Ns5atp9	NM_201418	1.20	2.2	0.9	blue	10911010	NS5A (hepatitis C virus) transactivated protein 9
Spag6	NM_001034960	0.76	-10.0	1.9	blue	10752563	sperm associated antigen 6
**Metabolism & Transport**							
Dio2	NM_031720	1.24	12.9	6.8	turq	10891402	deiodinase, iodothyronine, type II
Echdc1	NM_001007734	1.20	32.0	11.2	turq	10717413	enoyl Coenzyme A hydratase domain containing 1
Grp	NM_133570	1.21	5.0	1.1	blue	10802331	gastrin releasing peptide
Hpgds	NM_031644	1.21	5.9	1.1	blue	10862842	hematopoietic prostaglandin D synthase
**Kcnb2***	**NM_054000**	**1.22**	**13.1**	**2.4**	**blue**	**10874952**	**potassium voltage gated channel, Shab-related subfamily, member 2**
**Proteolysis**							
Adam1a	NM_020078	0.83	-7.1	3.2	brwn	10758697	a disintegrin and metallopeptidase domain 1a
Serpini1	NM_053779	1.22	189	7.6	turq	10815962	serine (or cysteine) peptidase inhibitor, clade I, member 1
**Receptors & Binding Proteins**							
Alb	NM_134326	0.57	-15.6	7.2	turq	10775968	albumin
Olr1425	NM_001000010	1.23	2.4	1.7	blue	10742940	olfactory receptor 1425
Olr1571	ENSRNOT00000034210	0.81	-2.5	2.3	blue	10752574	olfactory receptor 1571
**Signaling**							
Alpk1	ENSRNOT00000030798	0.81	-8.9	1.9	turq	10826680	alpha-kinase 1
Defb1	NM_031810	1.20	3.9	1.2	blue	10792530	defensin beta 1
LOC689617	ENSRNOT00000058250	0.81	-3.4	0.7	blue	10815215	similar to GTPase activating protein testicular GAP1
**Ptpro**	**NM_017336**	**1.25**	**29.9**	**2.3**	**blue**	**10859342**	**protein tyrosine phosphatase, receptor type, O**
Sh3bp4	NM_022693	1.29	13.9	10.0	turq	10925264	SH3-domain binding protein 4
**Transcription**							
Ccdc112	NM_001109124	1.20	11.4	5.6	turq	10804371	coiled-coil domain containing 112
Fam134b	ENSRNOT00000014423	0.78	-9.5	2.3	brwn	10813949	family with sequence similarity 134, member B
Klf5	NM_053394	1.28	7.6	3.8	turq	10781829	Kruppel-like factor 5
**Klhdc8a**	**NM_001100683**	**1.24**	**5.6**	**2.7**	**blue**	**10763883**	**kelch domain containing 8A**
Lzic	NM_001013241	1.24	15.8	6.5	turq	10874080	leucine zipper and CTNNBIP1 domain containing
Thoc4	NM_001109602	1.23	49.8	11.8	turq	10749681	THO complex 4
Twist1	NM_053530	1.21	2.5	2.0	blue	10884215	twist homolog 1 (Drosophila)
**Translation & Protein Modification**							
RGD1562905	ENSRNOT00000068097	0.83	-2.4	11.5	turq	10812148	similar to 60S ribosomal protein L17 (L23)
**Miscellaneous & Unknown**							
Depdc7	NM_001029916	1.22	3.9	0.4	blue	10847932	DEP domain containing 7
Fam111a	NM_001109163	1.26	4.3	1.4	blue	10714106	family with sequence similarity 111, member A
LOC683001	ENSRNOT00000032112	1.22	6.2	0.9	brwn	10858945	similar to Transmembrane protein 16B
LOC691921	NM_001135566	1.23	5.2	1.7	blue	10894640	hypothetical protein LOC691921
RGD1565493	XM_002729803	1.25	8.1	6.7	turq	10903292	similar to DKFZP434I092 protein
**ESTs**							
	**ENSRNOT00000053883**	**0.75**	**-34.1**	**18.0**	**turq**	**10765040**	
	**ENSRNOT00000054102**	**0.70**	**-32.6**	**15.6**	**turq**	**10723896**	
	**ENSRNOT00000063556**	**0.75**	**-10.9**	**15.4**	**turq**	**10913940**	
	**ENSRNOT00000053497**	**0.81**	**-26.6**	**15.2**	**turq**	**10878965**	
	**ENSRNOT00000052995**	**0.79**	**-11.4**	**15.0**	**turq**	**10723898**	

	**ENSRNOT00000052632**	**0.74**	**-81.8**	**15.0**	**turq**	**10846928**	
	**ENSRNOT00000052577**	**0.75**	**-20.4**	**14.7**	**turq**	**10878961**	
	**ENSRNOT00000053479**	**0.72**	**-8.4**	**13.6**	**turq**	**10851380**	
	AF188753	0.77	-340	13.6	turq	10855185	
	ENSRNOT00000052882	0.77	-29.9	13.5	turq	10759461	
	ENSRNOT00000053230	0.71	-34.3	12.9	turq	10758033	
	ENSRNOT00000062811	0.78	-9.7	12.9	turq	10739864	
	ENSRNOT00000053447	0.80	-17.8	11.9	turq	10765495	
	ENSRNOT00000053552	0.76	-32.2	11.9	turq	10713608	
	ENSRNOT00000052472	0.83	-28.1	11.8	turq	10728563	
	ENSRNOT00000052620	0.78	-25.4	11.5	turq	10828077	
	ENSRNOT00000053542	0.77	-25.5	11.3	turq	10871771	
	ENSRNOT00000053328	0.63	-149	11.3	turq	10855946	
	ENSRNOT00000053276	0.76	-93.4	10.9	turq	10909358	
	ENSRNOT00000053402	0.69	-292	10.9	turq	10721698	
	ENSRNOT00000055917	0.75	-4	10.2	turq	10722578	
	ENSRNOT00000052527	0.73	-438	9.9	turq	10758031	
	ENSRNOT00000052442	0.72	-79.3	9.8	turq	10709825	
	ENSRNOT00000052628	0.69	-44.1	9.7	turq	10834602	
	J05014	0.82	-179	9.5	turq	10876313	
	ENSRNOT00000059482	0.80	-14.0	9.3	turq	10788070	
	ENSRNOT00000054190	0.73	-19.1	8.8	turq	10756255	
	ENSRNOT00000052441	0.79	-11.3	8.8	turq	10841716	
	ENSRNOT00000053086	0.71	-342	8.7	turq	10811999	
	ENSRNOT00000053279	0.59	-277	7.9	turq	10708691	
	ENSRNOT00000053919	0.78	-85.4	7.9	turq	10755129	
	ENSRNOT00000030972	1.39	13.9	7.8	turq	10832081	
	ENSRNOT00000052854	0.80	-10.7	7.6	turq	10932269	
	ENSRNOT00000053273	0.71	-18.4	7.2	turq	10861395	
	ENSRNOT00000054140	0.79	-16.5	7.0	turq	10783318	
	---	0.81	-3.4	6.8	turq	10805580	
	AF272707	0.76	-17.8	6.2	turq	10867318	
	GENSCAN00000004438	1.20	4.7	6.2	turq	10807906	
	ENSRNOT00000052060	0.78	-4.5	5.8	turq	10783533	
	ENSRNOT00000054468	0.73	-4.6	5.2	turq	10759475	
	ENSRNOT00000053174	0.80	-10.5	5.2	turq	10851382	
	ENSRNOT00000052749	0.79	-4.3	4.8	turq	10779724	
	rno-mir-342	0.80	-7.1	4.3	turq	10886771	
	**rno-mir-27b**	**0.73**	**-8.9**	**4.0**	**brwn**	**10796987**	
	ENSRNOT00000034750	0.81	-9.6	4.0	turq	10710063	
	ENSRNOT00000055440	0.80	-3.8	3.9	turq	10859772	
	**ENSRNOT00000053917**	**0.83**	**-7.3**	**3.7**	**brwn**	**10887026**	
	ENSRNOT00000053313	0.81	-3.5	3.6	brwn	10835528	
	ENSRNOT00000052729	0.80	-4.4	3.5	brwn	10714632	
	ENSRNOT00000053992	0.81	-5.1	3.3	turq	10800840	
	ENSRNOT00000053325	0.77	-13.1	3.3	brwn	10711125	
	**---**	**0.81**	**-3.9**	**3.2**	**blue**	**10720518**	
	---	0.78	-76.0	2.9	turq	10813353	
	ENSRNOT00000052693	0.78	-4.7	2.4	brwn	10797939	
	ENSRNOT00000056936	0.81	-16.5	2.4	brwn	10878924	
	**ENSRNOT00000059747**	**1.32**	**5.4**	**2.3**	**blue**	**10732800**	
	ENSRNOT00000042737	0.81	-2.1	2.2	blue	10716557	
	rno-mir-493	0.79	-7.8	2.2	brwn	10886826	
	ENSRNOT00000054201	0.66	-7.0	2.2	blue	10894268	
RGD1566149	NM_001109038	0.82	-4.1	2.1	blue	10736265	similar to CDNA sequence BC017647
	ENSRNOT00000029007	0.82	-3.1	2.1	blue	10750470	
	---	1.22	7.1	2.1	turq	10937568	
	ENSRNOT00000053391	0.81	-38.0	2.1	blue	10722391	
	---	0.82	-12.1	2.0	blue	10730472	
	ENSRNOT00000053969	0.78	-32.1	1.9	blue	10722367	
	ENSRNOT00000053232	0.72	-9.8	1.8	turq	10714842	
	---	1.33	6.2	1.8	blue	10837170	
	rno-mir-323	0.79	-2.3	1.8	brwn	10887056	
	ENSRNOT00000052978	0.81	-18.8	1.7	blue	10721700	
	ENSRNOT00000053364	0.72	-56.8	1.7	blue	10721694	
	ENSRNOT00000046248	0.81	-7.8	1.6	blue	10860184	
	ENSRNOT00000005897	0.75	-4.4	1.6	blue	10902694	
	---	0.77	-5.0	1.6	brwn	10767747	
RGD1308448	NM_001107671	1.22	66.8	1.6	turq	10813249	similar to RIKEN cDNA B130016O10 gene

	ENSRNOT00000044342	0.80	-19.1	1.6	blue	10738162	
	ENSRNOT00000050523	0.80	-6.2	1.5	blue	10703461	
	ENSRNOT00000037766	1.26	6.3	1.5	turq	10806541	
RGD1305592	NM_001013901	0.81	-6.1	1.5	turq	10725908	similar to RIKEN cDNA 2900092E17
	ENSRNOT00000053071	0.76	-4.6	1.4	brwn	10806012	
	ENSRNOT00000057530	0.83	-5.8	1.4	blue	10743683	
	rno-mir-297	0.75	-9.0	1.3	blue	10748763	
	ENSRNOT00000061287	0.78	-7.9	1.3	blue	10731608	
	GENSCAN00000026399	1.22	3.1	1.2	blue	10902020	
	ENSRNOT00000053902	0.83	-117	1.2	blue	10827450	
	---	0.83	-5.7	1.1	blue	10898350	
	ENSRNOT00000052689	0.76	-16.0	1.0	brwn	10896964	
	ENSRNOT00000057455	0.82	-3.9	0.9	blue	10934659	
	ENSRNOT00000054104	0.77	-9.2	0.9	blue	10887336	
	ENSRNOT00000051623	0.82	-11.9	0.8	blue	10708649	
	ENSRNOT00000052821	1.26	7.0	0.7	blue	10767278	
	ENSRNOT00000058520	1.20	5.9	0.6	brwn	10869737	
	ENSRNOT00000047961	1.31	3.9	0.4	blue	10877368	
	---	0.83	-26.1	0.0	grey	10790615	

*** - genes belonning to top 10% of each module withing each brain region are marked by bold font**

**- Modules and k.in are given for seaparate networks; abbreviations used for modules: *turq* - turquoise, *ylw* -yellow, *brwn* - brown

Table S2-H. Differentially Expressed Genes in Female Rat Hippocampus (70 genes & ESTs)

Gene Symbol	GenBank, Ref.Sequence	Vin/Con Ratio	Vin-Con mean_dif	k.in **	Module**	ProbeSet ID	Gene Title
**Cytoskeleton-ECM**							
Dsp	ENSRNOT00000018649	1.23	2.3	18.6	turq	10797966	desmoplakin
Mcam	NM_023983	0.76	-8.6	31.2	turq	10909446	melanoma cell adhesion molecule
**Myo5b**	**NM_017083**	**1.26**	**11.4**	**37.0**	**turq**	**10802650**	**myosin Vb**
Myo9b	NM_012984	0.83	-13.0	29.8	turq	10787212	myosin IXb
**Golgi Apparatus**							
Gga3	NM_001108304	0.77	-19.4	34.0	turq	10748973	golgi associated, gamma adaptin ear containing, ARF binding protein 3
**Growth Factors**							
Bmp5	NM_001108168	1.25	3.2	29.8	turq	10911711	bone morphogenetic protein 5
**Immune Response**							
Igsf1	NM_175763	1.24	26.8	31.0	turq	10939668	immunoglobulin superfamily, member 1
RT1-M6-2	NM_001008853	1.50	5.8	29.6	turq	10827691	RT1 class I, locus M6, gene 2
RT1-N2	NM_001008854	1.57	16.3	32.2	turq	10827782	RT1 class Ib, locus N2
**Metabolism & Transport**							
Aldh1a1	NM_022407	0.83	-20.9	31.9	turq	10714323	aldehyde dehydrogenase 1 family, member A1
Car3	NM_019292	1.37	12.8	32.3	turq	10822242	carbonic anhydrase 3
Endog	NM_001034938	1.22	3.9	28.9	turq	10835150	endonuclease G
Kcnj16	NM_053314	1.36	52.4	36.0	turq	10739351	potassium inwardly-rectifying channel, subfamily J, member 16
Piga	NM_001108816	0.82	-15.4	24.2	turq	10937719	phosphatidylinositol glycan anchor biosynthesis, class A
Slc16a12	ENSRNOT00000030024	1.24	5.9	24.0	turq	10729795	solute carrier family 16, member 12 (monocarboxylic acid transporter 12)
Slc28a2	NM_031664	0.80	-7.1	36.3	turq	10849279	solute carrier family 28 (sodium-coupled nucleoside transporter), member 2
**Proteolysis**							
Rnf135	ENSRNOT00000005428	1.21	8.6	34.1	turq	10736606	ring finger protein 135
**Receptors & Binding Proteins**							
**Acvr2a**	**NM_031571**	**1.22**	**66.5**	**37.7**	**turq**	**10836072**	**activin A receptor, type IIA**
Olr1171	NM_001001005	1.21	4.3	19.8	turq	10915295	olfactory receptor 1171
Olr1471	NM_001000722	1.25	4.8	30.4	turq	10744782	olfactory receptor 1471
Olr1749	NM_001001429	0.65	-6.5	34.4	turq	10827669	olfactory receptor 1749
Olr669	NM_001000349	1.29	6.2	21.6	turq	10847148	olfactory receptor 669
**Signaling**							
Ppfibp1	NM_001107896	1.22	30.5	23.7	turq	10859693	PTPRF interacting protein, binding protein 1 (liprin beta 1)
Ptpro	NM_017336	1.25	29.9	37.0	turq	10859342	protein tyrosine phosphatase, receptor type, O
Sla	NM_178097	1.21	6.3	30.9	turq	10904161	src-like adaptor
**Transcription**							
Sox11	NM_053349	1.22	13.1	33.1	turq	10889415	SRY (sex determining region Y)-box 11
Zcchc12	NM_001014065	1.21	54.0	28.6	turq	10936360	zinc finger, CCHC domain containing 12
Zfp347	NM_133390	0.82	-10.4	36.2	turq	10893424	zinc finger protein 347
Znf667	NM_001008557	1.22	20.9	35.6	turq	10718696	zinc finger protein 667
**Translation & Protein Modification**							
LOC100362277	XM_002725437	0.81	-12.9	28.2	turq	10808893	60S ribosomal protein L29-like
Mrpl22	NM_001105781	1.27	11.7	25.5	turq	10733782	mitochondrial ribosomal protein L22
Arl4a	NM_019186	1.36	21.3	26.2	turq	10889728	ADP-ribosylation factor-like 4A

**Miscellaneous & Unknown**							
Fam181b	BC158703	1.22	13.7	33.9	turq	10708687	family with sequence similarity 181, member B
LOC304558	XM_222260	1.21	5.6	36.9	turq	10762932	similar to TPR repeat-containing protein KIAA1043
RGD1307051	NM_001108091	1.36	18.0	22.3	turq	10902112	similar to hypothetical protein FLJ21963
**ESTs**							
LOC363337	BC082068	0.75	-11.9	22.2	turq	10931083	similar to RIKEN cDNA 1700081O22
LOC365647	XM_002725911	0.82	-2.6	23.6	turq	10820567	hypothetical LOC365647
	GENSCAN00000037350	0.76	-14.8	29.2	turq	10701986	
	GENSCAN00000032787	0.72	-12.3	31.0	turq	10827665	
	ENSRNOT00000066008	1.23	12.1	34.2	turq	10750750	
	**ENSRNOT00000061081**	**1.34**	**3.6**	**37.6**	**turq**	**10860779**	
	ENSRNOT00000059999	1.20	15.6	35.2	turq	10889627	
	ENSRNOT00000054827	0.72	-9.2	26.5	turq	10728256	
	ENSRNOT00000052640	0.73	-9.8	28.4	turq	10735369	
	ENSRNOT00000052448	0.76	-10.0	20.9	turq	10899123	
	ENSRNOT00000051655	1.24	10.4	24.0	turq	10937641	
	ENSRNOT00000048377	0.77	-8.5	33.9	turq	10790302	
	**ENSRNOT00000047948**	**1.51**	**10.1**	**38.5**	**turq**	**10911377**	
	ENSRNOT00000047883	1.22	4.1	29.3	turq	10900109	
	ENSRNOT00000045890	1.33	9.4	34.1	turq	10898836	
	ENSRNOT00000042326	1.21	5.6	35.1	turq	10849305	
	ENSRNOT00000041436	0.78	-2.6	35.1	turq	10869874	
	ENSRNOT00000039932	0.78	-5.1	28.2	turq	10923212	
	ENSRNOT00000029914	0.82	-4.3	33.8	turq	10934032	
	ENSRNOT00000029830	1.23	2.1	35.3	turq	10822318	
	---	0.80	-3.2	34.1	turq	10860781	
	---	1.26	10.2	36.4	turq	10825510	
	---	1.23	3.1	23.7	turq	10852985	
	---	1.21	3.8	23.3	turq	10726674	
	---	1.21	6.8	33.9	turq	10751209	
	**---**	**0.48**	**-48.2**	**37.7**	**turq**	**10715250**	
	---	0.77	-13.6	36.4	turq	10791602	
	---	0.77	-13.6	36.4	turq	10833416	
	**---**	**0.76**	**-17.6**	**37.4**	**turq**	**10911847**	
	---	0.59	-43.3	31.1	turq	10792331	
	---	0.59	-43.3	31.1	turq	10918865	
	---	0.80	-3.8	36.9	turq	10790536	
	**---**	**0.82**	**-3.5**	**37.8**	**turq**	**10840005**	
	---	1.51	10.0	32.8	turq	10823419	
	---	0.81	-15.8	35.7	turq	10750023	

*** - genes belonning to top 10% of each module withing each brain region are marked by bold font**

**- Modules and k.in are given for seaparate networks; abbreviations used for modules: *turq* - turquoise, *ylw* -yellow, *brwn* - brown

Table S2-I. Differentially Expressed Genes in Female Rat Cingulate Cortex (691 genes and ESTs)

Gene Symbol	GenBank, Ref.Sequence	Vin/Con Ratio	Vin-Con mean_dif	k.in **	Module**	ProbeSet ID	Gene Title
**Apoptosis**							
**Atg9a**	**NM_001014218**	**0.80**	**-30.2**	**91.7**	**turq**	**10929086**	**ATG9 autophagy related 9 homolog A (S. cerevisiae)**
Bag1	NM_001106647	1.22	57.0	5.5	blue	10876052	BCL2-associated athanogene
Dapk1	NM_001107335	0.83	-32.1	23.1	blue	10797032	death associated protein kinase 1
Ddx52	NM_053525	1.26	16.1	74.2	turq	10736917	DEAD (Asp-Glu-Ala-Asp) box polypeptide 52
Faim2	NM_144756	0.74	-216	48.3	turq	10907165	Fas apoptotic inhibitory molecule 2
Parm1	NM_173114	0.71	-36.5	0.0	grey	10775862	prostate androgen-regulated mucin-like protein 1
Pdcd10	NM_001009542	1.30	71.3	13.6	blue	10823733	programmed cell death 10
Tmbim4	NM_199116	1.25	26.8	22.8	blue	10895669	transmembrane BAX inhibitor motif containing 4
**Cell Cycle**							
Ccnf	NM_001100474	0.82	-3.6	2.7	brwn	10741028	cyclin F
Cep70	NM_001017470	1.24	19.7	72.7	turq	10912464	centrosomal protein 70kDa
**Egr1**	**NM_012551**	**0.67**	**-90.5**	**89.9**	**turq**	**10800919**	**early growth response 1**
Egr2	NM_053633	0.76	-7.7	0.0	grey	10832802	early growth response 2
Egr3	NM_017086	0.74	-23.2	35.4	turq	10781337	early growth response 3
Hcfc1	NM_001139507	0.83	-24.3	46.7	turq	10940271	host cell factor C1
Pak6	NM_001106498	0.80	-28.1	5.0	blue	10838670	p21 protein (Cdc42/Rac)-activated kinase 6
**RGD1560888**	**NM_001109061**	**1.25**	**90.6**	**103.0**	**turq**	**10756521**	**similar to Cell division protein kinase 8 (Protein kinase K35)**
**Cytoskeleton_EMC**							
**Mapre3**	**NM_001007656**	**0.82**	**-60.4**	**91.2**	**turq**	**10888998**	**microtubule-associated protein, RP/EB family, member 3**
Tspan5	NM_001004090	0.81	-96.2	14.2	blue	10819442	tetraspanin 5

**Zyx**	**NM_053761**	**0.79**	**-9.8**	**8.6**	**brwn**	**10855062**	**zyxin**
**Ankh**	**NM_053714**	**0.83**	**-82**	**96.5**	**turq**	**10813969**	**ankylosis, progressive homolog (mouse)**
App	NM_019288	0.83	-313	56.8	turq	10752811	amyloid beta (A4) precursor protein
Celsr2	ENSRNOT00000027263	0.80	-32.5	69.4	turq	10826002	cadherin, EGF LAG seven-pass G-type receptor 2 (flamingo homolog, Drosophila)
Cldn11	NM_053457	0.61	-91.8	4.3	brwn	10822631	claudin 11
Cntn2	NM_012884	0.63	-46.1	2.0	ylw	10767605	contactin 2 (axonal)
Col6a3	NM_001109008	0.80	-4.6	0.5	ylw	10929766	procollagen, type VI, alpha 3
Coro2b	ENSRNOT00000020951	0.82	-42.2	46.8	turq	10918075	coronin, actin binding protein, 2B
Esco1	NM_001126299	1.31	78.4	73.6	turq	10767013	establishment of cohesion 1 homolog 1 (S. cerevisiae)
Flrt1	NM_001109160	0.77	-19.1	0.7	ylw	10728375	fibronectin leucine rich transmembrane protein 1
Flrt2	NM_001106750	0.76	-14.0	46.2	turq	10886269	fibronectin leucine rich transmembrane protein 2
Fmnl1	NM_001105846	0.79	-20.2	36.3	turq	10738676	formin-like 1
Gsn	BC079472	0.79	-6.2	0.5	ylw	10835775	gelsolin
Jup	NM_031047	0.80	-16.5	11.3	turq	10747330	junction plakoglobin
Kb23	NM_001008813	1.30	4.3	2.5	ylw	10907369	type II keratin Kb23
Lmo4	NM_001009708	0.80	-129	1.2	ylw	10819562	LIM domain only 4
Lrfn1	NM_001127694	0.75	-15.8	35.0	turq	10705519	leucine rich repeat and fibronectin type III domain containing 1
**Mast3**	**NM_001134796**	**0.82**	**-24.0**	**111.8**	**turq**	**10787447**	**microtubule associated serine/threonine kinase 3**
**Mast4**	**ENSRNOT00000061032**	**0.75**	**-27.2**	**9.4**	**brwn**	**10821072**	**microtubule associated serine/threonine kinase family member 4**
Ncan	NM_031653	0.83	-23.0	2.1	blue	10787630	neurocan
Ncdn	NM_053543	0.81	-159	37.6	turq	10879867	neurochondrin
Pcdh1	ENSRNOT00000026324	0.81	-49.0	6.4	blue	10804117	protocadherin 1
Pcdhb13	ENSRNOT00000027172	0.83	-12.6	1.2	ylw	10801191	protocadherin beta 13
Pcdhb15	ENSRNOT00000027164	0.78	-10.7	17.8	turq	10801195	protocadherin beta 15
Spock1	ENSRNOT00000061187	0.79	-69.5	1.2	ylw	10793909	sparc/osteonectin, cwcv and kazal-like domains proteoglycan (testican) 1
Tbca	NM_001013245	1.25	22.0	51.1	turq	10899579	tubulin folding cofactor A
Tmsb4x	NM_031136	1.32	35.6	13.8	blue	10876291	thymosin beta 4, X-linked
Tnr	NM_013045	0.82	-39.0	12.5	blue	10764947	tenascin R
Vamp2	NM_012663	0.82	-70.0	24.4	turq	10734875	vesicle-associated membrane protein 2
**Development**							
**Dscaml1**	**NM_001108141**	**0.75**	**-17.9**	**28.4**	**blue**	**10909655**	**Down syndrome cell adhesion molecule-like 1**
Bmper	NM_001135799	0.83	-6.0	6.6	blue	10908645	BMP-binding endothelial regulator
Cops2	NM_153297	1.26	120	11.5	blue	10849454	COP9 constitutive photomorphogenic homolog subunit 2 (Arabidopsis)
Cpne4	NM_001109003	0.80	-48.2	0.0	grey	10912718	copine IV
Dgcr2	NM_001012146	0.71	-21.0	35.1	turq	10752397	DiGeorge syndrome critical region gene 2
Dirc2	NM_001012017	0.77	-42.4	44.1	turq	10751488	disrupted in renal carcinoma 2 (human)
Disp2	NM_001107759	0.78	-47.6	2.9	brwn	10838683	dispatched homolog 2 (Drosophila)
Dopey2	ENSRNOT00000002302	0.80	-25.6	73.0	turq	10750333	dopey family member 2
Egln3	NM_019371	0.80	-14.5	2.4	brwn	10889923	EGL nine homolog 3 (C. elegans)
Fis1	NM_001105919	1.20	68.9	16.8	blue	10757448	fission 1 (mitochondrial outer membrane) homolog (S. cerevisiae)
Hypk	ENSRNOT00000021091	1.21	59.2	45.8	turq	10839174	Huntingtin interacting protein K
Inhba	NM_017128	0.77	-7.9	1.0	ylw	10798702	inhibin beta-A
Lingo1	NM_001100722	0.81	-11.5	2.0	brwn	10917711	leucine rich repeat and Ig domain containing 1
LOC683626	NM_001129880	0.79	-17.6	1.1	ylw	10888608	similar to limb-bud and heart
Lrrc8b	NM_001107204	0.73	-26.7	86.2	turq	10775278	leucine rich repeat containing 8 family, member B
Lrrn2	NM_001177368	0.81	-37.7	37.1	turq	10763933	leucine rich repeat neuronal 2
LRRTM1	NM_001109374	0.81	-53.4	13.6	turq	10856472	leucine rich repeat transmembrane neuronal 1
Midn	ENSRNOT00000060698	0.81	-18.7	2.4	brwn	10900592	midnolin
Mier2	NM_001108737	0.82	-6.8	2.0	ylw	10894087	mesoderm induction early response 1, family member 2
Myadm	NM_183332	0.80	-17.7	0.0	grey	10718591	myeloid-associated differentiation marker
Nell2	NM_031070	0.83	-132	61.2	turq	10906546	NEL-like 2 (chicken)
Nenf	NM_001002851	1.21	7.2	48.8	turq	10770714	neuron derived neurotrophic factor
Nlgn2	NM_053992	0.80	-24.9	0.7	ylw	10744245	neuroligin 2
Nmd3	NM_001107682	1.26	52.3	72.4	turq	10815927	NMD3 homolog (S. cerevisiae)
Nphp4	NM_001037650	0.82	-4.0	10.6	blue	10874382	nephronophthisis 4 (juvenile) homolog (human)
Nsf	NM_021748	0.80	-225	74.3	turq	10748118	N-ethylmaleimide-sensitive factor
Ntn3	NM_053732	0.83	-3.3	1.3	ylw	10741009	netrin 3
Odz2	NM_020088	0.79	-28.3	5.8	blue	10742072	odz, odd Oz/ten-m homolog 2 (Drosophila)
Pcnxl2	XM_001055579	0.78	-29.5	58.2	turq	10812077	pecanex-like 2 (Drosophila)
Pcnxl3	ENSRNOT00000064136	0.79	-15.2	77.9	turq	10727943	pecanex-like 3 (Drosophila)
Porcn	NM_001173355	0.82	-14.3	9.3	turq	10932394	porcupine homolog (Drosophila)
**Porf-2**	**NM_173122**	**0.79**	**-14.6**	**90.9**	**turq**	**10905128**	**preoptic regulatory factor-2**
Rcan2	NM_175578	0.81	-89.0	6.9	brwn	10926658	regulator of calcineurin 2
Rex2	ENSRNOT00000040918	1.26	21.0	78.2	turq	10873880	reduced expression 2
Sar1b	NM_001009622	1.23	139	19.3	blue	10733389	SAR1 homolog B (S. cerevisiae)
Sema4d	NM_001170563	0.74	-30.5	3.3	brwn	10794242	sema domain, immunoglobulin domain (Ig), transmembrane domain (TM) and short cytoplasm
Sema7a	NM_001108153	0.75	-63.4	23.1	turq	10910406	sema domain, immunoglobulin domain (Ig), and GPI membrane anchor, (semaphorin) 7A
Sez6	ENSRNOT00000048543	0.82	-51.5	4.2	brwn	10736119	seizure related 6 homolog (mouse)
Sez6l2	NM_001107550	0.79	-48.5	64.7	turq	10710965	seizure related 6 homolog (mouse)-like 2

Slitrk1	NM_001107283	0.83	-53.5	11.6	blue	10785782	SLIT and NTRK-like family, member 1
Spata2	NM_053675	0.81	-14.3	14.9	blue	10851960	spermatogenesis associated 2
Spata2L	NM_001109133	0.78	-20.2	67.4	turq	10811747	spermatogenesis associated 2-like
Sv2a	NM_057210	0.80	-115	5.3	brwn	10817512	synaptic vesicle glycoprotein 2a
Syn1	NM_019133	0.81	-75	64.0	turq	10932107	synapsin I
Tmem59l	BC089056	0.83	-47	1.7	ylw	10787556	transmembrane protein 59-like
Usmg5	NM_133544	1.21	120	25.2	blue	10747614	up-regulated during skeletal muscle growth 5 homolog (mouse)
Vamp8	NM_031827	1.35	13.4	8.4	blue	10863218	vesicle-associated membrane protein 8
Yipf3	NM_001007801	0.82	-20.8	49.7	turq	10926503	Yip1 domain family, member 3
A2bp1	NM_001106974	0.81	-108	55.8	turq	10740567	ataxin 2 binding protein 1
**DNA Repair**							
Atmin	ENSRNOT00000015180	0.81	-21.1	26.2	turq	10903264	ATM interactor
**Gadd45b**	**NM_001008321**	**0.80**	**-7.8**	**9.2**	**brwn**	**10900358**	**growth arrest and DNA-damage-inducible, beta**
**Electron Transport**							
**Cox7a2**	**NM_022503**	**1.22**	**257**	**31.4**	**blue**	**10772901**	**cytochrome c oxidase subunit VIIa polypeptide 2**
Cox7a2l	NM_001106704	1.23	99	14.6	blue	10888127	cytochrome c oxidase subunit VIIa polypeptide 2 like
Cox7b	NM_182819	1.35	85	17.6	blue	10934579	cytochrome c oxidase subunit VIIb
Cox7c	NM_001134705	1.29	163	24.8	blue	10820277	cytochrome c oxidase, subunit VIIc
Glrx3	NM_032614	1.23	45.0	0.7	ylw	10711881	glutaredoxin 3
LOC290508	ENSRNOT00000049636	1.22	5.2	11.8	blue	10779240	similar to RIKEN cDNA 1700001F09
LOC685322	NM_001170465	1.39	65.0	23.3	blue	10778108	similar to ubiquinol-cytochrome c reductase complex 7.2kDa protein isoform b
Prdx1	NM_057114	1.21	128	17.3	blue	10871286	peroxiredoxin 1
**Txn1**	**NM_053800**	**1.26**	**131**	**29.6**	**blue**	**10877005**	**thioredoxin 1**
**Epigenetics**							
Hist1h2ak	NM_001109423	1.45	5.7	9.5	blue	10798507	histone cluster 1, H2ak
Hist1h2bc	NM_001109400	1.29	46.6	11.5	blue	10798488	histone cluster 1, H2bc
Hist1h2bl	NM_022647	1.25	25.5	14.5	blue	10798497	histone cluster 1, H2bl
Hist1h4b	NM_022686	1.24	18.2	32.0	turq	10795262	histone cluster 1, H4b
Hist2h2ab	NM_001111341	1.20	36.3	10.6	blue	10825146	histone cluster 2, H2ab
Hist2h2bb	ENSRNOT00000042005	1.46	22.2	39.4	turq	10817539	histone cluster 2, H2bb
Hist2h3c2	NM_001107698	1.22	63.2	14.5	blue	10817537	histone cluster 2, H3c2
Hist2h4	NM_001123469	1.53	133	16.7	blue	10825151	histone cluster 2, H4
Hist3h2ba	NM_001111127	1.26	40.7	85.9	turq	10733938	histone cluster 3, H2ba
LOC100361110	XM_002727043	1.21	3.0	49.0	turq	10917992	histone H3.3B-like
LOC365114	ENSRNOT00000018945	1.28	7.7	0.0	grey	10703187	similar to High mobility group protein 1 (HMG-1) (High mobility group protein B1) (Amphoterin
LOC682649	ENSRNOT00000048748	1.24	61.8	14.5	blue	10795265	similar to Histone H2A type 1
LOC690521	ENSRNOT00000042148	1.26	7.6	0.6	ylw	10841240	similar to High mobility group protein 1 (HMG-1) (High mobility group protein B1) (Amphoterin
N5	NM_022857	0.76	-7.8	36.2	turq	10861560	DNA binding protein N5
Phf15	NM_001106998	0.83	-22.4	68.7	turq	10742533	PHD finger protein 15
Rcc2	ENSRNOT00000008940	0.80	-32.6	63.5	turq	10873567	regulator of chromosome condensation 2
Satb2	NM_001109306	0.81	-7.8	1.1	ylw	10928191	SATB homeobox 2
**Golgi Apparatus**							
Rpn2	NM_031698	0.83	-92.2	44.1	turq	10841602	ribophorin II
**Growth Factors**							
Cetn3	ENSRNOT00000021735	1.26	55.0	21.4	blue	10812346	centrin, EF-hand protein, 3 (CDC31 homolog, yeast)
**Cx3cl1**	**NM_134455**	**0.71**	**-210**	**108.9**	**turq**	**10809269**	**chemokine (C-X3-C motif) ligand 1**
Gas2	NM_001127504	1.26	15.2	23.0	turq	10707338	growth arrest-specific 2
Gdf1	NM_001044240	0.81	-25.4	35.7	turq	10791011	growth differentiation factor 1
Hbegf	NM_012945	0.77	-6.2	2.5	ylw	10803947	heparin-binding EGF-like growth factor
Vgf	NM_030997	0.69	-34.2	51.4	turq	10757489	VGF nerve growth factor inducible
**Immune Response**							
Cd34	NM_001107202	0.78	-10.2	1.2	ylw	10766869	CD34 molecule
Cd48	NM_139103	1.38	77.8	70.0	turq	10765634	Cd48 molecule
Siva1	NM_001100982	1.30	7.6	8.5	blue	10887506	SIVA1, apoptosis-inducing factor
**Metabolism & Transport**							
**Pqcp**	**NM_001012470**	**0.79**	**-18.0**	**93.1**	**turq**	**10891322**	**polyglutamine-containing protein**
**Acbd6**	**NM_001011906**	**1.35**	**53.7**	**110.6**	**turq**	**10764814**	**acyl-Coenzyme A binding domain containing 6**
Accn1	NM_001034014	0.81	-24.0	34.8	turq	10745533	amiloride-sensitive cation channel 1, neuronal
Acer3	ENSRNOT00000019987	1.28	28.2	18.0	blue	10723810	alkaline ceramidase 3
Acn9	NM_001047914	1.33	16.8	18.0	blue	10853676	ACN9 homolog (S. cerevisiae)
Agpat9	NM_001025670	0.82	-5.8	0.6	ylw	10775519	1-acylglycerol-3-phosphate O-acyltransferase 9
Atp13a4	ENSRNOT00000002333	1.21	14.4	1.5	ylw	10751803	ATPase type 13A4
Atp1a1	NM_012504	0.83	-173	0.8	brwn	10825472	ATPase, Na+/K+ transporting, alpha 1 polypeptide
Atp1a3	NM_012506	0.78	-338	7.8	brwn	10719728	ATPase, Na+/K+ transporting, alpha 3 polypeptide
Atp2b1	NM_053311	0.81	-310	21.7	blue	10895102	ATPase, Ca++ transporting, plasma membrane 1
Atp2b2	NM_012508	0.79	-91	50.5	turq	10864684	ATPase, Ca++ transporting, plasma membrane 2
Atp5e	NM_139099	1.26	101	20.8	blue	10773636	ATP synthase, H+ transporting, mitochondrial F1 complex, epsilon subunit
Atp5j2	ENSRNOT00000033537	1.23	264	23.3	blue	10756546	ATP synthase, H+ transporting, mitochondrial F0 complex, subunit F2
Atp5l	NM_212516	1.40	17	14.5	blue	10916920	ATP synthase, H+ transporting, mitochondrial F0 complex, subunit G

Atp6v0a1	NM_031604	0.83	-140	48.2	turq	10738237	ATPase, H+ transporting, lysosomal V0 subunit A1
B4galt5	NM_001108608	0.81	-20	32.8	turq	10851952	UDP-Gal:betaGlcNAc beta 1,4-galactosyltransferase, polypeptide 5
Bcat1	NM_017253	0.81	-284	26.0	turq	10866819	branched chain aminotransferase 1, cytosolic
Cacng3	NM_080691	0.77	-85.3	46.8	turq	10710669	calcium channel, voltage-dependent, gamma subunit 3
Car3	NM_019292	1.37	12.8	1.0	ylw	10822242	carbonic anhydrase 3
Car7	NM_001106165	0.82	-6.7	25.1	turq	10809007	carbonic anhydrase 7
Cdipt	NM_138899	0.74	-137	76.4	turq	10710985	CDP-diacylglycerol--inositol 3-phosphatidyltransferase (phosphatidylinositol synthase)
Cept1	NM_001007699	1.22	65.5	11.8	blue	10825796	choline/ethanolamine phosphotransferase 1
Cherp	NM_001106064	0.80	-14.3	0.8	ylw	10787129	calcium homeostasis endoplasmic reticulum protein
Chst1	NM_001011955	0.83	-33.8	39.6	turq	10838039	carbohydrate (keratan sulfate Gal-6) sulfotransferase 1
**Clcn7**	**NM_031568**	**0.83**	**-17.0**	**91.0**	**turq**	**10732302**	**chloride channel 7**
Clns1a	NM_031719	1.23	52.1	51.4	turq	10708824	chloride channel, nucleotide-sensitive, 1A
Clstn1	ENSRNOT00000022100	0.83	-117	2.4	brwn	10874095	calsyntenin 1
Cmbl	NM_001008770	1.20	21.6	5.8	blue	10814105	carboxymethylenebutenolidase homolog (Pseudomonas)
Cmc1	NM_001135259	1.30	4.0	15.8	blue	10920797	COX assembly mitochondrial protein homolog (S. cerevisiae)
Cox15	NM_001033699	0.83	-26.2	4.0	brwn	10715346	COX15 homolog, cytochrome c oxidase assembly protein (yeast)
Cpox	NM_001037095	0.83	-18.1	1.3	ylw	10753574	coproporphyrinogen oxidase
Dagla	NM_001005886	0.74	-37.5	78.4	turq	10728676	diacylglycerol lipase, alpha
Dpysl2	NM_001105717	0.83	-380	4.5	brwn	10784679	dihydropyrimidinase-like 2
Extl3	NM_020097	0.80	-40.6	38.7	turq	10784540	exostoses (multiple)-like 3
Galntl6	NM_001135756	1.28	101	63.4	turq	10787962	UDP-N-acetyl-alpha-D-galactosamine:polypeptide N-acetylgalactosaminyltransferase-like 6
Hck	NM_013185	1.24	2.6	0.0	grey	10840975	hemopoietic cell kinase
Idi1	NM_053539	1.21	27.8	14.3	blue	10799241	isopentenyl-diphosphate delta isomerase 1
Kcna3	NM_019270	0.83	-26.9	0.0	grey	10818239	potassium voltage-gated channel, shaker-related subfamily, member 3
Kcnd2	NM_031730	0.82	-82.5	3.2	blue	10853905	potassium voltage-gated channel, Shal-related subfamily, member 2
Kcnf1	NM_001169104	0.78	-49.0	0.0	grey	10889326	potassium voltage-gated channel, subfamily F, member 1
Kcnj6	NM_013192	0.81	-29.0	40.8	turq	10753269	potassium inwardly-rectifying channel, subfamily J, member 6
Kctd1	ENSRNOT00000061504	0.76	-25.5	32.2	turq	10803294	potassium channel tetramerisation domain containing 1
LOC100188932	NM_001134691	1.20	142	17.9	blue	10883219	dolichyl-diphosphooligosaccharide--protein glycosyltransferase subunit 4
LOC100360692	XM_002728946	1.30	5.7	3.9	blue	10829881	phenazine biosynthesis-like domain-containing protein-like
LOC691427	ENSRNOT00000051411	1.28	96.1	24.1	blue	10708095	similar to 6.8 kDa mitochondrial proteolipid
**Lpcat4**	**NM_001106494**	**0.76**	**-47.3**	**89.0**	**turq**	**10838451**	**lysophosphatidylcholine acyltransferase 4**
Lrp12	NM_001134883	0.81	-32.4	1.0	brwn	10903503	low density lipoprotein-related protein 12
Mcee	NM_001106341	1.24	23.7	25.0	blue	10707721	methylmalonyl CoA epimerase
Mfsd6	NM_001106911	0.81	-29.7	37.2	turq	10923191	major facilitator superfamily domain containing 6
Nat8l	ENSRNOT00000020731	0.75	-48.1	66.5	turq	10777722	N-acetyltransferase 8-like
Ndufa1	NM_001108813	1.28	50.6	9.1	blue	10931991	NADH dehydrogenase (ubiquinone) 1 alpha subcomplex, 1
Ndufa2	NM_001106153	1.54	27.7	59.1	turq	10803995	NADH dehydrogenase (ubiquinone) 1 alpha subcomplex, 2
Ndufa6	NM_001130505	1.30	51.6	22.2	blue	10905716	NADH dehydrogenase (ubiquinone) 1 alpha subcomplex, 6 (B14)
Ndufs5	NM_001030052	1.20	50.5	20.4	blue	10817274	NADH dehydrogenase (ubiquinone) Fe-S protein 5
Nosip	NM_001106260	1.25	12.4	18.2	blue	10706712	nitric oxide synthase interacting protein
Orai2	NM_001170403	0.75	-8.6	0.3	ylw	10757562	ORAI calcium release-activated calcium modulator 2
Panx1	NM_199397	0.81	-7.6	6.3	blue	10915018	Pannexin 1
Pcca	NM_019330	1.26	48.6	76.2	turq	10782166	propionyl-coenzyme A carboxylase, alpha polypeptide
Pigk	NM_001011953	1.23	39.6	86.9	turq	10819816	phosphatidylinositol glycan anchor biosynthesis, class K
Pigt	NM_001106540	0.79	-53.4	72.1	turq	10842130	phosphatidylinositol glycan anchor biosynthesis, class T
Pom121	NM_053622	0.78	-13.6	16.2	blue	10757674	nuclear pore membrane protein 121
Ptgis	NM_031557	0.82	-3.1	10.8	turq	10750966	prostaglandin I2 (prostacyclin) synthase
Ptgs2	NM_017232	0.79	-23.6	0.0	grey	10764551	prostaglandin-endoperoxide synthase 2
Rhbdd2	ENSRNOT00000001962	0.82	-31.7	60.4	turq	10761180	rhomboid domain containing 2
Rtn1	NM_053865	0.79	-442	42.8	turq	10890522	reticulon 1
**Sclt1**	**NM_153740**	**1.32**	**23.8**	**117.1**	**turq**	**10822989**	**sodium channel and clathrin linker 1**
Scn7a	ENSRNOT00000040299	1.27	18.9	6.3	turq	10845945	sodium channel, voltage-gated, type VII, alpha
Sh3bgrl	NM_001173339	1.27	121	66.0	turq	10934669	SH3 domain binding glutamic acid-rich protein like
Slc12a5	NM_134363	0.81	-79.9	63.8	turq	10842254	solute carrier family 12 (potassium-chloride transporter), member 5
Slc1a1	NM_013032	0.82	-49.0	4.4	brwn	10714616	solute carrier family 1 (neuronal/epithelial high affinity glutamate transporter, system Xag)
Slc1a4	NM_198763	0.82	-29.7	54.3	turq	10778620	solute carrier family 1 (glutamate/neutral amino acid transporter), member 4
Slc2a1	NM_138827	0.79	-60.9	42.6	turq	10871521	solute carrier family 2 (facilitated glucose transporter), member 1
Slc30a3	NM_001013243	0.79	-25.3	79.6	turq	10883162	solute carrier family 30 (zinc transporter), member 3
Slc32a1	NM_031782	0.79	-34.0	4.1	brwn	10841774	solute carrier family 32 (GABA vesicular transporter), member 1
Slc38a2	NM_181090	0.80	-144	10.5	blue	10906608	solute carrier family 38, member 2
Slc43a2	NM_001105812	0.72	-35.5	85.9	turq	10735959	solute carrier family 43, member 2
Slc4a8	NM_199497	0.83	-14.2	24.0	blue	10899278	solute carrier family 4 (anion exchanger), member 8
Slc6a6	NM_017206	0.77	-53.6	15.2	blue	10857314	solute carrier family 6 (neurotransmitter transporter, taurine), member 6
Slc7a5	NM_017353	0.74	-46.7	24.6	turq	10811531	solute carrier family 7 (cationic amino acid transporter, y+ system), member 5
Slc7a8	NM_053442	0.77	-32.2	11.7	blue	10783648	solute carrier family 7 (cationic amino acid transporter, y+ system), member 8
Slc8a2	NM_078619	0.81	-36.2	72.7	turq	10704437	solute carrier family 8 (sodium/calcium exchanger), member 2
St8sia5	NM_213628	0.76	-32.2	63.4	turq	10802795	ST8 alpha-N-acetyl-neuraminide alpha-2,8-sialyltransferase 5
Sulf2	NM_001034927	0.79	-35.1	44.4	turq	10851839	sulfatase 2

Syt1	NM_001033680	0.81	-300	72.8	turq	10902232	synaptotagmin I
**Timm8b**	**NM_022541**	**1.28**	**202**	**29.9**	**blue**	**10909883**	**translocase of inner mitochondrial membrane 8 homolog b (yeast)**
**Tomm7**	**NM_001135174**	**1.22**	**106**	**30.6**	**blue**	**10878270**	**translocase of outer mitochondrial membrane 7 homolog (yeast)**
**Proteolysis**							
Adamts19	ENSRNOT00000026543	1.22	7.5	67.3	turq	10801960	ADAM metallopeptidase with thrombospondin type 1 motif, 19
Capn7	NM_001030037	1.26	51.3	86.9	turq	10786680	calpain 7
Cst3	NM_012837	1.20	478	8.0	blue	10850563	cystatin C
**Dcun1d1**	**NM_001107668**	**1.23**	**83**	**92.8**	**turq**	**10822792**	**DCN1, defective in cullin neddylation 1, domain containing 1 (S. cerevisiae)**
LOC679835	ENSRNOT00000044960	1.27	3.6	0.1	ylw	10862194	similar to Anionic trypsin II precursor (Pretrypsinogen II)
Prss22	NM_001106984	1.41	11.3	3.5	blue	10740952	protease, serine, 22
Psma3	NM_017280	1.22	81.1	22.7	blue	10745271	proteasome (prosome, macropain) subunit, alpha type 3
Psma6	NM_017283	1.21	71.5	26.7	blue	10884656	proteasome (prosome, macropain) subunit, alpha type 6
Psmb4	NM_031629	1.28	272	25.5	blue	10824884	proteasome (prosome, macropain) subunit, beta type 4
Psmd10	NM_053925	1.31	24.1	18.9	blue	10822092	proteasome (prosome, macropain) 26S subunit, non-ATPase, 10
Psme2	NM_017257	1.20	42.5	4.4	brwn	10783805	proteasome (prosome, macropain) activator subunit 2
RGD1565317	ENSRNOT00000017230	1.34	21.0	12.7	blue	10736332	similar to ubiquitin-like/S30 ribosomal fusion protein
Rnf187	NM_001164264	0.81	-94.2	80.3	turq	10742983	ring finger protein 187
Rnf26	NM_001113748	0.71	-30.2	76.1	turq	10916664	ring finger protein 26
Rnf44	NM_001024795	0.80	-16.1	22.2	turq	10794146	ring finger protein 44
Ube2cbp	NM_001039610	1.23	8.2	66.1	turq	10919076	ubiquitin-conjugating enzyme E2C binding protein
Ube2v2	NM_183052	1.41	53.0	9.8	blue	10752621	ubiquitin-conjugating enzyme E2 variant 2
Usp42	NM_001105909	0.82	-14.5	1.2	ylw	10756704	ubiquitin specific peptidase 42
**Receptors & Binding Proteins**							
Abca3	BC088202	0.81	-16.4	8.0	blue	10732021	ATP-binding cassette, sub-family A (ABC1), member 3
**Abcc5**	**NM_053924**	**0.76**	**-39.6**	**34.2**	**blue**	**10752148**	**ATP-binding cassette, sub-family C (CFTR/MRP), member 5**
Abcc8	NM_013039	0.79	-8.8	71.6	turq	10722012	ATP-binding cassette, sub-family C (CFTR/MRP), member 8
Acvr1	NM_024486	0.82	-17.5	0.0	grey	10845429	activin A receptor, type I
Adora1	NM_017155	0.83	-39.4	4.3	brwn	10767771	adenosine A1 receptor
**Adra1d**	**NM_024483**	**0.78**	**-10.5**	**3.3**	**ylw**	**10850087**	**adrenergic, alpha-1D-, receptor**
Alb	NM_134326	0.57	-15.6	5.6	blue	10775968	albumin
Apbb2	XM_001077874	0.80	-60.8	45.9	turq	10772653	amyloid beta (A4) precursor protein-binding, family B, member 2
Cckbr	NM_013165	0.83	-15.8	2.2	ylw	10709565	cholecystokinin B receptor
**Chrm1**	**NM_080773**	**0.73**	**-72.6**	**101.5**	**turq**	**10713581**	**cholinergic receptor, muscarinic 1**
Grm1	NM_001114330	0.77	-30.8	18.4	blue	10716789	glutamate receptor, metabotropic 1
Htr1b	NM_022225	0.82	-15.6	51.3	turq	10918979	5-hydroxytryptamine (serotonin) receptor 1B
Il17ra	NM_001107883	0.83	-5.3	2.1	blue	10858315	interleukin 17 receptor A
Il7r	NM_001106418	1.32	7.2	0.9	ylw	10821851	interleukin 7 receptor
Ldlrap1	NM_001109271	0.78	-8.9	6.0	brwn	10880552	low density lipoprotein receptor adaptor protein 1
Leng4	NM_001134978	0.72	-72.6	63.4	turq	10703715	leukocyte receptor cluster (LRC) member 4
Nrsn2	NM_001109561	0.77	-75.5	2.6	ylw	10850783	neurensin 2
Nrxn3	NM_053817	0.83	-66.2	25.8	blue	10886212	neurexin 3
Olr1425	NM_001000010	1.23	2.4	19.4	turq	10742940	olfactory receptor 1425
Osbp2	NM_001107232	0.78	-20.0	19.6	turq	10778001	oxysterol binding protein 2
Osbpl10	ENSRNOT00000064852	0.83	-11.2	30.5	turq	10914025	oxysterol binding protein-like 10
Osbpl3	ENSRNOT00000013828	0.76	-10.9	8.3	blue	10862473	oxysterol binding protein-like 3
**Pnpla6**	**ENSRNOT00000001298**	**0.80**	**-14.5**	**8.4**	**brwn**	**10759662**	**patatin-like phospholipase domain containing 6**
Prrt1	NM_001032285	0.79	-16.1	42.1	turq	10831447	proline-rich transmembrane protein 1
Prrt3	NM_001109350	0.74	-14.9	65.9	turq	10864658	proline-rich transmembrane protein 3
Pvrl1	ENSRNOT00000008614	0.78	-20.5	13.4	turq	10909394	poliovirus receptor-related 1
Rtn4r	NM_053613	0.74	-27.2	54.5	turq	10755619	reticulon 4 receptor
Sidt1	NM_001100653	0.78	-43.9	65.9	turq	10751136	SID1 transmembrane family, member 1
Sidt2	NM_001108142	0.81	-42.2	67.2	turq	10917040	SID1 transmembrane family, member 2
Sstr4	NM_013036	0.81	-14.2	1.0	brwn	10840565	somatostatin receptor 4
Stambp	NM_138531	0.80	-20.7	13.8	turq	10863559	Stam binding protein
Stxbp1	NM_013038	0.82	-170	2.4	brwn	10844390	syntaxin binding protein 1
Tmem151a	NM_001107570	0.79	-24.6	25.2	blue	10727725	transmembrane protein 151A
Vom2r66	ENSRNOT00000051621	1.42	51.5	65.0	turq	10774903	vomeronasal 2 receptor, 66
Wipf3	NM_147211	0.82	-11.9	1.5	blue	10855637	WAS/WASL interacting protein family, member 3
**Signaling**							
Abl2	NM_001107186	0.80	-20.3	24.8	blue	10764852	v-abl Abelson murine leukemia viral oncogene homolog 2 (arg, Abelson-related gene)
Adcy5	NM_022600	0.78	-44.7	75.3	turq	10754482	adenylate cyclase 5
Arc	NM_019361	0.72	-68.6	31.9	turq	10904511	activity-regulated cytoskeleton-associated protein
Arhgap23	ENSRNOT00000055414	0.79	-14.5	5.1	brwn	10737838	Rho GTPase activating protein 23
Arhgef2	NM_001012079	0.82	-27.1	2.2	ylw	10816566	rho/rac guanine nucleotide exchange factor (GEF) 2
Avpi1	NM_134373	1.21	6.4	0.0	grey	10715452	arginine vasopressin-induced 1
Bccip	NM_001108505	1.26	39.4	13.0	blue	10711769	BRCA2 and CDKN1A interacting protein
Btbd12	ENSRNOT00000033919	0.83	-7.4	4.0	brwn	10731872	BTB (POZ) domain containing 12
Camk2b	NM_001042354	0.83	-77.5	66.8	turq	10778268	calcium/calmodulin-dependent protein kinase II beta
Cks1b	NM_001135749	1.60	10.6	10.0	blue	10878032	CDC28 protein kinase regulatory subunit 1B

Cmip	NM_001163273	0.82	-50.7	62.8	turq	10808249	c-Maf-inducing protein
Dbi	NM_031853	1.29	40.4	26.0	turq	10931154	diazepam binding inhibitor (GABA receptor modulator, acyl-Coenzyme A binding protein)
Defb49	NM_001037527	0.82	-3.2	1.5	ylw	10926837	defensin beta 49
Dusp1	NM_053769	0.81	-25.2	0.9	brwn	10732652	dual specificity phosphatase 1
Dusp7	ENSRNOT00000014770	0.80	-71	45.7	turq	10912837	dual specificity phosphatase 7
**Fkbp3**	**NM_001106736**	**1.25**	**144**	**31.3**	**blue**	**10890182**	**FK506 binding protein 3**
Gng3	NM_053658	0.81	-118			10728555	guanine nucleotide binding protein (G protein), gamma 3
Gng5	NM_024377	1.22	25.9	0.0	grey	10750808	guanine nucleotide binding protein (G protein), gamma 5
Gpr34	NM_001024925	1.25	42.2	35.4	turq	10936742	G protein-coupled receptor 34
Gpr63	NM_001106640	0.83	-16.9	1.0	ylw	10867948	G protein-coupled receptor 63
Gpr88	NM_031696	0.73	-10.0	5.1	turq	10826261	G-protein coupled receptor 88
Ifi27	NM_203410	1.33	59.9	7.3	blue	10886573	interferon, alpha-inducible protein 27
**Inpp5j**	**NM_133562**	**0.78**	**-15.8**	**89.4**	**turq**	**10777958**	**inositol polyphosphate-5-phosphatase J**
LOC500584	ENSRNOT00000051887	1.24	34.2	50.1	turq	10881461	similar to casein kinase 1, gamma 3 isoform 2
LOC686547	ENSRNOT00000064942	1.21	7.3	48.6	turq	10785618	similar to TBC1 domain family member 4 (Akt substrate of 160 kDa) (AS160)
**Magmas**	**NM_001100136**	**1.25**	**31.4**	**30.1**	**blue**	**10738705**	**mitochondria-associated protein involved in granulocyte-macrophage colony-stimulating**
Mark2	NM_021699	0.81	-35.1	77.4	turq	10728396	MAP/microtubule affinity-regulating kinase 2
Mbrl	NM_001100886	0.83	-38.6	68.3	turq	10893961	membralin
**Memo1**	**NM_001029917**	**1.37**	**53.7**	**104.6**	**turq**	**10882920**	**mediator of cell motility 1**
Mkln1	NM_031359	1.21	157	80.8	turq	10854338	muskelin 1, intracellular mediator containing kelch motifs
Ncln	NM_001014082	0.80	-16.7	70.0	turq	10893484	nicalin homolog (zebrafish)
Pde3b	NM_017229	1.28	39.1	72.3	turq	10710089	phosphodiesterase 3B, cGMP-inhibited
Pde7b	NM_080894	0.74	-33.2	3.5	brwn	10717069	phosphodiesterase 7B
Peli3	NM_001127542	0.83	-12.1	4.3	brwn	10727710	pellino 3
Plk3	NM_022187	0.81	-4.6	23.7	turq	10878938	polo-like kinase 3 (Drosophila)
Ppme1	ENSRNOT00000023648	0.82	-53.1	73.4	turq	10723956	protein phosphatase methylesterase 1
Ppp2r3c	NM_001014196	1.28	29.2	12.9	blue	10890003	protein phosphatase 2, regulatory subunit B'', gamma
Pragmin	NM_001107315	0.66	-24.8	11.6	blue	10791989	pragma of Rnd2
**Prkra**	**NM_001024780**	**1.23**	**12.9**	**2.7**	**ylw**	**10846331**	**protein kinase, interferon inducible double stranded RNA dependent activator**
Prosapip1	NM_172022	0.82	-36.8	62.9	turq	10849927	ProSAPiP1 protein
Ptprn2	NM_031600	0.82	-57.3	57.1	turq	10887746	protein tyrosine phosphatase, receptor type, N polypeptide 2
Rasd1	BC099136	0.73	-25.3	1.9	brwn	10743227	RAS, dexamethasone-induced 1
Rasgrp1	NM_019211	0.83	-162	50.8	turq	10848393	RAS guanyl releasing protein 1 (calcium and DAG-regulated)
RGD1563865	ENSRNOT00000065348	1.21	3.0	7.9	blue	10718188	similar to putative protein kinase
RGD1563970	ENSRNOT00000055203	1.43	3.1	1.4	ylw	10724391	similar to Tripartite motif protein 30-like
Rin1	NM_139038	0.81	-28.1	2.1	brwn	10712986	Ras and Rab interactor 1
Styxl1	NM_001037788	1.20	4.1	0.9	brwn	10757606	serine/threonine/tyrosine interacting-like 1
Trib1	NM_023985	0.80	-7.6	58.2	turq	10896793	tribbles homolog 1 (Drosophila)
**Transcription**							
Asb15	BC097405	0.77	-3.3	1.2	brwn	10853995	ankyrin repeat and SOCS box-containing protein 15
Bcl11a	BC086607	0.79	-34.7	49.3	turq	10774596	B-cell CLL/lymphoma 11A (zinc finger protein)
Bcl9	NM_001107703	0.80	-25.2	1.2	brwn	10825228	B-cell CLL/lymphoma 9
Ccdc132	NM_001173511	1.26	62.7	16.5	blue	10853522	coiled-coil domain containing 132
Ccdc46	NM_001105849	1.43	22.9	86.3	turq	10739277	coiled-coil domain containing 46
Ccdc53	NM_001106776	1.36	15.6	86.3	turq	10894722	coiled-coil domain containing 53
Ccdc92	NM_001083898	0.83	-21.4	1.3	brwn	10758208	coiled-coil domain containing 92
Chchd1	NM_001108369	1.32	32.4	27.0	blue	10782314	coiled-coil-helix-coiled-coil-helix domain containing 1
Dbc1	NM_080482	0.82	-86.0	39.8	turq	10877573	deleted in bladder cancer 1 (human)
Dexi	NM_001109026	0.82	-36.1	40.5	turq	10731514	dexamethasone-induced transcript
Dnajb5	NM_001108004	0.78	-45.5	85.3	turq	10868428	DnaJ (Hsp40) homolog, subfamily B, member 5
**Ets2**	**NM_001107107**	**0.82**	**-16.8**	**2.8**	**ylw**	**10750460**	**v-ets erythroblastosis virus E26 oncogene homolog 2 (avian)**
Etv5	NM_001107082	0.77	-18.5	69.0	turq	10752034	ets variant 5
Foxg1	NM_012560	0.82	-43.4	13.4	blue	10884478	forkhead box G1
Foxp2	ENSRNOT00000007759	0.82	-5.5	6.4	blue	10853764	forkhead box P2
Gatad2a	NM_001013881	0.82	-17.2	0.5	ylw	10787666	GATA zinc finger domain containing 2A
Hexim1	NM_001025136	0.82	-57.4	2.7	brwn	10738671	hexamethylene bis-acetamide inducible 1
**Hip1r**	**NM_001134763**	**0.82**	**-7.7**	**8.3**	**brwn**	**10761758**	**huntingtin interacting protein 1 related**
Kbtbd11	NM_001107326	0.80	-11.8	4.2	brwn	10792660	kelch repeat and BTB (POZ) domain containing 11
Klf13	NM_001109147	0.78	-20.4	42.6	turq	10722549	Kruppel-like factor 13
Klhl18	ENSRNOT00000056141	0.78	-13.0	24.3	turq	10920449	kelch-like 18 (Drosophila)
Lhfpl4	NM_181387	0.83	-30.8	32.7	turq	10864616	lipoma HMGIC fusion partner-like 4
LOC499124	NM_001100991	0.81	-12.1	1.9	brwn	10720539	mouse zinc finger protein 14-like
LOC680767	ENSRNOT00000060096	1.27	52.8	1.3	brwn	10760816	similar to transcription elongation factor B (SIII), polypeptide 1
LOC683241	XM_001065806	1.39	22.2	38.5	turq	10708984	similar to Finkel-Biskis-Reilly murine sarcoma virusubiquitously expressed
LOC683302	XM_001065309	1.21	18.5	0.7	ylw	10787048	similar to tumor protein, translationally-controlled 1
LOC687439	ENSRNOT00000048726	1.21	6.8	84.8	turq	10725043	similar to Finkel-Biskis-Reilly murine sarcoma virusubiquitously expressed
Lztfl1	NM_001024266	1.25	52.2	20.6	turq	10921131	leucine zipper transcription factor-like 1
Magoh	NM_001100536	1.27	28.3	12.6	blue	10870733	mago-nashi homolog, proliferation-associated (Drosophila)
Med13l	NM_001108337	0.81	-27.9	70.3	turq	10762389	mediator complex subunit 13-like

Med21	NM_001107895	1.28	25.4	18.8	blue	10859655	mediator complex subunit 21
**Med24**	**NM_001034079**	**0.81**	**-31.3**	**94.3**	**turq**	**10746923**	**mediator complex subunit 24**
Med31	NM_001135813	1.28	40.3	12.3	blue	10744682	mediator complex subunit 31
Mef2c	ENSRNOT00000041296	0.82	-89.2	38.5	turq	10820223	myocyte enhancer factor 2C
**Mll1**	**NM_001108139**	**0.78**	**-59.1**	**33.8**	**blue**	**10916880**	**myeloid/lymphoid or mixed-lineage leukemia 1**
Mll2	ENSRNOT00000040635	0.81	-21.4	17.4	blue	10906977	myeloid/lymphoid or mixed-lineage leukemia 2
Mllt11	NM_001013912	0.83	-83.9	52.4	turq	10824965	myeloid/lymphoid or mixed-lineage leukemia (trithorax homolog, Drosophila); translocated
Mlxip	ENSRNOT00000001690	0.79	-8.9	4.1	blue	10761897	MLX interacting protein
Mpp3	ENSRNOT00000028264	0.81	-13.6	1.8	brwn	10747697	membrane protein, palmitoylated 3 (MAGUK p55 subfamily member 3)
Ndrg1	NM_001011991	0.73	-64.1	4.6	brwn	10904169	N-myc downstream regulated gene 1
Nol8	NM_001108408	1.24	15.9	42.2	turq	10797631	nucleolar protein 8
Npas4	NM_153626	0.75	-12.1	71.4	turq	10727717	neuronal PAS domain protein 4
Nr4a1	NM_024388	0.66	-51.7	68.5	turq	10899387	nuclear receptor subfamily 4, group A, member 1
Nsbp1	NM_001134706	1.25	7.4	0.0	grey	10939063	nucleosomal binding protein 1
Nxt2	NM_001108120	1.23	6.5	81.8	turq	10937391	nuclear transport factor 2-like export factor 2
**Pnrc2**	**NM_001103360**	**1.26**	**71.6**	**89.1**	**turq**	**10880643**	**proline-rich nuclear receptor coactivator 2**
Rala	NM_031093	1.21	60.8	49.2	turq	10795422	v-ral simian leukemia viral oncogene homolog A (ras related)
RGD1566052	BC158667	1.23	19.4	70.5	turq	10847986	similar to elongation protein 4 homolog
Serf1	ENSRNOT00000024291	1.26	19.2	18.5	blue	10812734	small EDRK-rich factor 1
Sertad2	NM_001024903	0.79	-11.1	2.4	brwn	10774369	SERTA domain containing 2
Smarcd1	NM_001108752	0.81	-36.1	48.3	turq	10899174	SWI/SNF related, matrix associated, actin dependent regulator of chromatin, subfamily d
**Stx16**	**NM_001108610**	**0.82**	**-31.1**	**99.7**	**turq**	**10842635**	**syntaxin 16**
Tbr1	ENSRNOT00000065340	0.81	-32.6	29.2	turq	10845628	T-box, brain, 1
Zbtb39	NM_001130537	0.81	-9.3	21.3	turq	10895988	zinc finger and BTB domain containing 39
Zbtb45	NM_001107478	0.82	-5.3	30.3	turq	10704281	zinc finger and BTB domain containing 45
Zcchc14	ENSRNOT00000058486	0.83	-9.3	0.3	ylw	10811503	zinc finger, CCHC domain containing 14
Zdhhc22	NM_001039325	0.76	-30.9	2.0	ylw	10891324	zinc finger, DHHC-type containing 22
Zdhhc23	NM_213627	0.82	-11.1	1.1	ylw	10751190	zinc finger, DHHC-type containing 23
Zdhhc5	NM_001039338	0.82	-38.9	2.5	ylw	10846832	zinc finger, DHHC-type containing 5
Zfhx2	NM_001098803	0.82	-6.9	26.8	turq	10783759	zinc finger homeobox 2
Zfp35	NM_001013141	1.31	32.1	19.9	blue	10800493	zinc finger protein 35
Zfp462	ENSRNOT00000047225	0.83	-25.4	29.8	turq	10869158	zinc finger protein 462
Zfp709l1	ENSRNOT00000042498	1.27	93.1	82.3	turq	10893420	zinc finger protein 709-like 1
Znf513	NM_001012110	0.81	-6.6	27.6	turq	10883098	zinc finger protein 513
Zswim1	ENSRNOT00000021070	0.82	-13.9	48.3	turq	10842200	zinc finger, SWIM-type containing 1
**Translation & Protein Modification**							
Bat2l	NM_001134518	0.83	-87.0	55.6	turq	10835501	HLA-B associated transcript 2-like
Clp1	NM_001009599	1.21	17.9	0.8	ylw	10846846	CLP1, cleavage and polyadenylation factor I subunit, homolog (S. cerevisiae)
**Eef1b2**	**NM_001108799**	**1.22**	**33.9**	**34.0**	**blue**	**10923857**	**eukaryotic translation elongation factor 1 beta 2**
Eif3e	NM_001011990	1.27	118	28.5	turq	10903545	eukaryotic translation initiation factor 3, subunit E
Eif3m	NM_001168523	1.28	105	48.1	turq	10847965	eukaryotic translation initiation factor 3, subunit M
LOC100362998	XM_002729586	1.21	2.5	7.7	brwn	10877581	hypothetical protein LOC100362998
LOC367195	NM_001047920	1.40	15.8	50.8	turq	10921083	similar to 60S RIBOSOMAL PROTEIN L7
LOC499485	XM_002725892	1.22	16.5	14.4	blue	10820047	similar to 60S ribosomal protein L17 (L23) (Amino acid starvation-induced protein) (ASI)
LOC681338	ENSRNOT00000047517	1.31	2.2	18.0	blue	10928032	similar to ribosomal protein L31
LOC684806	ENSRNOT00000051764	1.30	23.4	38.2	turq	10781564	similar to 40S ribosomal protein S29
LOC688136	XM_001081257	1.23	329	86.5	turq	10737445	similar to 60S ribosomal protein L37a
LOC688712	XM_001068022	1.25	8.4	14.2	blue	10810373	similar to ribosomal protein L22 like 1
LOC688981	ENSRNOT00000046585	1.33	6.7	13.3	blue	10890597	similar to 60S ribosomal protein L26 (Silica-induced gene 20 protein) (SIG-20)
Lsm10	NM_001108976	1.20	9.5	2.0	ylw	10872000	LSM10, U7 small nuclear RNA associated
Lsm3	NM_001106611	1.22	41.5	10.1	blue	10857310	LSM3 homolog, U6 small nuclear RNA associated (S. cerevisiae)
Lsm5	NM_001107289	1.31	10.0	1.1	ylw	10862728	LSM5 homolog, U6 small nuclear RNA associated (S. cerevisiae)
**Mrpl1**	**NM_001105997**	**1.23**	**16.2**	**106.2**	**turq**	**10775719**	**mitochondrial ribosomal protein L1**
Mrpl23	NM_022529	1.24	34.6	10.8	blue	10712501	mitochondrial ribosomal protein L23
Mrpl30	NM_001106903	1.21	16.4	11.1	blue	10922695	mitochondrial ribosomal protein L30
Mrpl36	NM_001108879	1.29	29.7	26.4	blue	10796835	mitochondrial ribosomal protein L36
Mrpl42	NM_001106782	1.29	175	20.0	blue	10901999	mitochondrial ribosomal protein L42
Mrps18c	NM_001105996	1.26	135	58.0	turq	10775532	mitochondrial ribosomal protein S18C
Parp9	NM_001103351	1.22	4.6	2.4	ylw	10754426	poly (ADP-ribose) polymerase family, member 9
Pfdn1	NM_001108427	1.32	46.7	27.2	blue	10803943	prefoldin subunit 1
Pfdn5	NM_001106794	1.43	102	27.8	blue	10899531	prefoldin subunit 5
**Rbm4**	**ENSRNOT00000026621**	**0.82**	**-19.5**	**111.0**	**turq**	**10727643**	**RNA binding motif protein 4**
RGD1310899	AB256045	1.21	29.3	27.2	blue	10885962	similar to CGI-35 protein
**RGD1561984**	**ENSRNOT00000041555**	**1.23**	**25.0**	**9.2**	**brwn**	**10917299**	**similar to ribosomal protein L27a**
RGD1562547	ENSRNOT00000049211	1.29	5.5	60.6	turq	10901960	similar to ribosomal protein L31
RGD1563835	ENSRNOT00000042756	1.58	16.7	13.3	blue	10751237	similar to ribosomal protein L27
RGD1564552	ENSRNOT00000050803	1.23	26.5	4.0	brwn	10799888	similar to ribosomal protein L21
**RGD1564617**	**ENSRNOT00000043603**	**1.32**	**8.3**	**107.5**	**turq**	**10704198**	**similar to large subunit ribosomal protein L36a**
RGD1564849	ENSRNOT00000040519	1.20	2.3	19.6	turq	10830620	similar to 60S ribosomal protein L37a

RGD1565054	ENSRNOT00000034714	1.33	113	81.2	turq	10938483	similar to 60S acidic ribosomal protein P1
RGD1565131	ENSRNOT00000038919	1.24	43.5	15.9	turq	10756617	similar to ribosomal protein L15
RGD1565170	ENSRNOT00000043836	1.37	12.6	34.6	turq	10780763	similar to 60S ribosomal protein L23a
Rpl10a	NM_031065	1.21	40.8	33.0	turq	10890299	ribosomal protein L10A
**Rpl22**	**NM_031104**	**1.31**	**55.9**	**45.0**	**turq**	**10863772**	**ribosomal protein L22**
Rpl22l1	NM_001108548	1.43	50.7	26.5	blue	10814650	ribosomal protein L22 like 1
Rpl24	NM_022515	1.24	38.8	80.9	turq	10753732	ribosomal protein L24
**Rpl26**	**NM_001105788**	**1.48**	**327**	**102.4**	**turq**	**10717434**	**ribosomal protein L26**
Rpl27a	NM_001106290	1.24	97	41.7	turq	10706219	ribosomal protein L27a
**Rpl31**	**NM_022506**	**1.25**	**233**	**114.8**	**turq**	**10853663**	**ribosomal protein L31**
**Rpl35a**	**NM_021264**	**1.26**	**109**	**122.7**	**turq**	**10797473**	**ribosomal protein L35a**
**Rpl36**	**NM_022504**	**1.23**	**45.9**	**31.8**	**blue**	**10931410**	**ribosomal protein L36**
**Rpl36a**	**NM_001128065**	**1.32**	**301**	**105.4**	**turq**	**10735315**	**ribosomal protein L36a**
Rpl36al	NM_031105	1.29	46	48.1	turq	10890243	ribosomal protein L36a-like
**Rpl37**	**NM_031106**	**1.22**	**244**	**119.3**	**turq**	**10736053**	**ribosomal protein L37**
**Rpl39**	**NM_012875**	**1.44**	**145**	**104.7**	**turq**	**10782917**	**ribosomal protein L39**
Rpl41	NM_139083	1.43	269	59.4	turq	10774894	ribosomal protein L41
Rpl5	NM_031099	1.22	155	120.0	turq	10930534	ribosomal protein L5
Rps14	NM_022672	1.40	183	78.2	turq	10802007	ribosomal protein S14
Rps15	NM_017151	1.32	60	19.2	blue	10900533	ribosomal protein S15
Rps15a	NM_053982	1.35	128	70.3	turq	10725159	ribosomal protein S15a
Rps17	NM_017152	1.31	91.0	80.9	turq	10844946	ribosomal protein S17
Rps18	NM_213557	1.21	45.0	32.7	turq	10749058	ribosomal protein S18
**Rps19**	**NM_001037346**	**1.32**	**53.5**	**119.0**	**turq**	**10778918**	**ribosomal protein S19**
Rps20	NM_001007603	1.31	285	121.2	turq	10871337	ribosomal protein S20
Rps21	NM_031111	1.34	194	121.1	turq	10909758	ribosomal protein S21
**Rps24**	**NM_031112**	**1.46**	**33.1**	**106.1**	**turq**	**10786132**	**ribosomal protein S24**
Rps25	NM_001005528	1.36	127	106.8	turq	10863900	ribosomal protein s25
**Rps27a**	**NM_031113**	**1.38**	**104**	**106.5**	**turq**	**10803676**	**ribosomal protein S27a**
**Rps7**	**NM_031570**	**1.22**	**377**	**102.2**	**turq**	**10785933**	**ribosomal protein S7**
**Sf1**	**NM_001110793**	**0.78**	**-37.2**	**9.5**	**brwn**	**10713362**	**splicing factor 1**
Snrnp25	ENSRNOT00000067079	1.29	10.4	6.9	brwn	10741793	small nuclear ribonucleoprotein 25 (U11/U12)
Snrnp35	NM_001014127	0.81	-9.7	2.0	ylw	10761724	small nuclear ribonucleoprotein 35 (U11/U12)
Thyn1	NM_001007661	1.33	30.5	23.1	blue	10921175	thymocyte nuclear protein 1
**Miscellaneous & Unknown**							
**Bxdc1**	**NM_001106391**	**1.37**	**39.6**	**97.4**	**turq**	**10833643**	**brix domain containing 1**
Fam111a	NM_001109163	1.26	4.3	1.7	ylw	10714106	family with sequence similarity 111, member A
Fam122a	BC078922	0.79	-15.7	37.9	turq	10729460	family with sequence similarity 122A
Fam163b	NM_001109458	0.77	-52.6	21.5	turq	10843935	family with sequence similarity 163, member B
Fam57b	NM_001106296	0.76	-12.0	2.0	ylw	10710930	family with sequence similarity 57, member B
Fam96a	NM_001008327	1.22	40.9	14.0	blue	10911042	family with sequence similarity 96, member A
Ift74	NM_001007001	1.43	37.8	15.9	blue	10869925	intraflagellar transport 74 homolog (Chlamydomonas)
**LOC100125362**	**NM_001103354**	**0.76**	**-61.1**	**97.5**	**turq**	**10917361**	**hypothetical protein LOC100125362**
LOC100188933	NM_001134695	1.26	4.7	9.8	blue	10833598	hypothetical protein LOC100188933
LOC362526	NM_001025021	1.29	5.6	22.6	turq	10869244	hypothetical protein LOC362526
**LOC500420**	**BC089864**	**1.24**	**57.9**	**29.7**	**blue**	**10875751**	**similar to CG12279-PA**
**LOC682679**	**ENSRNOT00000054682**	**1.28**	**20.4**	**30.7**	**blue**	**10730895**	**hypothetical protein LOC682679**
LOC686031	NM_001109490	1.35	25.2	0.8	ylw	10937658	hypothetical protein LOC686031
LOC689165	ENSRNOT00000034542	0.79	-16.5	24.5	turq	10794257	hypothetical protein LOC689165
Lsmd1	NM_001105794	1.22	25.5	5.0	brwn	10708152	LSM domain containing 1
**RGD1307722**	**NM_001108730**	**0.82**	**-17.0**	**3.1**	**ylw**	**10900201**	**similar to hypothetical protein MGC20700**
RGD1308616	ENSRNOT00000039235	0.74	-14.7	76.8	turq	10879227	similar to KIAA0467 protein
RGD1309310	ENSRNOT00000005684	0.75	-77.1	67.2	turq	10739595	similar to mKIAA0195 protein
RGD1309414	ENSRNOT00000063831	0.74	-13.4	20.8	blue	10782284	similar to KIAA0913 protein
Samd9l	ENSRNOT00000013044	1.37	61.1	74.7	turq	10860812	sterile alpha motif domain containing 9-like
Tmem108	ENSRNOT00000014519	0.83	-7.0	8.4	blue	10919694	transmembrane protein 108
Tmem130	NM_001170399	0.80	-185	71.4	turq	10756597	transmembrane protein 130
Tmem175	NM_001013991	0.79	-35.1	30.8	turq	10775016	transmembrane protein 175
Tmem178	NM_001004282	0.82	-54.8	1.2	ylw	10882525	transmembrane protein 178
Tmem184b	NM_001173370	0.80	-21.8	45.3	turq	10905427	transmembrane protein 184B
Tmem205	NM_001106804	1.25	25.7	12.8	blue	10915657	transmembrane protein 205
Tmem39b	NM_001014192	0.80	-7.6	24.3	blue	10880047	transmembrane protein 39b
Tmem69	NM_001035001	1.20	31.6	6.5	blue	10878875	transmembrane protein 69
Tmem8b	ENSRNOT00000021227	0.80	-10.4	23.2	turq	10868577	transmembrane protein 8B
Trim21	NM_001082572	1.24	4.4	5.4	brwn	10724219	tripartite motif-containing 21
**Trim9**	**NM_130420**	**0.80**	**-62.4**	**29.7**	**blue**	**10890463**	**tripartite motif-containing 9**
Ttc3	NM_001108315	1.27	232	81.5	turq	10750426	tetratricopeptide repeat domain 3
Ttc35	NM_001113785	1.21	122	23.7	blue	10896380	tetratricopeptide repeat domain 35
Ttyh3	NM_001107124	0.82	-48.9	17.4	turq	10756931	tweety homolog 3 (Drosophila)

**Otud6b**	**NM_001106639**	**1.22**	**44.7**	**90.7**	**turq**	**10875610**	**OTU domain containing 6B**
Pnmal2	NM_001107481	0.80	-85.4	15.4	blue	10704571	PNMA-like 2
RGD1306595	NM_001025626	1.22	7.4	13.1	blue	10745492	similar to hypothetical protein
RGD1306991	NM_001014183	0.64	-139	65.1	turq	10840226	similar to Protein C20orf103 precursor
RGD1307966	ENSRNOT00000028563	0.80	-25.6	88.8	turq	10747847	similar to hypothetical protein
RGD1310819	ENSRNOT00000051376	0.81	-35.5	35.9	turq	10927477	similar to putative protein (5S487)
RGD1564195	ENSRNOT00000049044	0.79	-27.6	66.8	turq	10725914	similar to hypothetical protein
**ESTs**							
	**ENSRNOT00000063556**	**0.75**	**-10.9**	**3.0**	**ylw**	**10913940**	
	**---**	**1.47**	**155**	**2.9**	**ylw**	**10820008**	
	**ENSRNOT00000053339**	**2.02**	**17.7**	**2.8**	**ylw**	**10813885**	
	ENSRNOT00000035662	1.22	4.5	2.7	ylw	10933574	
	---	1.20	5.7	2.4	ylw	10875100	
	ENSRNOT00000054170	1.69	25.7	2.3	ylw	10817543	
	ENSRNOT00000057059	0.80	-3.9	2.1	ylw	10824111	
	ENSRNOT00000052661	1.21	207	1.6	ylw	10722441	
	ENSRNOT00000053417	1.42	4.2	1.5	ylw	10765986	
	ENSRNOT00000042547	1.31	9.5	1.2	ylw	10766057	
	---	0.80	-2.5	1.1	ylw	10920741	
	ENSRNOT00000052854	0.80	-10.7	1.0	ylw	10932269	
	ENSRNOT00000014607	1.29	8.4	0.9	ylw	10934978	
	GENSCAN00000021698	0.83	-3.8	0.8	ylw	10830199	
RGD1561277	ENSRNOT00000039449	1.20	5.4	0.7	ylw	10733920	RGD1561277
	---	1.24	1.9	0.4	ylw	10771002	
LOC363737	ENSRNOT00000032643	0.80	-2.4	0.3	ylw	10750089	hypothetical LOC363737
	ENSRNOT00000053880	1.23	38.4	0.2	ylw	10722459	
	**ENSRNOT00000054468**	**0.73**	**-4.6**	**119.5**	**turq**	**10759475**	
	**ENSRNOT00000052698**	**1.36**	**5.6**	**115.2**	**turq**	**10731138**	
	**NM_001105954**	**1.52**	**78.5**	**111.0**	**turq**	**10764376**	
	**ENSRNOT00000052727**	**1.46**	**27.4**	**100.4**	**turq**	**10910764**	
**RGD1562608**	**NM_001134607**	**1.32**	**68.4**	**100.1**	**turq**	**10803536**	**similar to KIAA1328 protein**
	**ENSRNOT00000038403**	**1.35**	**6.9**	**99.0**	**turq**	**10780735**	
	**ENSRNOT00000054485**	**1.26**	**9.0**	**96.4**	**turq**	**10764361**	
	K00780	1.54	38.2	92.8	turq	10737194	
	ENSRNOT00000053805	1.32	49.0	88.8	turq	10714346	
	ENSRNOT00000054624	1.26	24.8	88.7	turq	10905605	
	ENSRNOT00000054763	1.29	128	87.9	turq	10729629	
	ENSRNOT00000067839	1.33	38.7	87.0	turq	10896177	
	ENSRNOT00000016461	1.35	10.7	86.7	turq	10722816	
	ENSRNOT00000062230	1.43	36.4	86.0	turq	10892067	
	ENSRNOT00000004332	1.35	191	85.8	turq	10768301	
	ENSRNOT00000053811	1.30	118	85.2	turq	10914312	
	ENSRNOT00000052645	1.51	10.6	84.1	turq	10839168	
	ENSRNOT00000053552	0.76	-32.2	83.4	turq	10713608	
	ENSRNOT00000060806	0.79	-26.3	82.7	turq	10764005	
	ENSRNOT00000009569	1.37	75.0	82.7	turq	10856024	
	ENSRNOT00000052882	0.77	-29.9	82.7	turq	10759461	
	ENSRNOT00000052620	0.78	-25.4	82.3	turq	10828077	
	ENSRNOT00000053276	0.76	-93.4	79.9	turq	10909358	
	ENSRNOT00000038531	1.27	45.8	79.3	turq	10774930	
	ENSRNOT00000053968	1.50	47.8	79.3	turq	10914314	
	ENSRNOT00000052527	0.73	-438	78.6	turq	10758031	
	ENSRNOT00000061717	1.41	13.8	77.9	turq	10731408	
	ENSRNOT00000066479	0.78	-70.2	77.7	turq	10906975	
	ENSRNOT00000053296	1.51	8.9	77.5	turq	10805849	
RGD1310269	NM_001126280	0.80	-44.9	77.5	turq	10892309	hypothetical LOC314472
	ENSRNOT00000054102	0.70	-32.6	76.2	turq	10723896	
	ENSRNOT00000053919	0.78	-85.4	75.9	turq	10755129	
	J01884	2.07	35.0	75.2	turq	10830454	
	ENSRNOT00000054162	1.50	11.3	74.8	turq	10800832	
	AF272707	0.76	-17.8	73.8	turq	10867318	
	ENSRNOT00000052947	1.36	19.9	73.3	turq	10826602	
	ENSRNOT00000053975	1.30	37.2	73.1	turq	10798467	
	GENSCAN00000017522	1.34	3.6	72.3	turq	10779348	
	ENSRNOT00000052957	1.24	18.1	72.1	turq	10863888	
	ENSRNOT00000052978	0.81	-18.8	69.7	turq	10721700	
	ENSRNOT00000052442	0.72	-79.3	68.8	turq	10709825	
	ENSRNOT00000052632	0.74	-93.1	66.9	turq	10871769	
	ENSRNOT00000060493	0.78	-109	63.7	turq	10794544	

	ENSRNOT00000052472	0.83	-28.1	63.5	turq	10728561	
	ENSRNOT00000053447	0.80	-17.8	63.0	turq	10765495	
	ENSRNOT00000016662	1.31	5.7	62.4	turq	10718474	
	ENSRNOT00000044406	1.28	30.7	62.0	turq	10881468	
	ENSRNOT00000024516	1.46	34.2	61.6	turq	10729852	
	ENSRNOT00000052749	0.79	-4.3	60.9	turq	10779724	
	ENSRNOT00000053086	0.71	-342	59.7	turq	10811999	
	---	0.78	-72.5	58.2	turq	10851813	
	ENSRNOT00000053364	0.72	-56.8	57.7	turq	10721694	
	ENSRNOT00000054132	1.31	65.0	57.1	turq	10832137	
	ENSRNOT00000053049	1.23	14.4	55.4	turq	10762224	
	ENSRNOT00000054649	1.57	152	55.3	turq	10930226	
	ENSRNOT00000027783	0.80	-13.5	54.8	turq	10705126	
	ENSRNOT00000028590	1.39	17.9	54.7	turq	10746093	
	ENSRNOT00000053402	0.69	-292	54.5	turq	10721698	
RGD1304952	BC085924	0.82	-15.9	53.8	turq	10858726	similar to RIKEN cDNA C530028O21 gene
	ENSRNOT00000053479	0.72	-8.4	53.7	turq	10851380	
	ENSRNOT00000054352	1.64	8.2	53.7	turq	10778377	
	ENSRNOT00000053268	1.38	95.3	52.4	turq	10909356	
	ENSRNOT00000052577	0.75	-20.4	51.3	turq	10878961	
	ENSRNOT00000053476	1.33	18.0	50.8	turq	10702293	
	AF188753	0.77	-340	49.9	turq	10855185	
	ENSRNOT00000052060	0.78	-4.5	49.5	turq	10783533	
	ENSRNOT00000053071	0.76	-4.6	49.2	turq	10806012	
	NC_001665	1.48	176	48.9	turq	10930604	
	ENSRNOT00000040771	1.21	17.7	48.2	turq	10736914	
	ENSRNOT00000053505	1.31	6.3	47.3	turq	10872679	
	ENSRNOT00000021633	1.25	8.2	46.9	turq	10804560	
	ENSRNOT00000045406	1.44	32.3	46.4	turq	10881451	
	ENSRNOT00000040382	1.23	3.8	46.4	turq	10850084	
	ENSRNOT00000052861	1.37	8.0	46.0	turq	10734828	
	ENSRNOT00000052576	1.60	36.1	45.0	turq	10878967	
	ENSRNOT00000063523	1.28	891	44.4	turq	10761253	
	ENSRNOT00000052441	0.79	-11.3	44.0	turq	10841716	
	ENSRNOT00000039948	1.43	32.7	44.0	turq	10767044	
	ENSRNOT00000018039	1.23	2.8	43.3	turq	10923336	
	ENSRNOT00000053929	1.65	82.5	42.3	turq	10839872	
	ENSRNOT00000020541	1.30	6.8	40.1	turq	10858497	
	ENSRNOT00000049673	1.31	7.1	39.4	turq	10821820	
	ENSRNOT00000053328	0.63	-149	39.1	turq	10855946	
	---	1.23	7.3	38.6	turq	10838324	
	ENSRNOT00000054178	1.27	7.6	38.3	turq	10914316	
	ENSRNOT00000008028	1.33	15.3	37.3	turq	10752515	
	ENSRNOT00000027208	0.82	-13.5	37.1	turq	10924588	
	ENSRNOT00000063677	1.21	2.3	36.7	turq	10741802	
	ENSRNOT00000052710	1.55	33.5	36.4	turq	10924172	
	ENSRNOT00000053201	1.27	7.5	36.3	turq	10819998	
	ENSRNOT00000021284	1.30	28.2	34.0	turq	10859627	
	ENSRNOT00000033248	1.28	4.5	33.7	turq	10782607	
	ENSRNOT00000030198	1.28	7.7	29.1	turq	10798459	
	BC097994	0.79	-6.2	28.9	turq	10885029	
	ENSRNOT00000053105	1.23	9.3	28.2	turq	10881643	
	ENSRNOT00000022921	0.83	-16.6	26.4	turq	10880530	
	---	1.75	27.7	25.8	turq	10911811	
	J05014	0.82	-179	25.2	turq	10876313	
	ENSRNOT00000052628	0.69	-44.1	24.9	turq	10834602	
	---	0.81	-11.2	24.6	turq	10707118	
	ENSRNOT00000055733	1.23	3.2	24.5	turq	10881341	
	ENSRNOT00000053883	0.75	-34.1	23.8	turq	10765040	
	ENSRNOT00000009782	1.20	5.5	20.9	turq	10918653	
	ENSRNOT00000037995	1.22	14.1	19.9	turq	10758724	
RGD1359158	BC093390	1.21	30.6	19.6	turq	10877994	similar to RIKEN cDNA 1110059E24
	---	1.27	4.4	19.4	turq	10814426	
	ENSRNOT00000053542	0.77	-25.5	19.0	turq	10871771	
	NM_001108704	0.83	-19.8	18.4	turq	10883808	
	---	0.82	-22.2	17.9	turq	10811501	
	GENSCAN00000030991	0.83	-6.4	17.0	turq	10718830	
	ENSRNOT00000053410	1.39	7.3	15.1	turq	10736193	
	ENSRNOT00000031920	1.26	18.4	13.3	turq	10741861	

	ENSRNOT00000053250	1.25	7.0	9.9	turq	10766287	
	ENSRNOT00000053951	1.45	44.7	7.7	turq	10703224	
	ENSRNOT00000003022	1.21	20.4	4.1	turq	10825165	
	**ENSRNOT00000002159**	**1.27**	**20.0**	**7.9**	**brwn**	**10911944**	
	ENSRNOT00000039731	1.27	4.3	6.9	brwn	10785174	
	ENSRNOT00000054287	1.25	11.7	6.4	brwn	10802556	
	ENSRNOT00000054190	0.73	-19.1	4.8	brwn	10756255	
	ENSRNOT00000054495	1.21	2.6	3.9	brwn	10772160	
RGD1307830	ENSRNOT00000059984	1.21	33.3	2.9	brwn	10768397	similar to cDNA sequence BC003331
	ENSRNOT00000052689	0.76	-16.0	2.5	brwn	10896964	
	ENSRNOT00000040709	1.20	2.5	2.5	brwn	10776665	
	ENSRNOT00000051096	1.22	9.6	2.1	brwn	10787973	
	ENSRNOT00000051665	1.31	11.2	2.0	brwn	10720130	
	ENSRNOT00000056240	1.27	26.2	2.0	brwn	10926089	
	ENSRNOT00000057054	0.82	-9.6	1.9	brwn	10743964	
	ENSRNOT00000058957	0.82	-19.3	1.9	brwn	10937867	
	ENSRNOT00000053237	1.23	1.6	1.8	brwn	10757702	
	ENSRNOT00000010282	1.30	13.1	1.7	brwn	10812881	
	ENSRNOT00000054187	1.23	11.4	1.7	brwn	10757153	
	ENSRNOT00000036884	1.32	3.6	1.7	brwn	10795677	
	ENSRNOT00000060721	1.29	5.1	1.7	brwn	10883452	
	---	0.78	-5.2	1.3	brwn	10824526	
	---	1.71	60.4	1.0	brwn	10886894	
	ENSRNOT00000054140	0.79	-16.5	0.9	brwn	10783318	
LOC500700	ENSRNOT00000057756	1.24	6.0	0.7	brwn	10891405	similar to chromosome 14 open reading frame 145
	ENSRNOT00000054464	1.28	4.8	0.7	brwn	10759483	
	---	1.32	5.9	0.6	brwn	10850319	
	GENSCAN00000044942	1.22	7.2	0.5	brwn	10821147	
	---	0.78	-5.8	0.4	brwn	10859162	
	---	1.25	2.9	0.3	brwn	10852392	
	**ENSRNOT00000054517**	**1.38**	**15.6**	**33.1**	**blue**	**10932228**	
**RGD1311745**	**BC085913**	**1.20**	**21.3**	**29.4**	**blue**	**10921086**	**similar to RIKEN cDNA 1110059G10**
	ENSRNOT00000031340	0.77	-9.3	27.7	blue	10890232	
	ENSRNOT00000001251	1.32	5.5	26.0	blue	10758048	
RGD1305823	BC158662	1.20	26.8	25.0	blue	10731242	similar to RIKEN cDNA 0610037P05
	ENSRNOT00000009519	1.40	127	23.8	blue	10863469	
RGD1308923	NM_001108698	0.80	-5.1	19.4	blue	10874585	LOC362678
	ENSRNOT00000053230	0.71	-34.3	19.1	blue	10758033	
	ENSRNOT00000052065	1.23	87.8	18.1	blue	10739167	
LOC301124	NM_001134533	1.21	115	17.1	blue	10930914	hypothetical LOC301124
	ENSRNOT00000017930	1.42	130	17.0	blue	10791507	
	---	0.78	-6.4	16.1	blue	10791419	
	ENSRNOT00000058436	1.21	27.9	15.8	blue	10788858	
	ENSRNOT00000032056	0.83	-10.8	15.2	blue	10770924	
	ENSRNOT00000031724	1.31	17.4	13.7	blue	10872098	
	ENSRNOT00000053394	0.82	-3.8	13.0	blue	10900318	
	ENSRNOT00000036340	1.25	20.9	12.2	blue	10781280	
	ENSRNOT00000041981	1.29	19.8	11.9	blue	10763137	
	**ENSRNOT00000052502**	**0.82**	**-6.4**	**11.4**	**blue**	**10726170**	
	ENSRNOT00000068510	1.37	70.5	10.7	blue	10866486	
RGD1562342	NM_001109281	0.77	-11.3	10.6	blue	10899938	similar to RIKEN cDNA 1110012D08
	ENSRNOT00000019570	1.22	38.8	9.6	blue	10865483	
	ENSRNOT00000041909	0.82	-2.6	8.1	blue	10760807	
	ENSRNOT00000004471	0.82	-12.9	7.9	blue	10770066	
	ENSRNOT00000060122	1.20	10.2	7.1	blue	10884046	
	---	0.83	-74.0	7.0	blue	10767985	
	ENSRNOT00000058540	0.81	-5.3	6.9	blue	10885482	
	---	1.23	23.8	6.4	blue	10838352	
	ENSRNOT00000043131	1.34	45.9	5.7	blue	10774908	
	---	0.79	-5.8	5.7	blue	10909375	
	rno-mir-219-2	0.78	-3.3	4.5	blue	10844098	
	ENSRNOT00000045858	0.77	-2.4	4.4	blue	10772898	
	ENSRNOT00000053524	0.83	-6.8	1.4	blue	10850666	
	ENSRNOT00000047432	1.26	3.4	0.0	grey	10887118	
	ENSRNOT00000033332	1.21	2.3	0.0	grey	10880265	
	---	1.21	25.0	0.0	grey	10788687	
	---	0.81	-8.7	0.0	grey	10715344	

*** - genes belonning to top 10% of each module withing each brain region are marked by bold font**

**- Modules and k.in are given for seaparate networks; abbreviations used for modules: *turq* - turquoise, *ylw* -yellow, *brwn* - brown

Table S2-J.Differentially Expressed Genes in Female Rat Enterorhinal Cortex (424 genes and ESTs)							
Gene Symbol	GenBank, Ref.Sequence	Vin/Con Ratio	Vin-Con mean _dif	k.in **	Module**	ProbeSet ID	Gene Title
**Apoptosis**							
**Bcl2l1**	**NM_001033671**	**1.25**	**40.4**	**25.7**	**turq**	**10724228**	**Bcl2-like 1**
**Cell Cycle**							
Ccnk	NM_001109672	1.20	13.3	4.0	turq	10886714	cyclin K
Cdc25b	NM_133572	1.22	10.9	10.0	turq	10840020	cell division cycle 25 homolog B (S. pombe)
Cspp1	ENSRNOT00000008480	1.23	19.2	2.5	turq	10875089	centrosome and spindle pole associated protein 1
**Egr1**	**NM_012551**	**0.67**	**-90.5**	**23.0**	**turq**	**10800919**	**early growth response 1**
Pak7	NM_001107781	1.26	31.4	9.4	turq	10850208	p21 protein (Cdc42/Rac)-activated kinase 7
**Cytoskeleton-ECM**							
Cdh18	NM_001107656	1.32	54.8	8.3	turq	10813872	cadherin 18, type 2
Cldn11	NM_053457	0.61	-91.8	7.3	turq	10822631	claudin 11
Cldn9	NM_001011889	0.83	-2.6	14.7	turq	10740876	claudin 9
Clec4a3	NM_001005891	0.82	-10.4	5.7	turq	10858559	C-type lectin domain family 4, member a3
Cntn2	NM_012884	0.63	-46.1	17.6	turq	10767605	contactin 2 (axonal)
Cntn4	NM_053879	1.31	36.6	9.6	turq	10857512	contactin 4
Coro2b	ENSRNOT00000020951	0.82	-42.2	4.5	turq	10918075	coronin, actin binding protein, 2B
**Dnm3**	**NM_138538**	**1.37**	**67.9**	**26.9**	**turq**	**10769271**	**dynamin 3**
Dync1h1	NM_019226	1.20	95.6	19.2	turq	10887144	dynein cytoplasmic 1 heavy chain 1
Flrt2	NM_001106750	0.76	-14.0	14.4	turq	10886269	fibronectin leucine rich transmembrane protein 2
Gjb6	NM_053388	0.79	-76.1	6.0	turq	10784120	gap junction protein, beta 6
Hapln2	NM_022285	1.69	47.8	8.8	turq	10824242	hyaluronan and proteoglycan link protein 2
Ipcef1	NM_001170799	1.22	26.7	5.7	turq	10717803	interactor protein for cytohesin exchange factors 1
Lrfn1	NM_001127694	0.75	-15.8	10.7	turq	10705519	leucine rich repeat and fibronectin type III domain containing 1
**Mast4**	**ENSRNOT00000061032**	**0.75**	**-27.2**	**20.7**	**turq**	**10821072**	**microtubule associated serine/threonine kinase family member 4**
Mical2	NM_001139508	1.26	46.7	12.9	turq	10709951	microtubule associated monoxygenase, calponin and LIM domain containing 2
Myl12b	NM_017343	1.22	70.9	13.0	turq	10930416	myosin, light chain 12B, regulatory
Nup210	NM_053322	1.20	4.8	13.3	turq	10864048	nucleoporin 210
Snap47	NM_199389	1.22	14.2	6.4	turq	10743137	synaptosomal-associated protein, 47
**Sparc**	**NM_012656**	**0.75**	**-411**	**25.3**	**turq**	**10742802**	**secreted protein, acidic, cysteine-rich (osteonectin)**
Spock1	ENSRNOT00000061187	0.79	-70	8.7	turq	10793909	sparc/osteonectin, cwcv and kazal-like domains proteoglycan (testican) 1
**Stmn1**	**NM_017166**	**1.36**	**399**	**29.0**	**turq**	**10872801**	**stathmin 1**
Synm	NM_001134858	0.79	-8	2.8	blue	10722694	synemin, intermediate filament protein
Tspan12	NM_001015026	0.83	-63.3	9.6	turq	10861213	tetraspanin 12
Tspan33	NM_001109227	0.79	-33.6	8.0	turq	10854199	tetraspanin 33
Tspan6	NM_001100672	0.81	-46.8	18.2	turq	10939233	tetraspanin 6
**Tspan9**	**NM_001107890**	**0.78**	**-7.9**	**12.1**	**turq**	**10865800**	**tetraspanin 9**
Ttll7	ENSRNOT00000055318	1.24	118	17.1	turq	10819749	tubulin tyrosine ligase-like family, member 7
**Development**							
**Asap1**	**NM_001044245**	**1.34**	**83.3**	**24.6**	**turq**	**10904031**	**ArfGAP with SH3 domain, ankyrin repeat and PH domain 1**
**Bmper**	**NM_001135799**	**0.83**	**-6.0**	**23.3**	**turq**	**10908645**	**BMP-binding endothelial regulator**
Cbln2	NM_001012740	1.77	48.6	16.6	turq	10803051	cerebellin 2 precursor
Cecr6	XM_001058567	1.27	3.5	1.9	blue	10865198	cat eye syndrome chromosome region, candidate 6 homolog (human)
Clmn	NM_001106755	1.21	23.7	7.1	turq	10892035	calmin
**Cobl**	**NM_001107236**	**1.31**	**19.9**	**26.5**	**turq**	**10778525**	**cordon-bleu homolog (mouse)**
Dopey2	ENSRNOT00000002302	0.80	-25.6	4.0	turq	10750333	dopey family member 2
Fhl2	NM_031677	1.30	11.0	2.4	blue	10927692	four and a half LIM domains 2
Lix1	NM_001106214	0.78	-212	17.7	turq	10703414	Lix1 homolog (chicken)
Lrrc6	NM_001025659	1.21	5.3	4.7	turq	10904139	leucine rich repeat containing 6 (testis)
Lrrc8b	NM_001107204	0.73	-26.7	17.6	turq	10775278	leucine rich repeat containing 8 family, member B
LRRTM1	NM_001109374	0.81	-53.4	5.8	turq	10856472	leucine rich repeat transmembrane neuronal 1
Olfm3	NM_145777	1.45	45.8	11.7	turq	10818573	olfactomedin 3
Opalin	NM_001017386	1.57	35.3	16.3	turq	10730148	oligodendrocytic myelin paranodal and inner loop protein
Pno1	NM_199083	0.81	-29.8	0.0	grey	10778568	partner of NOB1 homolog (S. cerevisiae)
Prickle1	NM_199396	1.23	38.5	16.4	turq	10906479	prickle homolog 1 (Drosophila)
Prrx2	NM_001105739	1.21	1.9	0.0	grey	10835257	paired related homeobox 2
Rcan2	NM_175578	0.81	-89.0	11.4	turq	10926658	regulator of calcineurin 2
Scrg1	NM_033499	0.77	-83.7	9.5	turq	10791500	stimulator of chondrogenesis 1
Svs3	NM_001007605	0.82	-2.1	6.7	turq	10842091	seminal vesicle secretion 3
**Sytl2**	**NM_001108492**	**1.22**	**14.5**	**21.0**	**turq**	**10708591**	**synaptotagmin-like 2**
Trpc3	NM_021771	1.36	11.2	13.8	turq	10822888	transient receptor potential cation channel, subfamily C, member 3
**Unc13c**	**NM_173146**	**0.53**	**-44.6**	**22.9**	**turq**	**10918674**	**unc-13 homolog C (C. elegans)**
Vamp1	NM_013090	1.30	80.6	11.1	turq	10858858	vesicle-associated membrane protein 1
**Electron Transport**							

Cyp2b2	ENSRNOT00000028196	0.82	-2.3	18.0	turq	10705230	cytochrome P450, family 2, subfamily b, polypeptide 2
**Epigenetics**							
Chd3	ENSRNOT00000012983	0.82	-94.4	1.1	blue	10743914	chromodomain helicase DNA binding protein 3
Chmp6	NM_001105856	0.71	-32.7	3.5	blue	10749888	chromatin modifying protein 6
Cxxc5	NM_001007628	1.29	54.4	11.8	turq	10800969	CXXC finger 5
Jmjd1c	ENSRNOT00000000804	1.22	34.0	5.5	turq	10832831	jumonji domain containing 1C
Pcgf6	NM_001013154	1.28	12.6	4.0	turq	10730624	polycomb group ring finger 6
Pim3	NM_022602	1.21	8.6	19.7	turq	10898474	pim-3 oncogene
Satb1	NM_001012129	1.42	62.3	12.2	turq	10921274	SATB homeobox 1
**Satb2**	**NM_001109306**	**0.81**	**-7.8**	**21.8**	**turq**	**10928191**	**SATB homeobox 2**
**Golgi Apparatus**							
Gal3st3	NM_001024290	0.81	-18.4	4.1	turq	10713015	galactose-3-O-sulfotransferase 3
Hs3st2	NM_181370	1.25	4.6	5.2	turq	10710575	heparan sulfate (glucosamine) 3-O-sulfotransferase 2
Man2a1	NM_012979	1.24	18.9	13.8	turq	10925783	mannosidase, alpha, class 2A, member 1
Manba	NM_001031655	1.21	9.0	8.6	turq	10819245	mannosidase, beta A, lysosomal
St6gal2	AJ627626	1.23	30.5	1.5	turq	10930711	ST6 beta-galactosamide alpha-2,6-sialyltranferase 2
**Growth Factor**							
Angpt1	NM_053546	0.83	-8.7	6.3	turq	10903529	angiopoietin 1
Crlf1	NM_001106074	0.71	-22.6	11.0	turq	10791000	cytokine receptor-like factor 1
Grb14	NM_031623	1.26	25.1	14.7	turq	10845751	growth factor receptor bound protein 14
Megf11	ENSRNOT00000050649	0.79	-4.8	1.6	blue	10910776	multiple EGF-like-domains 11
Mfge8	NM_001040186	0.80	-71.9	4.1	turq	10722903	milk fat globule-EGF factor 8 protein
Npy	NM_012614	1.24	79.2	2.7	blue	10855506	neuropeptide Y
40427	NM_001173429	1.24	31.8	4.0	blue	10932016	septin 6
Tgfb3	NM_013174	0.71	-11.3	16.3	turq	10891303	transforming growth factor, beta 3
Wnt7b	NM_001009695	0.77	-28.0	15.5	turq	10905974	wingless-type MMTV integration site family, member 7B
**Immune Response**							
Cd34	NM_001107202	0.78	-10.2	8.7	turq	10766869	CD34 molecule
Cd38	NM_013127	0.81	-10.4	6.9	turq	10777232	CD38 molecule
Cd83	NM_001108410	1.20	8.8	7.5	turq	10797811	CD83 molecule
Igsf1	NM_175763	1.24	26.8	16.6	turq	10939668	immunoglobulin superfamily, member 1
Igsf8	NM_001014787	1.23	9.6	8.9	turq	10765730	immunoglobulin superfamily, member 8
LOC681426	ENSRNOT00000043963	0.82	-1.6	8.9	turq	10877336	similar to alpha-2u globulin PGCL4 isoform 1
Mup5	NM_203325	0.82	-2.3	4.6	turq	10877307	major urinary protein 5
Tox	NM_001108654	1.26	14.6	7.7	turq	10875363	thymocyte selection-associated high mobility group box
Xkr6	NM_001012042	1.33	20.0	5.1	turq	10781005	XK, Kell blood group complex subunit-related family, member 6
Xlr3a	ENSRNOT00000040782	0.83	-1.9	0.0	grey	10940120	X-linked lymphocyte-regulated 3A
**Metabolism & Transport**							
Acsl1	NM_012820	1.23	26.2	9.0	turq	10791677	acyl-CoA synthetase long-chain family member 1
Aldh7a1	ENSRNOT00000020325	0.81	-46.6	8.8	turq	10804522	aldehyde dehydrogenase 7 family, member A1
Asah2	NM_053646	1.37	13.4	5.2	turq	10729693	N-acylsphingosine amidohydrolase (non-lysosomal ceramidase) 2
Atad2	NM_001134879	1.25	6.6	13.0	turq	10903864	ATPase family, AAA domain containing 2
Atp6ap1l	ENSRNOT00000044237	0.74	-19.6	9.3	turq	10820308	ATPase, H+ transporting, lysosomal accessory protein 1-like
Car12	NM_001080756	0.67	-65.1	16.5	turq	10911145	carbonic anyhydrase 12
Clic5	NM_053603	1.41	4.5	18.9	turq	10926642	chloride intracellular channel 5
Clstn1	ENSRNOT00000022100	0.83	-117	15.4	turq	10874095	calsyntenin 1
Clstn2	NM_134377	1.24	47.2	10.2	turq	10912425	calsyntenin 2
Cox4nb	NM_001012165	0.83	-10.9	0.0	grey	10811493	COX4 neighbor
Cpt1a	NM_031559	0.82	-19.5	4.5	turq	10712657	carnitine palmitoyltransferase 1a, liver
Doc2a	NM_022937	1.24	18.2	15.6	turq	10710936	double C2-like domains, alpha
Dpyd	NM_031027	0.66	-19.1	3.5	turq	10818660	dihydropyrimidine dehydrogenase
Elovl2	NM_001109118	0.80	-60.6	12.1	turq	10794609	elongation of very long chain fatty acids (FEN1/Elo2, SUR4/Elo3, yeast)-like 2
Extl2	NM_001100704	1.26	66.6	14.3	turq	10818590	exostoses (multiple)-like 2
Far2	ENSRNOT00000002528	1.24	14.9	3.0	turq	10859748	fatty acyl CoA reductase 2
Gipr	NM_012714	1.21	3.1	3.1	turq	10719394	gastric inhibitory polypeptide receptor
Gldc	NM_001107583	0.83	-23.9	5.5	turq	10729635	glycine dehydrogenase (decarboxylating)
Gls2	NM_138904	1.23	22.4	10.8	turq	10893008	glutaminase 2 (liver, mitochondrial)
Heph	NM_133304	0.76	-6.7	6.3	turq	10934073	hephaestin
Hsd11b1	NM_017080	0.82	-50.9	5.3	turq	10770795	hydroxysteroid 11-beta dehydrogenase 1
Kcna2	NM_012970	1.20	40.6	15.7	turq	10818245	potassium voltage-gated channel, shaker-related subfamily, member 2
Kcna6	NM_023954	1.38	28.0	8.4	turq	10865738	potassium voltage gated channel, shaker related subfamily, member 6
Kcnc1	NM_012856	1.30	40.5	15.8	turq	10707113	potassium voltage gated channel, Shaw-related subfamily, member 1
**Kcnc2**	**NM_139216**	**1.21**	**36.5**	**23.5**	**turq**	**10895443**	**potassium voltage gated channel, Shaw-related subfamily, member 2**
Kcnh1	NM_031742	1.33	46.9	16.5	turq	10766782	potassium voltage-gated channel, subfamily H (eag-related), member 1
Kcnh5	NM_133610	2.40	142	9.8	turq	10890609	potassium voltage-gated channel, subfamily H (eag-related), member 5
Kcnh7	NM_131912	1.34	54.8	9.8	turq	10845725	potassium voltage-gated channel, subfamily H (eag-related), member 7
Kcnip4	NM_181365	1.24	37.7	17.4	turq	10773030	Kv channel interacting protein 4
Kcnj16	NM_053314	1.36	52.4	15.8	turq	10739351	potassium inwardly-rectifying channel, subfamily J, member 16
Kctd4	NM_001109650	0.79	-67.2	8.2	turq	10781566	potassium channel tetramerisation domain containing 4

Kpna5	NM_001025113	1.20	22.3	8.0	turq	10830088	karyopherin alpha 5 (importin alpha 6)
Lpcat4	NM_001106494	0.76	-47.3	16.2	turq	10838451	lysophosphatidylcholine acyltransferase 4
**Lrp1b**	**NM_001107843**	**1.45**	**152**	**31.3**	**turq**	**10845051**	**low density lipoprotein-related protein 1B (deleted in tumors)**
Magi2	NM_053621	1.21	43.8	19.7	turq	10860272	membrane associated guanylate kinase, WW and PDZ domain containing 2
Mrs2	NM_024001	1.25	17.9	16.9	turq	10795082	MRS2 magnesium homeostasis factor homolog (S. cerevisiae)
Nos1ap	NM_138922	1.37	35.2	7.9	turq	10769723	nitric oxide synthase 1 (neuronal) adaptor protein
Nppa	NM_012612	0.73	-10.8	5.2	turq	10873899	natriuretic peptide precursor A
Orai2	NM_001170403	0.75	-8.6	1.2	blue	10757562	ORAI calcium release-activated calcium modulator 2
Piga	NM_001108816	0.82	-15.4	8.7	turq	10937719	phosphatidylinositol glycan anchor biosynthesis, class A
Plcb4	NM_024353	1.61	34.9	18.4	turq	10840183	phospholipase C, beta 4
Plcd4	NM_080688	0.82	-17.8	4.0	turq	10924335	phospholipase C, delta 4
Sardh	NM_053664	0.78	-6.4	17.6	turq	10843938	sarcosine dehydrogenase
**Scn1a**	**NM_030875**	**1.37**	**82.7**	**25.4**	**turq**	**10845859**	**sodium channel, voltage-gated, type I, alpha**
Scn1b	NM_017288	1.35	79.6	9.7	turq	10720907	sodium channel, voltage-gated, type I, beta
Slc28a2	NM_031664	1.32	12.5	16.1	turq	10849275	solute carrier family 28 (sodium-coupled nucleoside transporter), member 2
Slc2a9	ENSRNOT00000042200	0.81	-3.2	2.6	turq	10773221	solute carrier family 2 (facilitated glucose transporter), member 9
Slc35a3	NM_001012082	0.79	-17.9	6.5	turq	10826326	solute carrier family 35 (UDP-N-acetylglucosamine (UDP-GlcNAc) transporter), member A3
**Slc39a10**	**NM_001108796**	**1.22**	**77.9**	**21.4**	**turq**	**10923294**	**solute carrier family 39 (zinc transporter), member 10**
Slc5a11	NM_001100482	1.23	4.2	7.1	turq	10710718	solute carrier family 5 (sodium/glucose cotransporter), member 11
Slc7a1	NM_013111	1.27	15.7	16.3	turq	10756393	solute carrier family 7 (cationic amino acid transporter, y+ system), member 1
Slc9a3r1	NM_021594	0.82	-31.7	6.9	turq	10739490	solute carrier family 9 (sodium/hydrogen exchanger), member 3 regulator 1
Slco4c1	NM_001002024	1.22	5.6	3.7	turq	10930098	solute carrier organic anion transporter family, member 4C1
Sulf2	NM_001034927	0.79	-35.1	10.1	turq	10851839	sulfatase 2
Sult5a1	NM_001106194	1.20	2.9	0.5	brwn	10811732	sulfotransferase family 5A, member 1
Wdr19	ENSRNOT00000003991	1.21	20.2	6.6	turq	10776835	WD repeat domain 19
**Proteolysis**							
Adamts4	NM_023959	1.51	9.9	4.1	turq	10765534	ADAM metallopeptidase with thrombospondin type 1 motif, 4
**Agt**	**NM_134432**	**0.81**	**-78.4**	**23.4**	**turq**	**10811900**	**angiotensinogen (serpin peptidase inhibitor, clade A, member 8)**
Hecw1	NM_001106117	1.23	37.8	6.5	turq	10795482	HECT, C2 and WW domain containing E3 ubiquitin protein ligase 1
LOC689226	BC167074	1.30	42.0	2.9	blue	10868343	similar to ubiquitin-conjugating enzyme E2R 2
Lonrf3	ENSRNOT00000017550	0.82	-22.3	5.2	turq	10936346	LON peptidase N-terminal domain and ring finger 3
**Pcsk2**	**NM_012746**	**1.29**	**110**	**28.5**	**turq**	**10840332**	**proprotein convertase subtilisin/kexin type 2**
Spint2	NM_001082549	0.78	-18.4	6.1	turq	10720479	serine peptidase inhibitor, Kunitz type, 2
**Ube2o**	**ENSRNOT00000014699**	**1.21**	**27.5**	**34.0**	**turq**	**10749287**	**ubiquitin-conjugating enzyme E2O**
Ubxn8	NM_001106086	0.82	-17.4	5.8	turq	10788583	UBX domain protein 8
**Receptors & Binding Proteins**							
Abca2	NM_024396	1.37	76.3	13.2	turq	10834241	ATP-binding cassette, sub-family A (ABC1), member 2
Abca8	ENSRNOT00000065947	0.80	-7.6	8.4	turq	10748564	ATP-binding cassette, sub-family A (ABC1), member 8
Abcc5	NM_053924	0.76	-39.6	10.7	turq	10752148	ATP-binding cassette, sub-family C (CFTR/MRP), member 5
Abcc8	NM_013039	0.79	-8.8	2.6	turq	10722012	ATP-binding cassette, sub-family C (CFTR/MRP), member 8
Adra1d	NM_024483	0.78	-10.5	13.6	turq	10850087	adrenergic, alpha-1D-, receptor
Alb	NM_134326	0.57	-15.6	13.2	turq	10775968	albumin
Antxr1	NM_001044249	1.30	10.2	6.6	turq	10863777	anthrax toxin receptor 1
Boc	NM_001108317	1.24	9.9	7.6	turq	10751115	biregional cell adhesion molecule-related/down-regulated by oncogenes (Cdon) binding
**Chrm2**	**NM_031016**	**1.42**	**33.1**	**23.3**	**turq**	**10854544**	**cholinergic receptor, muscarinic 2**
**Chrm3**	**NM_012527**	**1.21**	**29.1**	**22.7**	**turq**	**10795898**	**cholinergic receptor, muscarinic 3**
Chrm5	NM_017362	0.70	-7.7	3.0	turq	10848165	cholinergic receptor, muscarinic 5
Chrna4	NM_024354	1.23	4.8	7.8	turq	10852428	cholinergic receptor, nicotinic, alpha 4
Cttnbp2	NM_001114401	1.24	77.8	14.8	turq	10861171	cortactin binding protein 2
Fgfr3	NM_053429	0.82	-32.4	13.5	turq	10777748	fibroblast growth factor receptor 3
Gabrg3	NM_024370	1.24	26.5	2.3	turq	10722328	gamma-aminobutyric acid (GABA) A receptor, gamma 3
Glrb	NM_053296	1.21	83.8	5.5	turq	10823875	glycine receptor, beta
**Igfbp5**	**NM_012817**	**0.77**	**-49.1**	**20.5**	**turq**	**10928837**	**insulin-like growth factor binding protein 5**
**Il1rap**	**NM_012968**	**1.55**	**53.5**	**31.0**	**turq**	**10755013**	**interleukin 1 receptor accessory protein**
Il4ra	NM_133380	0.81	-12.5	0.0	grey	10710782	interleukin 4 receptor, alpha
Il6ra	NM_017020	0.82	-11.3	16.7	turq	10824530	interleukin 6 receptor, alpha
Lrp4	NM_031322	0.82	-22.8	0.8	blue	10837881	low density lipoprotein receptor-related protein 4
N4bp2	ENSRNOT00000031792	0.80	-10.8	10.4	turq	10776778	NEDD4 binding protein 2
Olr1437	NM_001000015	1.27	4.9	0.9	blue	10733876	olfactory receptor 1437
Olr712	NM_001000624	0.78	-2.8	4.2	turq	10847215	olfactory receptor 712
**Osbpl3**	**ENSRNOT00000013828**	**0.76**	**-10.9**	**23.6**	**turq**	**10862473**	**oxysterol binding protein-like 3**
P2ry12	NM_022800	0.79	-42.1	12.9	turq	10823365	purinergic receptor P2Y, G-protein coupled, 12
Pvalb	NM_022499	1.53	62.0	9.4	turq	10905277	parvalbumin
S100a10	NM_031114	1.24	48.1	0.0	grey	10817186	S100 calcium binding protein A10
Sec61g-ps1	AY383674	1.33	4.6	4.8	turq	10806545	Sec61 gamma subunit, pseudogene 1
Secisbp2l	NM_001168527	1.23	74.3	6.7	turq	10849436	SECIS binding protein 2-like
Selv	NM_001166396	0.83	-2.6	5.6	turq	10720144	selenoprotein V
Stxbp6	ENSRNOT00000005618	1.22	60.8	8.3	turq	10889772	syntaxin binding protein 6 (amisyn)
Tf	NM_001013110	1.71	268	17.6	turq	10919637	transferrin

Wbp2	NM_138975	1.24	59.3	15.0	turq	10749108	WW domain binding protein 2
Wipf2	ENSRNOT00000029021	1.26	8.9	2.8	turq	10738109	WAS/WASL interacting protein family, member 2
**Signaling**							
Adcyap1r1	NM_133511	0.80	-92.1	15.8	turq	10855727	adenylate cyclase activating polypeptide 1 (pituitary) receptor type I
Arc	NM_019361	0.72	-68.6	10.5	turq	10904511	activity-regulated cytoskeleton-associated protein
Arhgap23	ENSRNOT00000055414	0.79	-14.5	13.7	turq	10737838	Rho GTPase activating protein 23
**Arhgef3**	**NM_001106061**	**1.22**	**10.6**	**28.9**	**turq**	**10786338**	**Rho guanine nucleotide exchange factor (GEF) 3**
Bcar1	NM_012931	1.25	11.0	3.7	turq	10811185	breast cancer anti-estrogen resistance 1
**Diras2**	**NM_001169578**	**1.24**	**34.2**	**22.2**	**turq**	**10794240**	**DIRAS family, GTP-binding RAS-like 2**
**Dixdc1**	**NM_001037654**	**1.31**	**17.8**	**21.5**	**turq**	**10917260**	**DIX domain containing 1**
Dkk3	NM_138519	1.46	102	18.6	turq	10724967	dickkopf homolog 3 (Xenopus laevis)
Dlc1	NM_001127446	1.23	19.0	7.2	turq	10788462	deleted in liver cancer 1
Dok4	NM_001108438	0.79	-16.7	8.2	turq	10805976	docking protein 4
Dusp1	NM_053769	0.81	-25.2	10.1	turq	10732652	dual specificity phosphatase 1
Efna5	NM_053903	1.49	18.0	8.7	turq	10930204	ephrin A5
Efnb3	NM_001100980	0.71	-20.2	4.5	turq	10744081	ephrin B3
Egfl6	NM_001108254	0.72	-8.8	16.9	turq	10933349	EGF-like-domain, multiple 6
Entpd5	NM_199394	1.32	13.5	5.0	turq	10891104	ectonucleoside triphosphate diphosphohydrolase 5
**Eps8**	**ENSRNOT00000009328**	**0.80**	**-25.9**	**1.0**	**brwn**	**10866544**	**epidermal growth factor receptor pathway substrate 8**
Fkbp10	NM_001014120	0.77	-6.3	3.4	turq	10738177	FK506 binding protein 10
Fkbp14	NM_001013210	0.74	-33.6	12.9	turq	10862643	FK506 binding protein 14
Frmd5	ENSRNOT00000064908	1.47	64.4	14.9	turq	10849156	FERM domain containing 5
Frmpd4	NM_001106960	1.21	28.8	3.0	turq	10933312	FERM and PDZ domain containing 4
Gng8	NM_139185	1.24	8.0	13.4	turq	10704560	guanine nucleotide binding protein (G protein), gamma 8
Gpr68	NM_001108049	1.21	5.3	3.4	turq	10891679	G protein-coupled receptor 68
**Grik3**	**NM_001112716**	**1.41**	**20.6**	**26.5**	**turq**	**10871939**	**glutamate receptor, ionotropic, kainate 3**
Grik4	NM_012572	1.41	27.8	7.6	turq	10916579	glutamate receptor, ionotropic, kainate 4
Grk5	NM_030829	1.27	12.0	9.6	turq	10716480	G protein-coupled receptor kinase 5
Hsn2	NM_001002823	1.25	12.2	8.8	turq	10865163	hereditary sensory neuropathy, type II
Ifi27	NM_203410	1.33	59.9	11.6	turq	10886573	interferon, alpha-inducible protein 27
Itpr1	NM_001007235	1.29	143	4.4	turq	10857546	inositol 1,4,5-triphosphate receptor, type 1
Mapk11	NM_001109532	1.21	6.6	12.4	turq	10906156	mitogen-activated protein kinase 11
Mapk6	NM_031622	1.22	45.1	9.3	turq	10918708	mitogen-activated protein kinase 6
Mark4	ENSRNOT00000023392	1.21	9.8	2.9	turq	10719445	MAP/microtubule affinity-regulating kinase 4
Nrgn	NM_024140	1.26	153	4.0	turq	10916228	neurogranin
Nuak1	NM_001106774	1.22	11.2	6.6	turq	10894552	NUAK family, SNF1-like kinase, 1
Pde9a	NM_138543	0.83	-9.9	6.2	turq	10829137	phosphodiesterase 9A
Peli3	NM_001127542	0.83	-12.1	1.2	turq	10727710	pellino 3
Pex5l	NM_173152	1.32	60.0	12.0	turq	10822735	peroxisomal biogenesis factor 5-like
Phldb1	BC104704	1.31	14.8	17.9	turq	10916825	pleckstrin homology-like domain, family B, member 1
Plekhg3	ENSRNOT00000008573	1.35	10.2	12.9	turq	10885417	pleckstrin homology domain containing, family G (with RhoGef domain) member 3
Pragmin	NM_001107315	0.66	-24.8	4.2	turq	10791989	pragma of Rnd2
**Prkcb**	**NM_012713**	**1.34**	**264**	**25.5**	**turq**	**10710647**	**protein kinase C, beta**
Prkg1	NM_001105731	0.82	-9.5	3.5	turq	10729673	protein kinase, cGMP-dependent, type 1
**Ptpro**	**NM_017336**	**1.25**	**29.9**	**8.2**	**turq**	**10859342**	**protein tyrosine phosphatase, receptor type, O**
Rac2	NM_001008384	0.81	-4.1	1.9	turq	10905316	ras-related C3 botulinum toxin substrate 2 (rho family, small GTP binding protein Rac2)
Ramp3	NM_020100	1.39	11.1	7.2	turq	10774115	receptor (G protein-coupled) activity modifying protein 3
Rap1gds1	NM_001107728	1.22	77.6	2.4	turq	10827043	RAP1, GTP-GDP dissociation stimulator 1
Rasgrp1	NM_019211	0.83	-162	12.6	turq	10848393	RAS guanyl releasing protein 1 (calcium and DAG-regulated)
**RGD1561440**	**ENSRNOT00000041190**	**1.21**	**46.6**	**20.6**	**turq**	**10745273**	**similar to nemo like kinase**
RGD1561955	ENSRNOT00000058079	1.23	21.6	6.2	turq	10785405	similar to diacylglycerol kinase eta
**RGD1562220**	**ENSRNOT00000003207**	**1.42**	**33.7**	**31.2**	**turq**	**10771492**	**similar to GPI-gamma 4; GPIgamma4**
**Rgs6**	**ENSRNOT00000058181**	**1.27**	**22.6**	**24.3**	**turq**	**10885769**	**regulator of G-protein signaling 6**
Scube1	NM_001134884	1.31	13.1	0.0	grey	10905843	signal peptide, CUB domain, EGF-like 1
Scube2	ENSRNOT00000052377	1.26	3.9	9.0	turq	10724792	signal peptide, CUB domain, EGF-like 2
Sh3bp4	NM_022693	1.29	13.9	4.4	turq	10925264	SH3-domain binding protein 4
**Skap2**	**NM_130413**	**0.82**	**-22.9**	**27.8**	**turq**	**10862527**	**src kinase associated phosphoprotein 2**
Sla	NM_178097	1.21	6.3	3.7	turq	10904161	src-like adaptor
Spred2	NM_001047094	1.21	22.0	10.2	turq	10774345	sprouty-related, EVH1 domain containing 2
Susd2	NM_001106381	0.82	-8.8	2.5	turq	10832496	sushi domain containing 2
Tpte	NM_001108877	1.55	6.4	17.2	turq	10792456	transmembrane phosphatase with tensin homology
Tulp3	ENSRNOT00000007634	0.80	-14.2	4.6	turq	10865830	tubby-like protein 3
**Transcription**							
Ankrd33b	ENSRNOT00000059565	1.24	17.2	19.8	turq	10822178	ankyrin repeat domain 33B
Armc2	ENSRNOT00000031047	1.28	7.6	7.1	turq	10833806	armadillo repeat containing 2
**Bhlhe22**	**NM_001108940**	**0.77**	**-12.0**	**23.3**	**turq**	**10814396**	**basic helix-loop-helix family, member e22**
Bhlhe40	NM_053328	1.30	90.7	4.6	turq	10857610	basic helix-loop-helix family, member e40
Btf3l4	XM_002729523	0.81	-31.6	1.8	turq	10878671	basic transcription factor 3-like 4
Camta1	ENSRNOT00000025106	1.29	33.4	17.3	turq	10881892	calmodulin binding transcription activator 1

Crtc2	NM_001033895	1.22	13.9	11.0	turq	10816965	CREB regulated transcription coactivator 2
Dnajc21	NM_138856	1.35	20.4	10.1	turq	10821900	DnaJ (Hsp40) homolog, subfamily C, member 21
Ebf4	ENSRNOT00000064943	1.23	6.6	5.7	turq	10839878	early B-cell factor 4
Foxp2	ENSRNOT00000007759	0.82	-5.5	14.1	turq	10853764	forkhead box P2
Hap1	NM_024133	0.75	-63.4	10.1	turq	10747313	huntingtin-associated protein 1
Hes5	NM_024383	0.77	-15.1	13.1	turq	10874534	hairy and enhancer of split 5 (Drosophila)
Ier5	NM_001025137	1.45	22.5	14.9	turq	10768814	immediate early response 5
Ipo4	NM_001106038	1.21	13.4	10.4	turq	10783818	importin 4
Klra17	NM_001172088	0.82	-4.4	3.1	turq	10866098	killer cell lectin-like receptor, subfamily A, member 17
LOC100363462	XR_086055	1.42	18.3	5.6	turq	10796917	zinc finger protein 107-like
LOC679999	ENSRNOT00000057650	0.79	-8.2	13.0	turq	10934480	similar to TGF beta-inducible nuclear protein 1 (L-name-related protein 42) (LNR42)
Mlxip	ENSRNOT00000001690	0.79	-8.9	12.6	turq	10761897	MLX interacting protein
**Ngef**	**NM_001136241**	**1.30**	**36.7**	**25.5**	**turq**	**10929660**	**neuronal guanine nucleotide exchange factor**
Npas4	NM_153626	0.75	-12.1	20.1	turq	10727717	neuronal PAS domain protein 4
Nr2c1	NM_145780	1.27	10.9	4.0	turq	10894974	nuclear receptor subfamily 2, group C, member 1
Nr4a1	NM_024388	0.66	-51.7	16.1	turq	10899387	nuclear receptor subfamily 4, group A, member 1
Nsbp1	NM_001134706	0.82	-7.1	4.3	turq	10707658	nucleosomal binding protein 1
Pias4	NM_001100757	1.22	10.0	4.6	turq	10893618	protein inhibitor of activated STAT, 4
Plagl1	NM_012760	0.66	-19.0	12.7	turq	10701802	pleiomorphic adenoma gene-like 1
RGD1560095	ENSRNOT00000040584	1.22	5.3	2.4	turq	10833564	similar to double homeobox, 4
RGD1560191	ENSRNOT00000003373	1.20	18.4	0.8	turq	10733067	similar to Zinc finger protein 62 homolog (Zfp-62) (ZT3)
Rprm	NM_001044276	1.22	8.1	0.9	turq	10845372	reprimo, TP53 dependent G2 arrest mediator candidate
Sp2	NM_001107045	1.28	6.0	16.3	turq	10746612	Sp2 transcription factor
Tbr1	ENSRNOT00000065340	0.81	-32.6	15.9	turq	10845628	T-box, brain, 1
Terf2ip	NM_001013143	1.22	18.6	7.5	turq	10808103	telomeric repeat binding factor 2, interacting protein
Zdhhc2	NM_145096	1.23	68.0	19.9	turq	10791935	zinc finger, DHHC-type containing 2
Zdhhc9	NM_001039016	1.21	17.3	9.2	turq	10939570	zinc finger, DHHC-type containing 9
Zeb2	NM_001033701	1.23	74.6	10.4	turq	10845072	zinc finger E-box binding homeobox 2
Zfp161	NM_172325	1.21	12.4	17.3	turq	10925966	zinc finger protein 161
Zfpm2	NM_001130501	1.37	17.8	13.3	turq	10896337	zinc finger protein, multitype 2
**Znf597**	**NM_153732**	**1.22**	**10.2**	**20.8**	**turq**	**10731915**	**zinc finger protein 597**
Znf711	ENSRNOT00000006237	0.82	-11.9	6.0	turq	10934720	zinc finger protein 711
Znhit2	NM_001107574	0.82	-12.4	4.4	turq	10713184	zinc finger, HIT type 2
**Translation & Protein Modofication**							
Snrnp35	NM_001014127	0.81	-9.7	3.7	turq	10761724	small nuclear ribonucleoprotein 35 (U11/U12)
Lsm7	NM_001108732	1.24	9.6	1.7	turq	10893684	LSM7 homolog, U6 small nuclear RNA associated (S. cerevisiae)
Rbm24	ENSRNOT00000022768	1.22	10.0	5.4	turq	10797774	RNA binding motif protein 24
RGD1564325	ENSRNOT00000047252	0.76	-4.0	0.8	brwn	10790214	similar to ribosomal protein S24
Arl4d	NM_001107052	1.35	15.4	4.8	turq	10738477	ADP-ribosylation factor-like 4D
Sf3b5	NM_001126092	1.31	18.8	7.1	turq	10701797	splicing factor 3b, subunit 5
**Miscellaneous & Unknown**							
Cachd1	ENSRNOT00000014354	0.74	-30.0	7.3	turq	10870211	cache domain containing 1
Dcbld1	ENSRNOT00000000462	0.82	-4.5	5.8	turq	10830135	discoidin, CUB and LCCL domain containing 1
Fam160b1	ENSRNOT00000023247	1.31	15.2	14.1	turq	10716303	family with sequence similarity 160, member B1
Fam189a2	BC166504	1.24	6.9	6.4	turq	10729397	family with sequence similarity 189, member A2
Fam57b	NM_001106296	0.76	-12.0	15.4	turq	10710930	family with sequence similarity 57, member B
Fam70a	NM_182822	0.76	-58.0	7.8	turq	10931930	family with sequence similarity 70, member A
LOC100364350	XM_002725175	0.81	-22.4	8.1	turq	10790658	NACHT and WD repeat domain containing 1-like
LOC304558	XM_222260	1.21	5.6	3.7	blue	10762932	similar to TPR repeat-containing protein KIAA1043
LOC679583	ENSRNOT00000050921	1.26	19.2	0.5	brwn	10871487	similar to UPF0197 protein C11orf10 homolog
LOC687502	ENSRNOT00000037433	0.77	-3.3	4.4	blue	10740560	hypothetical protein LOC687502
Nacad	NM_001100655	1.22	6.0	2.0	blue	10778351	NAC alpha domain containing
RGD1305254	ENSRNOT00000036343	1.22	6.0	13.3	turq	10723383	similar to transmembrane protein 2
RGD1306271	ENSRNOT00000040391	1.20	17.5	18.4	turq	10861946	similar to KIAA1549 protein
RGD1563437	ENSRNOT00000057655	1.31	15.8	12.0	turq	10796683	similar to KIAA1217
RGD1307051	NM_001108091	1.36	18.0	7.7	turq	10902112	similar to hypothetical protein FLJ21963
RGD1307177	NM_001163277	1.24	5.2	12.9	turq	10711906	similar to Hypothetical protein KIAA0555
RGD1308026	BC168995	1.24	9.6	2.8	blue	10908573	similar to 2310047B19Rik protein
RGD1308116	ENSRNOT00000021964	0.77	-30.2	3.2	blue	10821581	similar to hypothetical protein MGC42105
RGD1308350	ENSRNOT00000018225	1.46	18.9	19.4	turq	10887486	similar to hypothetical protein MGC13251
RGD1308616	ENSRNOT00000068128	1.23	7.1	4.6	turq	10879221	similar to KIAA0467 protein
RGD1309414	ENSRNOT00000063831	0.74	-13.4	5.1	turq	10782284	similar to KIAA0913 protein
RGD1309492	NM_001108044	1.21	20.1	2.3	blue	10886157	similar to mKIAA1737 protein
RGD1559643	NM_001109056	1.28	19.8	13.6	turq	10751868	similar to hypothetical protein A430031N04
RGD1564195	ENSRNOT00000049044	0.79	-27.6	8.3	turq	10725914	similar to hypothetical protein
RGD1564843	ENSRNOT00000046713	0.83	-7.1	2.9	blue	10875815	similar to hypothetical protein 4930474N05
Rwdd2b	NM_001100559	0.82	-15.3	0.0	grey	10752897	RWD domain containing 2B
Samd14	NM_001024966	0.81	-5.8	8.1	turq	10737596	sterile alpha motif domain containing 14
Tmem100	NM_001017479	0.80	-42.0	6.4	turq	10737426	transmembrane protein 100

Tmem119	NM_001107155	0.78	-21.7	5.2	turq	10759177	transmembrane protein 119
Tmem150c	NM_001108354	0.75	-52.3	10.4	turq	10771456	transmembrane protein 150C
Tmem178	NM_001004282	0.82	-54.8	15.8	turq	10882525	transmembrane protein 178
Tmem90b	NM_001025020	1.48	21.6	16.1	turq	10840613	transmembrane protein 90B
Tmem98	NM_001007672	1.41	11.6	1.4	turq	10736679	transmembrane protein 98
Trim21	NM_001082572	1.24	4.4	6.2	turq	10724219	tripartite motif-containing 21
Ttc9	NM_001134731	1.24	21.2	12.0	turq	10885693	tetratricopeptide repeat domain 9
Wdr90	ENSRNOT00000027121	1.20	6.0	8.6	turq	10741610	WD repeat domain 90
**ESTs**							
	**ENSRNOT00000058764**	**1.73**	**29.7**	**22.5**	**turq**	**10822752**	
	ENSRNOT00000007474	0.80	-4.2	19.6	turq	10936145	
	NM_001107319	0.67	-47.3	15.7	turq	10788668	
	ENSRNOT00000068390	1.32	43.0	15.2	turq	10796679	
	ENSRNOT00000054185	1.27	5.8	14.1	turq	10721696	
	ENSRNOT00000041961	1.36	16.0	13.8	turq	10800562	
	BC097994	0.79	-6.2	13.4	turq	10885029	
	rno-mir-151	1.35	8.6	13.2	turq	10904414	
	---	1.23	4.1	12.5	turq	10928030	
	ENSRNOT00000025092	1.46	12.8	12.3	turq	10727480	
	ENSRNOT00000061231	1.27	11.6	12.2	turq	10793891	
	GENSCAN00000039363	1.20	19.8	11.8	turq	10727226	
	GENSCAN00000003821	1.22	3.4	11.5	turq	10749973	
RGD1310951	ENSRNOT00000022866	1.22	8.2	11.0	turq	10869282	similar to RIKEN cDNA E130308A19
	ENSRNOT00000031820	0.69	-8.4	10.7	turq	10806777	
	ENSRNOT00000040484	1.21	3.2	10.1	turq	10786517	
RGD1310827	NM_001034010	0.80	-26.9	9.8	turq	10862605	similar to RIKEN cDNA 1200009O22; EST AI316813
	ENSRNOT00000012895	1.31	25.2	9.8	turq	10886062	
	---	0.79	-1.9	9.7	turq	10856543	
RGD1562037	ENSRNOT00000067963	1.32	29.3	9.5	turq	10798828	similar to OTTHUMP00000046255
	---	1.21	25.0	9.3	turq	10788687	
	---	0.78	-2.2	9.1	turq	10832326	
	---	1.20	12.2	9.0	turq	10772697	
	ENSRNOT00000059478	1.25	26.5	8.9	turq	10927668	
	ENSRNOT00000023459	1.22	25.8	7.3	turq	10917019	
	---	0.78	-18.3	7.3	turq	10776216	
	---	0.80	-12.4	7.1	turq	10755067	
	ENSRNOT00000039250	1.24	24.3	7.0	turq	10791820	
	ENSRNOT00000055652	0.80	-2.7	6.8	turq	10859436	
MGC114464	BC097418	1.27	8.3	6.7	turq	10898991	similar to expressed sequence AI836003
	GENSCAN00000026399	1.22	3.1	6.4	turq	10902020	
RGD1304595	NM_001106661	1.26	6.5	6.4	turq	10869487	similar to RIKEN cDNA 6330416G13 gene
	ENSRNOT00000058520	1.20	5.9	6.3	turq	10869737	
	ENSRNOT00000061340	0.78	-4.4	6.3	turq	10767201	
	ENSRNOT00000045241	1.25	14.9	6.2	turq	10841037	
	ENSRNOT00000047883	1.22	4.1	5.8	turq	10900109	
	ENSRNOT00000059520	0.68	-11.6	5.5	turq	10772514	
	---	0.79	-6.2	5.4	turq	10742334	
	NC_001665	1.48	151	5.1	turq	10930555	
	---	1.33	2.7	4.8	turq	10854030	
	ENSRNOT00000039392	0.81	-4.9	4.6	turq	10801071	
	ENSRNOT00000053004	0.81	-2.3	4.6	turq	10927264	
	ENSRNOT00000052516	0.83	-7.3	4.4	turq	10886866	
	ENSRNOT00000053917	0.83	-7.3	4.3	turq	10887026	
	ENSRNOT00000015145	0.81	-8.0	4.2	turq	10701902	
	ENSRNOT00000034425	1.20	4.4	4.1	turq	10821105	
	---	0.78	-2.2	3.7	turq	10925452	
	GENSCAN00000013683	1.41	23.0	3.5	turq	10917205	
	---	1.33	48.1	3.4	turq	10920628	
	GENSCAN00000034333	0.80	-7.4	3.2	turq	10781960	
	GENSCAN00000015264	1.29	15.1	3.2	turq	10901115	
LOC363337	BC082068	1.25	7.3	3.2	turq	10775260	similar to RIKEN cDNA 1700081O22
	ENSRNOT00000038558	1.31	55.8	3.0	turq	10718134	
LOC296884	NM_001177442	1.29	19.8	2.5	turq	10853707	hypothetical LOC296884
	ENSRNOT00000021834	1.21	16.4	2.3	turq	10877540	
	ENSRNOT00000053137	1.25	3.6	1.8	turq	10759459	
	ENSRNOT00000029914	0.82	-4.3	1.6	turq	10934032	
	---	1.24	5.5	1.5	turq	10908746	
	rno-mir-7a-1	1.21	8.8	1.3	turq	10793838	
	---	1.28	4.0	1.3	turq	10868414	

LOC361016	BC098040	1.24	6.8	1.3	turq	10779436	similar to RIKEN cDNA 4933406L09
	---	0.83	-40.1	0.7	turq	10723297	
RGD1309501	NM_001127537	1.21	11.4	0.3	turq	10774403	hypothetical LOC305552
RGD1564804	NM_001107969	0.82	-8.5	0.8	brwn	10879366	similar to chromosome 1 open reading frame 50
	---	0.82	-2.5	0.6	brwn	10812182	
	ENSRNOT00000061856	0.72	-22.1	0.5	brwn	10774964	
	GENSCAN00000017978	1.24	22.6	0.2	brwn	10870983	
	ENSRNOT00000053184	1.21	107	0.01	brwn	10722429	
	**ENSRNOT00000053028**	**0.39**	**-34.7**	**5.7**	**blue**	**10802970**	
	**ENSRNOT00000060749**	**0.68**	**-11.7**	**5.0**	**blue**	**10908655**	
	**ENSRNOT00000062081**	**0.81**	**-13.4**	**4.8**	**blue**	**10834071**	
	**---**	**0.47**	**-35.1**	**4.7**	**blue**	**10851484**	
	ENSRNOT00000060603	0.83	-5.9	4.4	blue	10908817	
	ENSRNOT00000005413	0.72	-9.3	4.4	blue	10895439	
	ENSRNOT00000058158	0.82	-11.3	4.2	blue	10805597	
	ENSRNOT00000057365	0.73	-34.1	3.3	blue	10924441	
	ENSRNOT00000054507	0.83	-3.5	2.8	blue	10796012	
	ENSRNOT00000063576	0.75	-107	2.7	blue	10891487	
	ENSRNOT00000058123	0.76	-7.4	2.5	blue	10902762	
	ENSRNOT00000041882	0.74	-54.3	2.0	blue	10791650	
	ENSRNOT00000012267	0.81	-4.0	1.8	blue	10875951	
	ENSRNOT00000054628	0.67	-14.5	1.2	blue	10897037	
	---	0.58	-9.6	0.9	blue	10868796	
	---	1.31	63.4	0.0	grey	10921268	
	ENSRNOT00000006439	0.82	-1.8	0.0	grey	10934733	
	ENSRNOT00000047247	0.83	-2.7	0.0	grey	10913627	
	ENSRNOT00000050109	1.21	4.3	0.0	grey	10707225	
	ENSRNOT00000052741	0.82	-5.1	0.0	grey	10726878	
	ENSRNOT00000052922	0.66	-8.8	0.0	grey	10928205	
	ENSRNOT00000052964	1.37	59.9	0.0	grey	10801135	
	ENSRNOT00000057735	0.82	-43.4	0.0	grey	10770328	
	ENSRNOT00000060756	1.23	10.6	0.0	grey	10915778	
	ENSRNOT00000063601	1.22	4.5	0.0	grey	10867975	

*** - genes belonning to top 10% of each module withing each brain region are marked by bold font**

**- Modules and k.in are given for seaparate networks; abbreviations used for modules: *turq* - turquoise, *ylw* -yellow, *brwn* - brown

Table S2-K. Differentially Expressed Genes in Female Rat Olfactory Bulbs (618 genes and ESTs)

Gene Symbol	GenBank, Ref.Sequence	Vin/Con Ratio	Vin-Con mean_dif	k.in **	Module**	ProbeSet ID	Gene Title
**Apoptosis**							
Aifm3	NM_001013977	1.24	23.9	1.9	blue	10755728	apoptosis-inducing factor, mitochondrion-associated 3
Ddx52	NM_053525	1.26	16.1	15.9	blue	10736917	DEAD (Asp-Glu-Ala-Asp) box polypeptide 52
Parm1	NM_173114	0.71	-36.5	5.3	blue	10775862	prostate androgen-regulated mucin-like protein 1
Pdcd10	NM_001009542	1.30	71.3	27.3	turq	10823733	programmed cell death 10
Prr5l	NM_001080150	1.22	2.6	4.1	blue	10847735	proline rich 5 like
Tmbim4	NM_199116	1.25	26.8	65.4	turq	10895669	transmembrane BAX inhibitor motif containing 4
**Cell Cycle**							
Anapc10	NM_001108445	0.81	-13.8	2.4	blue	10810441	anaphase promoting complex subunit 10
Cdk6	ENSRNOT00000012597	1.27	7.2	12.1	blue	10860765	cyclin-dependent kinase 6
Cenpk	NM_001106407	0.77	-10.2	16.0	blue	10812823	centromere protein K
Cenpl	NM_001033061	0.79	-5.8	14.3	blue	10765044	centromere protein L
**Cytoskeleton-ECM**							
Clec2g	NM_001048075	0.64	-40.9	5.0	blue	10859108	C-type lectin domain family 2, member g
Cno	NM_001100766	0.82	-5.1	8.2	blue	10773331	cappuccino homolog (mouse)
Col19a1	ENSRNOT00000017169	0.83	-7.1	11.8	blue	10927122	collagen, type XIX, alpha 1
Dag1	ENSRNOT00000026327	1.21	34.8	7.6	blue	10920298	dystroglycan 1 (dystrophin-associated glycoprotein 1)
Esco1	NM_001126299	0.82	-34.3	83.2	turq	10803194	establishment of cohesion 1 homolog 1 (S. cerevisiae)
Fbrs	XM_001080034	1.21	5.0	74.8	turq	10711085	fibrosin
Fibcd1	NM_001107829	1.23	5.2	0.0	grey	10844275	fibrinogen C domain containing 1
Gsn	BC079472	0.79	-6.2	11.4	blue	10835775	gelsolin
Kb23	NM_001008813	1.30	4.3	2.3	blue	10907369	type II keratin Kb23
Krtap31-1	NM_001109409	1.28	34.8	34.8	turq	10738168	keratin associated protein 31-1
Lims2	NM_001012163	1.23	3.4	8.0	blue	10800696	LIM and senescent cell antigen like domains 2
LOC494539	AY856054	1.30	9.5	87.7	turq	10722165	spectrin beta-like
Mcam	NM_023983	0.76	-8.6	7.5	blue	10909446	melanoma cell adhesion molecule
Ncan	NM_031653	0.83	-23.0	11.7	blue	10787630	neurocan
Ncdn	NM_053543	0.81	-159	14.8	blue	10879867	neurochondrin

Papln	NM_001108039	1.28	3.2	0.9	brwn	10885823	papilin, proteoglycan-like sulfated glycoprotein
Pcdhb5	NM_001114602	1.21	6.9	1.5	blue	10801174	protocadherin beta 5
Pcdhb6	NM_001014780	1.22	4.7	10.3	blue	10801176	protocadherin beta 6
RGD1563615	ENSRNOT00000046525	0.83	-5.1	6.8	blue	10793433	similar to Contactin associated protein-like 3 precursor (Cell recognition molecule Caspr3)
RGD1564680	ENSRNOT00000045270	1.34	2.8	1.7	brwn	10860966	similar to matrilin 2 precursor
Rpa3	NM_001106584	0.83	-9.1	65.8	turq	10860996	replication protein A3
Scin	NM_198748	1.21	2.9	2.0	blue	10889731	scinderin
Ska2	NM_001009624	0.77	-13.3	109.3	turq	10711496	spindle and kinetochore associated complex subunit 2
Swap70	NM_001106288	1.23	8.4	3.7	blue	10709860	SWAP-70 protein
Tbca	NM_001013245	1.25	22.0	85.8	turq	10899579	tubulin folding cofactor A
Tmsb4x	NM_031136	1.32	35.6	11.5	blue	10876291	thymosin beta 4, X-linked
Tpbg	NM_031807	1.27	11.9	4.3	blue	10912003	trophoblast glycoprotein
Trdn	NM_021666	0.81	-4.2	12.2	blue	10717359	triadin
**Tspan9**	**NM_001107890**	**0.78**	**-7.9**	**21.6**	**blue**	**10865800**	**tetraspanin 9**
Tuba1c	NM_001011995	0.80	-17.4	3.9	blue	10899055	tubulin, alpha 1C
Vcan	NM_001170558	1.22	11.7	3.4	blue	10820282	versican
**Development**							
Bai1	NM_001170597	1.21	59.7	43.8	turq	10897006	brain-specific angiogenesis inhibitor 1
Brp44l	NM_133561	0.76	-34.1	112.6	turq	10718102	brain protein 44-like
Cecr6	XM_001058567	1.27	3.5	17.1	blue	10865198	cat eye syndrome chromosome region, candidate 6 homolog (human)
Cnih4	NM_001105981	0.83	-36.7	90.5	turq	10766289	cornichon homolog 4 (Drosophila)
Cops2	NM_153297	1.26	120	45.0	turq	10849454	COP9 constitutive photomorphogenic homolog subunit 2 (Arabidopsis)
Cpne6	ENSRNOT00000024978	1.22	68.3	31.0	turq	10780343	copine VI
Fhdc1	NM_001106437	1.29	6.4	15.9	blue	10824031	FH2 domain containing 1
LOC680643	XM_001058120	1.23	7.9	8.3	blue	10791540	similar to MIC2 like 1
LRRTM1	NM_001109374	0.81	-53.4	4.1	blue	10856472	leucine rich repeat transmembrane neuronal 1
Lrrtm4	NM_001134746	0.83	-22.2	5.4	blue	10856497	leucine rich repeat transmembrane neuronal 4
Mpzl2	NM_001106818	0.79	-4.0	6.9	blue	10909590	myelin protein zero-like 2
Muted	NM_001107347	0.83	-14.8	48.2	turq	10794685	muted homolog (mouse)
Nell1	NM_031069	1.27	52.9	1.4	brwn	10707275	NEL-like 1 (chicken)
Nenf	NM_001002851	1.21	7.2	54.5	turq	10770714	neuron derived neurotrophic factor
Nphp1	NM_001106506	0.73	-27.9	9.6	blue	10849735	nephronophthisis 1 (juvenile) homolog (human)
Popdc2	NM_199113	1.23	4.0	67.0	turq	10754179	popeye domain containing 2
Reln	NM_080394	1.23	40.0	12.3	blue	10853020	reelin
Rex2	ENSRNOT00000040918	1.26	21.0	8.5	blue	10873880	reduced expression 2
Sar1b	NM_001009622	1.23	139	79.4	turq	10733389	SAR1 homolog B (S. cerevisiae)
Sema3c	NM_001106578	0.83	-8.8	62.4	turq	10860327	sema domain, immunoglobulin domain (Ig), short basic domain, secreted, (semaphorin) 3C
Sema4c	NM_001106902	1.21	3.5	16.1	blue	10927377	sema domain, immunoglobulin domain (Ig), transmembrane domain (TM) and short cytoplasm
Sntg1	ENSRNOT00000009679	0.83	-42.9	18.0	blue	10867345	syntrophin, gamma 1
Spem1	NM_001109653	1.21	2.2	2.9	blue	10744241	spermatid maturation 1
Syngr3	NM_001106985	1.21	68.1	19.5	turq	10741203	synaptogyrin 3
Tm2d3	NM_001106267	0.82	-21.7	11.0	blue	10707779	TM2 domain containing 3
Vamp8	NM_031827	1.35	13.4	53.2	turq	10863218	vesicle-associated membrane protein 8
Vangl1	NM_001109584	1.25	5.3	7.9	blue	10825512	vang-like 1 (van gogh, Drosophila)
Vof16	NM_147207	1.59	47.2	58.4	turq	10909382	ischemia related factor vof-16
Vof21	AB089205	1.42	21.7	50.3	turq	10867444	ischemia related factor vof-21
Ypel4	NM_001024369	1.24	29.2	29.3	turq	10837337	yippee-like 4 (Drosophila)
Dbndd1	NM_001014156	0.83	-3.6	0.5	ylw	10811811	dysbindin (dystrobrevin binding protein 1) domain containing 1
**Electron Transport**							
**Cox7a2l**	**NM_001106704**	**1.23**	**99**	**137.7**	**turq**	**10888127**	**cytochrome c oxidase subunit VIIa polypeptide 2 like**
Cox7b	NM_182819	1.35	85.4	79.6	turq	10934579	cytochrome c oxidase subunit VIIb
**Glrx3**	**NM_032614**	**1.23**	**45.0**	**133.0**	**turq**	**10711881**	**glutaredoxin 3**
Gpx1	NM_030826	0.82	-47.1	9.9	blue	10793103	glutathione peroxidase 1
LOC685322	NM_001170465	1.39	65.0	114.9	turq	10778108	similar to ubiquinol-cytochrome c reductase complex 7.2kDa protein isoform b
Tmem126a	NM_001011557	0.78	-13.4	48.2	turq	10723639	transmembrane protein 126A
Txn1	NM_053800	1.26	131	87.5	turq	10877005	thioredoxin 1
Ucp3	NM_013167	1.33	3.8	5.7	blue	10709083	uncoupling protein 3 (mitochondrial, proton carrier)
**Epigenetics**							
H2afz	NM_022674	0.83	-91	24.6	turq	10819322	H2A histone family, member Z
H3f3b	ENSRNOT00000004329	0.83	-100	103.9	turq	10753921	H3 histone, family 3B
Hist1h2ail	NM_001013056	0.83	-23.9	54.1	turq	10798455	histone cluster 1, H2ai-like
Hist1h2ak	NM_001109423	1.45	5.7	3.8	blue	10798507	histone cluster 1, H2ak
Hist1h2bc	NM_001109400	1.29	46.6	112.2	turq	10798488	histone cluster 1, H2bc
Hist1h2bf	ENSRNOT00000049626	0.71	-15.6	49.9	turq	10795282	histone cluster 1, H2bf
**Hist1h2bl**	**NM_022647**	**1.25**	**25.5**	**128.2**	**turq**	**10798497**	**histone cluster 1, H2bl**
Hist1h2bn	NM_001106114	0.78	-26.6	112.1	turq	10798494	histone cluster 1, H2bn
Hist1h4b	NM_022686	0.72	-200	63.8	turq	10798490	histone cluster 1, H4b
Hist2h2ab	NM_001111341	1.20	36.3	92.2	turq	10825146	histone cluster 2, H2ab
**Hist2h2bb**	**ENSRNOT00000042005**	**1.46**	**22.2**	**117.6**	**turq**	**10817539**	**histone cluster 2, H2bb**

Hist2h3c2	NM_001107698	1.22	63.2	10.9	blue	10817537	histone cluster 2, H3c2
Hist2h4	NM_001123469	1.53	133	71.4	turq	10825151	histone cluster 2, H4
Hist3h2ba	NM_001111127	1.26	40.7	84.8	turq	10733938	histone cluster 3, H2ba
L3mbtl2	NM_001033695	1.23	16.2	89.5	turq	10897952	l(3)mbt-like 2 (Drosophila)
LOC679840	XM_001054684	0.71	-31.1	49.6	turq	10798442	similar to germinal histone H4 gene
LOC682649	ENSRNOT00000048748	1.24	61.8	48.8	turq	10795265	similar to Histone H2A type 1
LOC685909	NM_001106019	0.81	-22.8	116.4	turq	10805582	similar to H2A histone family, member V isoform 1
N5	NM_022857	0.76	-7.8	57.6	turq	10861560	DNA binding protein N5
Pygo2	NM_001106447	1.24	11.9	82.1	turq	10816801	pygopus 2
Snhg8	ENSRNOT00000061204	0.82	-3.2	9.4	blue	10790016	small nucleolar RNA host gene (non-protein coding) 8
**Golgi Apparatus**							
B3galt2	NM_001109492	0.73	-95.8	27.2	turq	10764495	UDP-Gal:betaGlcNAc beta 1,3-galactosyltransferase, polypeptide 2
Gga3	NM_001108304	0.77	-19.4	11.7	blue	10748973	golgi associated, gamma adaptin ear containing, ARF binding protein 3
Pigs	NM_001006602	1.21	12.3	10.2	blue	10745081	phosphatidylinositol glycan anchor biosynthesis, class S
St6gal2	AJ627626	1.23	30.5	79.2	turq	10930711	ST6 beta-galactosamide alpha-2,6-sialyltranferase 2
Stard10	NM_001013069	0.82	-32.9	12.8	blue	10709157	StAR-related lipid transfer (START) domain containing 10
Stard9	ENSRNOT00000048141	1.21	6.7	7.9	blue	10839050	StAR-related lipid transfer (START) domain containing 9
**Growth Factor**							
**Dner**	**BC086329**	**1.25**	**91.0**	**19.2**	**blue**	**10929482**	**delta/notch-like EGF repeat containing**
Cetn3	ENSRNOT00000021735	1.26	55.0	36.8	turq	10812346	centrin, EF-hand protein, 3 (CDC31 homolog, yeast)
Cgrrf1	NM_053899	0.79	-14.8	50.9	turq	10779646	cell growth regulator with ring finger domain 1
Cxcl13	NM_001017496	0.69	-6.4	15.5	blue	10775731	chemokine (C-X-C motif) ligand 13
**Gdf1**	**NM_001044240**	**0.81**	**-25.4**	**21.2**	**blue**	**10791011**	**growth differentiation factor 1**
Gfer	NM_013222	0.82	-9.1	10.9	blue	10741208	growth factor, augmenter of liver regeneration
Igf2	NM_031511	0.73	-9.9	3.5	blue	10726999	insulin-like growth factor 2
Megf8	ENSRNOT00000027831	1.25	14.2	38.0	turq	10705142	multiple EGF-like-domains 8
Ogn	NM_001106103	0.62	-168	5.9	blue	10797648	osteoglycin
RGD1307225	NM_001107663	0.71	-87.2	56.8	turq	10814540	similar to MEGF6
Tgfb3	NM_013174	0.71	-11.3	6.6	blue	10891303	transforming growth factor, beta 3
Vegfa	NM_031836	1.21	20.9	10.1	blue	10921772	vascular endothelial growth factor A
**Immune Response**							
Cd24	NM_012752	0.78	-42.9	2.5	blue	10830624	CD24 molecule
**Cd48**	**NM_139103**	**1.38**	**77.8**	**121.0**	**turq**	**10765634**	**Cd48 molecule**
Cd83	NM_001108410	1.20	8.8	8.1	blue	10797811	CD83 molecule
Fcgr2b	NM_175756	0.79	-7.7	5.5	blue	10769771	Fc fragment of IgG, low affinity IIb, receptor (CD32)
Ifitm2	NM_030833	0.69	-17.2	10.7	blue	10726679	interferon induced transmembrane protein 2
Igdcc3	NM_001108160	1.23	4.6	17.9	blue	10910895	immunoglobulin superfamily, DCC subclass, member 3
LOC287167	NM_001013853	0.71	-34.1	3.1	blue	10741765	globin, alpha
Ly6g6e	NM_001001972	1.33	5.9	0.5	brwn	10831229	lymphocyte antigen 6 complex, locus G6E
RT1-S3	NM_001008886	0.81	-4.9	1.7	blue	10827809	RT1 class Ib, locus S3
Spag6	NM_001034960	0.76	-10.0	0.3	ylw	10752563	sperm associated antigen 6
**Metabolism & Transport**							
Acn9	NM_001047914	1.33	16.8	100.9	turq	10853676	ACN9 homolog (S. cerevisiae)
Akr1c14	NM_138547	0.65	-9.8	6.8	blue	10799346	aldo-keto reductase family 1, member C14
**Arsb**	**ENSRNOT00000014860**	**1.22**	**91.0**	**21.2**	**blue**	**10812524**	**arylsulfatase B**
Atox1	NM_053359	0.80	-12.4	92.2	turq	10742813	ATX1 antioxidant protein 1 homolog (yeast)
**Atp11a**	**NM_001107324**	**1.31**	**45.0**	**22.4**	**blue**	**10792859**	**ATPase, class VI, type 11A**
**Atp5e**	**NM_139099**	**1.26**	**101**	**144.8**	**turq**	**10773636**	**ATP synthase, H+ transporting, mitochondrial F1 complex, epsilon subunit**
Atp5j2	ENSRNOT00000033537	1.23	264	107.5	turq	10756546	ATP synthase, H+ transporting, mitochondrial F0 complex, subunit F2
Atp6v0a1	NM_031604	0.83	-140	12.3	blue	10738237	ATPase, H+ transporting, lysosomal V0 subunit A1
Chst12	NM_001037775	1.22	5.1	13.5	blue	10760605	carbohydrate (chondroitin 4) sulfotransferase 12
Clcn7	NM_031568	0.83	-17.0	35.5	turq	10732302	chloride channel 7
Clns1a	NM_031719	1.23	52.1	94.9	turq	10708824	chloride channel, nucleotide-sensitive, 1A
Cmc1	NM_001135259	1.30	4.0	75.2	turq	10920797	COX assembly mitochondrial protein homolog (S. cerevisiae)
Cpt1c	NM_001034925	1.24	23.9	48.7	turq	10721563	carnitine palmitoyltransferase 1c
Ddc	NM_012545	1.24	4.2	0.7	ylw	10778486	dopa decarboxylase (aromatic L-amino acid decarboxylase)
Dhrs3	NM_001037199	1.22	18.3	1.8	blue	10873838	dehydrogenase/reductase (SDR family) member 3
Dio2	NM_031720	1.24	12.9	4.1	blue	10891402	deiodinase, iodothyronine, type II
Elovl1	BC085795	1.21	34.1	17.4	blue	10871479	elongation of very long chain fatty acids (FEN1/Elo2, SUR4/Elo3, yeast)-like 1
Enkur	NM_001106126	0.76	-19.3	13.8	blue	10799977	enkurin, TRPC channel interacting protein
Exoc8	NM_139043	1.21	15.8	16.9	blue	10811947	exocyst complex component 8
Extl3	NM_020097	0.80	-40.6	9.8	blue	10784540	exostoses (multiple)-like 3
G6pc3	NM_176077	1.23	32.0	11.5	blue	10738530	glucose 6 phosphatase, catalytic, 3
**Galntl6**	**NM_001135756**	**1.28**	**101**	**19.3**	**blue**	**10787962**	**UDP-N-acetyl-alpha-D-galactosamine:polypeptide N-acetylgalactosaminyltransferase-like 6**
Gdpd1	NM_001044238	0.83	-51.0	104.7	turq	10746000	glycerophosphodiester phosphodiesterase domain containing 1
Gpld1	NM_001100512	1.22	25.1	12.9	blue	10798331	glycosylphosphatidylinositol specific phospholipase D1
Gsto1	NM_001007602	0.83	-66.3	15.1	blue	10715990	glutathione S-transferase omega 1
Guk1	NM_001013115	0.83	-61.6	36.5	turq	10743110	guanylate kinase 1
Hba-a2	NM_013096	0.68	-639	5.7	blue	10741778	hemoglobin alpha, adult chain 2

Hpgds	NM_031644	1.21	5.9	23.3	turq	10862842	hematopoietic prostaglandin D synthase
Iah1	NM_001100540	0.83	-21.6	22.0	turq	10883858	isoamyl acetate-hydrolyzing esterase 1 homolog (S. cerevisiae)
Inmt	NM_001109022	0.73	-5.9	71.6	turq	10862694	indolethylamine N-methyltransferase
Kcnh5	NM_133610	2.40	142	2.8	blue	10890609	potassium voltage-gated channel, subfamily H (eag-related), member 5
Kcnn3	NM_019315	1.23	24.5	1.0	brwn	10816824	potassium intermediate/small conductance calcium-activated channel, subfamily N, member 3
Kcnt2	NM_198762	0.77	-40.5	3.6	blue	10764460	potassium channel, subfamily T, member 2
Kcnv1	NM_021697	0.77	-51.0	17.8	blue	10903585	potassium channel, subfamily V, member 1
Ldha	NM_017025	0.81	-99.9	84.1	turq	10707137	lactate dehydrogenase A
Lin7a	NM_053514	0.79	-32.1	92.2	turq	10895303	lin-7 homolog a (C. elegans)
LOC100188932	NM_001134691	1.20	142	53.2	turq	10883219	dolichyl-diphosphooligosaccharide--protein glycosyltransferase subunit 4
LOC682386	ENSRNOT00000027244	0.75	-13.2	53.2	turq	10803759	similar to Nucleoside diphosphate kinase homolog 5 (NDK-H 5) (NDP kinase homolog 5)
LOC688972	ENSRNOT00000059890	0.77	-3.0	8.9	blue	10810434	similar to Glycophorin
Lyz2	NM_012771	0.63	-44.3	12.2	blue	10902547	lysozyme 2
Mfsd6	NM_001106911	0.81	-29.7	5.8	blue	10923191	major facilitator superfamily domain containing 6
MGC72973	NM_198776	0.51	-263	14.1	blue	10724319	beta-glo
**Nat5**	**NM_001108595**	**0.76**	**-35.0**	**123.4**	**turq**	**10840460**	**N-acetyltransferase 5**
**Ndufa2**	**NM_001106153**	**1.54**	**27.7**	**133.7**	**turq**	**10803995**	**NADH dehydrogenase (ubiquinone) 1 alpha subcomplex, 2**
Ndufa6	NM_001130505	1.30	51.6	103.2	turq	10905716	NADH dehydrogenase (ubiquinone) 1 alpha subcomplex, 6 (B14)
Ndufb7	NM_001108442	0.81	-31.9	64.5	turq	10810299	NADH dehydrogenase (ubiquinone) 1 beta subcomplex, 7
Nkain4	NM_001106550	0.80	-42.2	56.2	turq	10852421	Na+/K+ transporting ATPase interacting 4
Ostc	NM_001108566	0.77	-38.9	61.7	turq	10826814	oligosaccharyltransferase complex subunit
Pigk	NM_001011953	1.23	39.6	101.1	turq	10819816	phosphatidylinositol glycan anchor biosynthesis, class K
Pigx	NM_001100651	0.77	-36.3	73.8	turq	10751664	phosphatidylinositol glycan anchor biosynthesis, class X
Pld5	ENSRNOT00000005332	0.71	-19.5	7.4	blue	10770161	phospholipase D family, member 5
Pter	NM_022224	0.81	-6.7	4.9	blue	10796440	phosphotriesterase related
Ptgds	NM_013015	0.72	-393	4.5	blue	10843460	prostaglandin D2 synthase (brain)
Selenbp1	NM_080892	0.82	-8.1	3.0	blue	10824892	selenium binding protein 1
Sh3bgrl	NM_001173339	1.27	121	103.6	turq	10934669	SH3 domain binding glutamic acid-rich protein like
Slc15a3	NM_139341	1.21	5.7	4.0	blue	10713974	solute carrier family 15, member 3
Slc19a3	NM_001108228	1.21	3.2	0.5	ylw	10929450	solute carrier family 19, member 3
**Slc20a1**	**NM_031148**	**1.21**	**88.2**	**18.0**	**blue**	**10839771**	**solute carrier family 20 (phosphate transporter), member 1**
Slc33a1	NM_022252	1.20	36.8	18.3	turq	10823462	solute carrier family 33 (acetyl-CoA transporter), member 1
**Slc3a2**	**NM_019283**	**1.22**	**169**	**20.7**	**blue**	**10728507**	**solute carrier family 3 (activators of dibasic and neutral amino acid transport), member 2**
Slc44a2	NM_001134715	1.34	19.0	16.0	blue	10908391	solute carrier family 44, member 2
Slc4a4	NM_053424	1.22	121	15.4	blue	10775997	solute carrier family 4 (anion exchanger), member 4
Slc4a8	NM_199497	0.83	-14.2	16.0	blue	10899278	solute carrier family 4 (anion exchanger), member 8
Slc6a7	NM_053996	1.28	15.3	12.8	blue	10804632	solute carrier family 6 (neurotransmitter transporter, L-proline), member 7
Slc6a9	NM_053818	1.22	41.9	17.5	blue	10871444	solute carrier family 6 (neurotransmitter transporter, glycine), member 9
Slc7a3	NM_017217	1.48	20.0	3.7	blue	10938635	solute carrier family 7 (cationic amino acid transporter, y+ system), member 3
Slc7a5	NM_017353	0.74	-46.7	12.9	blue	10811531	solute carrier family 7 (cationic amino acid transporter, y+ system), member 5
Slc7a8	NM_053442	0.77	-32.2	90.4	turq	10783648	solute carrier family 7 (cationic amino acid transporter, y+ system), member 8
Slco3a1	NM_177481	1.28	19.8	11.9	blue	10722818	solute carrier organic anion transporter family, member 3a1
**Proteolysis**							
Adam23	NM_001029899	1.28	54.7	2.0	brwn	10923877	ADAM metallopeptidase domain 23
**Cstb**	**NM_012838**	**0.75**	**-64.9**	**130.5**	**turq**	**10832228**	**cystatin B (stefin B)**
Psmb3	NM_017285	0.79	-30.5	54.2	turq	10737897	proteasome (prosome, macropain) subunit, beta type 3
RGD1565317	ENSRNOT00000017230	1.34	21.0	13.1	blue	10736332	similar to ubiquitin-like/S30 ribosomal fusion protein
Rnf213	ENSRNOT00000004904	1.21	7.2	5.2	blue	10739984	ring finger protein 213
**Uqcrq**	**NM_001025134**	**0.78**	**-76.0**	**121.5**	**turq**	**10742601**	**ubiquinol-cytochrome c reductase, complex III subunit VII**
Usp1	NM_001015015	0.78	-20.9	70.3	turq	10870098	ubiquitin specific peptidase 1
Usp21	NM_001127638	1.23	25.7	17.1	blue	10769854	ubiquitin specific peptidase 21
**Receptors & Binding Proteins**							
Abca8	ENSRNOT00000065947	0.80	-7.6	6.2	blue	10748564	ATP-binding cassette, sub-family A (ABC1), member 8
Abcb4	NM_012690	1.31	11.9	12.2	blue	10853347	ATP-binding cassette, sub-family B (MDR/TAP), member 4
Crabp1	NM_001105716	0.78	-8.6	29.8	turq	10910089	cellular retinoic acid binding protein 1
**Crabp2**	**NM_017244**	**0.65**	**-19.1**	**18.7**	**blue**	**10816405**	**cellular retinoic acid binding protein 2**
**Ednrb**	**NM_017333**	**1.25**	**157**	**24.5**	**blue**	**10785724**	**endothelin receptor type B**
Egfl8	NM_001165880	1.20	3.7	3.9	blue	10828303	EGF-like-domain, multiple 8
Frzb	NM_001100527	1.24	9.2	5.2	blue	10846740	frizzled-related protein
Fubp3	NM_001039337	1.21	16.5	26.6	turq	10835371	far upstream element (FUSE) binding protein 3
Grm1	NM_001114330	0.77	-30.8	69.1	turq	10716789	glutamate receptor, metabotropic 1
Grm2	NM_001105711	1.23	11.2	6.7	blue	10919953	glutamate receptor, metabotropic 2
Igfbp2	NM_013122	0.66	-22.1	6.7	blue	10924223	insulin-like growth factor binding protein 2
Igfbp6	NM_013104	0.81	-6.5	0.9	brwn	10899465	insulin-like growth factor binding protein 6
Itgb3bp	NM_001013213	0.81	-7.0	17.7	blue	10878272	integrin beta 3 binding protein (beta3-endonexin)
Leng4	NM_001134978	0.72	-72.6	14.2	blue	10703715	leukocyte receptor cluster (LRC) member 4
**Leng8**	**NM_001037790**	**1.38**	**14.1**	**118.1**	**turq**	**10719002**	**leukocyte receptor cluster (LRC) member 8**
Lrp4	NM_031322	0.82	-22.8	8.1	blue	10837881	low density lipoprotein receptor-related protein 4
Npy2r	NM_023968	1.21	5.8	15.4	blue	10823937	neuropeptide Y receptor Y2

Nrp1	NM_145098	1.23	16.1	7.2	blue	10808959	neuropilin 1
Ntsr1	NM_001108967	1.25	4.9	9.6	blue	10842828	neurotensin receptor 1
Olr1059	NM_001001362	0.83	-5.0	8.2	turq	10900085	olfactory receptor 1059
Olr1571	ENSRNOT00000034210	0.81	-2.5	0.5	ylw	10752574	olfactory receptor 1571
Olr635	NM_001000646	0.83	-2.3	0.8	blue	10837586	olfactory receptor 635
Olr648	NM_001000641	0.82	-1.9	2.8	blue	10837604	olfactory receptor 648
Olr729	NM_001000574	0.80	-2.6	4.7	blue	10847235	olfactory receptor 729
Olr796	NM_001000600	0.81	-4.3	2.2	blue	10838449	olfactory receptor 796
Plxdc1	NM_001107046	1.35	4.3	5.8	blue	10746804	plexin domain containing 1
Plxnb3	NM_001135878	1.21	5.9	74.5	turq	10935962	plexin B3
Pvalb	NM_022499	1.53	62.0	70.3	turq	10905277	parvalbumin
Ranbp3l	ENSRNOT00000050942	0.76	-19.6	3.4	blue	10813563	RAN binding protein 3-like
Rbp1	NM_012733	0.79	-22.4	4.9	blue	10912439	retinol binding protein 1, cellular
Rbp4	NM_013162	0.80	-10.7	6.1	blue	10729970	retinol binding protein 4, plasma
Ryr1l	ENSRNOT00000064639	1.27	4.9	93.0	turq	10720308	ryanodine receptor 1-like
S100a10	NM_031114	1.24	48.1	59.5	turq	10817186	S100 calcium binding protein A10
Scfd1	NM_019364	0.81	-58.5	90.3	turq	10884505	sec1 family domain containing 1
Sec61g	NM_001135020	0.73	-45.8	95.9	turq	10778555	SEC61, gamma subunit
**Stxbp5**	**NM_178345**	**1.21**	**54.7**	**18.4**	**blue**	**10716712**	**syntaxin binding protein 5 (tomosyn)**
Tmem151a	NM_001107570	0.79	-24.6	8.8	blue	10727725	transmembrane protein 151A
Unc5c	NM_199407	1.22	16.4	16.2	blue	10819469	unc-5 homolog C (C. elegans)
Vom2r66	ENSRNOT00000051621	1.42	51.5	30.6	turq	10774903	vomeronasal 2 receptor, 66
Wbp5	NM_001127502	0.78	-25.2	81.6	turq	10935043	WW domain binding protein 5
**Signaling**							
Adcy8	NM_017142	1.29	30.6	10.6	blue	10904063	adenylate cyclase 8 (brain)
**Adcyap1r1**	**NM_133511**	**0.80**	**-92.1**	**128.6**	**turq**	**10855727**	**adenylate cyclase activating polypeptide 1 (pituitary) receptor type I**
Anxa2	NM_019905	0.67	-23.4	7.6	blue	10911287	annexin A2
Aurkc	ENSRNOT00000021245	1.26	6.5	100.6	turq	10718658	aurora kinase C
Bccip	NM_001108505	1.26	39.4	65.9	turq	10711769	BRCA2 and CDKN1A interacting protein
**Calml3**	**BC086350**	**0.79**	**-12.0**	**18.5**	**blue**	**10796092**	**calmodulin-like 3**
**Dennd3**	**ENSRNOT00000014541**	**1.25**	**4.7**	**18.9**	**blue**	**10896966**	**DENN/MADD domain containing 3**
Dgkg	NM_013126	1.20	28.0	12.2	blue	10752007	diacylglycerol kinase, gamma
Efnb3	NM_001100980	0.71	-20.2	8.5	blue	10744081	ephrin B3
Entpd2	NM_172030	1.33	27.5	6.8	blue	10834213	ectonucleoside triphosphate diphosphohydrolase 2
Entpd6	NM_053498	1.25	39.6	15.4	blue	10840684	ectonucleoside triphosphate diphosphohydrolase 6
Fgd5	NM_001108637	1.36	8.5	1.9	brwn	10857382	FYVE, RhoGEF and PH domain containing 5
Gnb2l1	NM_130734	0.82	-145	24.9	turq	10733039	guanine nucleotide binding protein (G protein), beta polypeptide 2 like 1
Gng5	NM_024377	1.25	23.8	36.3	turq	10704486	guanine nucleotide binding protein (G protein), gamma 5
Gpr116	NM_139110	1.23	8.0	12.1	blue	10926698	G protein-coupled receptor 116
Gpr165	NM_001106582	1.21	14.5	0.0	grey	10934098	G protein-coupled receptor 165
Gpr34	NM_001024925	1.25	42.2	57.3	turq	10936742	G protein-coupled receptor 34
Grin2c	NM_012575	1.25	11.4	26.2	turq	10748891	glutamate receptor, ionotropic, N-methyl D-aspartate 2C
Gtpbp2	NM_001013225	1.20	7.1	0.0	ylw	10926549	GTP binding protein 2
Itfg2	NM_001108648	1.28	9.3	92.3	turq	10865841	integrin alpha FG-GAP repeat containing 2
Itga7	NM_030842	1.25	6.8	10.4	blue	10893267	integrin, alpha 7
LOC686547	ENSRNOT00000064942	1.21	7.3	111.1	turq	10785618	similar to TBC1 domain family member 4 (Akt substrate of 160 kDa) (AS160)
Mab21l1	ENSRNOT00000040631	0.76	-5.4	115.7	turq	10815500	mab-21-like 1 (C. elegans)
Magmas	NM_001100136	0.68	-18.1	8.3	blue	10731783	mitochondria-associated protein involved in granulocyte-macrophage colony-stimulating factor
Mapk1ip1l	NM_001108373	1.24	32.4	65.9	turq	10779671	mitogen-activated protein kinase 1 interacting protein 1-like
Notch3	NM_020087	1.22	4.1	15.1	blue	10900907	Notch homolog 3 (Drosophila)
**Nrgn**	**NM_024140**	**1.26**	**153**	**2.3**	**brwn**	**10916228**	**neurogranin**
Ppp1r1a	NM_022676	0.80	-23.0	8.7	blue	10907738	protein phosphatase 1, regulatory (inhibitor) subunit 1A
Ppp2r3c	NM_001014196	1.28	29.2	43.8	turq	10890003	protein phosphatase 2, regulatory subunit B'', gamma
Rab32	NM_001108902	0.82	-2.9	2.1	blue	10716785	RAB32, member RAS oncogene family
Ramp2	NM_031646	1.26	10.1	11.1	blue	10738341	receptor (G protein-coupled) activity modifying protein 2
Ret	NM_012643	1.23	6.2	8.0	blue	10864918	ret proto-oncogene
Rgs17	NM_001107459	0.79	-34.9	51.1	turq	10717793	regulator of G-protein signaling 17
Rgs2	NM_053453	0.80	-36.8	12.2	blue	10768332	regulator of G-protein signaling 2
Rhoc	NM_001106461	0.82	-13.5	108.8	turq	10818108	ras homolog gene family, member C
Rhog	NM_001037195	1.25	32.7	17.5	blue	10724208	ras homolog gene family, member G (rho G)
Samsn1	NM_130821	1.35	6.6	5.5	blue	10752744	SAM domain, SH3 domain and nuclear localization signals, 1
Sfrp1	ENSRNOT00000024128	0.74	-6.6	2.2	blue	10792344	secreted frizzled-related protein 1
Stk3	NM_031735	0.82	-23.2	50.1	turq	10903246	serine/threonine kinase 3 (STE20 homolog, yeast)
Tank	NM_001164073	0.80	-25.3	14.7	blue	10836404	TRAF family member-associated NFKB activator
Tek	NM_001105737	1.27	6.6	3.5	blue	10869946	TEK tyrosine kinase, endothelial
Tpte	NM_001108877	1.55	6.4	8.1	blue	10792456	transmembrane phosphatase with tensin homology
Trib1	NM_023985	0.80	-7.6	10.8	blue	10896793	tribbles homolog 1 (Drosophila)
**Transcription**							
Anks1b	ENSRNOT00000064242	0.74	-54.2	87.5	turq	10894810	ankyrin repeat and sterile alpha motif domain containing 1B

Ccdc46	NM_001105849	1.43	22.9	41.0	turq	10739277	coiled-coil domain containing 46
Ccdc86	NM_001006974	0.81	-6.1	5.5	blue	10728830	coiled-coil domain containing 86
Dnajc30	NM_001109024	0.77	-6.1	4.6	blue	10761225	DnaJ (Hsp40) homolog, subfamily C, member 30
Duxbl	ENSRNOT00000052224	1.21	2.7	12.1	blue	10786197	double homeobox B-like
Fam134b	ENSRNOT00000014423	0.78	-9.5	9.9	blue	10813949	family with sequence similarity 134, member B
Fbxo15	NM_001108436	0.78	-20.6	43.9	turq	10803025	F-box protein 15
Fosb	ENSRNOT00000022556	1.40	11.2	12.0	blue	10719432	FBJ osteosarcoma oncogene B
Kank1	NM_001037197	1.23	18.0	0.0	grey	10714505	KN motif and ankyrin repeat domains 1
Kbtbd3	NM_001108121	0.83	-38.6	101.5	turq	10907806	kelch repeat and BTB (POZ) domain containing 3
Klrc2	NM_019261	0.83	-1.9	5.2	blue	10866056	killer cell lectin-like receptor subfamily C, member 2
LOC100363462	XR_086055	1.42	18.3	11.7	blue	10796917	zinc finger protein 107-like
LOC499124	NM_001100991	0.81	-12.1	5.5	blue	10720539	mouse zinc finger protein 14-like
LOC683241	XM_001065806	1.39	22.2	64.1	turq	10708984	similar to Finkel-Biskis-Reilly murine sarcoma virusubiquitously expressed
40239	NM_001034108	0.76	-33.0	74.0	turq	10901152	membrane-associated ring finger (C3HC4) 2
Nip7	NM_138847	0.78	-13.1	10.1	blue	10807625	nuclear import 7 homolog (S. cerevisiae)
Nr4a1	NM_024388	0.66	-51.7	20.0	turq	10899387	nuclear receptor subfamily 4, group A, member 1
Nupr1	NM_053611	0.67	-24.1	6.2	blue	10725778	nuclear protein, transcriptional regulator, 1
Ptchd2	NM_001107992	1.22	6.1	17.6	blue	10881596	patched domain containing 2
**Rag1ap1**	**NM_001106445**	**1.26**	**22.2**	**18.6**	**blue**	**10824434**	**recombination activating gene 1 activating protein 1**
RGD1311064	ENSRNOT00000040609	0.83	-22.8	15.7	blue	10703495	similar to KRAB zinc finger protein KR18
RGD1561241	ENSRNOT00000005121	0.76	-24.2	66.8	turq	10773047	similar to Mblk1-related protein-1
RGD1563216	NM_001109278	0.77	-4.0	77.2	turq	10885959	similar to HESB like domain containing 1
Stx2	NM_012748	1.21	8.8	73.1	turq	10758050	syntaxin 2
Th	NM_012740	1.25	5.5	3.3	blue	10727011	tyrosine hydroxylase
Tiam1	NM_001100558	1.20	37.4	70.3	turq	10750144	T-cell lymphoma invasion and metastasis 1
Zcwpw2	ENSRNOT00000055976	0.81	-12.1	94.8	turq	10920787	zinc finger, CW type with PWWP domain 2
Zdhhc23	NM_213627	0.82	-11.1	3.5	blue	10751190	zinc finger, DHHC-type containing 23
Zfp212	ENSRNOT00000009054	1.21	7.7	9.4	blue	10855203	Zinc finger protein 212
Zfp35	NM_001013141	1.31	32.1	73.1	turq	10800493	zinc finger protein 35
Zfp472	NM_182823	0.83	-5.3	7.9	blue	10894221	zinc finger protein 472
**Zfp709l1**	**ENSRNOT00000042498**	**1.27**	**93.1**	**19.7**	**blue**	**10893420**	**zinc finger protein 709-like 1**
Zfp748	ENSRNOT00000022527	0.76	-46.0	83.5	turq	10796904	zinc finger protein 748
Zfx	NM_001109017	0.81	-45.3	8.2	blue	10938396	zinc finger protein X-linked
Znf292	NM_001008879	0.83	-65.9	17.1	blue	10875936	zinc finger protein 292
**Translation & Protein Modification**							
Snrnp25	ENSRNOT00000067079	1.29	10.4	84.4	turq	10741793	small nuclear ribonucleoprotein 25 (U11/U12)
**Eef1b2**	**NM_001108799**	**1.22**	**33.9**	**118.3**	**turq**	**10923857**	**eukaryotic translation elongation factor 1 beta 2**
Eif3e	NM_001011990	1.27	118	86.7	turq	10903545	eukaryotic translation initiation factor 3, subunit E
Eif3m	NM_001168523	1.28	105	10.5	blue	10847965	eukaryotic translation initiation factor 3, subunit M
LOC499485	XM_002725892	1.22	16.5	103.1	turq	10820047	similar to 60S ribosomal protein L17 (L23) (Amino acid starvation-induced protein) (ASI)
LOC499782	NM_001109198	0.83	-106	60.2	turq	10902467	similar to 60S ribosomal protein L12
**LOC681338**	**ENSRNOT00000047517**	**1.31**	**2.2**	**118.4**	**turq**	**10928032**	**similar to ribosomal protein L31**
LOC684806	ENSRNOT00000051764	1.30	23.4	92.4	turq	10781564	similar to 40S ribosomal protein S29
LOC688574	ENSRNOT00000043132	1.25	12.0	1.6	blue	10720555	similar to DNA-directed RNA polymerase II largest subunit (RPB1)
LOC688981	ENSRNOT00000046585	1.33	6.7	12.7	blue	10890597	similar to 60S ribosomal protein L26 (Silica-induced gene 20 protein) (SIG-20)
**Mrpl22**	**NM_001105781**	**1.27**	**11.7**	**18.9**	**blue**	**10733782**	**mitochondrial ribosomal protein L22**
Msi2	ENSRNOT00000003164	1.21	21.0	78.8	turq	10746114	Musashi homolog 2 (Drosophila)
Rbm4	ENSRNOT00000026621	0.82	-19.5	11.9	blue	10727643	RNA binding motif protein 4
Rbm46	NM_001135717	1.23	5.0	1.3	brwn	10823941	RNA binding motif protein 46
RGD1559743	XM_001077121	1.23	2.5	3.1	blue	10920785	similar to 40S ribosomal protein S16
RGD1561984	ENSRNOT00000041555	1.23	25.0	111.0	turq	10917299	similar to ribosomal protein L27a
RGD1562547	ENSRNOT00000049211	1.29	5.5	79.2	turq	10901960	similar to ribosomal protein L31
RGD1565415	ENSRNOT00000041621	0.82	-29.6	7.5	blue	10862499	similar to ribosomal protein L27a
Sfrs12ip1	NM_001008373	0.83	-22.4	7.4	blue	10812865	SFRS12-interacting protein 1
Taf9	NM_001037310	0.83	-34.9	71.3	turq	10812748	TAF9 RNA polymerase II, TATA box binding protein (TBP)-associated factor
Arl4a	NM_019186	1.36	21.3	38.9	turq	10889728	ADP-ribosylation factor-like 4A
Arl4d	NM_001107052	1.35	15.4	5.4	blue	10738477	ADP-ribosylation factor-like 4D
Cstf2t	NM_001107586	1.22	13.6	32.9	turq	10714788	cleavage stimulation factor, 3' pre-RNA subunit 2, tau
Gemin6	NM_001009466	0.81	-7.7	24.0	turq	10882507	gem (nuclear organelle) associated protein 6
Gucy2f	L36030	0.76	-13.5	16.5	blue	10932836	guanylate cyclase 2F
Hnrpm	NM_001109911	1.24	63.6	114.7	turq	10901142	heterogeneous nuclear ribonucleoprotein M
Mrpl30	NM_001106903	1.21	16.4	79.0	turq	10922695	mitochondrial ribosomal protein L30
Mrpl34	NM_001006965	0.83	-13.1	8.8	turq	10787281	mitochondrial ribosomal protein L34
Mrpl44	NM_001031650	0.82	-6.9	4.6	blue	10924694	mitochondrial ribosomal protein L44
Mrpl48	NM_001106282	0.80	-43.0	72.7	turq	10723982	mitochondrial ribosomal protein L48
Pfdn1	NM_001108427	1.32	46.7	108.4	turq	10803943	prefoldin subunit 1
**Rpl11**	**NM_001025739**	**0.81**	**-237**	**18.8**	**blue**	**10880678**	**ribosomal protein L11**
Rpl13	NM_031101	0.81	-79	82.4	turq	10774102	ribosomal protein L13
Rpl15	NM_139114	0.79	-144	92.3	turq	10801783	ribosomal protein L15

Rpl17	NM_201415	0.81	-364	103.0	turq	10802700	ribosomal protein L17
Rpl18a	NM_212510	0.83	-254	47.3	turq	10787396	ribosomal protein L18A
**Rpl19**	**NM_031103**	**0.81**	**-221**	**134.6**	**turq**	**10737920**	**ribosomal protein L19**
**Rpl22**	**NM_031104**	**1.31**	**56**	**120.7**	**turq**	**10863772**	**ribosomal protein L22**
Rpl22l1	NM_001108548	1.34	57	75.5	turq	10839803	ribosomal protein L22 like 1
Rpl23	NM_001007599	0.80	-35	101.6	turq	10746783	ribosomal protein L23
Rpl23a	NM_001108283	0.82	-182	103.2	turq	10736232	ribosomal protein L23a
Rpl24	NM_022515	0.80	-24.3	9.3	blue	10842667	ribosomal protein L24
**Rpl26**	**NM_001105788**	**1.44**	**193**	**153.6**	**turq**	**10905558**	**ribosomal protein L26**
Rpl27	NM_022514	0.82	-29.1	94.9	turq	10938579	ribosomal protein L27
**Rpl27a**	**NM_001106290**	**1.24**	**97**	**147.5**	**turq**	**10706219**	**ribosomal protein L27a**
Rpl29	NM_017150	0.82	-119	72.8	turq	10727922	ribosomal protein L29
**Rpl31**	**NM_022506**	**1.24**	**234**	**119.0**	**turq**	**10877446**	**ribosomal protein L31**
Rpl32	NM_013226	0.83	-205	113.6	turq	10892702	ribosomal protein L32
**Rpl34**	**NM_001108567**	**0.80**	**-241**	**22.3**	**blue**	**10826818**	**ribosomal protein L34**
**Rpl35a**	**NM_021264**	**1.24**	**110**	**120.7**	**turq**	**10760018**	**ribosomal protein L35a**
Rpl36	NM_022504	1.23	45.9	114.4	turq	10931410	ribosomal protein L36
**Rpl36a**	**NM_001128065**	**1.35**	**381**	**137.7**	**turq**	**10770410**	**ribosomal protein L36a**
Rpl36al	NM_031105	1.29	45.5	87.1	turq	10890243	ribosomal protein L36a-like
Rpl37	NM_031106	1.21	250	59.0	turq	10923855	ribosomal protein L37
**Rpl39**	**NM_012875**	**1.46**	**150**	**140.3**	**turq**	**10803301**	**ribosomal protein L39**
**Rpl41**	**NM_139083**	**1.43**	**269**	**124.4**	**turq**	**10774894**	**ribosomal protein L41**
Rpl7	NM_001100534	0.79	-347	94.1	turq	10867099	ribosomal protein L7
Rpp14	NM_001108372	0.76	-43.6	64.4	turq	10782689	ribonuclease P 14 subunit (human)
Rps10	NM_031109	0.83	-39.8	95.7	turq	10884620	ribosomal protein S10
Rps11	NM_031110	0.78	-58.1	82.8	turq	10721676	ribosomal protein S11
Rps12	NM_031709	0.82	-73.9	114.6	turq	10807514	ribosomal protein S12
Rps13	NM_130432	0.83	-22.5	76.5	turq	10725105	ribosomal protein S13
**Rps14**	**NM_022672**	**1.40**	**183**	**130.5**	**turq**	**10802007**	**ribosomal protein S14**
**Rps15**	**NM_017151**	**1.32**	**59.6**	**133.7**	**turq**	**10900533**	**ribosomal protein S15**
**Rps15a**	**NM_053982**	**1.32**	**122**	**143.4**	**turq**	**10752146**	**ribosomal protein S15a**
Rps17	NM_017152	1.31	91.0	9.6	blue	10844946	ribosomal protein S17
**Rps18**	**NM_213557**	**0.83**	**-31.2**	**116.9**	**turq**	**10870043**	**ribosomal protein S18**
**Rps19**	**NM_001037346**	**1.36**	**39.8**	**143.4**	**turq**	**10799987**	**ribosomal protein S19**
**Rps20**	**NM_001007603**	**1.33**	**262**	**152.6**	**turq**	**10873786**	**ribosomal protein S20**
**Rps21**	**NM_031111**	**1.34**	**194**	**118.8**	**turq**	**10909758**	**ribosomal protein S21**
Rps24	NM_031112	1.46	33	112.1	turq	10786132	ribosomal protein S24
**Rps25**	**NM_001005528**	**1.39**	**115**	**134.1**	**turq**	**10909521**	**ribosomal protein s25**
**Rps26**	**NM_013224**	**0.83**	**-169**	**22.0**	**blue**	**10899868**	**ribosomal protein S26**
**Rps27a**	**NM_031113**	**1.42**	**218**	**121.7**	**turq**	**10879392**	**ribosomal protein S27a**
Rps28	NM_001105730	0.74	-81	100.3	turq	10774372	ribosomal protein S28
Rps29	NM_012876	0.79	-785	96.1	turq	10896225	ribosomal protein S29
**Rps3a**	**NM_017153**	**0.81**	**-389**	**138.0**	**turq**	**10824082**	**ribosomal protein S3a**
Rps5	NM_001105722	0.82	-91	103.0	turq	10704242	ribosomal protein S5
**Rps7**	**NM_031570**	**1.20**	**235**	**143.1**	**turq**	**10881922**	**ribosomal protein S7**
Rps9	NM_031108	0.83	-90.3	79.9	turq	10718510	ribosomal protein S9
**Sf3b5**	**NM_001126092**	**1.31**	**18.8**	**19.0**	**blue**	**10701797**	**splicing factor 3b, subunit 5**
Sumo1	NM_001009672	0.77	-25.5	66.7	turq	10928423	SMT3 suppressor of mif two 3 homolog 1 (S. cerevisiae)
Taf9	NM_184048	0.72	-102	89.1	turq	10832879	TAF9 RNA polymerase II, TATA box binding protein (TBP)-associated factor
**Miscellaneous & Unknown**							
Bxdc1	NM_001106391	1.37	39.6	45.3	turq	10833643	brix domain containing 1
Chic1	ENSRNOT00000015693	0.82	-36.2	111.9	turq	10934502	cysteine-rich hydrophobic domain 1
Fam111a	NM_001109163	1.26	4.3	14.5	blue	10714106	family with sequence similarity 111, member A
Ift74	NM_001007001	1.43	37.8	92.0	turq	10869925	intraflagellar transport 74 homolog (Chlamydomonas)
LOC100125384	NM_001103363	1.29	3.8	8.7	blue	10707297	hypothetical protein LOC100125384
**LOC302473**	**NM_001106947**	**0.79**	**-54.2**	**20.1**	**blue**	**10940016**	**similar to SLIT and NTRK-like family, member 4**
LOC304558	XM_222260	1.21	5.6	7.9	blue	10762932	similar to TPR repeat-containing protein KIAA1043
LOC310926	NM_001025002	1.22	612	78.5	turq	10930624	hypothetical protein LOC310926
LOC362419	NM_001108642	0.80	-11.5	9.7	blue	10864722	similar to CG33331-PA
LOC682679	ENSRNOT00000054682	1.28	20.4	11.0	blue	10730895	hypothetical protein LOC682679
**LOC685233**	**XM_001062937**	**1.31**	**38.0**	**143.1**	**turq**	**10747891**	**hypothetical protein LOC685233**
**LOC690333**	**NM_001109580**	**1.27**	**17.7**	**1.0**	**ylw**	**10823196**	**hypothetical protein LOC690333**
**MGC95208**	**NM_001005552**	**0.80**	**-29.7**	**117.2**	**turq**	**10716936**	**similar to 4930453N24Rik protein**
Nhlrc3	ENSRNOT00000014562	1.22	10.1	15.4	blue	10823119	NHL repeat containing 3
RGD1307071	ENSRNOT00000036527	0.82	-7.3	16.5	blue	10840504	similar to uncharacterized hypothalamus protein HT013
RGD1308616	ENSRNOT00000039235	1.31	7.7	90.4	turq	10879240	similar to KIAA0467 protein
RGD1559961	NM_001163736	0.80	-36.2	78.5	turq	10745785	similar to novel protein
RGD1561672	ENSRNOT00000056708	1.21	5.4	4.0	blue	10778861	similar to novel protein
RGD1563065	ENSRNOT00000002978	0.78	-21.2	15.2	blue	10772788	similar to 3110047P20Rik protein

RGD1564140	BC166863	0.72	-77.3	90.2	turq	10800592	similar to AW554918 protein
Svep1	ENSRNOT00000047200	1.21	3.3	25.7	turq	10877020	sushi, von Willebrand factor type A, EGF and pentraxin domain containing 1
**Tanc1**	**NM_001002854**	**1.21**	**6.7**	**20.6**	**blue**	**10836354**	**tetratricopeptide repeat, ankyrin repeat and coiled-coil containing 1**
Tmem100	NM_001017479	0.80	-42.0	5.9	blue	10737426	transmembrane protein 100
Tmem184b	NM_001173370	0.80	-21.8	108.4	turq	10905427	transmembrane protein 184B
Tmem185b	ENSRNOT00000031412	1.22	38.5	62.4	turq	10763547	transmembrane protein 185B
Tmem192	NM_001014141	0.78	-62.4	103.0	turq	10791318	transmembrane protein 192
**Tmem25**	**NM_001109528**	**1.26**	**18.6**	**18.1**	**blue**	**10916867**	**transmembrane protein 25**
Tmem69	NM_001035001	1.20	31.6	45.9	turq	10878875	transmembrane protein 69
Tmem88b	NM_001109426	1.28	18.5	6.7	blue	10882221	transmembrane protein 88B
Ttc9	NM_001134731	1.24	21.2	37.9	turq	10885693	tetratricopeptide repeat domain 9
**ESTs**							
	ENSRNOT00000043419	1.22	2.1	1.0	ylw	10764047	
	ENSRNOT00000063746	0.77	-5.8	0.6	ylw	10800599	
	---	1.51	10.0	0.6	ylw	10823419	
	**ENSRNOT00000063523**	**1.28**	**891**	**142.7**	**turq**	**10761253**	
	**ENSRNOT00000031724**	**1.31**	**17.4**	**142.6**	**turq**	**10872098**	
	**J01884**	**2.07**	**35.0**	**130.5**	**turq**	**10830454**	
	**NC_001665**	**0.63**	**-275**	**130.2**	**turq**	**10930569**	
	**ENSRNOT00000056706**	**1.34**	**13.5**	**128.2**	**turq**	**10778876**	
	**ENSRNOT00000053479**	**0.72**	**-8.4**	**123.1**	**turq**	**10851380**	
	ENSRNOT00000054132	1.30	60.4	116.5	turq	10822420	
	ENSRNOT00000053364	0.72	-56.8	115.3	turq	10721694	
	ENSRNOT00000053230	0.71	-34.3	114.4	turq	10758033	
	ENSRNOT00000054449	1.50	16.0	110.8	turq	10751877	
	ENSRNOT00000042326	1.21	5.6	110.3	turq	10849305	
	ENSRNOT00000030232	0.81	-24.4	108.2	turq	10928976	
	BC091375	1.24	24.3	106.9	turq	10760068	
	ENSRNOT00000052442	0.72	-79.3	101.6	turq	10709825	
	K00780	1.54	38.2	101.3	turq	10737194	
	ENSRNOT00000006275	0.80	-5.3	98.3	turq	10903704	
RGD1311745	BC085913	1.20	21.3	95.7	turq	10921086	similar to RIKEN cDNA 1110059G10
	ENSRNOT00000024516	1.46	34.2	95.2	turq	10729852	
	ENSRNOT00000052472	0.83	-28.1	93.7	turq	10728563	
	ENSRNOT00000053276	0.76	-93	89.1	turq	10909358	
	ENSRNOT00000049219	0.79	-3.5	88.5	turq	10769894	
	NM_001105954	1.52	78.5	87.5	turq	10764376	
	ENSRNOT00000055081	1.21	4.0	85.6	turq	10852153	
	GENSCAN00000013683	1.41	23.0	84.0	turq	10917205	
	ENSRNOT00000052947	1.36	19.9	81.3	turq	10826602	
	---	0.83	-26.1	80.2	turq	10790615	
	ENSRNOT00000068510	1.37	70.5	79.3	turq	10866486	
	ENSRNOT00000017930	1.42	130	79.2	turq	10791507	
	ENSRNOT00000052447	0.51	-12.7	78.9	turq	10844062	
	---	1.30	12.8	78.1	turq	10727640	
RGD1305235	BC078920	0.81	-16.6	75.7	turq	10702670	similar to RIKEN cDNA 1700052N19
	---	1.69	46.0	73.4	turq	10911268	
	---	1.54	513	73.4	turq	10811898	
	ENSRNOT00000067296	0.83	-27.0	73.0	turq	10805514	
	ENSRNOT00000057159	1.44	60.2	72.9	turq	10766878	
	ENSRNOT00000053847	0.76	-61.4	71.8	turq	10767075	
	ENSRNOT00000049610	0.71	-6.1	71.1	turq	10873723	
	---	1.24	26.7	67.4	turq	10764312	
	ENSRNOT00000053811	1.30	118	62.4	turq	10914312	
	ENSRNOT00000054763	1.29	128	61.6	turq	10729629	
	ENSRNOT00000048070	0.83	-6.3	60.9	turq	10821769	
	ENSRNOT00000054170	1.69	25.7	58.8	turq	10817543	
	ENSRNOT00000053529	0.69	-425	55.8	turq	10722449	
	ENSRNOT00000060617	1.26	28.9	55.7	turq	10844248	
	ENSRNOT00000009569	1.37	75.0	52.5	turq	10856024	
	ENSRNOT00000041344	0.74	-18.0	51.0	turq	10785800	
	ENSRNOT00000063761	1.21	2.2	50.8	turq	10915781	
	J05014	0.82	-179	50.4	turq	10876313	
	ENSRNOT00000063341	1.31	8.5	47.6	turq	10836267	
	ENSRNOT00000002985	0.72	-12.3	47.4	turq	10776964	
	ENSRNOT00000056606	1.22	14.9	47.1	turq	10824548	
	ENSRNOT00000059478	1.25	26.5	47.0	turq	10927668	
	---	0.81	-8.7	45.7	turq	10715344	
	ENSRNOT00000053954	0.79	-3.3	45.2	turq	10803679	

	ENSRNOT00000045485	0.82	-24.0	45.1	turq	10796908	
	ENSRNOT00000032933	0.82	-24.8	45.1	turq	10705422	
	ENSRNOT00000053250	1.25	7.0	44.4	turq	10766287	
	rno-mir-30b	1.22	2.6	44.1	turq	10904219	
	ENSRNOT00000053296	1.51	8.9	42.9	turq	10805849	
RGD1309501	NM_001127537	1.21	11.4	42.0	turq	10774403	hypothetical LOC305552
	ENSRNOT00000052417	1.20	2.1	41.2	turq	10856141	
	ENSRNOT00000053957	0.62	-29.0	40.1	turq	10829357	
	ENSRNOT00000052576	1.60	36.1	39.3	turq	10878967	
	---	0.74	-10.2	38.1	turq	10774265	
	ENSRNOT00000055266	1.25	14.0	37.6	turq	10819852	
	ENSRNOT00000036265	0.80	-4.2	37.4	turq	10845363	
	ENSRNOT00000045359	1.22	4.3	37.2	turq	10767010	
	ENSRNOT00000052435	0.77	-37.9	36.7	turq	10908098	
	ENSRNOT00000052790	0.78	-7.1	36.6	turq	10831150	
	ENSRNOT00000054649	1.57	152	36.2	turq	10930226	
	ENSRNOT00000014009	0.82	-13.7	34.1	turq	10822175	
	ENSRNOT00000052640	0.73	-9.8	33.6	turq	10735369	
	ENSRNOT00000052698	1.36	5.6	33.6	turq	10731138	
	ENSRNOT00000054306	0.80	-4.5	31.6	turq	10707376	
	---	0.81	-3.5	30.2	turq	10865336	
	ENSRNOT00000017777	0.82	-14.2	28.5	turq	10792049	
	---	0.79	-6.2	28.5	turq	10742334	
	ENSRNOT00000005711	0.82	-4.7	27.7	turq	10903800	
	ENSRNOT00000047993	1.22	2.6	27.2	turq	10791331	
	ENSRNOT00000039565	0.83	-4.4	26.1	turq	10711498	
	ENSRNOT00000013475	0.72	-10.2	25.2	turq	10785895	
	ENSRNOT00000034545	0.82	-27.9	24.6	turq	10894115	
	---	1.24	3.5	23.9	turq	10864272	
	ENSRNOT00000001251	1.32	5.5	23.1	turq	10758048	
	ENSRNOT00000053294	1.24	3.5	22.1	turq	10733743	
RGD1562094	ENSRNOT00000019278	0.83	-32.6	21.6	turq	10706272	similar to RIKEN cDNA 2810426N06
	ENSRNOT00000038644	0.82	-3.0	19.8	turq	10938553	
	ENSRNOT00000053408	0.81	-22.2	17.1	turq	10918383	
	ENSRNOT00000053268	1.38	95.3	12.1	turq	10909356	
	ENSRNOT00000000361	1.22	10.3	8.2	turq	10743263	
RGD1562342	NM_001109281	0.77	-11.3	1.2	brwn	10899938	similar to RIKEN cDNA 1110012D08
	**---**	**0.79**	**-5.8**	**22.6**	**blue**	**10909375**	
	**ENSRNOT00000052502**	**0.82**	**-6.4**	**21.8**	**blue**	**10726170**	
	**---**	**1.20**	**5.7**	**21.7**	**blue**	**10875100**	
	**ENSRNOT00000008101**	**0.79**	**-41.3**	**21.5**	**blue**	**10840657**	
	**---**	**0.77**	**-5.8**	**19.2**	**blue**	**10931079**	
**RGD1306839**	**NM_001106347**	**0.70**	**-10.9**	**18.5**	**blue**	**10729604**	**similar to RIKEN cDNA 5033414D02**
	**---**	**1.20**	**6.3**	**18.1**	**blue**	**10804413**	
	GENSCAN00000015264	1.29	15.1	17.4	blue	10901115	
LOC363737	ENSRNOT00000032643	0.80	-57.1	17.2	blue	10778207	hypothetical LOC363737
	ENSRNOT00000053929	1.65	82.5	16.9	blue	10839872	
	ENSRNOT00000052957	1.24	18.1	16.5	blue	10717949	
	ENSRNOT00000016662	1.31	5.7	16.5	blue	10718474	
	ENSRNOT00000017331	0.81	-5.0	16.4	blue	10703137	
RGD1563666	NM_001134568	0.79	-7.7	15.5	blue	10939118	similar to chromosome X open reading frame 43
	GENSCAN00000038580	0.80	-3.6	15.3	blue	10864898	
	ENSRNOT00000036884	1.32	3.6	14.7	blue	10795677	
	ENSRNOT00000043813	0.82	-33.3	13.7	blue	10881482	
	ENSRNOT00000052854	0.80	-10.7	13.7	blue	10932269	
	ENSRNOT00000053505	1.31	6.3	13.3	blue	10872679	
	---	1.22	44.5	13.0	blue	10761285	
RGD1560652	ENSRNOT00000007422	0.65	-11.8	12.8	blue	10866501	RGD1560652
LOC290577	XM_002725158	0.75	-9.1	12.7	blue	10786993	hypothetical LOC290577
	rno-mir-124-2	1.24	2.6	12.0	blue	10814392	
	U22321	0.83	-25.5	11.7	blue	10931387	
	GENSCAN00000030991	0.83	-6.4	11.5	blue	10718830	
	BC168232	0.70	-38.4	11.3	blue	10881474	
	ENSRNOT00000018648	0.82	-1.8	10.9	blue	10825734	
	ENSRNOT00000053028	0.39	-34.7	10.8	blue	10802970	
	ENSRNOT00000043131	1.34	45.9	10.6	blue	10774908	
	ENSRNOT00000054027	0.81	-16.7	10.5	blue	10765034	
	ENSRNOT00000047350	0.82	-4.0	10.1	blue	10793387	
	ENSRNOT00000036737	0.83	-10.9	9.6	blue	10915988	

	ENSRNOT00000061413	1.47	4.5	9.6	blue	10875500	
	GENSCAN00000031414	0.77	-20.3	9.6	blue	10869614	
	ENSRNOT00000033266	0.81	-5.4	9.3	blue	10823421	
	ENSRNOT00000004583	0.78	-2.9	9.1	blue	10740812	
	ENSRNOT00000041141	0.83	-7.9	8.5	blue	10867488	
	ENSRNOT00000053878	0.70	-18.1	8.5	blue	10778349	
	ENSRNOT00000016287	0.83	-3.8	8.5	blue	10850866	
	---	0.81	-4.6	8.4	blue	10833673	
	ENSRNOT00000053902	0.83	-117	8.3	blue	10827450	
	ENSRNOT00000002159	1.27	20.0	8.2	blue	10911944	
	ENSRNOT00000048377	0.77	-8.5	8.2	blue	10790302	
	ENSRNOT00000003022	1.21	20.4	8.2	blue	10825165	
	GENSCAN00000044942	1.22	7.2	7.9	blue	10821147	
	ENSRNOT00000035662	1.22	4.5	7.5	blue	10933574	
	ENSRNOT00000029013	0.80	-23.2	7.2	blue	10805044	
	ENSRNOT00000048926	0.79	-6.7	7.1	blue	10889421	
	rno-let-7f-1	0.80	-17.5	7.1	blue	10794444	
	ENSRNOT00000058454	0.83	-3.7	7.0	blue	10938173	
	---	0.73	-3.9	6.8	blue	10765728	
	ENSRNOT00000054563	1.24	175	6.4	blue	10866957	
	ENSRNOT00000041929	0.81	-7.8	6.3	blue	10795295	
	GENSCAN00000017522	1.34	3.6	6.3	blue	10779348	
	ENSRNOT00000039731	1.27	4.3	6.2	blue	10785174	
	---	1.24	6.0	5.7	blue	10909070	
	ENSRNOT00000011063	0.77	-3.5	5.7	blue	10899981	
	ENSRNOT00000030972	1.39	13.9	5.6	blue	10832081	
	---	1.63	16.8	5.6	blue	10916853	
	ENSRNOT00000005636	1.21	6.9	5.5	blue	10904230	
	ENSRNOT00000009220	0.83	-4.2	5.2	blue	10898947	
	ENSRNOT00000041507	0.82	-5.7	5.1	blue	10718421	
	ENSRNOT00000031538	0.79	-21.5	5.1	blue	10741664	
	ENSRNOT00000033598	0.78	-6.6	5.1	blue	10729282	
	GENSCAN00000019030	1.26	2.7	5.1	blue	10723654	
	ENSRNOT00000046191	0.81	-5.8	4.8	blue	10795260	
	ENSRNOT00000052628	0.69	-44.1	4.3	blue	10834602	
	ENSRNOT00000053523	1.23	1.9	4.0	blue	10886190	
	ENSRNOT00000052253	0.73	-8.8	4.0	blue	10802934	
	ENSRNOT00000053019	0.77	-143	3.6	blue	10722451	
	ENSRNOT00000054162	1.50	11.3	2.7	blue	10800832	
	ENSRNOT00000052686	0.83	-6.1	2.5	blue	10835652	
	ENSRNOT00000051665	1.31	11.2	2.4	blue	10720130	
	ENSRNOT00000005413	0.72	-9.3	2.3	blue	10895439	
	ENSRNOT00000054622	1.30	5.7	2.2	blue	10898938	
	ENSRNOT00000053261	0.83	-38.0	2.0	blue	10707641	
	ENSRNOT00000039652	0.83	-9.5	1.9	blue	10820145	
	GENSCAN00000015919	1.30	11.0	1.8	blue	10864713	
	---	0.78	-18.3	1.8	blue	10776216	
	ENSRNOT00000028990	1.40	4.3	1.8	blue	10814363	
	---	1.32	10.0	1.7	blue	10838153	
	---	0.76	-7.7	1.6	blue	10834604	
	---	0.76	-3.7	1.4	blue	10843137	
	ENSRNOT00000056846	1.43	41.2	1.2	blue	10939615	
	ENSRNOT00000040892	1.32	4.6	0.0	grey	10789042	
	ENSRNOT00000052577	0.75	-20.4	0.0	grey	10878961	
	ENSRNOT00000057735	0.82	-43.4	0.0	grey	10770328	

*** - genes belonning to top 10% of each module withing each brain region are marked by bold font**

**- Modules and k.in are given for seaparate networks; abbreviations used for modules: *turq* - turquoise, *ylw* -yellow, *brwn* - brown

**Table S2-L. Differentially Expressed Genes in Female Rat Preoptic Area-Anterior Hypothalamus (51 genes and ESTs)**							
**Gene Symbol**	**GenBank, Ref.Sequence**	**Vin/Con Ratio**	**Vin-Con mean_dif**	**k.in ****	**Module****	**ProbeSet ID**	**Gene Title**
**Apoptosis**							
Higd2a	NM_001106102	1.25	49.7	36.7	turq	10797432	HIG1 hypoxia inducible domain family, member 2A
Parm1	NM_173114	0.71	-36.5	27.3	turq	10775862	prostate androgen-regulated mucin-like protein 1
**Cytoskeleton-ECM**							
Pcdhb5	NM_001114602	1.21	6.9	25.7	turq	10801174	protocadherin beta 5
**Development**							

Cpne9	NM_001024982	0.82	-6.7	31.6	turq	10857718	copine family member IX
Inhba	NM_017128	0.77	-7.9	33.6	turq	10798702	inhibin beta-A
Sncg	NM_031688	0.66	-59.0	37.2	turq	10790471	synuclein, gamma (breast cancer-specific protein 1)
**Growth Factors**							
RGD1307225	NM_001107663	0.80	-7.5	37.1	turq	10814545	similar to MEGF6
**Immune Response**							
Cd74	NM_013069	0.75	-22.1	31.0	turq	10802013	Cd74 molecule, major histocompatibility complex, class II invariant chain
RT1-Da	NM_001008847	0.67	-15.4	27.2	turq	10828344	RT1 class II, locus Da
RT1-N2	NM_001008854	1.57	16.3	36.0	turq	10827782	RT1 class Ib, locus N2
Spag6	NM_001034960	0.76	-10.0	31.5	turq	10752563	sperm associated antigen 6
**Metabolism & Transport**							
Car12	NM_001080756	0.67	-65.1	31.0	turq	10911145	carbonic anyhydrase 12
Car3	NM_019292	1.37	12.8	35.3	turq	10822242	carbonic anhydrase 3
Kcnc2	NM_139216	1.21	36.5	29.3	turq	10895443	potassium voltage gated channel, Shaw-related subfamily, member 2
Kcnh5	NM_133610	2.40	142	36.8	turq	10890609	potassium voltage-gated channel, subfamily H (eag-related), member 5
**Kpna5**	**NM_001025113**	**1.20**	**22.3**	**38.1**	**turq**	**10830088**	**karyopherin alpha 5 (importin alpha 6)**
Pgd	ENSRNOT00000018401	1.23	4.6	35.0	turq	10881669	phosphogluconate dehydrogenase
Plcb4	NM_024353	1.61	34.9	35.3	turq	10840183	phospholipase C, beta 4
**Pld5**	**ENSRNOT00000005332**	**0.71**	**-19.5**	**38.4**	**turq**	**10770161**	**phospholipase D family, member 5**
Pter	NM_022224	0.81	-6.7	37.3	turq	10796440	phosphotriesterase related
**Receptors & Binding Proteins**							
Necab2	NM_133415	1.20	25.6	35.9	turq	10808312	N-terminal EF-hand calcium binding protein 2
Olr1292	NM_001000960	1.25	4.0	35.6	turq	10916334	olfactory receptor 1292
**Vom2r65**	**NM_001099654**	**1.25**	**3.1**	**38.3**	**turq**	**10770892**	**vomeronasal 2 receptor, 65**
**Signaling**							
Adcyap1	NM_016989	0.80	-17.2	30.3	turq	10930539	adenylate cyclase activating polypeptide 1 (pituitary)
Hspb1	NM_031970	1.24	6.1	28.7	turq	10761128	heat shock protein 1
Ramp3	NM_020100	1.39	11.1	26.9	turq	10774115	receptor (G protein-coupled) activity modifying protein 3
**Transcription**							
Ankrd42	NM_001135013	0.83	-13.0	32.1	turq	10723656	ankyrin repeat domain 42
Asb15	BC097405	0.77	-3.3	35.8	turq	10853995	ankyrin repeat and SOCS box-containing protein 15
Foxp2	XM_001056575	0.83	-3.6	35.9	turq	10853783	forkhead box P2
Klhl4	NM_001108244	0.80	-11.1	36.4	turq	10934754	kelch-like 4 (Drosophila)
Med31	NM_001135813	1.28	40.3	30.0	turq	10744682	mediator complex subunit 31
Npas4	NM_153626	0.75	-12.1	29.9	turq	10727717	neuronal PAS domain protein 4
Plagl1	NM_012760	0.66	-19.0	36.2	turq	10701802	pleiomorphic adenoma gene-like 1
**Zcchc17**	**NM_001109267**	**0.83**	**-23.4**	**37.5**	**turq**	**10872473**	**zinc finger, CCHC domain containing 17**
**Miscellaneous & Unknown**							
Fam111a	NM_001109163	1.26	4.3	36.3	turq	10714106	family with sequence similarity 111, member A
**Tmem196**	**NM_001044269**	**0.79**	**-19.3**	**39.0**	**turq**	**10887826**	**transmembrane protein 196**
**ESTs**							
RGD1305537	NM_001108822	0.82	-4.8	36.7	turq	10740359	similar to RIKEN cDNA 3110001I22
RGD1309501	NM_001127537	1.21	11.4	32.4	turq	10774403	hypothetical LOC305552
RGD1564814	ENSRNOT00000038133	1.22	3.1	36.6	turq	10775737	similar to CDNA sequence BC061212
	---	0.82	-23.3	31.4	turq	10871661	
	ENSRNOT00000054619	1.36	66.7	33.3	turq	10889221	
	ENSRNOT00000057534	1.21	4.2	34.9	turq	10743680	
	---	1.21	3.8	32.1	turq	10726674	
	**ENSRNOT00000063480**	**1.21**	**3.4**	**38.6**	**turq**	**10860865**	
	ENSRNOT00000054766	1.21	3.6	24.2	turq	10729550	
	ENSRNOT00000052939	1.30	5.5	34.3	turq	10816564	
	ENSRNOT00000054462	1.32	25.8	32.3	turq	10756285	
	ENSRNOT00000054306	0.80	-4.5	31.3	turq	10707376	
	ENSRNOT00000023217	1.26	8.9	36.6	turq	10929562	
	ENSRNOT00000035080	1.21	12.1	37.2	turq	10813244	
	ENSRNOT00000054530	1.20	137	31.9	turq	10805787	

*** - genes belonning to top 10% of each module withing each brain region are marked by bold font**

**- Modules and k.in are given for seaparate networks; abbreviations used for modules: *turq* - turquoise, *ylw* -yellow, *brwn* - brown
