# Supplementary material for: Gene bionetworks involved in the epigenetic transgenerational inheritance of altered mate preference: environmental epigenetics and evolutionary biology
Source: BMC Genomics. 2014 May 16;15(1):377. doi: 10.1186/1471-2164-15-377 (PMC4073506; doi:10.1186/1471-2164-15-377)
Supplement: Supplementary file 5 — Additional file 5: Top cellular pathways affected by signature gene lists and chosen modules from separate networks. (PDF 80 KB) [file 12864_2013_6162_MOESM5_ESM.pdf]

Supplemental Table S3.

Pathways Affected Male and Female Brain Region Signature Gene Lists and Chosen Modules from Separate Networks

(pathways with 5 and more affected genes per pathway are shown)

Pathway Class / Pathway Name	Female Signature Lists								Male Signature Lists								Male and Female 6 Regions	Modules of Separate Networks													
	Impact Factor ONTOGA*	# Input Genes in PW							Impact Factor ONTOGA*	# Input Genes in PW								# Input Genes in Pathway													
		6 Brain Regions	Amy	Hipp	CngCTX	EnCTX	OlfB	POAH		6 Brain Regions	Amy	Hipp	CngCTX	EnCTX	OlfB	POAH		Fem-Amy-Turq	Fem-CngCTX_Yellow	Fem-OlfB-Yellow	Male-Amy-Blue	Male-Amy-Turq	Male-CngCTX-Turq	Male-EnCTX-Turq	Male-Hipp-Blue	Male-Hipp-Turq	Male-OlfB-Blue	Male-OlfB-Brown	Male-OlfB-Green	Male-OlfB-Yellow	Male-POAH-Turq
**Total Number of Affected KEGG Pathways (at least with 1 gene)**		**175**	**15**	**22**	**105**	**125**	**120**	**40**		**195**	**51**	**60**	**163**	**116**	**150**	**11**	**202**	**5**	**26**	**6**	**12**	**51**	**163**	**112**	**3**	**63**	**51**	**10**	**4**	**2**	**12**
**Carbohydrate Metabolism**																															
Propanoate metabolism		6		1	2	2	1			7			5		2		11						3								
Inositol phosphate metabolism		4			2	2		1		8		3	4	1	1		10						3	1		3					
Glycolysis / Gluconeogenesis		3				1	1			5		1	1		3		7						1			1					
Pyruvate metabolism		3				1	1			4			2		2		6						2								
**Energy Metabolism**																															
Oxidative phosphorylation		16			14		10			2			1	1			18						1	1							
Nitrogen metabolism		4		1	2	2		2		5	1		3	1	2		8		1			1	3	1			1				
**Lipid Metabolism**																															
Glycerophospholipid metabolism		8			5	2	3			9	1		3	2	3		16		1			1	3	2				1			
Fatty acid metabolism		5				3	1			7			4	1	3		11						4	1							
Ether lipid metabolism		2			2	1				8	1	1	4		3		10					1	4			1		2			
Arachidonic acid metabolism		6	1		2	1	3			4	1	1	1		1		10					1	1			1					
Sphingolipid metabolism		2			1	1				4			3		1		6						3								
**Nucleotide Metabolism**																															
Purine metabolism		10			3	2	4			10			7	1	2		19						7	1							
Pyrimidine metabolism		4				2	1			6			4	2	1		9						4	2							
**Amino Acid Metabolism**																															
Valine, leucine and isoleucine degradation		5			3	1				9			6	1	3		13						5	1							
Tryptophan metabolism		4				1	2			8			6	1	3		11			1			6	1							
Arginine and proline metabolism		3				2				7			4	1	2		9						4	1							
Glycine, serine and threonine metabolism		3				3				8	1		5		3		8					1	5				1				
Lysine degradation		2				1				7	1		5	1	3		8					1	5	1							
Histidine metabolism		3				1	1			5			3		2		7			1			3								
Alanine, aspartate and glutamate metabolism		1				1				4			1		3		5						1								
**Metabolism of Other Amino Acids**																															
Glutathione metabolism		2					2			5		2	3	1	1		7						3	1		2					
beta-Alanine metabolism		3				2				4			2		2		5						2								
**Glycan Biosynthesis and Metabolism**																															
Glycerolipid metabolism		6			1	2	2			7	1		2	1	3		11		1			1	2	1							
Glycosylphosphatidylinositol (GPI)-anchor biosynthesis		6								1					1		6														
Glycosaminoglycan biosynthesis - heparan sulfate		3			1	2	1			3		1	1	1	1		5						1	1		1					
Other glycan degradation		1				1				4				1	3		5							1			1				
**Metabolism of Cofactors and Vitamins**																															
Retinol metabolism		5	2	1		1	1			3	1			1	1		7	1				1		1							
Porphyrin and chlorophyll metabolism		2			2					3	1	1		1			5		1			1		1		1					
**Xenobiotics Biodegradation and Metabolism**																															

Drug metabolism - cytochrome P450		2				1	1			5		1	4	1	1		7						4	1		1					
Metabolism of xenobiotics by cytochrome P450		2				1	1			4		2	2	1	1		6						2	1		2					
**Transcription**																															
Spliceosome		7			4	2	2			4	1		2	1			12	1	2			1	2	1							
**Translation**																															
Ribosome	1.0	52			26		46		0.9	3			1	1	1		54						1	1			1				
**Folding, Sorting and Degradation**																															
RNA transport		11	1		6	1	5			11			10	1			21	1					9	1							
Ubiquitin mediated proteolysis	0.9	4				3	1		1.5	9			8	2			13						8	2							
Protein processing in endoplasmic reticulum		4			3		2			9			6	2	2		12						6	2							
SNARE interactions in vesicular transport	5.2	6			3	2	2		2.7	4		1	2	1			8						2	1		1					
Proteasome	3.7	6			4		1		0.8	1			1				7						1								
RNA degradation		3			2	1				2			1	1			5		1				1	1		1					1
**Membrane Transport**																															
ABC transporters	3.2	6			3	4	2		1.2	2	1		1				8				1		1								
**Signal Transduction**																															
MAPK signaling pathway	2.7	17			7	10	3	1	8.6	31	3	2	15	7	7		45					3	15	7		2	1				
Calcium signaling pathway	7.5	21			8	9	6	1	6.7	20	2	3	7	3	7		35		2			2	7	3		3	1	1			
Wnt signaling pathway	2.5	10			2	5	2	1	2.5	12	1	1	5	2	6		21				1		5	2		1	1				
ErbB signaling pathway	2.2	6			4	2			2.9	10			6	2	4		16		1				6	2							
Phosphatidylinositol signaling system	8.5	8			2	5	1	1	28	9		3	3	2	2		14						3	2		3					
TGF-beta signaling pathway	2.8	5		2	2	1	1	1	5.8	9		2	6	1			14		1				6	1		2					
VEGF signaling pathway	3.4	6			1	3	1	1	2.0	6	1		4		1		12					1	4								
Jak-STAT signaling pathway	1.7	7			2	5	1		2.9	8			4	2	3		12		1				3	2							
Notch signaling pathway	1.8	2				1	1		3.5	6			3		3		8						3						1		
Toll-like receptor signaling pathway	0.8	2	1			1			2.3	6		1	3	1	1		8						2	1		1					
Hedgehog signaling pathway	1.2	2		1		1			3.4	5		2	1	1	1		7						1	1		2	1				
**Signaling Molecules and Interaction**																															
Neuroactive ligand-receptor interaction	4.5	24			8	12	8		4.9	26	5	3	12	4	4		45		2			5	12	4		3	1	1			
Cell adhesion molecules (CAMs)	157	14	1	2	6	4	2	2	14	18	2	1	12	2	2	1	30		3		1	1	8	1		1					1
Cytokine-cytokine receptor interaction	1.7	10		1	4	4	2		2.3	16		2	8	3	5		23		1				8	3		2					
ECM-receptor interaction	2.6	6			3		3		2.4	7		3	3		1		12		1				3			3					
**Transport and Catabolism**																															
Endocytosis		13		2	2	6	3	1		25	2	3	15	2	5		37				1	1	11	1		3					
Phagosome		11		2	1	1	5	2		21	2	3	14	1	2		30				1	1	9		1	2					
Lysosome		5		1	1	2	3			14	1		5	3	5		19					1	5	2			1				
Peroxisome		3			1	2				7			4	3			9						4	3							
**Cell Motility**																															
Regulation of actin cytoskeleton	2.7	14			3	8	4		3.6	20	1	3	10	2	5	1	31					1	10	2		3	2	1			1
**Cell Growth and Death**																															
Cell cycle	1.4	5			1	2	3		2.1	9	1		6	1	2		13					1	6	1							
Oocyte meiosis		5			2	1	2			8			6	1	2		13						6	1			1				
p53 signaling pathway	1.2	4			1	1	2		2.7	6	1		2	2	3		9				1		2	2							
Apoptosis	1.4	3		1		2			1.8	4			3	1	2		7						3	1			1				
**Cell Communication**																															
Focal adhesion	2.6	12			4	5	3		4.1	18		5	10	1	6		30		1				10	1		5	1				
Gap junction	4.2	8			2	4	3	1	7.2	11	1	2	6		3		18					1	6			2	1				
Tight junction	5.0	5			1	5			2.5	10			7		2	1	15						7				2				1
Adherens junction	5.8	3			1	1			6.1	5		1	2	1	1		7						2	1		1	1				
**Immune System**																															
Chemokine signaling pathway		14			5	6	4	1		19	1	2	11		6	1	31					1	10			2	1				1
Antigen processing and presentation	27	8		2	1		2	3	10	13	2		9	1	2		21				1	1	5								

Leukocyte transendothelial migration	348	7			1	7			23	8		1	3		3	1	15						3		1		2				1
Fc gamma R-mediated phagocytosis		8			1	4	2			9		1	6	1	1		13						5	1		1		1			
T cell receptor signaling pathway	1.5	4			2	3			1.8	8			5	3			12						5	3							
Hematopoietic cell lineage	2.9	7			2	4	1	1	3.9	9		3	3	2	2		11		2				3	2		3					
Natural killer cell mediated cytotoxicity	3.2	4			1	2	2		1.8	7			7		3		10						7								
B cell receptor signaling pathway	1.3	3				2			4.1	8			7	1			10						6	1							
Complement and coagulation cascades	4.7	1	1						3.9	8		3	2	1	2	1	9						2	1		3	1			1	1
Fc epsilon RI signaling pathway	1.7	2				2			1.9	6	1		4		1		8					1	4								
**Endocrine System**																															
GnRH signaling pathway	4.4	8			3	4	1	1	2.7	9	1	1	4		3		16		1			1	4			1	1				
Melanogenesis	3.3	7			2	3	2	1	4.4	9		1	7	1	2		14						7	1		1	1				
Progesterone-mediated oocyte maturation		6			2	2	2			8	1		6	1	1		13					1	6	1			1				
PPAR signaling pathway	1.4	5			1	2	1	1	2.0	5	1		2		2		10					1	2								
Adipocytokine signaling pathway	1.6	5			1	3	1		2.9	3			1	1	2		8						1	1							
Insulin signaling pathway	1.0	1			1				1.1	6			3	1	3		7						3	1			1	1			
**Circulatory System**																															
Vascular smooth muscle contraction		9			2	6	2	2		14	1	2	6		5		20		1			1	6			2	2				
Cardiac muscle contraction	2.8	10			9		5		1.0	2			1	1			12						1	1							
**Digestive System**																															
Gastric acid secretion		11		1	5	5	1	1		11	1	1	4	1	4		20		1			1	4	1		1	2				
Salivary secretion		16			8	7	2	1		5		1	1		3		19		1				1			1	1				
Pancreatic secretion		12			5	5	2	1		8	1	1	3		3		18					1	3			1					
Protein digestion and absorption		8			7		2			3			2		2		10		1				2				1				
**Excretory System**																															
Vasopressin-regulated water reabsorption		3			2	1				3			2		1		6						2				1				
Proximal tubule bicarbonate reclamation		4			2	1	1			1			1				5						1								
**Nervous System**																															
Long-term depression	3.9	6			1	4	2	1	9.8	13	1	1	8	1	2		18					1	8	1		1					
Long-term potentiation	6.0	8			2	3	4	1	4.7	8		1	4	1	2		15						4	1		1	1				
Neurotrophin signaling pathway		2			1	1				9			7	2	2		11						7	2							
**Sensory System**																															
Olfactory transduction	1.4	12	2	2	2	2	6		1.2	31	1	1	16	13	3		40			1		1	14	12		1	1		1		
Taste transduction	2.4	3			2		1		2.1	2					2		5										1				
Development																															
**Axon guidance**	8.0	16	1		5	5	6		8.7	17	1		10	7	3		32	1	1			1	10	7							
**Cancers**																															
Pathways in cancer (overview)	2.2	18			7	7	4		4.3	28	1	2	16	7	6		43														
Renal cell carcinoma	2.7	6			3	2	2		3.0	7			6	1	1		13						6	1							
Small cell lung cancer	1.4	6			2	2	1		1.9	8		1	4	1	2		12						2	1		1					
Pancreatic cancer	3.0	6			1	3	3		2.3	5		1	3		1		11						2			1					
Chronic myeloid leukemia	1.3	3				2	2		2.7	8		1	6		2		11						6			1					
Colorectal cancer	1.6	2				2	1		3.5	7		1	3	2	2		9						3	2		1					
Glioma	1.5	3			1	1	1		1.7	5			4		2		8						4								
Melanoma	1.0	1					1		5.2	6			4	1	2		7						4	1							
Non-small cell lung cancer	2.5	2				1	1		2.5	5			3		2		7						2								
Bladder cancer	2.8	3			1	1	1		2.0	3			2		1		6						2								
Endometrial cancer									2.4	5			2	1	2		5						2	1			1				
Thyroid cancer	3.4	1					1		3.4	4			2		2		5						2								
Acute myeloid leukemia	1.9	1			1				2.4	5			3	1	1		5						3	1							
**Immune System Diseases**																															
Systemic lupus erythematosus	12	23	1		9	1	14	1	1.2	4		1	2		1		25						1			1					
Autoimmune thyroid disease	2.9	6	1	2			1	2	5.3	9	2		6	1	1		15				1	1	2								
Allograft rejection	3.1	6	1	2			1	2	6.0	9	2		6	1	1		15				1	1	2								
Graft-versus-host disease	3.8	6	1	2			1	2	6.4	9	2		6	1	1		15				1	1	2								
**Neurodegenerative Diseases**																															

Huntington's disease	3.1	19			12	3	11	1	1.0	9		2	4	3	2		27						4	3		2				1	
Alzheimer's disease	2.6	17			12	2	10	1	1.3	9	1	1	4	2	3		25					1	4	2		1			1		
Parkinson's disease	2.7	14			11		10		1.0	3			2	1	1		17						2	1							
Amyotrophic lateral sclerosis (ALS)	2.0	4				2	2		4.4	7			4	4	1		11						4	4							
**Cardiovascular Diseases**																															
Viral myocarditis		9	1	2	1	1	2	2		15	2	1	9	1	5		23				1	1	5			1					
Dilated cardiomyopathy		6			2	1	4			10		3	4	1	2		15						4	1		3	1				
Arrhythmogenic right ventricular cardiomyopathy (ARVC)		5		1	2		2			11		3	5	1	2		14						5	1		3	1				
Hypertrophic cardiomyopathy (HCM)		4			1	1	3			10		3	4	2	1	1	13														
**Metabolic Diseases**																															
Type I diabetes mellitus	3.4	7	1	2	1		1	2	5.2	9	2		6	1	1		16				1	1	2								
**Infectious Diseases**																															
Toxoplasmosis		4				3	1	1		14	1	1	10	2	3		18					1	9	2		1					
Chagas disease		3				3	1	1		15		2	9	4	3	1	17														
Hepatitis C		5			2	4	1			9		1	6	1	1	1	14						5	1		1					1
Amoebiasis		4				3	1	2		9		2	3	1	5		12						3	1		2	2				
Leishmaniasis		6			1	3	2	1		6			4	2	1		11						3	2							
Malaria		9				1	6			8	5	1	1		1		11					2	1			1					
Bacterial invasion of epithelial cells		3				2	1			6			5		5		9						5				2				
Staphylococcus aureus infection		3					1	1		5		1	3		1		6						2			1	1				

*- impact factor was calculated using online software Ontoga-tool which features only signaling pathways
