# Supplementary material for: Gene bionetworks involved in the epigenetic transgenerational inheritance of altered mate preference: environmental epigenetics and evolutionary biology
Source: BMC Genomics. 2014 May 16;15(1):377. doi: 10.1186/1471-2164-15-377 (PMC4073506; doi:10.1186/1471-2164-15-377)
Supplement: Supplementary file 6 — Additional file 6: Table S3: Pathways Affected Male and Female Brain Region Signature Gene Lists and Chosen Modules from Separate Networks. (PDF 54 KB) [file 12864_2013_6162_MOESM6_ESM.pdf]

Supplemental Table S4. Correlation between combined network modules and behavior trait for F3-Vinclozolin rat brain regions

Rat Group	Behavior Trait		Wire Mesh		Facial Investigation		Plexiglas		Still		Walking	
	**Module**	**# of PCs***	**Correlation**	**p-value**	**Correlation**	**p-value**	**Correlation**	**p-value**	**Correlation**	**p-value**	**Correlation**	**p-value**
**Female**	blue	1	0.05	1	0.05	1	0.10	1	-0.09	1	0.10	1
	brown	1	-0.01	1	0.04	1	0.03	1	-0.06	1	0.02	1
	turquoise	1	0.01	1	-0.02	1	-0.01	1	0.02	1	-0.01	1
	yellow	1	-0.01	1	0.00	1	0.03	1	-0.03	1	0.03	1
**Male**	black	1	0.13	1	-0.15	1	-0.03	1	-0.13	1	-0.01	1
	blue	1	0.05	1	-0.02	1	0.01	1	0.01	1	0.06	1
	brown	1	0.01	1	-0.03	1	-0.03	1	-0.05	1	-0.03	1
	green	1	0.002	1	0.02	1	0.02	1	0.01	1	0.00	1
	magenta	1	-0.05	1	0.05	1	-0.04	1	-0.02	1	-0.06	1
	pink	1	-0.26	1	0.12	1	-0.02	1	0.01	1	-0.12	1
	red	1	0.01	1	0.00	1	0.01	1	0.002	1	0.01	1
	turquoise	1	-0.04	1	0.00	1	-0.04	1	-0.02	1	-0.04	1
	yellow	1	-0.03	1	0.02	1	0.06	1	0.04	1	-0.02	1

*** - number of principal components (PC) used for calculation of correlation between module and behavior trait**
