# Supplementary material for: Gene bionetworks involved in the epigenetic transgenerational inheritance of altered mate preference: environmental epigenetics and evolutionary biology
Source: BMC Genomics. 2014 May 16;15(1):377. doi: 10.1186/1471-2164-15-377 (PMC4073506; doi:10.1186/1471-2164-15-377)

Supplemental Figure S2 (Color)

Brain Region Specific Signature List Direct Connection Gene Sub-Networks

A. F-Amy(139)-Direct Connection Gene Sub-Networks

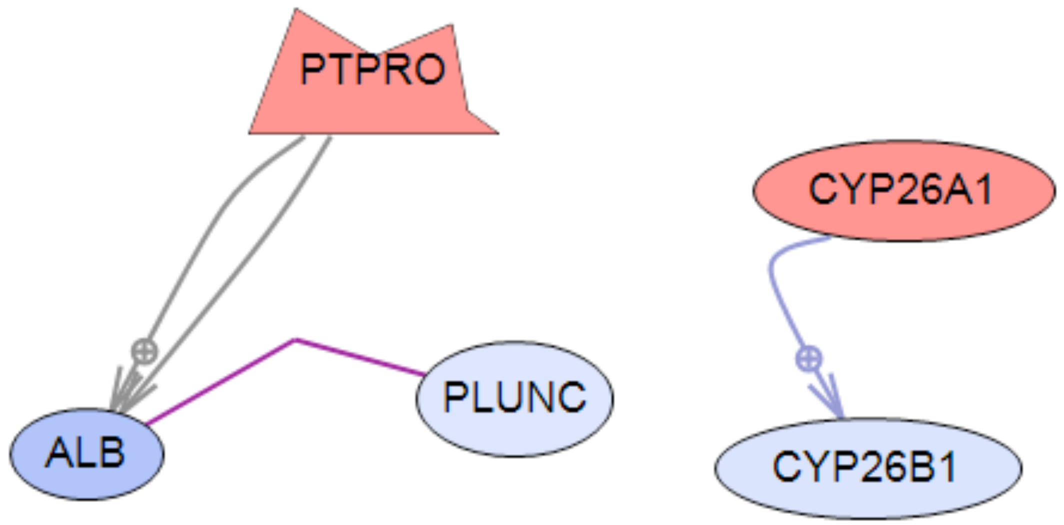

B. F-POAH(56)-Direct Connection Gene Sub-Networks

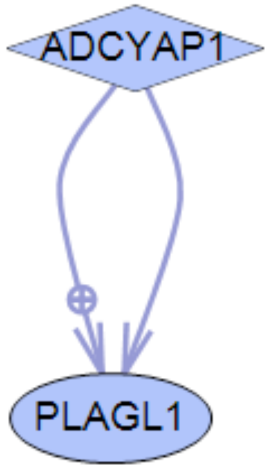

C. F-Hipp(70)-DirectConnection Gene Sub-Networks

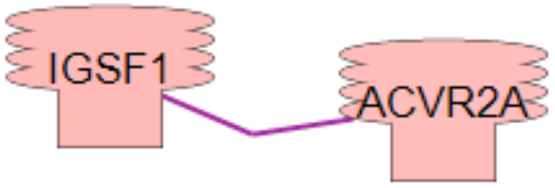

D.   F -EnCTX(433)-Direct Connection Gene Sub-Networks

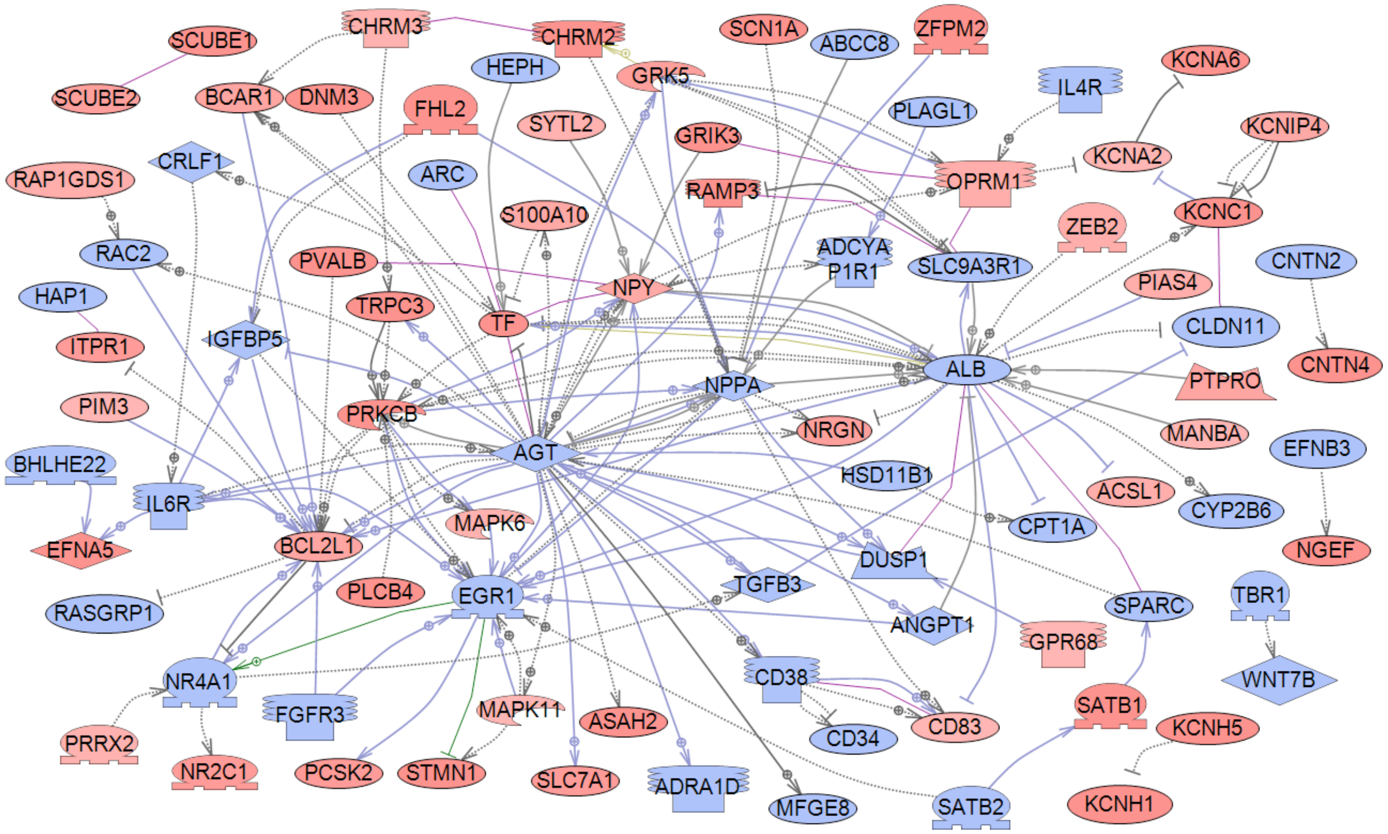

E.   F-CngCTX(803)-Direct Connection Gene Sub-Networks

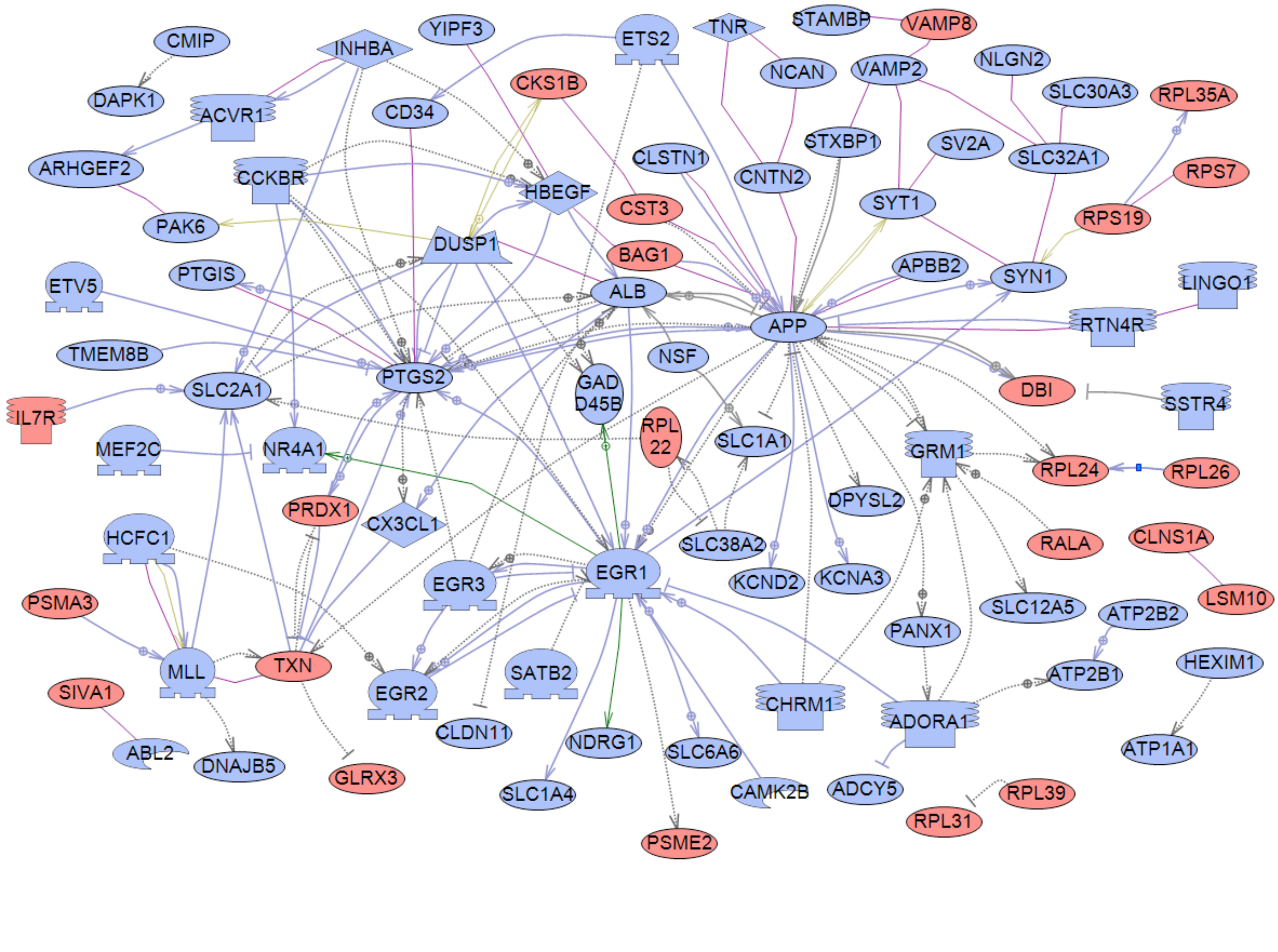

F. F –OlfB (748)-Direct Connection Gene Sub-Networks

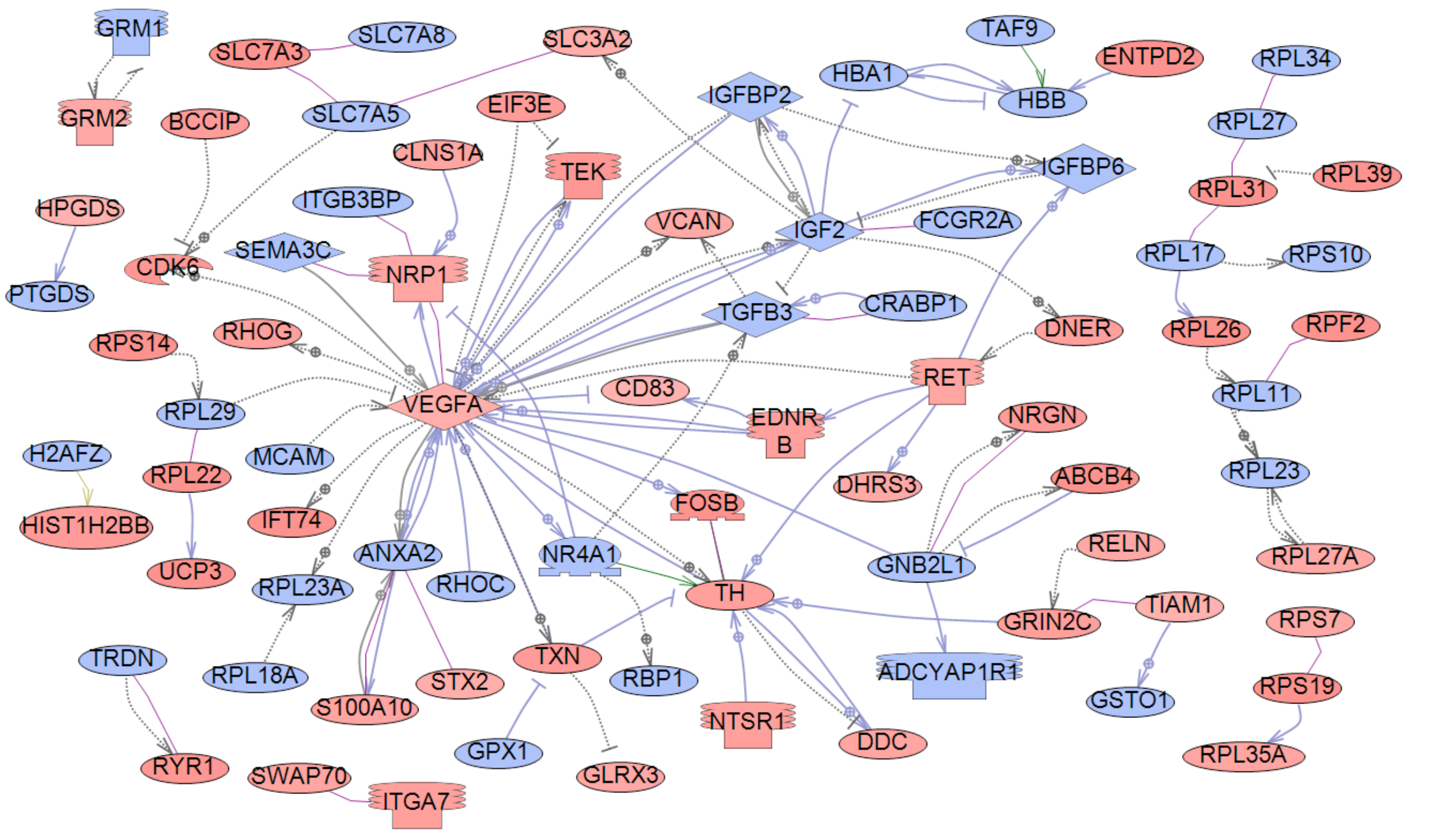

G. M-Amy(175)-Direct Connection Gene Sub-Networks

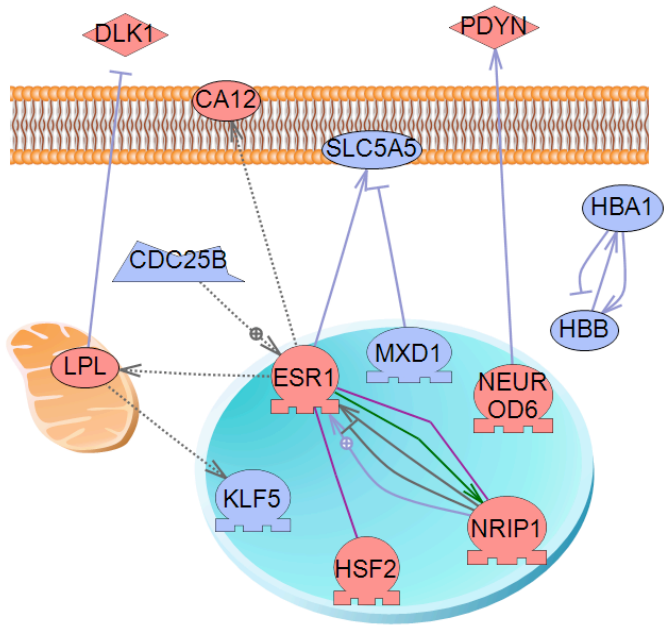

H. M-Hipp(151)-Direct Connection Gene Sub-Networks

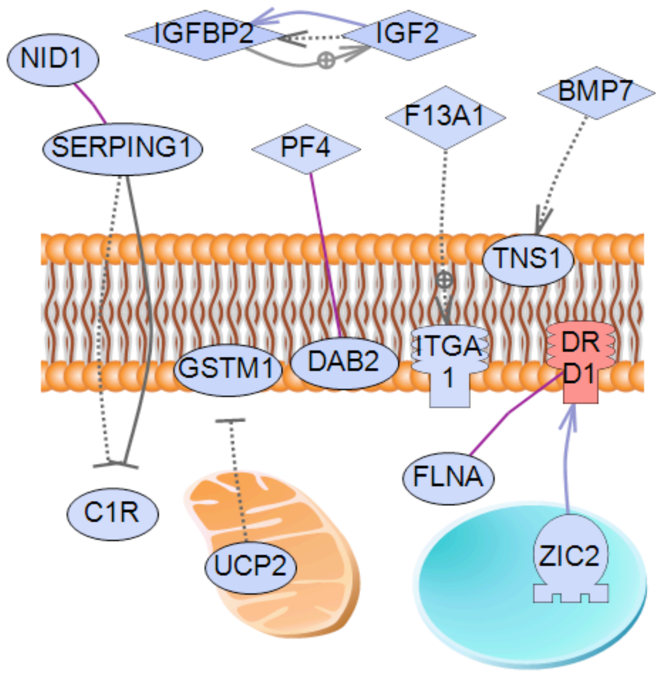

I. M-CngCTX (785)-Direct Connection Gene Sub-Networks

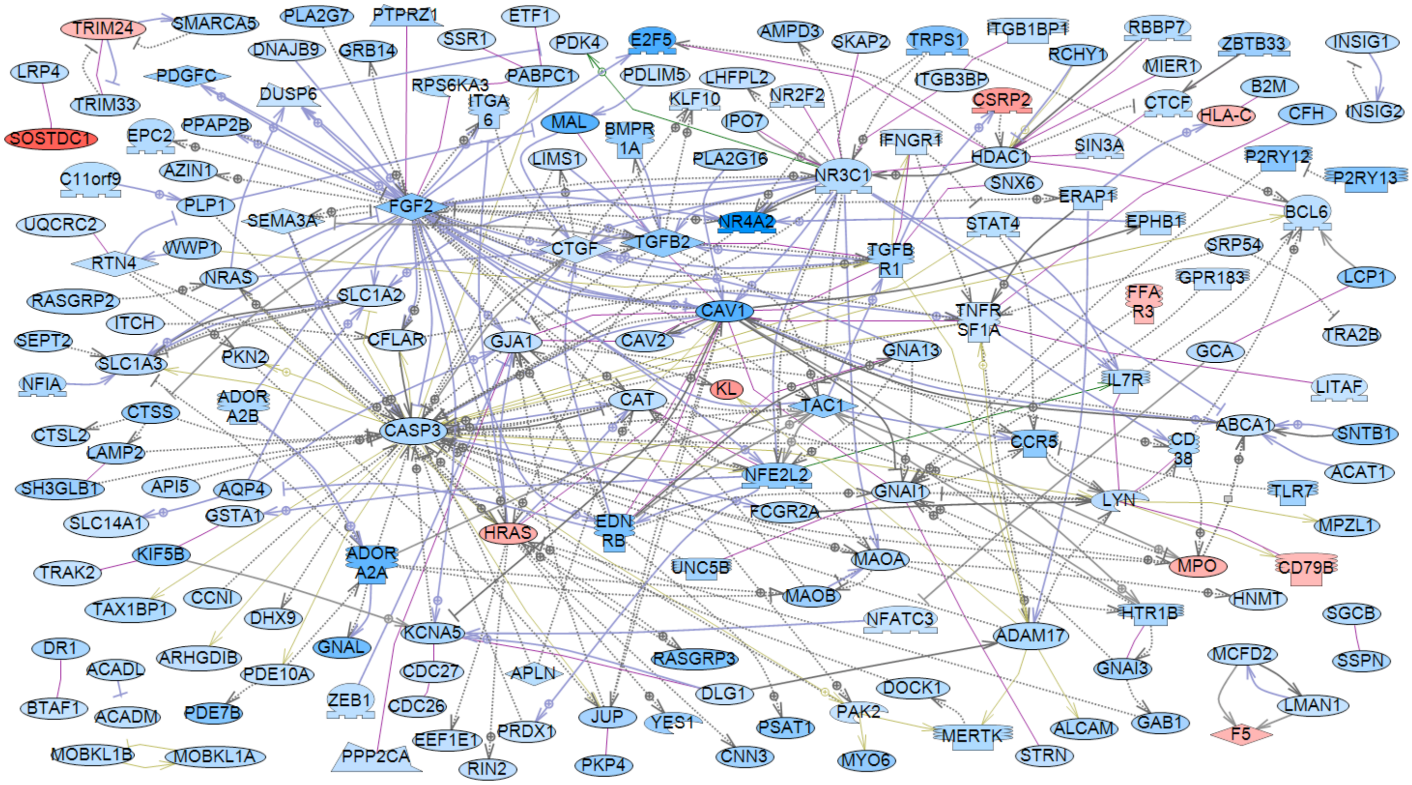

J. M-EnCTX (385)-Direct Connection Gene Sub-Networks

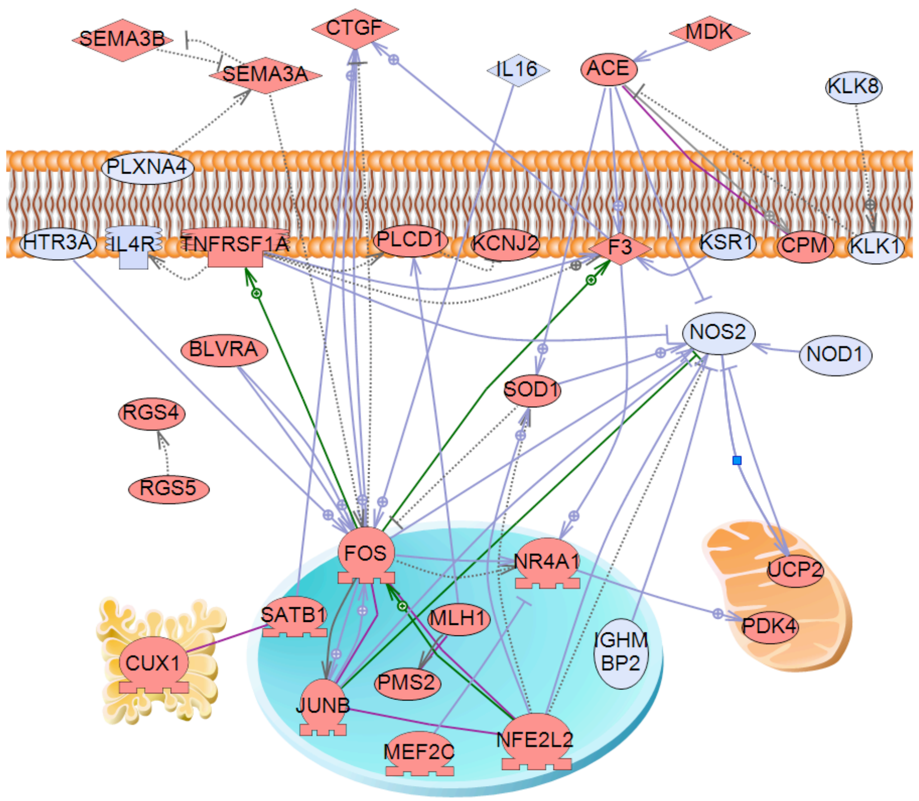

K. M –OlfB (356)-Direct Connection Gene Sub-Networks

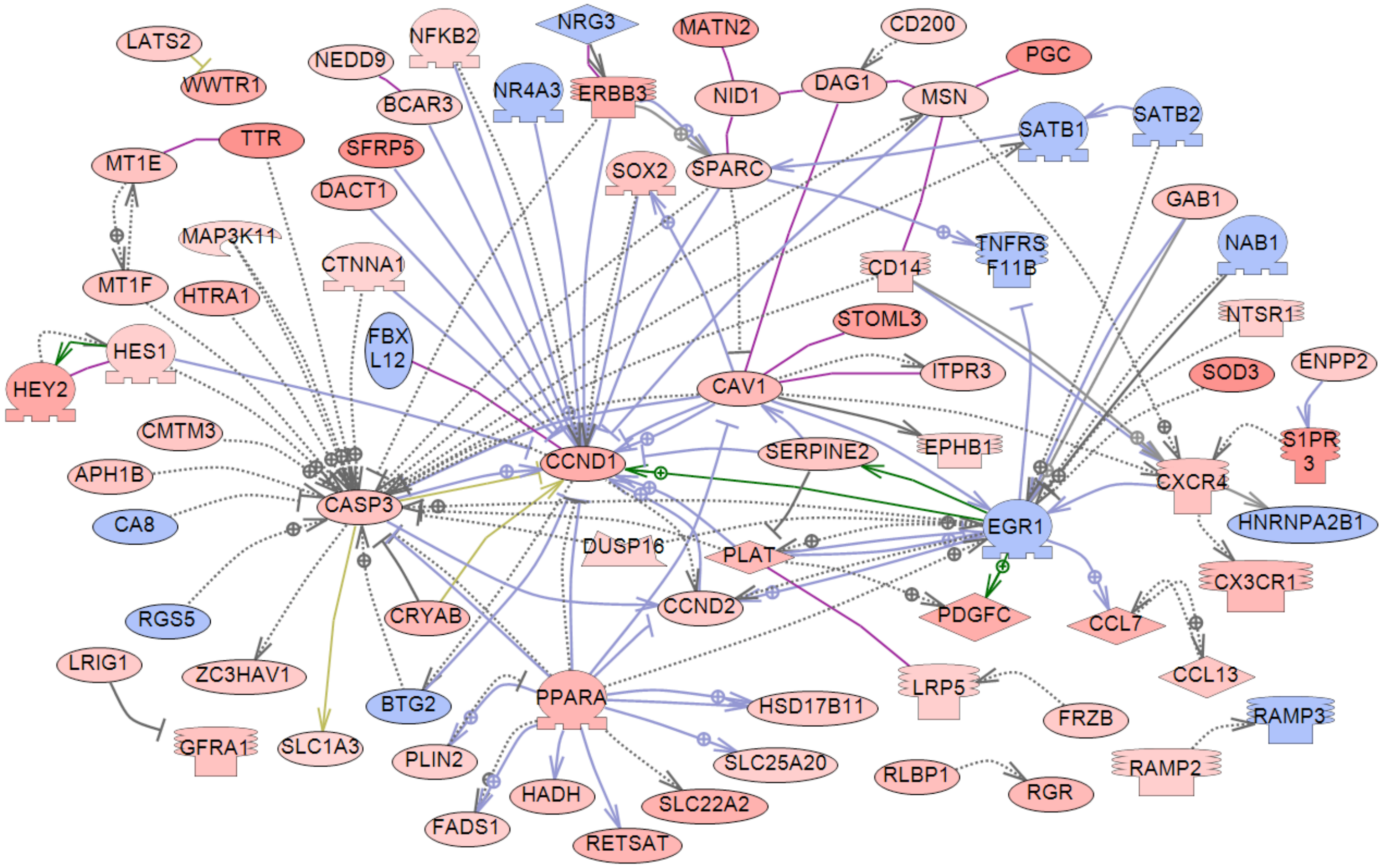

Supplement: Supplementary file 7 — Additional file 7: Table S4: Correlation between combined network modules and behavior trait for F3-Vinclozolin rat brain regions. (PDF 2 MB) [file 12864_2013_6162_MOESM7_ESM.pdf]
