# Supplementary material for: Gene bionetworks involved in the epigenetic transgenerational inheritance of altered mate preference: environmental epigenetics and evolutionary biology
Source: BMC Genomics. 2014 May 16;15(1):377. doi: 10.1186/1471-2164-15-377 (PMC4073506; doi:10.1186/1471-2164-15-377)
Supplement: Supplementary file 8 — Additional file 8: Figure S2: (Color) Brain Region Specific Signature List Direct Connection Gene Sub-Networks. Legend: Figure S2. Direct connection sub-networks for signature lists:female amygdala (A), female preoptic area-anterior hypothalamus(B), female hippocampus (C), female enterorhinal cortex (D), female cingulate cortex(E), female olfactory bulbs (F), male amygdala (G), male hippocampus (H), male cingulate cortex(I), male enterorhinal cortex (J), male olfactory bulbs (K) obtained by global literature analysis using Pathway Studio 8.0 software (Ariadne Genomics, Inc., Rockville, MD). Numbers in brackets on figures subtitles indicate number of genes in the list. Only directly connected genes are shown. Some sub-networks (G, H, J) show gene location in the cell (on membrane, in Golgi apparatus, nucleus, cytoplasm or outside the cell). Node shapes and color code: oval and circle – protein; diamond – ligand; circle/oval on tripod platform – transcription factor; ice cream cone – receptor; crescent – kinase or protein kinase; irregular polygon – phosphatase; red color indicates up-regulated genes, blue – down-regulated. Arrows with plus sign show positive regulation/activation, arrows with minus sign – negative regulation/inhibition; grey arrows represent regulation, lilac - expression, purple – binding, green – promoter binding, and yellow – protein modification. (PDF 66 KB) [file 12864_2013_6162_MOESM8_ESM.pdf]

Supplemental Table S5. Correlation between separate network modules and behavior trait for F3-Vinlozolin rat brain regions

Sex-Region	Behavior Trait		Wire Mesh		Facial Investigation		Plexiglas		Still		Walking	
	Module	# of PCs*	Correlation	p-value	Correlation	p-value	Correlation	p-value	Correlation	p-value	Correlation	p-value
F- Amy	blue	3	0.55	**0.438**	0.63	**0.285**	0.38	**0.763**	0.28	**0.892**	0.31	**0.860**
	brown	1	0.00	**0.989**	0.01	**0.972**	0.26	**0.443**	0.08	**0.823**	0.17	**0.622**
	turquoise	2	0.60	**0.172**	0.44	**0.426**	0.52	**0.028**	0.82	**0.012**	0.90	**0.001**
F-CngCTX	blue	1	0.30	**0.345**	0.29	**0.361**	0.40	**0.194**	0.10	**0.749**	0.09	**0.779**
	brown	2	0.55	**0.198**	0.36	**0.532**	0.41	**0.441**	0.22	**0.803**	0.17	**0.883**
	turquoise	1	0.37	**0.232**	0.29	**0.362**	0.38	**0.221**	0.03	**0.933**	0.03	**0.937**
	yellow	3	0.51	**0.468**	0.64	**0.213**	0.49	**0.0878**	0.36	**0.763**	0.41	**0.676**
F-EnCTX	blue	1	0.355	**0.258**	0.26	**0.424**	0.55	**0.066**	0.25	**0.437**	0.40	**0.203**
	brown	2	0.505	**0.266**	0.57	**0.176**	0.64	**0.092**	0.34	**0.581**	0.43	**0.402**
	turquoise	3	0.81	**0.029**	0.28	**0.100**	0.41	**0.037**	0.53	**0.424**	0.51	**0.458**
F-Hipp	turquoise	2	0.312	**0.736**	0.362	**0.655**	-0.53	**0.083**	0.593	**0.272**	0.588	**0.28**
F-OlfB	blue	3	0.61	**0.330**	0.49	**0.563**	0.48	**0.574**	0.23	**0.937**	0.24	**0.934**
	brown	1	0.17	**0.623**	0.01	**0.976**	0.34	**0.302**	0.16	**0.635**	0.16	**0.634**
	turquoise	1	0.19	**0.580**	0.21	**0.542**	0.20	**0.552**	0.02	**0.953**	0.002	**0.996**
	yellow	2	0.50	**0.311**	0.65	**0.107**	0.74	**0.044**	0.37	**0.558**	0.41	**0.488**
F-POAH	turquoise	1	0.55	**0.123**	0.264	**0.325**	0.627	**0.039**	-0.255	**0.354**	0.291	**0.259**
M- Amy	blue	2	0.54	**0.215**	0.47	**0.318**	0.81	**0.008**	0.55	**0.197**	0.47	**0.322**
	turquoise	3	-0.51	**0.022**	0.70	**0.134**	0.19	**0.957**	0.54	**0.399**	0.42	**0.649**
M-CngCTX	turquoise	3	0.54	**0.048**	0.38	**0.768**	0.42	**0.701**	0.44	**0.665**	0.60	**0.348**
M-EnCTX	turquoise	3	-0.58	**0.037**	0.57	**0.470**	0.74	**0.161**	0.40	**0.772**	0.43	**0.730**
M-Hipp	blue	2	0.57	**0.023**	0.45	**0.363**	0.77	**0.017**	0.39	**0.468**	0.39	**0.472**
	turquoise	2	0.60	**0.034**	0.33	**0.590**	0.43	**0.408**	0.29	**0.680**	0.16	**0.888**
M-OlfB	blue	2	-0.59	**0.015**	0.27	**0.720**	0.37	**0.512**	0.19	**0.844**	0.67	**0.072**
	brown	2	0.73	**0.012**	0.23	**0.784**	0.35	**0.559**	0.32	**0.614**	0.28	**0.690**
	green	1	0.62	**0.020**	0.36	**0.253**	0.14	**0.666**	0.06	**0.850**	0.16	**0.630**
	red	1	0.46	**0.132**	0.52	**0.081**	0.04	**0.897**	0.42	**0.174**	0.22	**0.495**
	turquoise	1	0.51	**0.088**	0.44	**0.158**	0.25	**0.437**	0.17	**0.594**	0.04	**0.906**
	yellow	2	-0.60	**0.033**	0.26	**0.732**	0.19	**0.843**	0.06	**0.982**	0.44	**0.385**
M.POAH	turquoise	2	0.87	**0.002**	0.43	**0.549**	0.30	**0.756**	0.03	**0.997**	0.753	**0.091**

* - number of principal components (PC) used for calculation of correlation between module and behavior trait
